# RECON gene disruption enhances host resistance to enable genome-wide evaluation of intracellular pathogen fitness during infection

**DOI:** 10.1128/mbio.01332-24

**Published:** 2024-06-28

**Authors:** Chelsea E. Stamm, Adelle P. McFarland, Melissa N. Locke, Hannah Tabakh, Qing Tang, Maureen K. Thomason, Joshua J. Woodward

**Affiliations:** 1 Department of Microbiology, University of Washington, Seattle, Washington, USA; 2 Molecular and Cellular Biology Program, University of Washington, Seattle, Washington, USA; Universite de Geneve, Geneva, Switzerland

**Keywords:** RECON, *Listeria monocytogenes*, bacterial pathogenesis

## Abstract

Transposon sequencing (Tn-seq) is a powerful genome-wide technique to assess bacterial fitness under varying growth conditions. However, screening via Tn-seq *in vivo* is challenging. Dose limitations and host restrictions create bottlenecks that diminish the transposon mutant pool being screened. Here, we have developed a murine model with a disruption in *Akr1c13* that renders the resulting RECON^−/−^ mouse resistant to high-dose infection. We leveraged this model to perform a Tn-seq screen of the human pathogen *Listeria monocytogenes in vivo*. We identified 135 genes which were required for *L. monocytogenes* growth in mice including novel genes not previously identified for host survival. We identified organ-specific requirements for *L. monocytogenes* survival and investigated the role of the folate enzyme FolD in *L. monocytogenes* liver pathogenesis. A mutant lacking *folD* was impaired for growth in murine livers by 2.5-log_10_ compared to wild type and failed to spread cell-to-cell in fibroblasts. In contrast, a mutant in *alsR,* which encodes a transcription factor that represses an operon involved in D-allose catabolism, was attenuated in both livers and spleens of mice by 4-log_10_ and 3-log_10_, respectively, but showed modest phenotypes in *in vitro* models. We confirmed that dysregulation of the D-allose catabolism operon is responsible for the *in vivo* growth defect, as deletion of the operon in the ∆*alsR* background rescued virulence. By undertaking an unbiased, genome-wide screen in mice, we have identified novel fitness determinants for *L. monocytogenes* host infection, which highlights the utility of the RECON^−/−^ mouse model for future screening efforts.

**IMPORTANCE:**

*Listeria monocytogenes* is the gram-positive bacterium responsible for the food-borne disease listeriosis. Although infections with *L. monocytogenes* are limiting in healthy hosts, vulnerable populations, including pregnant and elderly people, can experience high rates of mortality. Thus, understanding the breadth of genetic requirements for *L. monocytogenes in vivo* survival will present new opportunities for treatment and prevention of listeriosis. We developed a murine model of infection using a RECON^−/−^ mouse that is restrictive to systemic *L. monocytogenes* infection. We utilized this model to screen for *L. monocytogenes* genes required *in vivo* via transposon sequencing. We identified the liver-specific gene *folD* and a repressor, *alsR*, that only exhibits an *in vivo* growth defect. AlsR controls the expression of the D-allose operon which is a marker in diagnostic techniques to identify pathogenic *Listeria*. A better understanding of the role of the D-allose operon in human disease may further inform diagnostic and prevention measures.

## INTRODUCTION

*Listeria monocytogenes* (Lm) is a gram-positive, facultative pathogen and is the main agent responsible for the food-borne disease listeriosis. Lm is ubiquitous in the environment and can grow under conditions used in food processing such as high salt and low temperatures (1). Asymptomatic ingestion of contaminated food by immune-competent individuals is predicted to occur several times a year (2). However, exposure among vulnerable populations such as pregnant people, the immunocompromised, and the elderly frequently causes invasive disease with severe morbidity and mortality (3, 4). In these individuals, the liver and spleen are replicative niches where Lm grows to high numbers and can further disseminate across the blood-brain or placental barriers to cause severe disease (5–7). Thus, understanding the genetic requirements for Lm to replicate in the liver and spleen may provide novel treatment targets for prevention of severe disease.

Lm pathogenesis is facilitated by its ability to infect multiple cell types intracellularly. The Lm intracellular life cycle is well-established and relies on virulence genes clustered together on the Listeria Pathogenicity Island-I (LPI), which are under the control of PrfA (4). Functions for endosomal escape, actin-based motility, and cell-to-cell spread are all encoded by genes on the LPI (8). In addition to virulence gene expression, intracellular replication requires metabolic reprogramming to withstand nutrient restriction in the cytosol (9, 10). While much of the intracellular life cycle of Lm and virulence properties of the LPI were comprehensively determined using cell culture methods, discrepancies exist among *in vitro* models. For example, the actin nucleating protein ActA is required for development of plaques in epithelial cells, but it is dispensable for intracellular replication in macrophage infections (11). In addition, binding of the internalin InlB to non-phagocytic cells in cell culture is promiscuous and in opposition to observed tropism *in vivo* where it is not required for Lm intestinal barrier crossing but for dissemination to peripheral organs (12, 13). Finally, it is clear that homogenous cell lines cannot replicate the complex host-pathogen interactions inherent in a whole organism (14). Therefore, establishing the genetic requirements of Lm survival *in vivo* is of utmost interest.

Transposon mutagenesis has long facilitated genotype-to-phenotype discoveries in bacteria but, until recently, was hampered by low-throughput screening methods. Transposon sequencing (Tn-seq) leverages next-generation sequencing to assess the fitness of a pool of transposon-mutagenized bacteria under a given selection in a high-throughput manner (15, 16). Tn-seq technologies have greatly increased the genetic landscape that can be assessed in a single experiment and have been used to establish essential genes and genes required for host survival for several pathogenic bacteria of interest including *Streptococcus pneumoniae* (15), *Vibrio cholerae* (17), *Acinetobacter baumannii*, (18) and *Staphylococcus aureus* (19, 20). However, the technical challenges associated with utilizing Tn-seq *in vivo* including bottlenecks of the transposon pool that arise from host restrictions and the potential cost-prohibitive nature of screening in animals limits Tn-seq broader usage (16). For example, the low founding population in mouse peripheral organs at a lethal dose 50 (LD50) of Lm restricts the total number of transposon mutants that can be delivered to each animal (21). Here, we describe the development of a mouse model lacking a single protein, RECON^−/−^, that can survive increased bacterial burdens. Using Lm as a model pathogen, we show that this mouse is a viable model for assessing bacterial fitness factors *in vivo* via Tn-seq. Finally, we validate two hits, *folD* and *alsR* in wild-type mice and characterize their roles in Lm *in vivo* pathogenesis.

## RESULTS

### Generation of RECON-deficient mice

**Fig 1:**
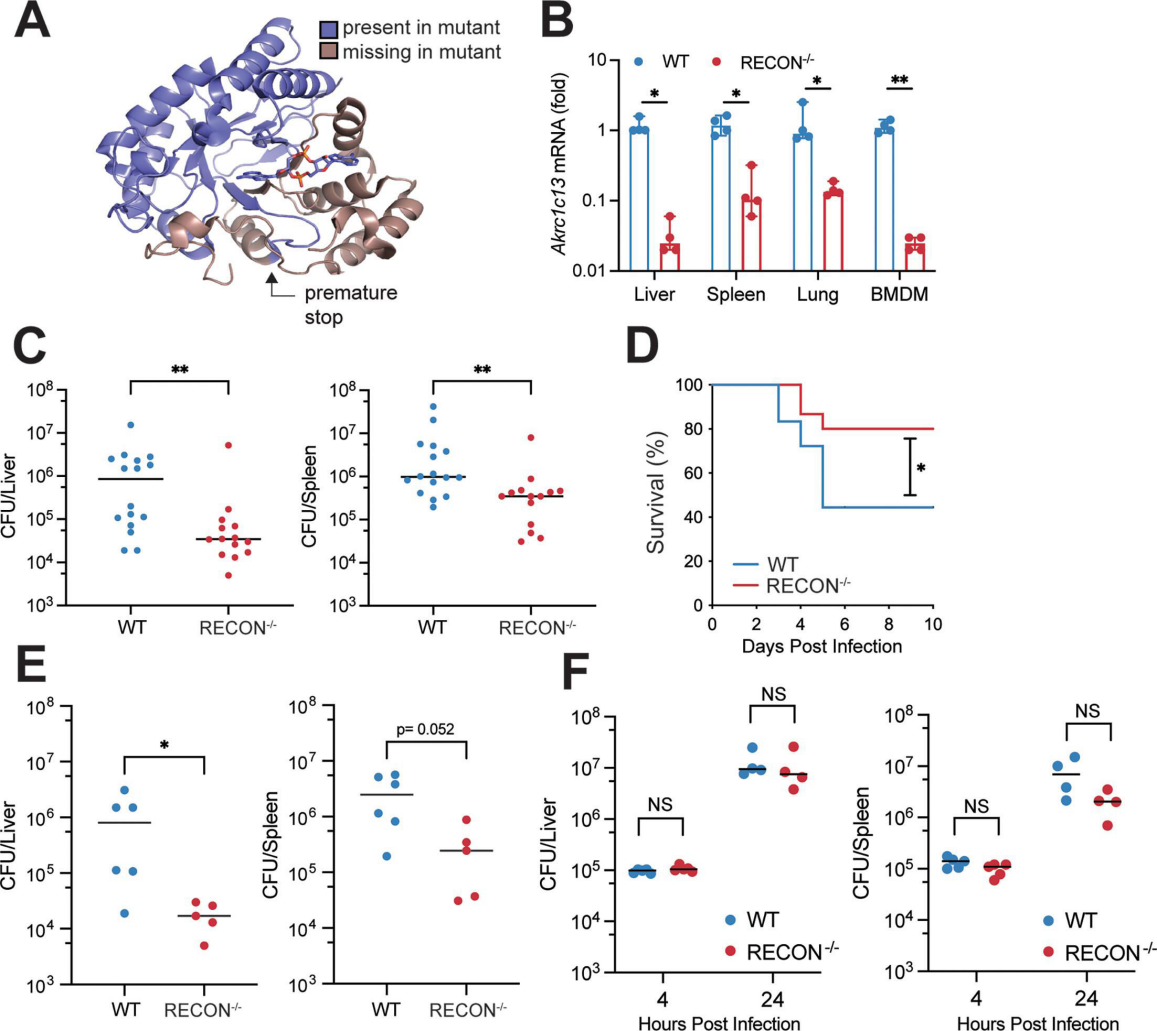
Loss of RECON leads to bacterial clearance. (**A**) RECON protein structure with the region missing in the truncated mutant protein colored in brown. Truncation destroys the active and c-di-AMP binding sites. (**B**) qRT-PCR analysis on mRNA from mouse liver, spleen, lung, and BMDM isolated from WT and RECON^−/−^ mice (*n* = 4 for each genotype). The endogenous control gene was *Hprt* and data are normalized to the mean value of *Akr1c13*/*Hprt* within each WT tissue. Median values are indicated by a bar. Statistical significance was determined by an unpaired Student’s *t-*test with *P < 0.05, **P < 0.01. (**C**) WT (*n* = 16) or RECON*^−/^*^−^ (*n* = 14) mice were injected IP with 1 × 10^4^ CFU Lm. CFU were enumerated from liver (left) and spleen (right) 72 h post infection. Data are combined from two independent experiments. (**D**) Kaplan-Meier survival curve shown for WT (*n* = 18) or RECON^−/−^ (*n* = 15) mice injected IP with 1 × 10^6^ CFU Lm. Survival was analyzed by log-rank test with *P < 0.05. (**E**) WT (*n* = 6) or RECON*^−/^*^−^ (*n* = 5) mice were injected IV with 1 × 10^4^ CFU Lm. CFU were enumerated from liver (left) and spleen (right) 72 h post infection. Data are representative of two independent experiments. (**F**) WT or RECON*^−/^*^−^ mice were injected IV with 2 × 10^6^ CFU Lm. CFU were enumerated from liver (left) and spleen (right) at 4 (*n* = 5/group) or 24 h (*n* = 4/group) post infection. For CFU enumeration in C, E and F, statistical significance was assessed by Mann-Whitney U-test with *P < 0.05, **P < 0.005.

We previously identified a murine aldo-keto reductase (AKR) encoded by *Akr1c13* that binds the bacterial second messenger cyclic diadenosine monophosphate (c-di-AMP). Binding of the protein RECON to c-di-AMP inhibits its enzymatic activity and results in augmented nuclear factor kappa B (NF-κB) activation (22). To better understand the role of RECON in the immune response to bacterial infection, we used CRISPR/Cas9 to mutagenize *Akr1c13* in mice. *In vitro* transcribed sgRNA and Cas9 mRNA were microinjected into C57BL/6J embryos and implanted into pseudo-pregnant female mice (Fig. S1A). We selected a heterozygous male founder that had a single G insertion in exon 6 of *Akr1c13* (Fig. S1A and B) and bred the line to homozygosity. The single nucleotide insertion in the *Akr1c13* coding sequencing caused a frameshift resulting in a premature stop codon after residue 200, which truncates the protein prior to the active site and c-di-AMP binding site (Fig. 1A; Fig. S1B). Due to close homology with other murine AKRs, there is not a RECON-specific antibody. However, we were able to analyze the expression of *Akr1c13* in liver, spleen, lung, and bone marrow-derived macrophages (BMDM) via quantitative RT-PCR (qRT-PCR). We observed that RNA levels of *Akr1c13* decreased in the CRISPR/Cas9-targeted mice compared to wild-type mice (Fig. 1B), likely due to nonsense-mediated decay of unstable mRNAs (23). Therefore, we concluded these mice are deficient in RECON (RECON^−/−^) as a result of a frameshift mutation that reduces mRNA stability and abolishes expression of the full-length protein.

### RECON-deficient mice are more resistant to bacterial infection

As we had previously demonstrated that altering expression levels of RECON affected the intracellular survival of Lm in macrophages but not hepatocytes (22), we sought to determine how loss of RECON would influence the outcome of Lm infection *in vivo*. To that end, we infected WT or RECON-deficient littermates with Lm by intraperitoneal (IP) injection and determined bacterial burden at 72 h post infection (hpi). Bacterial colony forming units (CFU) collected from livers and spleens of RECON^−/−^ mice were significantly lower than in wild-type littermates (Fig. 1C). To determine if this decrease in CFU resulted in protection for RECON^−/−^ mice, we again infected Lm intraperitoneally and monitored murine survival over the course of 10 days. Consistent with the decreased bacterial burden, RECON^−/−^ mice exhibited increased survival to Lm infection compared to wild-type mice (Fig. 1D). Next, we asked if route of infection influenced Lm restriction in RECON^−/−^ mice. Thus, we infected WT or RECON^−/−^ mice via an intravenous (IV) route and determined CFU in livers and spleens at 72 hpi. We observed that similar to IP injection, loss of RECON resulted in decreased Lm burden in livers of spleens (Fig. 1E). Finally, to understand the dynamics of acute infection in this model, we infected WT or RECON^−/−^ mice with a high dose (2 × 10^6^ CFU/mouse) via IV and enumerated CFU from livers and spleens at 4 and 24 hpi. We observed no significant difference in the Lm burden in either organ between genotypes at 4 or 24 hpi, though the CFU in RECON^−/−^ spleens at 24 hpi began showing signs of restriction compared to WT (Fig. 1F). Together, these data demonstrate that loss of RECON promotes faster bacterial clearance during systemic challenge, starting after 24 hpi and that bacterial restriction is protective.

### RECON-deficient mice are a viable model for *in vivo* screening by transposon sequencing

As previously discussed, technical limitations impede the use of Tn-seq *in vivo*. However, since RECON^−/−^ mice were more resistant to Lm infection (Fig. 1C through E), we hypothesized that this mouse genotype would survive acute infection with a high-dose inoculum, allowing for sufficient bacterial replication at early time points and be a powerful animal model to assess bacterial fitness using Tn-seq *in vivo*.

**Fig 2:**
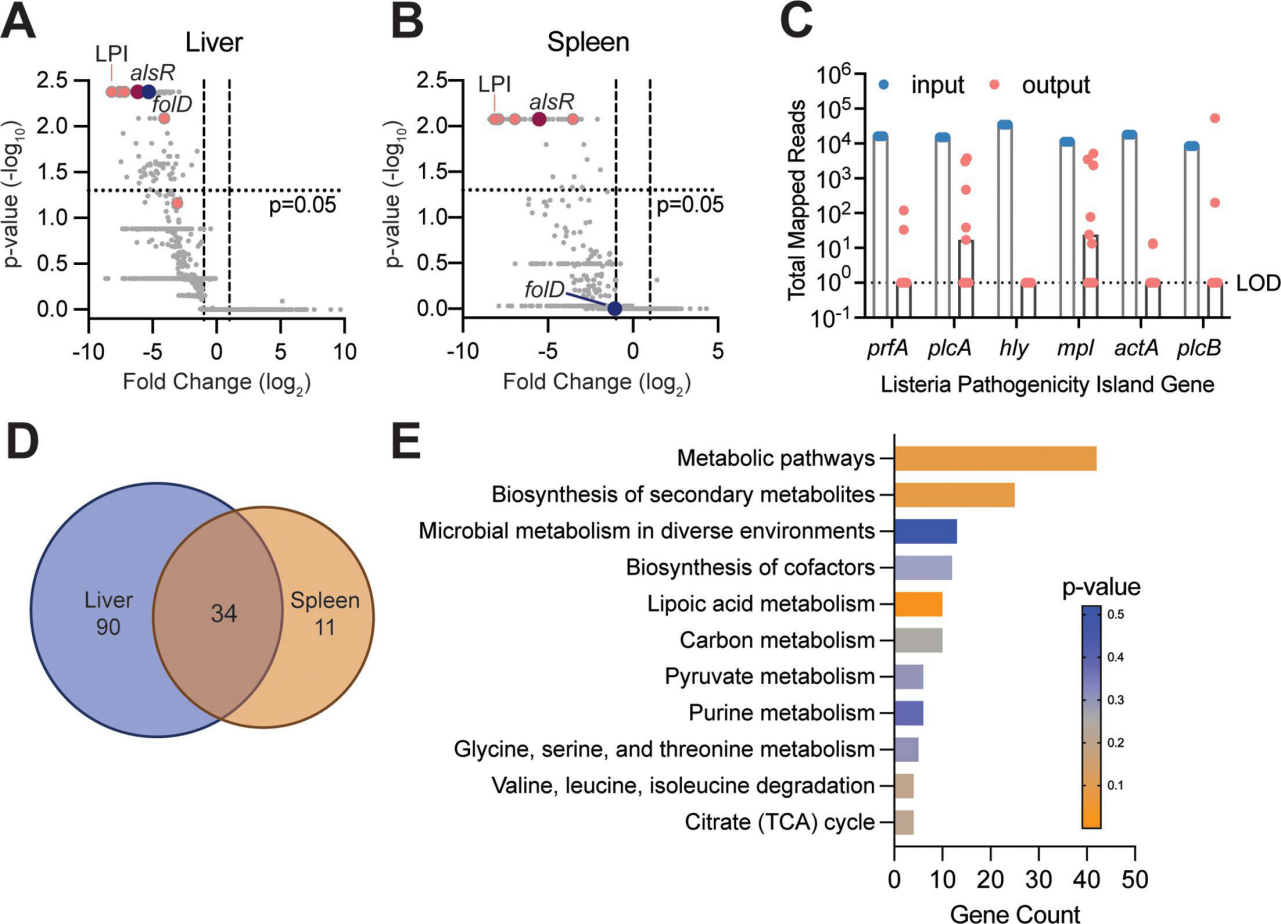
Identification of Lm genes required for murine infection via TN-Seq. (**A, B**) Volcano plots of mapped Lm genes from livers (**A**) and spleens (**B**) of infected RECON^−/−^ mice (*n* = 9). Each dot represents a single gene and vertical lines denote twofold change. The LPI is highlighted in pink and two hits, *alsR* and *folD,* are indicated by red and blue, respectively. (**C**) Total mapped reads for each gene in the LPI in the input library compared to the liver output library. Data for each mouse are plotted as a dot and the median is indicated by the bars. LOD, limit of detection. (**D**) Venn diagram comparing the 124 genes required for Lm infection of the liver with the 45 genes required in the spleen. (**E**) KEGG pathway enrichment analysis of all hits.

**Fig 3:**
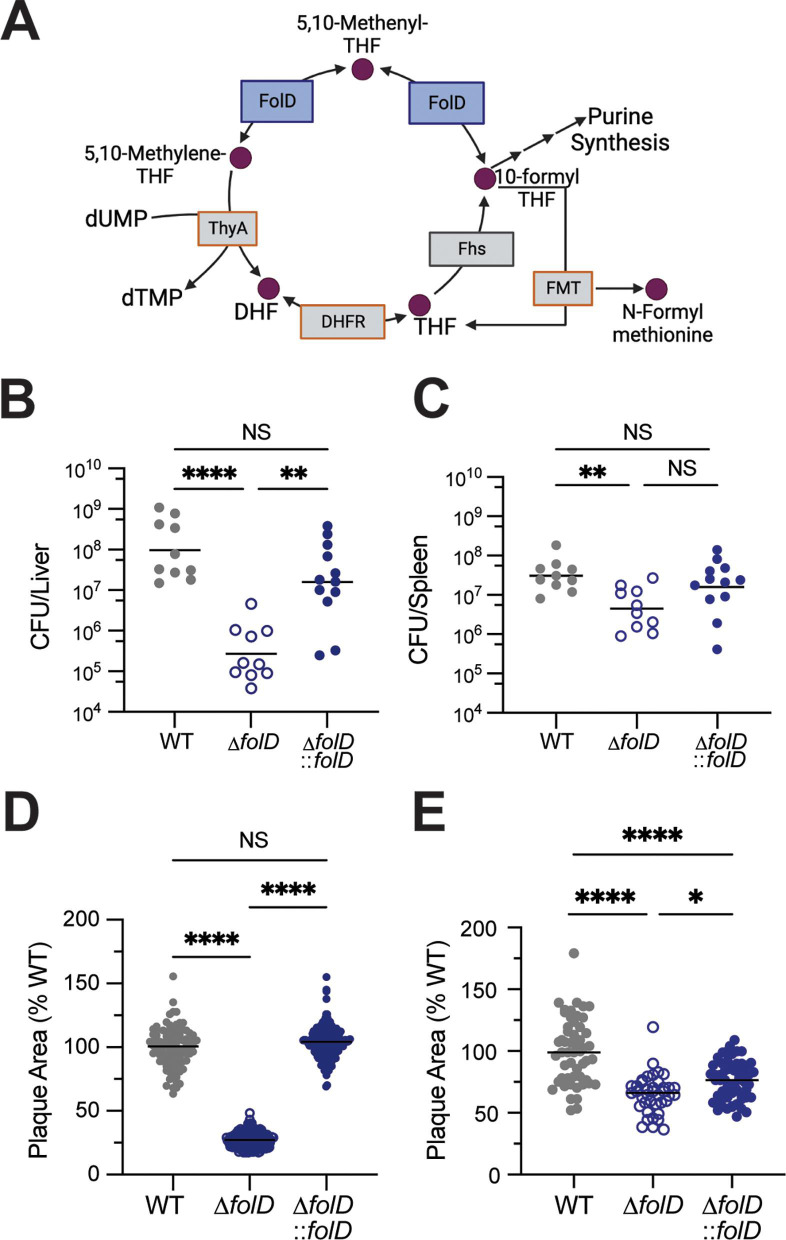
The folate metabolism gene *folD* contributes to Lm fitness in the murine liver. (**A**) Schematic of folate cycle in Lm. FolD is indicated in blue. Genes outlined in orange were essential in our library. ThyA, thymidylate synthase; DHFR, dihydrofolate reductase; Fhs, formyltetrahydrofolate synthetase; FMT, methionyl-tRNA formyltransferase. (**B, C**) CFU harvested from livers (**B**) and spleens (**C**) of WT mice infected WT (*n* = 10), ∆*folD* (*n* = 10), or ∆*folD::folD* (*n* = 12) Lm via IV with 1 × 10^5^ CFU for 72 h. Data are combined from two independent experiments. **P < 0.01, ****P < 0.0001 by Kruskal-Wallis test. (**D, E**) Plaque area measured in fibroblasts (**D**) or hepatocytes (**E**) that were infected with WT, ∆*folD,* or ∆*folD::folD* Lm, stained with neutral red and analzyed after 60 h. Data are combined from triplicate wells and are representative of two independent experiments. *P < 0.05, ****P < 0.0001 by nonparametric analysis of variance.

**Fig 4:**
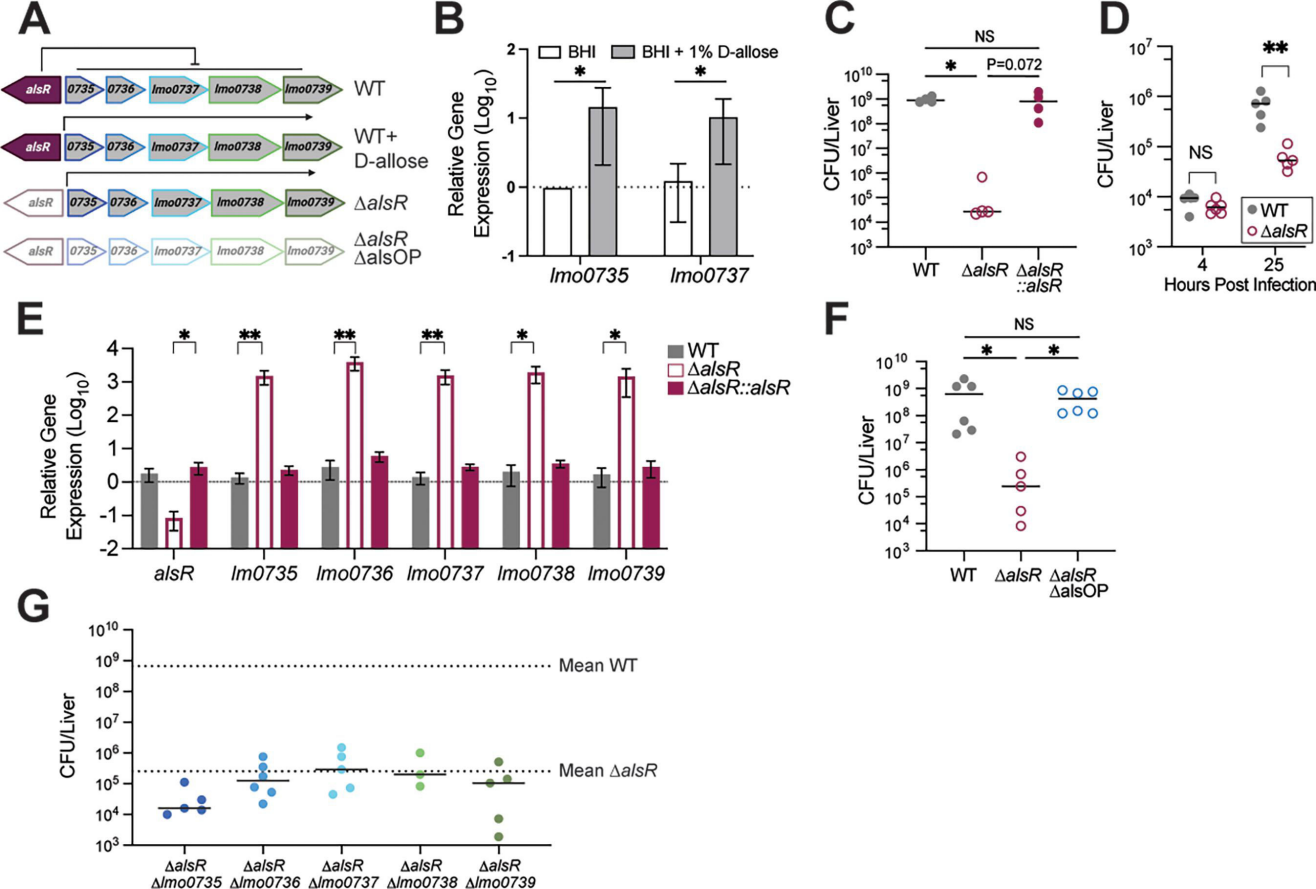
Dysregulation of the Lm D-allose operon leads to decreased virulence in mice. (**A**) Schematic of the ALO locus in Lm and expected expression under indicated conditions. (**B**) Relative gene expression of *lmo0735* and *lmo0737* in Lm grown to mid-log phase in BHI or BHI supplemented with 1% D-allose. Data are normalized to BHI for each gene. *P < 0.05 by Mann-Whitney analysis. (**C**) CFU harvested from livers of WT mice infected via IV with 1 × 10^5^ CFU of WT, ∆*alsR*, or ∆*alsR::alsR* Lm (*n* = 4/group) for 72 h. (**D**) CFU harvested from livers of WT mice infected via IV with 1 × 10^5^ CFU of WT or ∆*alsR* Lm for 4 (*n* = 6/group) or 25 h (*n* = 5/group). **P < 0.01 by Mann-Whitney analysis. (**E**) Relative gene expression of the ALO in WT, ∆*alsR*, and ∆*alsR::alsR* Lm grown in BHI until mid-log phase. Data are plotted as fold change over WT for each gene. *P < 0.05, **P < 0.01 by one way analysis of variance of ∆∆C_t_ values. (**F**) WT mice were infected via IV with 1 × 10^5^ CFU of WT (*n* = 6), ∆*alsR* (*n* = 5), or ∆*alsR*∆alsOP (*n* = 6) Lm. CFU were harvested from livers at 72 hpi. (**G**) WT mice were infected via IV with 1 × 10^5^ CFU of the indicated strains (*n* = 3 for ∆*alsR*∆*lmo0738* and *n* = 5 for all other strains) and CFU were enumerated from livers at 72 hpi. Mean WT and ∆*alsR* CFU were calculated from at least four independent experiments. CFU for individual gene mutants in ∆*alsR* background were collected on independent days. Experiments in D, F, and G were performed once. CFU in C and F were analyzed by Kruskal-Wallis test with *P < 0.05.

To that end, we generated a *mariner*-based transposon library in Lm strain 10403S containing 17,000 mutants and infected nine RECON^−/−^ mice with 2.4 × 10^6^ CFU/mouse via IV. At this dose, we observed no significant difference in organ colonization by 4 hpi compared to WT (Fig. 1F). To achieve sufficient bacterial growth *in vivo*, we allowed the infection to proceed for 34 h. We isolated, on average, 1.6 × 10^5^ CFU and 2.0 × 10^5^ CFU from livers and spleens, respectively. We then isolated genomic DNA and prepared libraries for massive parallel sequencing of the transposon insertion junctions. Then, we used PATRIC (24) to map transposon junctions and determine genes that were depleted in livers or spleens of mice compared to the input transposon library (Fig. 2A and B, respectively). The full table of mapped transposon junction reads can be found in the supplemental material. We identified 124 genes in the liver and 45 genes in the spleen with at least a twofold decrease in reads and an adjusted *P*-value less than 0.05 compared to the input (Tables S1 and S2, respectively). Of these, 34 genes were required for growth in both organs (Fig. 2D). Importantly, reads in *prfA*, *hly*, *actA,* and *plcB* which are components of the LPI, and required for Lm virulence (4), were absent in all but two liver samples (Fig. 2C). In fact, the LPI genes were among the most significantly depleted genes in both organs (Fig. 2A and B). Of the 135 genes required for host survival, 51 have previously been implicated in virulence (Tables S1 and S2). In addition, we have validated four additional genes below, two of which are liver-specific (Fig. S2; Fig. 3 and 4). Taken together, these observations confirm the robustness of our screening approach.

We identified novel hits for further validation. Two components of an ABC transporter (*eslA* and *eslB*) important for lysozyme resistance were significantly depleted in the liver. We also observed a significant depletion of *eslA* in spleens. Previous studies with individual mutants of *eslA* or *eslB* observed no defect in plaque size (25), or growth in macrophages or flies (26). Because both *eslA* and *eslB* were required for growth in the liver, we sought to test whether a double deletion mutant would be impaired *in vivo*. Thus, we generated an in-frame deletion of both genes (∆*eslAB*) in Lm 10403S and infected WT C57BL/6 mice via IV and harvested CFU from livers and spleens at 72 hpi. We observed a significant decrease in ∆*eslAB* CFU in both livers and spleens compared to WT (Fig. S2A). Consistent with previously published data, we observed no defect in the ability of ∆*eslAB* to replicate in BMDM or form plaques (Fig. S2B and C). Next, we validated *lmo0897*, which is positioned adjacent to the sigma B operon and was depleted over 25-fold in the liver. We infected mice IV with WT Lm or a *lmo0897* mutant (∆*lmo0897*) and measured CFU from livers and spleens 72 hpi. Consistent with the results of our screen, we observed no difference in CFU collected from spleens of mice infected with WT or ∆*lmo0897* bacteria (Fig. S2D). However, we did observe a small but not significant difference in ∆*lmo0897* CFU isolated from livers (Fig. S2D). Loss of *lmo0897* had no effect on the ability of Lm to grow in BMDM or form plaques (Fig. S2E and F). These validations suggest that our screen successfully identified *in vivo*-specific genes as well as organ-specific factors.

Finally, we analyzed the genes required for growth *in vivo* by pathway enrichment analysis (Fig. 2E). Lm is an auxotroph for lipoic acid and genes required for lipoic acid acquisition were the most significantly enriched. However, the majority of enriched genes clustered into the general metabolic pathways group and could be further broken down into genes enriched for energy production (carbon metabolism, pyruvate metabolism, and citrate [TCA] cycle). In addition, genes required for *in vivo* survival were enriched in pathways for the synthesis of secondary metabolites, essential cofactors, purines, and amino acids. Finally, nearly 50% of the genes required for *in vivo* survival were not significantly enriched in KEGG pathways including a proportion of the genes (9%) that remained unannotated and could not be mapped to any pathway. Taken together, these data confirm that core metabolic processes are indispensable for growth in the murine host and suggests that there are genes with *in vivo*-specific functions yet to be elucidated.

### The folate metabolism gene *folD* contributes to *L. monocytogenes* fitness in the murine liver

The primary niches for Lm in the murine liver and spleen are distinct, with hepatocytes being the main cell type for Lm intracellular growth in the liver (27, 28). As we had previously observed mutants with varying degrees of growth deficit in murine livers (Fig. S2A and D), we sought to better understand the genetic requirements for liver colonization. One gene, *folD* (*lmo1360*), which encodes the bifunctional enzyme 5,10-methylene-tetrahydrofolate dehydrogenase/5,10-methylene-tetrahydrofolate cyclohydrolase, was depleted nearly 40-fold in livers of mice but was not significantly changed in the spleens (Fig. 2A and B; Table S1). The bioactive tetrahydrofolates produced by FolD in the one-carbon cycle are used to synthesize deoxythymidine monophosphate, purines, and the initiator amino acid N-formylmethionine (Fig. 3A) (29, 30). To validate the organ specificity of *folD*, we deleted it from the Lm 10403S chromosome, infected WT C57BL/6 mice via IV, and harvested CFU from livers and spleens at 72 hpi. We recovered over two logs fewer ∆*folD* bacteria than WT from livers of mice, which could be rescued by constitutive expression of *folD* from a separate chromosomal locus (∆*folD::folD*) (Fig. 3B). In contrast, ∆*folD* growth in the spleen was only reduced by half a log compared to WT and was similarly rescued by complementation (Fig. 3C). We confirmed the virulence defect of ∆*folD* in the murine liver was not due to an overall replication defect by measuring growth over time in brain heart infusion (BHI) and *Listeria* defined (31) minimal media (MM) and observed no difference in growth between WT and ∆*folD* in either condition (Fig. S3A and B).

Lm replicates to high CFU in the liver in part due to its ability to spread cell-to-cell, without encountering the extracellular space (32, 33). In order to determine if *folD* is required for cell-to-cell spread, we infected monolayers of rat fibroblasts with WT, ∆*folD,* or ∆*folD::folD* Lm for 60 h and measured the size of plaques formed by each strain. The area of plaques formed by ∆*folD* was nearly 70% smaller than that of WT, whereas the plaque size of ∆*folD::folD* bacteria was comparable with WT (Fig. 3D). We saw similar but less drastic results when we measured plaque size in immortalized murine hepatocytes (Fig. 3E). In contrast, ∆*folD* replicated to equal levels as WT in both naïve and Interferon gamma (IFN-γ)-stimulated BMDMs (Fig. S3C and D). In all, these data suggest that the requirement for folates produced by FolD is cell type specific and contributes to growth in the murine liver.

### Dysregulation of the *L. monocytogenes* D-allose utilization operon leads to decreased virulence in mice

D-allose is a C3 epimer of glucose which is found in low quantities in nature, primarily in plants (34). D-allose and its derivative D-allulose are gaining favor as safe, low-caloric sweeteners (34, 35). In addition, D-allose is used in a novel enrichment broth to preferentially culture pathogenic *Listeria* (*L. monocytogenes* and *Listeria ivanovii*) from environmental samples (36). A six-gene operon *lmo0734–lmo0739* confers the ability for Lm to grow on D-allose as a sole carbon source (Fig. 4A) (37). We confirmed that addition of 1% D-allose to rich media was sufficient to induce components of the D-allose operon (ALO) in Lm 10403S (Fig. 4B). Thus, we were intrigued to find that the transcriptional regulator of the ALO, *alsR* (*lmo0734*), was depleted over 70-fold in the liver and 45-fold in the spleen (Fig. 2A and B; Tables S1 and S2). To validate the role of *alsR* in Lm *in vivo* pathogenesis, we used allelic exchange to make an in-frame deletion of *alsR,* infected WT C57BL/6 mice via IV, and enumerated CFU from livers and spleens 72 hpi. We observed a significant reduction in ∆*alsR* CFU in both livers (4.5 log_10_) and spleens (2.5 log_10_) compared to WT (Fig. 4C; Fig. S4A). We complemented *alsR* back to a separate chromosomal locus with its native promotor (∆*alsR::alsR*) and observed that these bacteria grew to the same levels as WT in both the liver and spleen (Fig. 4C; Fig. S4A). To determine if ∆*alsR* bacteria were impaired prior to organ colonization, we repeated the IV infection but collected bacteria from livers and spleens at 4 and 25 hpi. At 4 hpi, we counted similar numbers of WT and ∆*alsR* CFU isolated from the livers of mice (Fig. 4D) but observed a small decrease in ∆*alsR* bacteria isolated from spleens compared to WT (Fig. S4B). In contrast, there were significantly fewer ∆*alsR* CFU in both organs at 25 hpi (Fig. 4D; Fig. S4B). When observed over time, the growth of ∆*alsR* slowed after 4 hpi compared to WT (Fig. S4C) and suggests that the ∆*alsR* growth defect occurs after organ colonization. Interestingly, although ∆*alsR* survival was impaired significantly *in vivo* as early as 25 hpi, we observed no difference in intracellular replication in BMDM between WT and ∆*alsR* (Fig. S4D) and only a modest decrease in plaque size (Fig. S4E).

Because AlsR is a predicted repressor, we measured ALO expression in the mutant by qRT-PCR. We confirmed that in the absence of *alsR*, all genes in the operon were upregulated in BHI without the presence of D-allose (Fig. 4E). The upregulation of the ALO in the absence of alsR was 2-log_10_ higher than that observed in WT cells supplemented with D-allose (Fig. 4B). Importantly, this overexpression did not result in *in vitro* growth defects in BHI compared to WT Lm (Fig. S4F). Additionally, since phosphoenolpyruvate-dependent phosphotransferase system (PTS) sugars suppress PrfA-dependent transcription (38), we explored whether aberrant expression of the ALO could influence virulence gene expression. To test this, we generated a strain of ∆*alsR* that contains a constitutively active mutant of PrfA (∆*alsR* PrfA* [39]) and infected mice via IV. PrfA* did not rescue the ∆*alsR* growth defect in either livers or spleens of mice at 72 hpi (Fig. S4G and H), suggesting ∆*alsR* does not affect virulence gene expression. Thus, because AlsR is not predicted to regulate other genes (RegPrecise) (40), we postulated that dysregulation of the ALO is the direct cause for the ∆*alsR in vivo* growth defect. To test this, we deleted the entire operon including *alsR* from the chromosome (∆*alsR*∆alsOP) and infected mice via IV. In contrast to ∆*alsR*, ∆*alsR*∆alsOP bacteria replicated to WT levels in the liver and spleen at 72 hpi (Fig. 4F; Fig. S4I), suggesting that dysregulation of the ALO is responsible for the impaired growth of ∆*alsR*. To determine if overexpression of a single gene in the ALO was sufficient to produce the ∆*alsR in vivo* growth defect, we deleted each gene individually in the ∆*alsR* background, performed IV infections of mice and enumerated CFU at 72 hpi. Fewer CFU were recovered from livers of mice infected with ∆*alsR*∆*lmo0735* compared to ∆*alsR* alone (Fig. 4G; Fig. S4J). In contrast, ∆*alsR*∆*lmo0736*, ∆*alsR*∆*lmo0737*, ∆*alsR*∆*lmo0738,* and ∆*alsR*∆*lmo0739* CFU were similar to ∆*alsR* CFU (Fig. 4G; Fig. S4K through N). In all, no single ALO gene deletion rescued the growth of ∆*alsR* to WT or ∆*alsR*∆alsOP levels.

## DISCUSSION

A genome-wide understanding of the Lm fitness determinants in the host has yet to be explored. Here, we generated a murine model, RECON^−/−^, that sustained high-dose, acute bacterial infection. We used this host model to perform an *in vivo* Tn-seq of the human pathogen Lm and successfully identified both previously known and novel virulence determinants.

Mice lacking RECON restricted Lm systemic infection and consequently were protected from Lm pathogenesis. We previously observed that loss of RECON augmented immune signaling via NF-κB (22). This suggests that RECON^−/−^ mice are poised to respond quicker to bacterial infection which contributes to the bacterial restriction we observed in the context of Lm infection (Fig. 1C and D; Fig. S1C). As c-di-AMP binding to RECON inhibits enzyme activity (22), the immune environment of the RECON^−/−^ mouse represents a c-di-AMP bound state and suggests that build-up of a RECON substrate influences cross-talk with the immune system and contributes to bacterial restriction. Prostaglandins and the α,β-unsaturated aldehydes produced from lipid peroxidation are intriguing substrate possibilities for RECON (41, 42). Indeed, 4-hydroxynonenal influences NF-κB-dependent transcription by covalently modifying an upstream ubiquitin-conjugating enzyme Ube2V1 (43). Work is ongoing to define the interaction of RECON (and its substrates or products) on the NF-κB signaling axis in RECON^−/−^ mice. However, we exploited the increased bacterial clearance at late time points of the RECON^−/−^ mouse to perform *in vivo* Tn-seq of Lm. A third of the genes we identified were previously implicated in Lm virulence in the host (Tables S1 and S2), which confirms the usefulness of RECON^−/−^ as a screening model. However, we cannot rule out that some of the novel hits from our screen are specific to the predicted heightened immune response in our model.

We have completed a genome-wide screen for Lm *in vivo* survival. Excitingly, two-thirds of the genes required for Lm growth in either livers or spleens of mice have not been implicated in virulence prior to our screen. This provides a wealth of novel genetic targets to explore for disease prevention and treatment. However, the Tn library we used was not saturated and short genes were likely underrepresented. Indeed,after accounting for essential genes from a recent screen (10), our library is still missing 299 genes, or about 11% of the Lm open reading frames (ORF) (Tables S6 and S7). This suggests that there may be more genes important for *in vivo* infection still to be discovered. In addition, the analysis we performed only mapped ORF to the reference genome and did not take into account placement of small, non-coding RNAs (ncRNA) which are known to influence Lm virulence (44). For example, the multicopy ncRNA *lhrC5* is positioned downstream of *lmo0947*, which was depleted in our screen (45). Deletion of all five *lhrC* ncRNAs decreases Lm survival in macrophages (46). It is possible that Tn disruption of *lmo0974*, or similarly juxtaposed ORF-ncRNA pairs, decreased Lm survival due to altered ncRNA expression and subsequent target regulation. Finally, 9% of the genes required for Lm *in vivo* growth are unannotated. Further characterization of this unannotated subset is needed to understand the function of these genes in Lm physiology and their possible role as host virulence determinants.

Recently, a Tn-seq was performed for Lm in J774 macrophages (10). Our screen identified nearly half (20/42) of the genes required for growth in macrophages which shows our methods were complementary. The lack of a total overlap between the two datasets is largely a result of our library density, as previously mentioned. For the remaining 22 genes required for macrophage growth, six were not represented in our input library and 10 were also depleted in our screen but did not reach statistical significance. However, our screening *in vivo* led to identification of a larger subset of genes (135 compared to 42). One explanation for this is that many single gene deletions do not have a macrophage growth defect. Thus, screening *in vivo* allowed us to identify factors such as the known virulence genes *actA* (11), *gshF* (47), and *iap* (p60) (48), the lysozyme resistance ABC transporter *eslAB* (26), the redox-responsive regulator *rex* (49) and *alsR*, which are all absent from the J774 data set. In addition, although both screening conditions highlighted the requirement for lipoic acid uptake, and *de novo* purine and menaquinone synthesis, other nutrient restrictions only became apparent through screening *in vivo*. For example, the uptake genes for thiamine (*lmo1429*) and biotin (*lmo0598*) were only required in our *in vivo* screen despite being required for host infection (50, 51). Thus, by screening *in vivo,* we identified genes involved in pathways that cell culture cannot recapitulate.

We separately characterized genes required for Lm survival in murine spleens and livers and identified 90 genes uniquely required for growth in the liver. It is likely that this number is an overrepresentation considering over 30 of the genes were also depleted in spleens at least twofold but did not reach statistical significance. For some, such as the flavin metabolism gene *ribF*, and a menaquinone synthesis gene *menA*, the *P*-value of the spleen samples was just above our cutoff of *P* < 0.05, emphasizing the requirement of these essential cofactors for *in vivo* growth. Interestingly, while glycerol utilization genes (e.g., *pgm*, and *pfkA*) were required in both organs, there seems to be a liver-specific energy requirement not found in the spleen. The first indication for this is that nine membrane transport mechanisms were required in the liver only, including several genes required for ion transport such as *pstB* and its regulator *phoU*, *lmo1849,* and *lmo0366*. In addition, we also identified several genes for ethanolamine usage (*eutC*, *eutE,* and *lmo1161*) which can contribute to acetyl-CoA production (52) or act as a nitrogen source (53) from host phosphatidylethanolamine. Finally, we characterized the liver-specific requirement for the folate enzyme FolD which was recently described as an N-formylmethionine-dependent phenotype by Feng and colleagues (54). We observed a larger growth defect for ∆*folD* in livers, likely because we used a different mouse strain and harvested CFU at a later time point. That we independently identified *folD* and observed similar phenotypes again emphasizes the universal benefit of the RECON^−/−^ model for bacterial fitness evaluation.

The D-allose utilization repressor gene *alsR* was required for Lm survival in both livers and spleens of mice but not in *in vitro* cell culture models. Overexpression of the ALO in the ∆*alsR* background was sufficient to cause the *in vivo* growth defect, which reinforces the importance of tight metabolic regulation in the host (55, 56). Indeed, the loss of two other regulators, *lmo0020* and *lmo1253,* that control PTS^Man^-1 and trehalose metabolism, respectively, also impaired Lm survival in the liver (Table S1). It is feasible the aberrant overexpression of the ALO in ∆*alsR* results in depletion of an important metabolite, accumulation of a toxic intermediate, or an overall energy burden on the cells which is only detrimental in the nutrient-limiting environment *in vivo*. Two lines of evidence favor the latter of these possibilities. First, while we cannot exclude the possibility that the concerted effort of two or more enzymes produce by the ALO creates a toxic metabolite or deplete an important metabolite, the observation that no single deletion within the ALO rescue ∆*alsR* most likely suggests multiple gene overexpression is adversely impacting survival. Second, ALO transcripts are increased 1,000-fold in ∆*alsR* compared to WT bacteria and are approximately 100-fold higher than WT Lm grown with the AlsR inducer D-allose. These observations are reminiscent of the impacts of the constitutively active PrfA* allele that exhibits comparable levels of transcriptional upregulation of the LPI-1 (57). In this case, aberrant gene regulation results in comparable levels of growth relative to WT bacteria *in vivo*, but orders of magnitude of competitive growth defect relative to WT bacteria grown in standard culture media (58). However, expression of the ALO in the environment likely benefits Lm to outcompete other microbes in the community and this growth advantage is exploited to enrich Lm during sampling studies (36). Interestingly, the ALO is only encoded by Lineage II Lm and is used to distinguish Lineage II from Lineage I and III strains in PCR-based serotyping methods (59). Since we were able to delete the ALO and observed WT growth in the host, it is not surprising that Lineage I strains, which are frequently associated with human listeriosis outbreaks (4), have lost the operon. In contrast, some Lineage I serotypes have acquired the ALO (60, 61), which not only calls into question the accuracy of using the ALO as a serotype marker (62) but also suggests that acquisition of the operon is beneficial to Lm prior to human colonization, such as during contamination of food processing environments. It is intriguing to speculate how the ∆*alsR* phenotype could be utilized to prevent disease. Since AlsR is predicted to relieve repression upon binding D-allose, identification of a non-convertible, synthetic activator would lead to expression of the ALO without the growth advantage provided by the endogenous substrate, D-allose. Thus, conceivably, addition of the synthetic activator to ready-to-eat foods could act as a listeriosis prophylactic.

## MATERIALS AND METHODS

### Bacterial growth conditions

All *L. monocytogenes* strains used in this study were on the background of the WT strain 10403S. Lm was grown in BHI broth or on BHI + 1.5% agar (RPI # B11000-5000.0). Overnight cultures of Lm were obtained by growth at 30°C statically for 18 h. When needed, cultures were supplemented with the following antibiotics: chloramphenicol (5–10 µg/mL), erythromycin (2 µg/mL), streptomycin (200 µg/mL), or tetracyline (2 µg/mL). Unless otherwise specified, catalog numbers refer to Thermo Scientific products. Donor *Escherichia coli* SM10 were grown in LB broth (Fisher Scientific BP97235) or LB + 1.5% agar supplemented when needed with carbenicillin (100 µg/mL) or chloramphenicol (34 µg/mL).

### Plasmid and strain construction

The strains used in this study are listed in Table S3 and primers in Table S4. Chromosomal deletions were made using allelic exchange from the pLIM backbone followed by counterselection on 18 mM DL-4-chlorohenylalanine (#157280250) (63). Complementation strains were made by integrating pPL2 (64) to the chromosome under pHyper (65) (*folD::folD*) or the native promoter (*alsR::alsR*) with selection on tetracyline. All plasmids were introduced to Lm via conjugation from *E. coli* SM10. A Lm transposon library was constructed as previously described (66) and stored at −80°C in 1 mL aliquots in BHI + 40% glycerol.

### Mice

Animals were housed at the University of Washington Department of Comparative Medicine vivarium under specific pathogen-free conditions. The heterozygous RECON^+/−^ founder as well as age-matched wild-type controls were bred with mice purchased from Jackson Labs (#000664).

### Generation of RECON-deficient mice

CRISPR/Cas9-engineered mice were generated as previously described (67, 68) with the University of Washington Transgenic Resources Program. Guide RNAs targeting exon 6 of *Akr1c13* were cloned into pX330-U6-Chimeric_BB-CBh-hSpCas9 (Addgene #42230). A T7-sgRNA PCR product was amplified and *in vitro* transcribed as previously described (68) using the MEGAshortscript T7 kit (#AM1354) and purified with the MEGAclear kit (#AM1908). *Cas9* mRNA used for injections was purchased from Sigma (#CAS9MRNA). Primer sequences are provided in the Table S4.

### RGEN-RFLP assay

Genotyping of founder mice using CRISPR/Cas-derived RNA-guided engineered nucleases (RGEN) restriction fragment length polymorphism (RFLP) analysis was done as previously described (67, 69). Guide RNAs were cloned and purified as detailed above. A 1.2 kb amplicon spanning the *Akr1c13* exon 6 genomic region was used as substrate DNA. Primer sequences are provided in Table S4. The RGEN-RFLP assay with Cas9 nuclease from *Streptococcus pyogenes* was carried out according to manufacturer’s instructions (NEB #M0386) with 30 nM sgRNA and 3 nM substrate DNA final concentrations.

### Primary macrophages

Primary BMDM from C57BL/6 and RECON^−/−^ mice were isolated (70) and differentiated (71) as previously described. BMDM were grown at 37°C in 5% CO_2_ in Dulbecco’s Modified Eagle Medium (DMEM) GlutaMAX (Gibco #10569-010) supplemented with 1 mM sodium pyruvate, 55 µM 2-mercaptoethanol, 10% heat-inactivated fetal bovine serum (FBS), and 10% L929 conditioned medium.

### RNA isolation and qRT-PCR analysis

For *Akr1c13* expression in tissues, organs were harvested, placed in 1 mL LBP from the NucleoSpin RNA Plus kit (Clontech #740984.250) with silica disruption beads, and snap frozen in liquid nitrogen. Samples were thawed on ice, homogenized with a multivortexer, and RNA was isolated with NucleoSpin RNA Plus kit per manufacturer’s instructions. cDNA was synthesized using Maxima H Minus First Strand cDNA synthesis kit (#EP0752). TaqMan Gene Expression Assay probes were used for quantification of gene expression (#Mm00657347_m1) with *Hprt* (#Mm03024075_m1) as an endogenous control. For ALO gene expression, overnight Lm were diluted to OD_600_ = 0.05 in BHI or BHI supplemented with 1% D-allose (MedChem Express #HY-128741) and grown at 37°C with shaking until OD_600_ = 0.4–0.6 and RNA was extracted as previously described (72). Briefly, equal volumes of culture and ice cold 100% methanol were mixed on ice and bacteria were pelleted, flash frozen and stored at −80°C. RNA was extracted using acidified phenol:chloroform:isoamyl alcohol with vortex agitation. RNA was precipitated, DNAse treated (#AM1907), and reverse transcribed using iScript cDNA synthesis (Bio-Rad #1708891). qPCR was performed using SYBR Green Master Mix (#A25742). Primers for qPCR are listed in Table S4.

### Mouse infections

All infections were carried out in 7- to 12-week-old RECON^−/−^ or C57BL/6 mice with equal sex distribution. For Tn-seq, a single vial of a Lm transposon library of approximately 17,000 mutants was thawed, diluted in 3 mL BHI, and grown 3 h at 37 °C, 240 rpm. The culture was then diluted in sterile phosphate buffered saline (PBS) to 1.2 × 10^7^ CFU/mL and 200 µL of this suspension was administered IV through the retroorbital injection to nine RECON^−/−^ mice to give 2.4 × 10^6^ CFU/mouse. Mice were sacrificed at 34 hpi and total liver and spleen homogenates were plated on BHI agar. Bacterial biomass was scraped and stored at −80°C. For all other infections, Lm were prepared as follows: overnight cultures of Lm were back-diluted in BHI (1.2 mL into 4.8 mL) and grown for 1 h at 37°C shaking then diluted in sterile PBS accordingly. For mouse survival studies, mice were IP injected with 1 × 10^6^ CFU/mouse in 200 µL. For experiments in Fig. 1C, 1 × 10^4^ CFU/mouse was injected IP in 200 µL and 72 hpi the livers and spleens were collected and plated for CFU. For experiments in Fig. 1E, 1 × 10^4^ CFU/mouse was injected via IV in 200 µL and bacterial burden was enumerated at 72 hpi. For experiments in Fig. 1F, 2 × 10^6^ CFU/mouse was injected via IV in 200 µL and CFU were collected from livers and spleens 4 and 24 hpi. All remaining infections in Fig. 3 and 4 and Fig. S3 and S4 were carried out in C56BL/6 mice with 1 × 10^5^ Lm CFU delivered IV in 200 µL. Unless otherwise noted, livers and spleens were collected at indicated time points and homogenized in 10 mL or 5 mL (respectively) 0.1% IGEPAL and plated on BHI agar with streptomycin.

### Plaque assay

Immortalized L2 rat fibroblasts and TIB73 mouse hepatocytes were cultures in DMEM GlutaMAX with 1 mM sodium pyruvate and 10% FBS. Plaque assays were performed on monolayers of L2 or TIB73 cells as previously described (73) with the following modifications. Cells were seeded in 3 mL media/well. Overnight Lm cultures were pelleted and resuspended in 1× PBS to OD_600_ = 1.0, then diluted 1:30 in PBS. To infect, 5 µL of the diluted bacteria were added directly to each well and swirled to mix (multiplicity of infection[MOI] 0.2). After 1 h, cells were washed twice in 1× PBS prior to the addition of an agar plug (DMEM + 10% FBS, 1 mM sodium pyruvate, 2 mM glutamine, 0.7% agarose, 10 µg/mL gentamicin). After 2 days, the staining mix (DMEM + 10% FBS, 1 mM sodium pyruvate, 2 mM glutamine, 0.7% agarose, 0.25% neutral red) was added for 18 h before plates were scanned and plaques analyzed in ImageJ. Superpure agarose was purchased from Biotech Sources (#G02PD-125) and neutral red from Sigma (#N6264).

### Tn-seq library construction and sequencing

Genomic DNA was extracted using MasterPure Gram-Positive DNA Purification Kit (Fisher Scientific #NC9197506) with 300 U/mL mutanolysin (Sigma #M9901-10KU) in place of lysosome. DNA was diluted to 3 µg/130 µL in microTUBEs (Covaris #520045) and sheared in triplicate to 300 bp fragments using the following settings on a Covaris LE220 Focused-Ultrasonicator: duty cycle 10%, peak intensity 450, cycles per burst 100, duration 200 s/column. Fragmented DNA was end-repaired (NEB #E6050) and purified on Ampure SPRIselect beads (Beckman-Coulter #B23317). Poly-C-tails were added to 1 µg of each end-repaired sample in duplicate using Terminal Transferase (Promega) with a ratio of 9.5 mM dCTP to 0.5 mM ddCTP to limit chain length, and duplicate reactions were combined and purified with SPRIselect beads. Transposon junctions were amplified with oligos pJZ_Fwd_RND1 and olj376 using 500 ng DNA and KAPA HiFi Hotstart Mix (Kapa Biosystems, #KK2602). Reactions were stopped at the inflection point of amplification (6–14 cycles). Transposon junction amplicons were purified using SPRIselect beads. Finally, barcoded adaptors were added to the samples using KAPA HiFi Hotstart Mix, pJZ_Fwd_RND2 and adaptors (listed in Table S4) to allow for pooled sequencing. The amount of each sample to add was empirically determined such that each sample reached inflection point after 17 rounds. DNA was purified and size selected on SPRIselect beads for fragments 250–450 bp in size. Samples were pooled to 9 nM and sequenced as single end 50 bp reads on NextSeq HO with a 10% PhiX spike in, resulting in 140 million reads with an average of 7.4 million reads per sample

### Conditional essentiality and pathway analysis

The *L. monocytogenes* 10403S NC_017544 genome was uploaded to PATRIC (24) as the reference genome. To determine organ specificity, reads from spleens and livers were separately analyzed. For each organ, reads from all mice were analyzed together as biological replicates. Trimmed reads were mapped and assessed for essentiality using the Tn-seq Analysis tool on PATRIC which performs TRANSIT with resampling (74). All genes that reached an adjusted *P*-value less than 0.05 with at least a twofold depletion were considered essential *in vivo*. These genes were further analyzed for KEGG Pathway enrichment using DAVID (75).

### Stats and quantification

All numerical data were analyzed and visualized in GraphPad Prism 9.0 software. For plaque assay and murine infection data, all data points are plotted with the bar at the median. The ALO qRT-PCR data are represented as mean with standard deviation. The number of repeats and statistical test used for each experiment are detailed in the figure legends.

## Data Availability

Tn-seq data generated in this study have been deposited to the Sequence Read Archive under BioProject PRJNA1062841.

## SUPPLEMENTAL MATERIAL

Supplemental file mbio.01332-24-s0001.pdf Supplemental figures and methods.

Supplemental tables mbio.01332-24-s0002.docx Tables S1 to S7.

ASM does not own the copyrights to Supplemental Material that may be linked to, or accessed through, an article. The authors have granted ASM a non-exclusive, world-wide license to publish the Supplemental Material files. Please contact the corresponding author directly for reuse.
