# Supplementary material for: RECON gene disruption enhances host resistance to enable genome-wide evaluation of intracellular pathogen fitness during infection
Source: mBio. 2024 Jun 28;15(8):e01332-24. doi: 10.1128/mbio.01332-24 (PMC11323731; doi:10.1128/mbio.01332-24)
Supplement: Supplemental file — Supplemental figures and methods. [file mbio.01332-24-s0001.pdf]

Supplemental Materials

Supplemental Figure 1

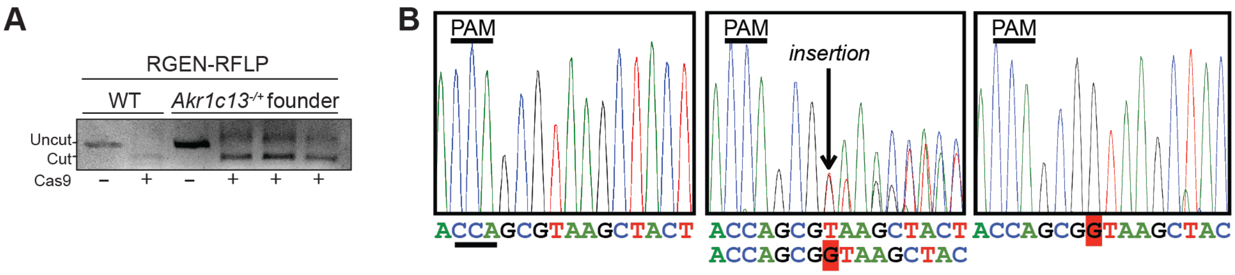

Supplemental Figure 1. Generation of RECON-deficient mice using CRISPR/Cas9-mediated mutagenesis. (**A**) RGEN-RFLP analysis of CRISPR-targeted region (exon 6) in the *Akr1c13* gene in the heterozygous founder. (**B**) Sequence of mutated allele with a single G insertion leading to a frameshift mutation.

Supplemental Figure 2

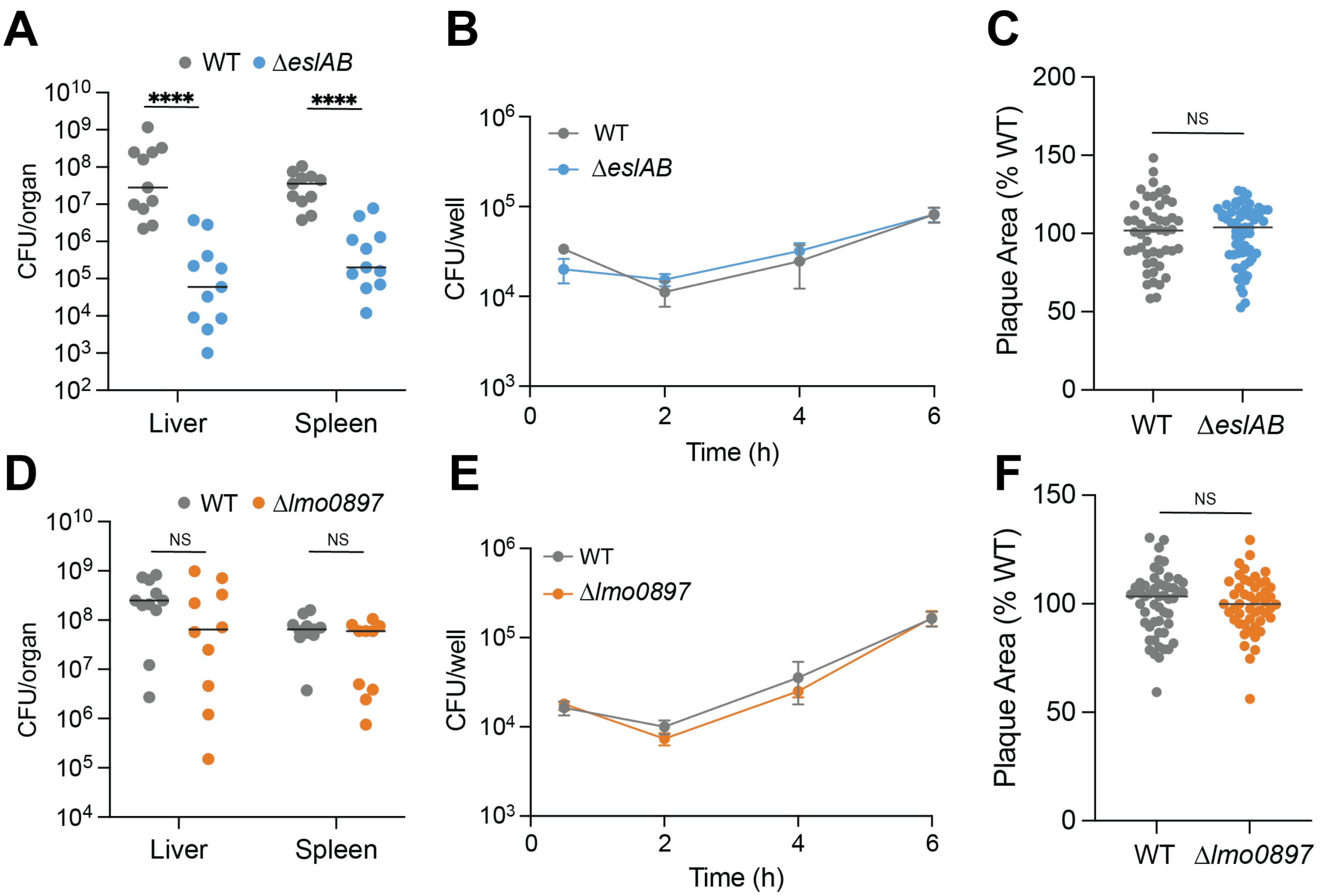

Supplemental Figure 2. Identification of novel genes required for in vivo growth only. (**A**) CFU harvested from livers and spleens of WT mice infected via IV with 1×10^5 CFU of WT (n=11) or Δ*eslAB* (n=11) Lm for 72 hours. Data are combined from two independent experiments. ****, P<0.0001 by Mann Whitney test. (**B**) Naïve BMDM were infected with WT or Δ*eslAB* Lm at an MOI 1. Intracellular CFU were collected at the indicated time points. (**C**) Plaque area measured in fibroblasts infected with WT or Δ*elsAB* Lm for 48 hours and stained with neutral red. (**D**) CFU harvested from livers and spleens of WT mice infected via IV with 1×10^5 CFU of WT (n=11) or Δ*lmo0897* (n=10) Lm for 72 hours. Data are combined from two independent experiments. (E) Naïve BMDM were infected with WT or Δ*lmo0897* Lm at an MOI 1. Intracellular CFU were collected at the indicated time points. (**F**) Plaque area measured in fibroblasts infected with WT or Δ*lmo0897* Lm for 48 hours and stained with neutral red.

Supplemental Figure 3

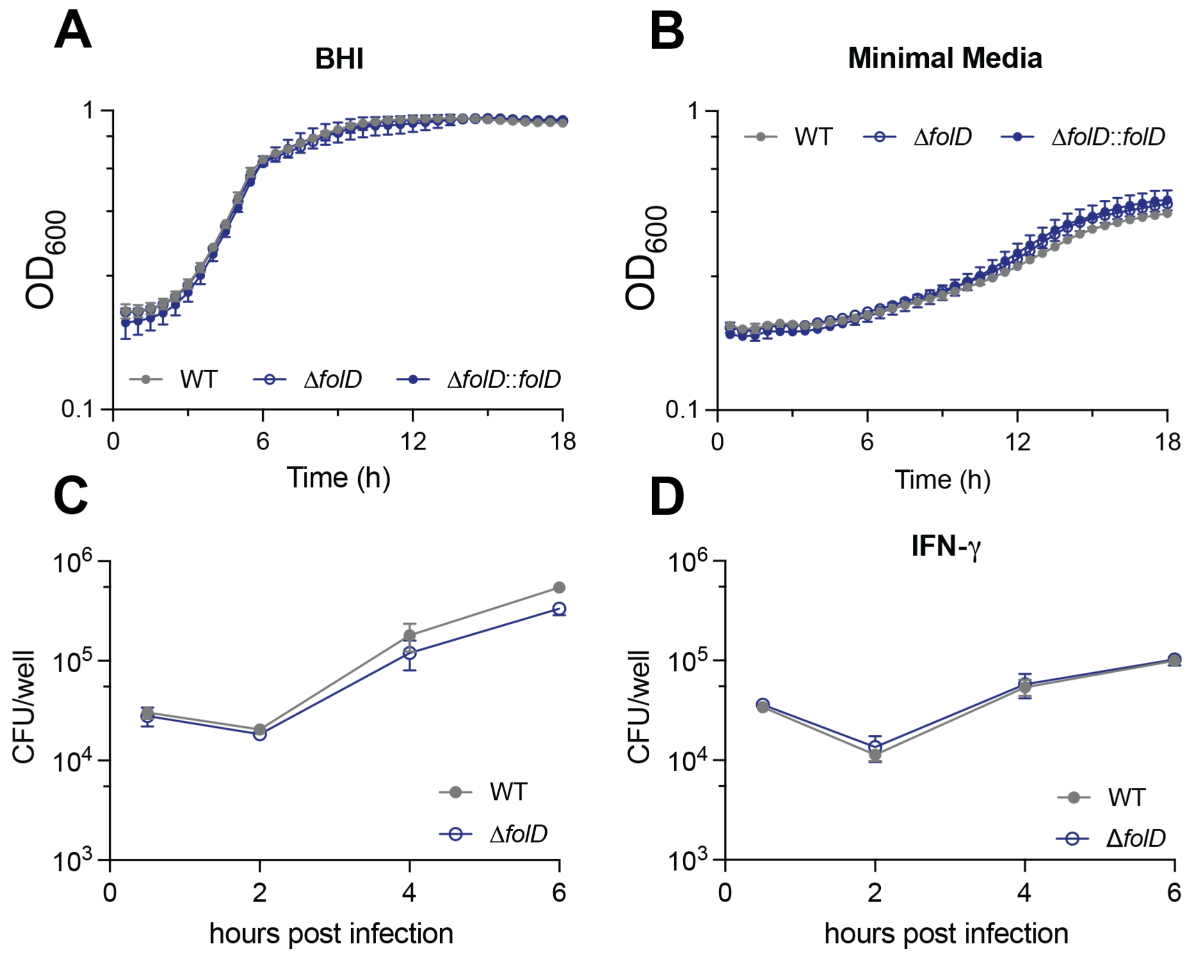

Supplemental Figure 3. The folate cycle gene *folD* is not required for Lm *in vitro* growth or growth in macrophages. (**A-B**) Bacterial growth as measured by OD600 over time in BHI (**A**) or minimal media (**B**). (**C-D**) BMDMs were left naïve (**C**) or stimulated with IFN-γ (**D**) for 18 hours prior to infection with Lm. Intracellular CFU were collected at the indicated time points.

Supplemental Figure 4

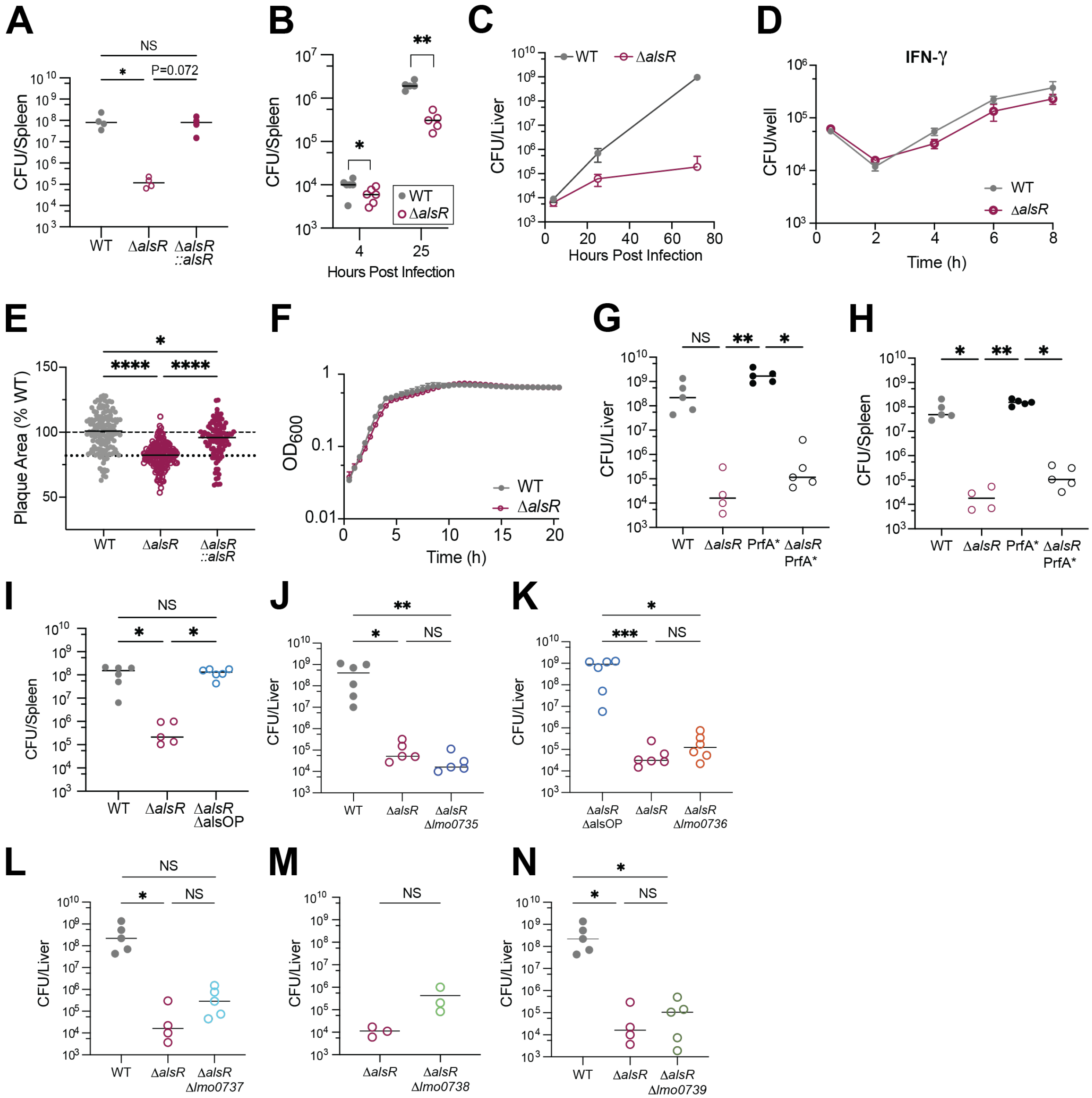

Supplemental Figure 4. Dysregulation of the D-allose utilization operon impairs Lm growth *in vivo* but not *in vitro*. (**A**) CFU harvested from spleens of WT mice infected via IV with 1x10^5 CFU of WT, Δ*alsR* or Δ*alsR::alsR* Lm (n=4/group) for 72 hours. (**B**) CFU harvested from spleens of WT mice infected via IV with 1x10^5 CFU of WT or Δ*alsR* for 4 (n=6/group) or 25 (n=5/group) hours. (**C**) Mean CFU of WT or Δ*alsR* from panels Figures 4B and 4C plotted over time. (**D**) IFN-γ-stimulated BMDMs were infected with Lm for the indicated time points and intracellular CFU were enumerated. (**E**) Plaque area measured in

fibroblasts infected with WT, Δ*alsR* or Δ*alsR*::*alsR* Lm for 48 hours and stained with neutral red. (**F**) Bacterial growth as measured by OD_{600} over time in BHI. (**G-H**) CFU harvested from livers (**G**) and spleens (**H**) of WT mice infected via IV with $1x10^5$ CFU of WT (n=5), Δ*alsR* (n=4), PrfA* (n=5) or Δ*alsR* PrfA* (n=5) Lm for 72 hours. (**I**). CFU harvested from spleens of WT mice infected via IV with $1x10^5$ CFU of WT (n=6), Δ*alsR* (n=5) or Δ*alsR*ΔalsOP (n=6) Lm for 72 hours. (**J-N**) CFU harvested from livers of WT mice infected via IV with $1x10^5$ CFU with the indicated strains for 72 hours. **J**, n=5/group. **K**, n=6/group. **L**, n=4-5/group. **M**, n=3/group. **N**, n=4-5/group. Experiments in **B**, **G-H**, and **I-N** were performed once. Plaque area in **E** and CFU in **A, G-N** were analyzed by Kruskal-Wallis test with * p<0.05, ** p< 0.005 and **** p< 0.0001. CFU in **B** was analyzed by Mann-Whitney analysis with *p<0.05, **p<0.01.

Supplemental Methods

SNP PCR genotyping of mice

Mouse genotyping was performed using a custom multiplex *Akr1c13* SNP-based genotyping assay. An end-point PCR was first performed to amplify *Akr1c13* exon 6. The product was diluted 1:1,000 in nuclease-free water, and 1.0 µL was used in a 20 µL qRT-PCR reaction, along with TaqMan Master Mix, primers that amplify *Akr1c13* exon 6 (500 nM final concentration, Table S4) and FAM probes that detect the WT allele mixed with HEX probes that detect the mutant allele (250 nM final probe concentrations, Table S4).

Bacterial growth curves

Overnight cultures of Lm were back diluted to OD_{600}=0.05 and grown at 37˚C with shaking until OD_{600} reached 0.4. Bacteria were resuspended in 1X PBS to OD_{600}=1.0, diluted 1:100 in BHI or MM and 200 µL of this suspension was distributed to a 96-well plate. The plate was covered in Breathe Easy film (Diversified Biotech #BEM-1) and incubated at 37˚C, 237 CPM in a BioTek Synergy HTX plate reader with OD_{600} reads every 30 minutes.

BMDM growth curves with Lm

BMDM were seeded in 24-well tissue culture treated plates at a density of 2.5 x 10^5 cells/well with the addition of 100ng/µL murine IFN-γ when indicated and incubated

overnight at 37˚C 5% CO_2. Cells were washed once in 1X PBS prior to infection. Overnight cultures of Lm were resuspended in 1X PBS to OD_{600}=1.0 and diluted 1:1,000 in DMEM +10% FBS +10% CSF and 500 µL of this infection medium was added to each well (MOI 3). Cells were incubated for 30 minutes to allow phagocytosis, then were washed twice in 1X PBS and replaced with fresh media containing 100µg/mL gentamicin for the remaining time points. At each time point the cells were washed twice in 1X PBS and lysed in 1 mL (0.5, 4, 6 hpi) or 500 µL (2 hpi) ice cold nanopure water. Lysates were diluted in PBS and plated for CFU enumeration on BHI + Streptomycin.
