# Supplementary material for: RECON gene disruption enhances host resistance to enable genome-wide evaluation of intracellular pathogen fitness during infection
Source: mBio. 2024 Jun 28;15(8):e01332-24. doi: 10.1128/mbio.01332-24 (PMC11323731; doi:10.1128/mbio.01332-24)
Supplement: Supplemental tables — Tables S1 to S7. [file mbio.01332-24-s0002.docx]

**Supplemental Tables**

Supplemental Table 1. Lm genes required for survival in the murine liver.

| **10403S locus^a,b^** | **EGD-e locus** | **Gene name** | **Description** | **log2FC** | **Adj. p-value** | | **Implicated in Virulence** |
| --- | --- | --- | --- | --- | --- | --- | --- |
| *LMRG_00822* | *lmo1372* | *bkdA1* | Branched-chain alpha-keto acid dehydrogenase | -8.41 | 0 | | (1) |
| ***LMRG_02624*** | ***lmo0202*** | ***hly*** | **Thiol-activated cytolysin** | -8.2 | 0 | | (2) |
| ***LMRG_01823*** | ***lmo2425*** | ***gcvH*** | **Glycine cleavage system H protein** | -8.13 | 0 | |  |
| ***LMRG_01613*** | ***lmo2219*** | ***prsA2*** | **Foldase protein** | -8.07 | 0 | | (3) |
| ***LMRG_02703*** | ***lmo2566*** | ***lipL*** | **Lipoyl-[GcvH]:protein N-lipoyltransferase** | -7.62 | 0 | | (4) |
| ***LMRG_02622*** | ***lmo0200*** | ***prfA*** | **Virulence regulatory factor PrfA** | -7.59 | 0 | | (5) |
| *LMRG_02841* | N/A |  | hypothetical protein | -7.35 | 0 | |  |
| *LMRG_01870* | *lmo2828* |  | hypothetical protein | -7.28 | 0 | |  |
| ***LMRG_02626*** | ***lmo0204*** | ***actA*** | **Actin-assembly inducing protein** | -7.2 | 0 | | (6) |
| ***LMRG_00514*** | ***lmo1052*** | ***pdhA*** | **Pyruvate dehydrogenase E1 alpha subunit** | -7.13 | 0 | |  |
| ***LMRG_02498*** | ***lmo1773*** | ***purB*** | **Adenylosuccinate lyase/ SAICAR lyase** | -7.07 | 0 | | (7) |
| ***LMRG_00281*** | ***lmo0598*** |  | **Biotin ECF transporter subunit (BioY)** | -7.01 | 0 | | (8) |
| *LMRG_00939* | *lmo1486* |  | Ribosomal silencing factor RsfA | -7.01 | 0 | |  |
| *LMRG_00204* | *lmo0523* |  | Uncharacterized MFS-type transporter | -6.94 | 0 | |  |
| ***LMRG_02078*** | ***lmo0978*** |  | **Branched-chain amino acid aminotransferase** | -6.87 | 0 | |  |
| ***LMRG_02485*** | ***lmo0055*** | ***purA*** | **Adenylosuccinate synthetase** | -6.82 | 0 | | (7) |
| *LMRG_00107* | *lmo0415* | *pgdA* | Peptidoglycan N-acetylglucosamine deacetylase | -6.8 | 0 | | (9) |
| ***LMRG_01946*** | ***lmo2750*** | ***pabBC*** | **Para-aminobenzoate synthase subunit** | -6.73 | 0 | | (10) |
| ***LMRG_01978*** | ***lmo2718*** | ***cydA*** | **Cytochrome d ubiquinol oxidase subunit I** | -6.64 | 0 | | (11) |
| ***LMRG_00265*** | ***lmo0583*** | ***secA2*** | **Protein translocase subunit SecA paralog 1** | -6.42 | 0 | | (12) |
| ***LMRG_01256*** | ***lmo2103*** | ***eutD*** | **Phosphate acetyltransferase** | -6.25 | 0 | |  |
| *LMRG_01717* | *lmo2531* | *atpA* | ATP synthase alpha chain | -6.21 | 0 | | (13) |
| *LMRG_02659* | *lmo0247* |  | hypothetical protein | -6.18 | 0 | |  |
| ***LMRG_00422*** | ***lmo0734*** | ***alsR*** | **Transcriptional regulator, LacI family** | -6.15 | 0 | |  |
| ***LMRG_00516*** | ***lmo1054*** | ***pdhC*** | **pyruvate dehydrogenase complex E2 subunit** | -6.08 | 0 | | (4) |
| *LMRG_01603* | *lmo2229* |  | penicillin-binding protein | -5.97 | 0 | | (14) |
| *LMRG_02467* | *lmo0038* |  | Agmatine deiminase | -5.96 | 0 | |  |
| *LMRG_01392* | *lmo1575* | *nrnA* | Linear Dinucleotide Phosphodiesterase | -5.88 | 0 | | (15) |
| *LMRG_00049* | *lmo0358* |  | PTS system, IIB component/ IIC component | -5.65 | 0 | |  |
| *LMRG_00878* | *lmo1426* | *opuCC* | carnitine ABC transporter substrate binding subunit | -5.58 | 0 | | (16) |
| ***LMRG_00327*** | ***lmo0641*** | ***frvA*** | **Iron (II) efflux ATPase** | -5.48 | 0 | | (17) |
| ***LMRG_01925*** | ***lmo2770*** | ***gshF*** | **Glutathione biosynthesis bifunctional protein gshF** | -5.39 | 0 | | (18) |
| *LMRG_00625* | *lmo1179* | *eutE* | Acetaldehyde dehydrogenase/ ethanolamine utilization cluster | -5.38 | 0 | |  |
| *LMRG_02449* | *lmo0020* |  | GntR Transcriptional regulator of PTSman-1 | -5.37 | 0 | |  |
| *LMRG_00823* | *lmo1373* | *bkdA2* | Branched-chain alpha-keto acid dehydrogenase | -5.37 | 0 | | (1) |
| *LMRG_00810* | *lmo1360* | *folD* | Methenyltetrahydrofolate cyclohydrolase/ Methylenetetrahydrofolate dehydrogenase | -5.31 | 0 | | (19) |
| *LMRG_01920* | *lmo2777* |  | Multidrug resistance transporter, Bcr/CflA family | -5.28 | 0 | |  |
| ***LMRG_01980*** | ***lmo2716*** | ***cydC*** | **ABC transporter/cysteine exporter** | -5.24 | 0 | |  |
| ***LMRG_01981*** | ***lmo2715*** | ***cydD*** | **ABC transporter/cysteine exporter** | -5.16 | 0 | | (11) |
| *LMRG_02580* | *lmo0291* | *walJ* | Zn-dependent hydrolase YycJ/WalJ | -5.06 | 0 | | (11) |
| ***LMRG_01792*** | ***lmo2456*** | ***pgm*** | **phosphoglycerate mutase** | -5.04 | 0 | |  |
| *LMRG_00622* | *lmo1176* | *eutC* | Ethanolamine ammonia-lyase light chain | -4.99 | 0 | |  |
| *LMRG_02507* | *lmo1764* | *purD* | Phosphoribosylamine--glycine ligase | -4.92 | 0 | |  |
| ***LMRG_01368*** | ***lmo1599*** | ***ccpA*** | **Catabolite control protein A** | -4.46 | 0 | |  |
| *LMRG_02673* | *lmo0233* | *radA* | DNA repair protein RadA | -4.38 | 0 | |  |
| *LMRG_01439* | *lmo1531* | *queA* | S-adenosylmethionine:tRNA ribosyltransferase-isomerase | -4.25 | 0 | |  |
| *LMRG_00718* | *lmo1268* | *clpX* | ATP-dependent Clp protease ATP-binding subunit ClpX | -4.19 | 0 | | (20) |
| *LMRG_02675* | *lmo0231* |  | Protein-arginine kinase McsB | -4.05 | 0 | |  |
| *LMRG_01938* | *lmo2758* | *guaB* | Inosine-5'-monophosphate dehydrogenase | -3.92 | 0 | |  |
| ***LMRG_01077*** | ***lmo1930*** |  | **Heptaprenyl diphosphate synthase component II** | -3.83 | 0 | |  |
| ***LMRG_01039*** | ***lmo1892*** | ***pbpA*** | **Penicillin-binding protein 1A/1B** | -3.79 | 0 | | (21) |
| ***LMRG_02030*** | ***lmo0931*** | ***lplA1*** | **Lipoate-protein ligase A** | -3.74 | 0 | | (22) |
| ***LMRG_00838*** | ***lmo1386*** | ***ftsK*** | **DNA translocase** | -3.7 | 0 | | (11) |
| *LMRG_01547* | *lmo2285* |  | Bacteriophage A118 gp18 | -3.43 | 0 | |  |
| ***LMRG_01195*** | ***lmo2046*** |  | **2-dehydropantoate 2-reductase** | -2.92 | 0 | |  |
| *LMRG_01078* | *lmo1931* | *menG* | Demethylmenaquinone methyltransferase | -7.58 | 0.0043 | |  |
| *LMRG_01467* | *lmo1503* | *reoM* | UPF0297 protein YrzL | -7.42 | 0.0043 | | (23) |
| *LMRG_00264* | *lmo0582* | *Iap* | Invasion associated secreted endopeptidase p60 | -7.01 | 0.0043 | | (12) |
| *LMRG_00751* | *lmo1301* |  | Acetyltransferase, GNAT family | -6.34 | 0.0043 | |  |
| *LMRG_00996* | *lmo1849* |  | Manganese ABC transporter, ATP-binding protein SitB | -5.48 | 0.0043 | |  |
| ***LMRG_01926*** | ***lmo2769*** | ***eslA*** | **Efflux ABC transporter, ATP-binding protein** | -5.4 | 0.0043 | |  |
| *LMRG_01854* | *lmo2844* |  | spermidine N1-acetyltransferase | -5.08 | 0.0043 | |  |
| *LMRG_00778* | *lmo1328* | *truB* | tRNA pseudouridine(55) synthase | -4.69 | 0.0043 | |  |
| *LMRG_01375* | *lmo1592* |  | tRNA 4-thiouridine synthase | -4.69 | 0.0043 | |  |
| *LMRG_01106* | *lmo1959* |  | Ferrichrome-binding periplasmic protein precursor | -4.49 | 0.0043 | |  |
| *LMRG_01237* | *lmo2086* |  | DNA-binding protein | -4.13 | 0.0043 | |  |
| *LMRG_00457* | *lmo0769* |  | putative alpha-1,6-mannanase | -4.01 | 0.0043 | |  |
| ***LMRG_00515*** | ***lmo1053*** | ***pdhB*** | **Pyruvate dehydrogenase E1 beta subunit** | -3.64 | 0.0043 | |  |
| *LMRG_00702* | *lmo1253* |  | Trehalose operon transcriptional repressor | -6.11 | 0.0082 | |  |
| *LMRG_01753* | *lmo2495* | *pstB* | Phosphate ABC transporter, ATP-binding protein PstB | -5.21 | 0.0082 | |  |
| *LMRG_00894* | *lmo1442* |  | Transmembrane transporter | -4.84 | 0.0082 | |  |
| *LMRG_02623* | *lmo0201* | *plcA* | Phosphatidylinositol-specific phospholipase C | -4.08 | 0.0082 | | (24) |
| *LMRG_02559* | *lmo1710* |  | Flavodoxin | -7.31 | 0.0119 | |  |
| *LMRG_01700* | *lmo2547* | *hom* | Homoserine dehydrogenase | -3.84 | 0.0119 | | (25) |
| *LMRG_01655* | *lmo2177* |  | Membrane protein | -6.3 | 0.0153 | |  |
| *LMRG_01897* | *lmo2800* |  | Oxidoreductase, Gfo/Idh/MocA family | -5.08 | 0.0153 | |  |
| ***LMRG_01396*** | ***lmo1571*** | ***pfkA*** | **6-phosphofructokinase** | -3.59 | 0.0153 | |  |
| ***LMRG_00429*** | ***lmo0741*** |  | **Transcriptional regulator, GntR family** | -4.11 | 0.0186 | |  |
| ***LMRG_02892*** | **N/A** |  | **hypothetical protein** | -1.51 | 0.0186 | |  |
| *LMRG_02524* | *lmo1747* | *virA* | ABC transporter-like sensor ATP-binding protein | -6.09 | 0.0213 | | (26) |
| ***LMRG_01223*** | ***lmo2072*** | ***rex*** | **Redox-sensing transcriptional repressor Rex** | -3.76 | 0.0213 | | (27) |
| *LMRG_02892* | N/A |  | hypothetical protein | -3.65 | 0.0213 | |  |
| *LMRG_01290* | *lmo1677* | *menA* | 1,4-dihydroxy-2-naphthoate polyprenyltransferase | -3.07 | 0.0213 | | (28) |
| *LMRG_02071* | *lmo0972* | *dltC* | D-alanine--poly(phosphoribitol) ligase ACP subunit | -7.48 | 0.0242 | | (29) |
| *LMRG_00604* | *lmo1161* |  | Propanediol utilization protein similar to EutJ | -4.76 | 0.0242 | |  |
| *LMRG_02864* | *lmo2711* |  | hypothetical protein | -5.18 | 0.0256 | |  |
| *LMRG_00730* | *lmo1280* | *codY* | GTP-sensing transcriptional pleiotropic repressor CodY | -4.74 | 0.0256 | | (30) |
| *LMRG_01905* | *lmo2792* |  | DNA-binding protein | -4.43 | 0.0256 | |  |
| *LMRG_02501* | *lmo1770* | *purL* | Phosphoribosylformylglycinamidine synthase | -4.2 | 0.0256 | | (25) |
| *LMRG_01387* | *lmo1580* |  | Universal stress protein family | -4.13 | 0.0256 | |  |
| *LMRG_00811* | *lmo1361* | *xseA* | Exodeoxyribonuclease VII large subunit | -3.81 | 0.0256 | |  |
| *LMRG_01973* | *lmo2723* |  | Acetyltransferase, GNAT family | -3.19 | 0.0256 | |  |
| *LMRG_01589* | *lmo2243* |  | AraC family transcriptional regulator | -5.38 | 0.0265 | |  |
| *LMRG_02500* | *lmo1771* | *purS* | Phosphoribosylformylglycinamidine synthase | -5.23 | 0.0265 | | (13) |
| *LMRG_01927* | *lmo2768* | *eslB* | Membrane protein | -5.16 | 0.0265 | |  |
| *LMRG_02046* | *lmo0947* |  | Uncharacterized MFS-type transporter YycB | -4.49 | 0.0265 | |  |
| *LMRG_01558* | N/A |  | Phage protein | -4.42 | 0.0265 | |  |
| *LMRG_01317* | *lmo1650* |  | CcdC protein | -4.39 | 0.0265 | |  |
| *LMRG_00270* | *lmo0588* |  | Deoxyribodipyrimidine photolyase | -4.36 | 0.0265 | | (13) |
| *LMRG_02103* | *lmo1003* | *ptsI* | phosphotransferase system E1 | -1.94 | 0.0265 | |  |
| *LMRG_00443* | *lmo0755* |  | Lipase/Acylhydrolase with GDSL-like motif in BtlB locus | -5.65 | 0.0286 | | (31) |
| *LMRG_01565* | *lmo2266* |  | Fumarylacetoacetate hydrolase family protein | -4.17 | 0.0286 | |  |
| *LMRG_01280* | *lmo2126* |  | Maltogenic amylase | -3.41 | 0.0286 | |  |
| *LMRG_02801* | *lmo2139* |  | Efflux ABC transporter, ATP-binding protein | -5.97 | 0.0308 | |  |
| *LMRG_02321* | *lmo0897* |  | Sulfate permease | -4.71 | 0.0308 | |  |
| *LMRG_02089* | *lmo0989* |  | Transcriptional regulator, MarR family | -5.63 | 0.0324 | |  |
| *LMRG_00665* | *lmo1219* |  | hypothetical protein | -5.12 | 0.0324 | | (13) |
| *LMRG_02053* | *lmo0954* |  | hypothetical protein | -4.6 | 0.0324 | |  |
| *LMRG_00800* | *lmo1350* | *gcvPB* | Glycine dehydrogenase P2 protein | -3.48 | 0.0324 | | (11) |
| *LMRG_01266* | *lmo2112* |  | DNA-binding protein | -5.57 | 0.0339 | |  |
| *LMRG_00416* | *lmo0728* | *ribF* | FMN adenylyltransferase / Riboflavin kinase | -4.98 | 0.0339 | | (32) |
| *LMRG_00824* | *lmo1374* | *bkdB* | Branched-chain alpha-keto acid dehydrogenase complex | -3.69 | 0.0339 | | (1) |
| *LMRG_01419* | *lmo1550* | *comC* | Late competence protein ComC, processing protease | -2.62 | 0.0339 | |  |
| *LMRG_02697* | *lmo2571* |  | Nicotinamidase | -7.03 | 0.0358 | |  |
| *LMRG_01754* | *lmo2494* | *phoU* | Phosphate transport system regulatory protein PhoU | -5.87 | 0.0358 | |  |
| *LMRG_01607* | *lmo2225* | *fumC* | Fumarate hydratase class II | -4.74 | 0.0374 | |  |
| *LMRG_01355* | *lmo1611* |  | Glutamyl aminopeptidase | -3.56 | 0.0374 | |  |
| *LMRG_02274* | *lmo0851* |  | hypothetical protein | -3.31 | 0.0374 | |  |
| *LMRG_00058* | *lmo0366* |  | Ferrous iron transport periplasmic protein EfeO | -4.93 | 0.0396 | |  |
| *LMRG_01095* | *lmo1948* | *resD* | DNA-binding response regulator | -6.06 | 0.0417 | |  |
| *LMRG_00701* | *lmos46* |  | hypothetical protein | -5.18 | 0.0487 | |  |
| *LMRG_00431* | *lmo0743* |  | ABC transporter, permease protein | -3.57 | 0.0507 | |  |
| *LMRG_01906* | *lmo2791* | *parA* | Chromosome (plasmid) partitioning protein ParA | -4.64 | 0.0527 | | (11) |
| *LMRG_00260* | *lmo0578* |  | hypothetical protein | -3.7 | 0.0546 | |  |
| **^a^**Genes underlined are also required in J774 macrophages (11). | | | | | |
| ^b^Genes in **bold** were also required in the spleen (Table S2). | | | | | |

Supplemental Table 2. Lm genes required for survival in the murine spleen.

| **10403S Locus^a,b^** | **EGD-e locus** | **Gene Name** | **Description** | **log2FC** | **Adj. p-value** | **Implicated in virulence** |
| --- | --- | --- | --- | --- | --- | --- |
| ***LMRG_01823*** | ***lmo2425*** | ***gcvH*** | **Glycine cleavage system H protein** | -8.43 | 0 |  |
| ***LMRG_01925*** | ***lmo2770*** | ***gshF*** | **Glutathione biosynthesis bifunctional protein gshF** | -8.36 | 0 | (18) |
| ***LMRG_02624*** | ***lmo0202*** | ***hly*** | **Thiol-activated cytolysin** | -8.13 | 0 | (2) |
| ***LMRG_01613*** | ***lmo2219*** | ***prsA2*** | **Foldase protein** | -7.94 | 0 | (3) |
| ***LMRG_02622*** | ***lmo0200*** | ***prfA*** | **Virulence regulatory factor PrfA** | -7.9 | 0 | (5) |
| *LMRG_01774* | *lmo2474* |  | RNase adapter protein RapZ | -7.66 | 0 |  |
| ***LMRG_02703*** | ***lmo2566*** | ***lipL*** | **Lipoyl-[GcvH]:protein N-lipoyltransferase** | -7.62 | 0 | (4) |
| ***LMRG_00514*** | ***lmo1052*** | ***pdhA*** | **Pyruvate dehydrogenase E1 component alpha subunit** | -7.39 | 0 |  |
| ***LMRG_02498*** | ***lmo1773*** | ***purB*** | **Adenylosuccinate lyase SAICAR lyase** | -6.88 | 0 | (7) |
| ***LMRG_00281*** | ***lmo0598*** |  | **Biotin ECF transporter subunit (BioY)** | -6.88 | 0 | (8) |
| ***LMRG_02718*** | ***lmo2718*** | ***cydA*** | **Cytochrome d ubiquinol oxidase subunit I** | -6.65 | 0 | (11) |
| ***LMRG_00516*** | ***lmo1054*** | ***pdhC*** | **pyruvate dehydrogenase complex E2 subunit** | -6.41 | 0 | (4) |
| ***LMRG_01926*** | ***lmo2769*** | ***eslA*** | **Efflux ABC transporter, ATP-binding protein** | -6.34 | 0 |  |
| ***LMRG_02485*** | ***lmo0055*** | ***purA*** | **Adenylosuccinate synthetase** | -6.03 | 0 | (7) |
| ***LMRG_00265*** | ***lmo0583*** | ***secA2*** | **Protein translocase subunit SecA paralog 1** | -6.02 | 0 | (12) |
| ***LMRG_01256*** | ***lmo2103*** | ***eutD*** | **Phosphate acetyltransferase** | -5.66 | 0 |  |
| ***LMRG_00422*** | ***lmo0734*** | ***alsR*** | **Transcriptional regulator, LacI family** | -5.51 | 0 |  |
| ***LMRG_00327*** | ***lmo0641*** | ***frvA*** | **Iron (II) efflux ATPase** | -5.46 | 0 |  |
| ***LMRG_01980*** | ***lmo2716*** | ***cydC*** | **ABC transporter/cysteine exporter** | -5.21 | 0 |  |
| ***LMRG_01981*** | ***lmo2715*** | ***cydD*** | **ABC transporter/cysteine exporter** | -5.08 | 0 | (11) |
| ***LMRG_02030*** | ***lmo0931*** | ***lplA1*** | **Lipoate-protein ligase A** | -4.94 | 0 | (22) |
| ***LMRG_01792*** | ***lmo2456*** | ***pgm*** | **phosphoglycerate mutase** | -4.72 | 0 |  |
| ***LMRG_01946*** | ***lmo2750*** | ***pabBC*** | **Para-aminobenzoate synthase subunit I** | -4.39 | 0 | (10) |
| *LMRG_02460* | *lmo0031* |  | Transcriptional regulator, LacI family | -4.21 | 0 |  |
| ***LMRG_01223*** | ***lmo2072*** | ***rex*** | **Redox-sensing transcriptional repressor Rex** | -4.17 | 0 | (27) |
| ***LMRG_01077*** | ***lmo1930*** |  | **Heptaprenyl diphosphate synthase component II** | -4 | 0 |  |
| ***LMRG_00515*** | ***lmo1053*** | ***pdhB*** | **Pyruvate dehydrogenase E1 beta subunit** | -3.71 | 0 |  |
| ***LMRG_00838*** | ***lmo1386*** | ***ftsK*** | **DNA translocase FtsK** | -3.7 | 0 | (11) |
| *LMRG_02627* | *lmo0205* | *plcB* | Broad-substrate range phospholipase C | -3.52 | 0 | (33) |
| ***LMRG_01195*** | ***lmo2046*** |  | **2-dehydropantoate 2-reductase** | -3.28 | 0 |  |
| ***LMRG_01368*** | ***lmo1599*** | ***ccpA*** | **Catabolite control protein A** | -3.22 | 0 |  |
| *LMRG_02636* | *lmo0214* | *mfd* | Transcription-repair coupling factor | -2.1 | 0 | (13) |
| ***LMRG_02626*** | ***lmo0204*** | ***actA*** | **Actin-assembly inducing protein** | -6.95 | 0.0084 | (6) |
| *LMRG_01386* | *lmo1581* | *ackA* | Acetate kinase | -3.84 | 0.0084 |  |
| ***LMRG_01039*** | ***lmo1892*** | ***pbpA*** | **Penicillin-binding protein 1A/1B** | -3.03 | 0.0084 | (21) |
| *LMRG_02063* | *lmo0964* | *yjbH* | Thioredoxin-fold protein | -5.11 | 0.0159 | (34) |
| *LMRG_01701* | *lmo2546* | *thrC* | Threonine synthase | -2.89 | 0.0159 |  |
| ***LMRG_02078*** | ***lmo0978*** |  | **Branched-chain amino acid aminotransferase** | -2.69 | 0.0232 |  |
| *LMRG_01923* | *lmo2772* |  | PTS system, beta-glucoside-specific | -1.91 | 0.0302 |  |
| *LMRG_00881* | *lmo1429* | *thiT* | Thiamine ECF transporter | -4.71 | 0.0359 | (35) |
| *LMRG_01204* | *lmo2054* |  | UPF0298 protein YlbG | -3.46 | 0.0359 |  |
| ***LMRG_00429*** | ***lmo0741*** |  | **Transcriptional regulator, GntR family** | -4.19 | 0.0468 |  |
| ***LMRG_01396*** | ***lmo1571*** | ***pfkA*** | **6-phosphofructokinase** | -3.57 | 0.0468 |  |
| *LMRG_02625* | *lmo0203* | *mpl* | Zinc metalloproteinase aureolysin | -2.97 | 0.0468 | (36) |
| ***LMRG_02892*** | **N/A** |  | **hypothetical protein** | -1.51 | 0.0523 |  |
| **^a^**Genes underlined are also required in J774 macrophages (11). | | | | |
| ^b^Genes in **bold** were also required in the liver (Table S1). | | | | |

Supplemental Table 3. Strains used in this study.

| **Strain Name** | **Background** | **Genotype** | **Plasmid** | **Reference** |
| --- | --- | --- | --- | --- |
| JW655 | *E. coli* XL1Blue |  | pLIM | (37) |
| CS315 | *E. coli* XL1Blue |  | pLIM-∆*folD* | This study |
| CS317 | *E. coli* XL1Blue |  | pLIM-∆*alsR* | This study |
| CS318 | *E. coli* XL1Blue |  | pPL2x-pHyper-*folD* | This study |
| CS322 | *E. coli* XL1Blue |  | pPL2t-n200-*alsR* | This study |
| CS102 | *L. monocytogenes* 10403S |  |  | (38) |
| CS108 | *L. monocytogenes* 10403S | ∆*eslAB* |  | This study |
| CS466 | *L. monocytogenes* 10403S | ∆*lmo0897* |  | This study |
| CS141 | *L. monocytogenes* 10403S | ∆*folD* |  | This study |
| CS148 | *L. monocytogenes* 10403S | ∆*folD*::*folD* |  | This study |
| CS147 | *L. monocytogenes* 10403S | ∆*alsR* |  | This study |
| CS152 | *L. monocytogenes* 10403S | ∆*alsR*::*alsR* |  | This study |
| JW130 | *L. monocytogenes* 10403S | PrfA* (G145S) |  | (39) |
| CS445 | *L. monocytogenes* 10403S | ∆*alsR* PrfA* |  | This study |
| CS456 | *L. monocytogenes* 10403S | ∆*alsR*∆alsOP |  | This study |
| CS175 | *L. monocytogenes* 10403S | ∆*alsR*∆*lmo0735* |  | This study |
| CS437 | *L. monocytogenes* 10403S | ∆*alsR*∆*lmo0736* |  | This study |
| CS449 | *L. monocytogenes* 10403S | ∆*alsR*∆*lmo0737* |  | This study |
| CS166 | *L. monocytogenes* 10403S | ∆*alsR*∆*lmo0738* |  | This study |
| CS441 | *L. monocytogenes* 10403S | ∆*alsR*∆*lmo0739* |  | This study |

Supplemental Table 4. Primers used in this study.

| **Oligo Name** | **Sequence (5' to 3')** | **Description** | **Ref** |
| --- | --- | --- | --- |
| AM_ex6g1_F | CACCgAGTAATCCAGTAGCTTACGC | Clone *Akr1c13* exon 6 guide into px330 |  |
| AM_ex6g1_R | AAACGCGTAAGCTACTGGATTACTc |  |
| T7-sgRNA_F | ttaatacgactcactataggAGTAATCCAGTAGCTTACGCg | *In vitro* transcription of *Akr1c13* sgRNA | (40) |
| T7-px330-sgRNA_R | AAAAGCACCGACTCGGTGCC |  |
| AM_FWD2_Akr1c13ex6-7 | TTGCAGCTGGCGTCTTTACC | *Akr1c13* substrate DNA for RGEN-RFLP |  |
| AM_REV2_Akr1c13ex6-7 | CAGACAGCACTTGACTGACAATGC |  |
| pJZ_Fwd_RND1 | GATAAATTTGAATACTAGTCTCGAGTGGGGTACG | Amplify transposon junction |  |
| olj376 | GTGACTGGAGTTCAGACGTGTGCTCTTCCGATCTGGGGGGGGGGGGGGGG |  |
| pJZ_Fwd_RND2 | AATGATACGGCGACCACCGAGACACCACTCTAGAGACCGGGGACTTATCAGCC |  |
| TdT_Index_1_ATCACG | CAAGCAGAAGACGGCATACGAGAT**CGTGAT**GTGACTGGAGTTCAGACGTGTGCTCTTCCGATCT | **Barcoded** Illumina adaptors |  |
| TdT_Index_2_CGATGT | CAAGCAGAAGACGGCATACGAGAT**ACATCG**GTGACTGGAGTTCAGACGTGTGCTCTTCCGATCT |  |
| TdT_Index_3_TTAGGC | CAAGCAGAAGACGGCATACGAGAT**GCCTAA**GTGACTGGAGTTCAGACGTGTGCTCTTCCGATCT |  |
| TdT_Index_4_TGACCA | CAAGCAGAAGACGGCATACGAGAT**TGGTCA**GTGACTGGAGTTCAGACGTGTGCTCTTCCGATCT |  |
| TdT_Index_5_TCTGAA | CAAGCAGAAGACGGCATACGAGAT**TTCAGA**GTGACTGGAGTTCAGACGTGTGCTCTTCCGATCT |  |
| TdT_Index_6_GAGTTC | CAAGCAGAAGACGGCATACGAGAT**GAACTC**GTGACTGGAGTTCAGACGTGTGCTCTTCCGATCT |  |
| TdT_Index_7_CGAGAT | CAAGCAGAAGACGGCATACGAGAT**ATCTCG**GTGACTGGAGTTCAGACGTGTGCTCTTCCGATCT |  |
| TdT_Index_8_TCTCGA | CAAGCAGAAGACGGCATACGAGAT**TCGAGA**GTGACTGGAGTTCAGACGTGTGCTCTTCCGATCT |  |
| TdT_Index_9_TAGCAG | CAAGCAGAAGACGGCATACGAGAT**CTGCTA**GTGACTGGAGTTCAGACGTGTGCTCTTCCGATCT |  |
| TdT_Index_10_AGCAGA | CAAGCAGAAGACGGCATACGAGAT**TCTGCT**GTGACTGGAGTTCAGACGTGTGCTCTTCCGATCT |  |
| TdT_Index_11_CAGCTT | CAAGCAGAAGACGGCATACGAGAT**AAGCTG**GTGACTGGAGTTCAGACGTGTGCTCTTCCGATCT |  |
| TdT_Index_12_ACAGTG | CAAGCAGAAGACGGCATACGAGAT**CACTGT**GTGACTGGAGTTCAGACGTGTGCTCTTCCGATCT |  |
| TdT_Index_13_GCCAAT | CAAGCAGAAGACGGCATACGAGAT**ATTGGC**GTGACTGGAGTTCAGACGTGTGCTCTTCCGATCT |  |
| TdT_Index_14_CAGATC | CAAGCAGAAGACGGCATACGAGAT**GATCTG**GTGACTGGAGTTCAGACGTGTGCTCTTCCGATCT |  |
| TdT_Index_15_ACTTGA | CAAGCAGAAGACGGCATACGAGAT**TCAAGT**GTGACTGGAGTTCAGACGTGTGCTCTTCCGATCT |  |
| TdT_Index_16_GATCAG | CAAGCAGAAGACGGCATACGAGAT**CTGATC**GTGACTGGAGTTCAGACGTGTGCTCTTCCGATCT |  |
| TdT_Index_17_GTCCGC | CAAGCAGAAGACGGCATACGAGAT**GCGGAC**GTGACTGGAGTTCAGACGTGTGCTCTTCCGATCT |  |
| TdT_Index_18_AGTCAA | CAAGCAGAAGACGGCATACGAGAT**TTGACT**GTGACTGGAGTTCAGACGTGTGCTCTTCCGATCT |  |
| pJZTnSq_Seq | CACCACTCTAGAGACCGGGGACTTATCAGCCAAC | Illumina sequencing primer | |
| CES055_F | cttggtaccGAACAAAAGGAGCAGCTTTAAC | Clone *folD* flanks into pLIM |  |
| CES056_R | CATTTCACATCCAATTAATCATCCCATGAGTATTGCCCCCT |  |
| CES057_F | AGGGGGCAATACTCATGGGATGATTAATTGGATGTGAAATG |  |
| CES058_R | gaaggatccCGATGAATGGTGGTTAGAATA |  |
| CES067_F | cttggtaccCAGCTTCTGGATGAATGTAAATAATATC | Clone *alsR* flanks into pLIM |  |
| CES060_R | GGACAGCCTATTTTTATTTACATAGTATCCTCCTTCTATG |  |
| CES061_F | CATAGAAGGAGGATACTATGTAAATAAAAATAGGCTGTCC |  |
| CES068_R | gaatctagaGTAAGCACTTTGCTTGGAGAAACTG |  |
| CES069_F | cttgtcgacATGGGAGAAATTATTGATGGC | *folD* complementation in pPL2 |  |
| CES070_R | gaagcggccgcTCAATTCATTTTCCAAATGCG |  |
| CES075_F | cttgtcgacCTTTGAGATAACGATTACTC | *alsR* complementation in pPL2 |  |
| CES074_R | gaagcggccgcTTAAATTGAGTCTTTTAAAGATAATTC |  |
| CES223_F | cttggtaccCCTTACTTAGGTTGGG | ∆*alsR*∆alsOP construction in pLIM |  |
| CES224_R | GATATATGAGATTTATATTAATTTATAAATAAAAATAGGCTGTCCA |  |
| CES225_F | TGGACAGCCTATTTTTATTTATAAATTAATATAAATCTCATATATC |  |
| CES146_R | ggaggatccCATGAGTGAAAGTCTTATGCTT |  |
| CES139_F | cttggtaccCCTCCTTCTATGAGTAAACG | ∆*lmo0735* |  |
| CES134_R | CATTTCCAATAGCAATTTTCACATCAATTTTCCACTCCTTTG | construction |  |
| CES135_F | CAAAGGAGTGGAAAATTGATGTGAAAATTGCTATTGGAAATG | in pLIM |  |
| CES136_R | gaaggatccGCATGTCTTCCACCTTCAAATTC |  |  |
| CES195_F | cttggtaccCCAGAAGCTGATTTACATCCAG | ∆*lmo0736* |  |
| CES196_R | CTCCATAACAACCCCTCTTACATCGTAATTCCTCCATCTTT | construction |  |
| CES197_F | AAAGATGGAGGAATTACGATGTAAGAGGGGTTGTTATGGAG | in pLIM |  |
| CES198_R | gaaggatccCTTAATAAATGTTCATCGGTAG |  |  |
| CES202_F | cttggtaccCGTATTTACAGGATTTAGGTC | ∆*lmo0737* |  |
| CES203_R | CACTCCTTTTTATAGTTTTACATAACAACCCCTCTTAATC | construction |  |
| CES204_F | GATTAAGAGGGGTTGTTATGTAAAACTATAAAAAGGAGTG | in pLIM |  |
| CES205_R | gaaggatccCAATCCTTTGAGCATACCACC |  |  |
| CES129_F | cttggtaccGCGATCCACTTGCTAAACAAG | ∆*lmo0738* |  |
| CES130_R | ATCCTCCTTCGATATTACATTTTTTTCACTCCTTTTTATAG | construction |  |
| CES131_F | TAAAAAGGAGTGAAAAAAATGTAATATCGAAGGAGGATAAAAAATG | in pLIM |  |
| CES132_R | gaaggatccCATAAGCATATAAAGTAACAAC |  |  |
| CES199_F | cttggtaccGGGTTACTTGGAAAAGGTGT | ∆*lmo0739* |  |
| CES200_R | ATGAGATTTATATTAATTTACATTTTTTATCCTCCTTCG | construction |  |
| CES201_F | CGAAGGAGGATAAAAAATGTAAATTAATATAAATCTCAT | in pLIM |  |
| CES146_R | ggaggatccCATGAGTGAAAGTCTTATGCTT |  |  |
| CES001_F | cttggtaccTCAGCAAGGTAAACCGGACTTGCG | ∆*eslAB* |  |
| CES002_R | CTATTTTTttaATTCGTTTTAGACCGAATTTTcatTATTCCACCTCTTTC | construction |  |
| CES003_F | GAATAatgAAAATTCGGTCTAAAACGAATtaaAAAAATAGCTTTTTT | in pLIM |  |
| CES004_R | caatctagaCAGGCGACCGCTTCGCATTATG |  |  |
| MKT0363_F | AGAAATATCGGTTGCAATCG | ∆*lmo0897* |  |
| MKT0364_R | ACCTTTTACACTATTCAACAAAAC | construction |  |
| MKT0365_F | GTTTTGTTGAATAGTGTAAAAGGTCATTATGCGGAAAATGCTTTAAATAAGtga | in pLIM |  |
| MKT0366_R | CGATTGTTGCTTTCGTGCGT |  |  |
| MKT0367_F | gccacggaattcAGAAATATCGGTTGCAATCG |  |  |
| MKT0368_R | gaccgtgtcgacCGATTGTTGCTTTCGTGCGT |  |  |
| CES080_F | GGTGGATGGGTTGATTGTTATTTC | qPCR *alsR* |  |
| CES081_R | TGGTTGGCGGTCAATACATAC |  |
| CES082_F | AATCGCAGAACTTGTCGAATTG | qPCR *lmo0735* |  |
| CES083_R | CCCTTAACACCTAACTGAGAAAGT |  |
| CES084_F | GTGGTTCAGGAGTGGGTATTT | qPCR *lmo0736* |  |
| CES085_R | TATTGTGCTCGCGTGATAGTT |  |
| CES086_F | CCAGGATCAAGACACGGTATAAG | qPCR *lmo0737* |  |
| CES087_R | GCGAGTGTACCTGGTTTGTAT |  |
| CES088_F | CAGGTGGTGGTATGCTCAAA | qPCR *lmo0738* |  |
| CES089_R | CTATCAGCAGCCGCGTATAAA |  |
| CES090_F | ACGATGCTGGAGTTGAGTTT | qPCR *lmo0739* |  |
| CES091_R | CAAGCAGTTGTAACGGCATATC |  |
| CES102_F | GAAGCAGTTGGTGTTGGTTTC | qPCR *GroEL* |  |
| CES103_R | GTAGAGCGGAACGTGTTACTT |  |
| Akr1c13-F | TCTATGGTAGGTTGAATGTCATCTC | Genotyping RECON^-/-^ mice via qPCR |  |
| Akr1c13-R | AGCAACGAGAACAATGTCTTTTG |  |
| Akr1c13-probe-FAM-WT | FAM-CAGCGTAAGCTACTGGATTACT |  |
| Akr1c13-probe-HEX-KO | HEX-CAGCGGTAAGCTACTGGATTACT |  |

Supplemental Figure 5. All mapped reads in liver samples.

| **EGDe locus** | **10403S locus** | **Description** | **log2FC** | **Adj. p-value** |
| --- | --- | --- | --- | --- |
| lmo1372 | LMRG_00822 | Branched-chain alpha-keto acid dehydrogenase, E1 component, alpha subunit (EC 1.2.4.4) | -8.41 | 0 |
| lmo0202 | LMRG_02624 | Thiol-activated cytolysin | -8.2 | 0 |
| lmo2425 | LMRG_01823 | Glycine cleavage system H protein | -8.13 | 0 |
| lmo2219 | LMRG_01613 | Foldase protein PrsA precursor (EC 5.2.1.8) @ Foldase clustered with pyrimidine conversion | -8.07 | 0 |
| lmo2566 | LMRG_02703 | Lipoyl-[GcvH]:protein N-lipoyltransferase (EC 2.3.1.200) | -7.62 | 0 |
| lmo0200 | LMRG_02622 | Virulence regulatory factor PrfA / Transcriptional regulator, Crp/Fnr family | -7.59 | 0 |
| N/A | LMRG_02841 | FIG00775234: hypothetical protein | -7.35 | 0 |
| lmo2828 | LMRG_01870 | hypothetical protein | -7.28 | 0 |
| lmo0204 | LMRG_02626 | Actin-assembly inducing protein ActA precursor | -7.2 | 0 |
| lmo1052 | LMRG_00514 | Pyruvate dehydrogenase E1 component alpha subunit (EC 1.2.4.1) | -7.13 | 0 |
| lmo1773 | LMRG_02498 | Adenylosuccinate lyase (EC 4.3.2.2) @ SAICAR lyase (EC 4.3.2.2) | -7.07 | 0 |
| lmo0597a | LMRG_00281 | Substrate-specific component BioY of biotin ECF transporter | -7.01 | 0 |
| lmo1486 | LMRG_00939 | Ribosomal silencing factor RsfA | -7.01 | 0 |
| lmo0523 | LMRG_00204 | Uncharacterized MFS-type transporter | -6.94 | 0 |
| lmo0978 | LMRG_02078 | Branched-chain amino acid aminotransferase (EC 2.6.1.42) | -6.87 | 0 |
| lmo0055 | LMRG_02485 | Adenylosuccinate synthetase (EC 6.3.4.4) | -6.82 | 0 |
| lmo0415 | LMRG_00107 | Peptidoglycan N-acetylglucosamine deacetylase (EC 3.5.1.-) | -6.8 | 0 |
| lmo2750 | LMRG_01946 | Para-aminobenzoate synthase, aminase component (EC 2.6.1.85) / Aminodeoxychorismate lyase (EC 4.1.3.38) | -6.73 | 0 |
| lmo2718 | LMRG_01978 | Cytochrome d ubiquinol oxidase subunit I (EC 1.10.3.-) | -6.64 | 0 |
| lmo0583 | LMRG_00265 | Protein translocase subunit SecA paralog 1 | -6.42 | 0 |
| lmo2103 | LMRG_01256 | Phosphate acetyltransferase (EC 2.3.1.8) | -6.25 | 0 |
| lmo2531 | LMRG_01717 | ATP synthase alpha chain (EC 3.6.3.14) | -6.21 | 0 |
| lmo0247 | LMRG_02659 | hypothetical protein | -6.18 | 0 |
| lmo0734 | LMRG_00422 | Transcriptional regulator, LacI family | -6.15 | 0 |
| lmo1054 | LMRG_00516 | Dihydrolipoamide acetyltransferase component of pyruvate dehydrogenase complex (EC 2.3.1.12) | -6.08 | 0 |
| lmo2229 | LMRG_01603 | Multimodular transpeptidase-transglycosylase (EC 2.4.1.129) (EC 3.4.-.-) | -5.97 | 0 |
| lmo0038 | LMRG_02467 | Agmatine deiminase (EC 3.5.3.12) | -5.96 | 0 |
| lmo1575 | LMRG_01392 | 3'-to-5' oligoribonuclease A, Bacillus type | -5.88 | 0 |
| lmo0358 | LMRG_00049 | PTS system, IIB component / PTS system, IIC component | -5.65 | 0 |
| lmo1426 | LMRG_00878 | Choline ABC transport system, choline-binding protein OpuBC | -5.58 | 0 |
| lmo0641 | LMRG_00327 | Lead, cadmium, zinc and mercury transporting ATPase (EC 3.6.3.3) (EC 3.6.3.5) | -5.48 | 0 |
| lmo2770 | LMRG_01925 | Glutathione biosynthesis bifunctional protein gshF (EC 6.3.2.2)(EC 6.3.2.3) | -5.39 | 0 |
| lmo1179 | LMRG_00625 | Acetaldehyde dehydrogenase (EC 1.2.1.10) @ Acetaldehyde dehydrogenase (EC 1.2.1.10), ethanolamine utilization cluster | -5.38 | 0 |
| lmo0020 | LMRG_02449 | Transcriptional regulator, GntR family | -5.37 | 0 |
| lmo1373 | LMRG_00823 | Branched-chain alpha-keto acid dehydrogenase, E1 component, beta subunit (EC 1.2.4.4) | -5.37 | 0 |
| lmo1360 | LMRG_00810 | Methenyltetrahydrofolate cyclohydrolase (EC 3.5.4.9) / Methylenetetrahydrofolate dehydrogenase (NADP+) (EC 1.5.1.5) | -5.31 | 0 |
| lmo2777 | LMRG_01920 | Multidrug resistance transporter, Bcr/CflA family | -5.28 | 0 |
| lmo2716 | LMRG_01980 | Efflux ABC transporter for glutathione/L-cysteine, essential for assembly of bd-type respiratory oxidases => CydC subunit | -5.24 | 0 |
| lmo2715 | LMRG_01981 | Efflux ABC transporter for glutathione/L-cysteine, essential for assembly of bd-type respiratory oxidases => CydD subunit | -5.16 | 0 |
| lmo0291 | LMRG_02580 | Zn-dependent hydrolase YycJ/WalJ, required for cell wall metabolism and coordination of cell division with DNA replication | -5.06 | 0 |
| lmo2456 | LMRG_01792 | 2,3-bisphosphoglycerate-independent phosphoglycerate mutase (EC 5.4.2.12) | -5.04 | 0 |
| lmo1176 | LMRG_00622 | Ethanolamine ammonia-lyase light chain (EC 4.3.1.7) | -4.99 | 0 |
| lmo1764 | LMRG_02507 | Phosphoribosylamine--glycine ligase (EC 6.3.4.13) | -4.92 | 0 |
| lmo1599 | LMRG_01368 | Catabolite control protein A | -4.46 | 0 |
| lmo0233 | LMRG_02673 | DNA repair protein RadA | -4.38 | 0 |
| lmo1531 | LMRG_01439 | S-adenosylmethionine:tRNA ribosyltransferase-isomerase (EC 2.4.99.17) | -4.25 | 0 |
| lmo1268 | LMRG_00718 | ATP-dependent Clp protease ATP-binding subunit ClpX | -4.19 | 0 |
| lmo0231 | LMRG_02675 | Protein-arginine kinase McsB (EC 2.7.14.1) | -4.05 | 0 |
| lmo2758 | LMRG_01938 | Inosine-5'-monophosphate dehydrogenase (EC 1.1.1.205) / CBS domain | -3.92 | 0 |
| lmo1930 | LMRG_01077 | Heptaprenyl diphosphate synthase component II (EC 2.5.1.30) | -3.83 | 0 |
| lmo1892 | LMRG_01039 | Penicillin-binding protein 1A/1B @ Multimodular transpeptidase-transglycosylase | -3.79 | 0 |
| lmo0931 | LMRG_02030 | Lipoate-protein ligase A | -3.74 | 0 |
| lmo1386 | LMRG_00838 | DNA translocase FtsK | -3.7 | 0 |
| lmo2285 | LMRG_01547 | Putative tail or base plate protein gp18 [Bacteriophage A118] | -3.43 | 0 |
| lmo2046 | LMRG_01195 | 2-dehydropantoate 2-reductase (EC 1.1.1.169) | -2.92 | 0 |
| lmo1931 | LMRG_01078 | Demethylmenaquinone methyltransferase (EC 2.1.1.163) | -7.58 | 0.00433 |
| lmo1503 | LMRG_01467 | UPF0297 protein YrzL | -7.42 | 0.00433 |
| lmo0582 | LMRG_00264 | P60 extracellular protein, invasion associated protein Iap | -7.01 | 0.00433 |
| lmo1301 | LMRG_00751 | Acetyltransferase, GNAT family | -6.34 | 0.00433 |
| lmo1849 | LMRG_00996 | Manganese ABC transporter, ATP-binding protein SitB | -5.48 | 0.00433 |
| lmo2769 | LMRG_01926 | Efflux ABC transporter, ATP-binding protein | -5.4 | 0.00433 |
| lmo2844 | LMRG_01854 | spermidine N1-acetyltransferase | -5.08 | 0.00433 |
| lmo1328 | LMRG_00778 | tRNA pseudouridine(55) synthase (EC 5.4.99.25) | -4.69 | 0.00433 |
| lmo1592 | LMRG_01375 | tRNA 4-thiouridine synthase (EC 2.8.1.4) | -4.69 | 0.00433 |
| lmo1959 | LMRG_01106 | Ferrichrome-binding periplasmic protein precursor (TC 3.A.1.14.3) | -4.49 | 0.00433 |
| lmo2086 | LMRG_01237 | DNA-binding protein | -4.13 | 0.00433 |
| lmo0769 | LMRG_00457 | putative alpha-1,6-mannanase | -4.01 | 0.00433 |
| lmo1053 | LMRG_00515 | Pyruvate dehydrogenase E1 component beta subunit (EC 1.2.4.1) | -3.64 | 0.00433 |
| lmo1253 | LMRG_00702 | Trehalose operon transcriptional repressor | -6.11 | 0.00818 |
| lmo2495 | LMRG_01753 | Phosphate ABC transporter, ATP-binding protein PstB (TC 3.A.1.7.1) | -5.21 | 0.00818 |
| lmo1442 | LMRG_00894 | hypothetical protein | -4.84 | 0.00818 |
| lmo0201 | LMRG_02623 | Phosphatidylinositol-specific phospholipase C (EC 4.6.1.13) | -4.08 | 0.00818 |
| lmo1710 | LMRG_02559 | Flavodoxin | -7.31 | 0.01194 |
| lmo2547 | LMRG_01700 | Homoserine dehydrogenase (EC 1.1.1.3) | -3.84 | 0.01194 |
| lmo2177 | LMRG_01655 | Membrane protein | -6.3 | 0.01529 |
| lmo2800 | LMRG_01897 | Oxidoreductase, Gfo/Idh/MocA family | -5.08 | 0.01529 |
| lmo1571 | LMRG_01396 | 6-phosphofructokinase (EC 2.7.1.11) | -3.59 | 0.01529 |
| lmo0741 | LMRG_00429 | Transcriptional regulator, GntR family | -4.11 | 0.01863 |
| #N/A | LMRG_02892 | hypothetical protein | -1.51 | 0.01863 |
| lmo1747 | LMRG_02524 | ABC transporter-like sensor ATP-binding protein | -6.09 | 0.02128 |
| lmo2072 | LMRG_01223 | Redox-sensing transcriptional repressor Rex | -3.76 | 0.02128 |
| lmo0081 | LMRG_02891 | hypothetical protein | -3.65 | 0.02128 |
| lmo1677 | LMRG_01290 | 1,4-dihydroxy-2-naphthoate polyprenyltransferase (EC 2.5.1.74) | -3.07 | 0.02128 |
| lmo0972 | LMRG_02071 | D-alanine--poly(phosphoribitol) ligase ACP subunit (EC 6.1.1.13) | -7.48 | 0.02424 |
| lmo1161 | LMRG_00604 | Propanediol utilization protein similar to EutJ | -4.76 | 0.02424 |
| lmo2711 | LMRG_02864 | hypothetical protein | -5.18 | 0.0256 |
| lmo1280 | LMRG_00730 | GTP-sensing transcriptional pleiotropic repressor CodY | -4.74 | 0.0256 |
| lmo2792 | LMRG_01905 | DNA-binding protein | -4.43 | 0.0256 |
| lmo1770 | LMRG_02501 | Phosphoribosylformylglycinamidine synthase, glutamine amidotransferase subunit (EC 6.3.5.3) | -4.2 | 0.0256 |
| lmo1580 | LMRG_01387 | Universal stress protein family | -4.13 | 0.0256 |
| lmo1361 | LMRG_00811 | Exodeoxyribonuclease VII large subunit (EC 3.1.11.6) | -3.81 | 0.0256 |
| lmo2723 | LMRG_01973 | Acetyltransferase, GNAT family | -3.19 | 0.0256 |
| lmo2243 | LMRG_01589 | Methylphosphotriester-DNA--protein-cysteine S-methyltransferase (EC 2.1.1.n11) / ADA regulatory protein | -5.38 | 0.0265 |
| lmo1771 | LMRG_02500 | Phosphoribosylformylglycinamidine synthase, PurS subunit (EC 6.3.5.3) | -5.23 | 0.0265 |
| lmo2768 | LMRG_01927 | Membrane protein | -5.16 | 0.0265 |
| lmo0947 | LMRG_02046 | Uncharacterized MFS-type transporter YycB | -4.49 | 0.0265 |
| #N/A | LMRG_01558 | Phage protein | -4.42 | 0.0265 |
| lmo1650 | LMRG_01317 | CcdC protein | -4.39 | 0.0265 |
| lmo0588 | LMRG_00270 | Deoxyribodipyrimidine photolyase (EC 4.1.99.3) | -4.36 | 0.0265 |
| lmo1003 | LMRG_02103 | Phosphoenolpyruvate-protein phosphotransferase of PTS system (EC 2.7.3.9) | -1.94 | 0.0265 |
| lmo0755 | LMRG_00443 | Lipase/Acylhydrolase with GDSL-like motif in BtlB locus | -5.65 | 0.02858 |
| lmo2266 | LMRG_01565 | Fumarylacetoacetate hydrolase family protein | -4.17 | 0.02858 |
| lmo2126 | LMRG_01280 | Neopullulanase (EC 3.2.1.135) | -3.41 | 0.02858 |
| lmo2139 | LMRG_02801 | Efflux ABC transporter, ATP-binding protein | -5.97 | 0.03084 |
| lmo0897 | LMRG_02321 | Sulfate permease | -4.71 | 0.03084 |
| lmo0989 | LMRG_02089 | Transcriptional regulator, MarR family | -5.63 | 0.03241 |
| lmo1219 | LMRG_00665 | hypothetical protein | -5.12 | 0.03241 |
| lmo0954 | LMRG_02053 | hypothetical protein | -4.6 | 0.03241 |
| lmo1350 | LMRG_00800 | Glycine dehydrogenase [decarboxylating] (glycine cleavage system P2 protein) (EC 1.4.4.2) | -3.48 | 0.03241 |
| lmo2112 | LMRG_01266 | DNA-binding protein | -5.57 | 0.03387 |
| lmo0728 | LMRG_00416 | FMN adenylyltransferase (EC 2.7.7.2) / Riboflavin kinase (EC 2.7.1.26) | -4.98 | 0.03387 |
| lmo1374 | LMRG_00824 | Dihydrolipoamide acyltransferase component of branched-chain alpha-keto acid dehydrogenase complex (EC 2.3.1.168) | -3.69 | 0.03387 |
| lmo1550 | LMRG_01419 | Late competence protein ComC, processing protease | -2.62 | 0.03387 |
| lmo2571 | LMRG_02697 | Nicotinamidase (EC 3.5.1.19) | -7.03 | 0.03584 |
| lmo2494 | LMRG_01754 | Phosphate transport system regulatory protein PhoU | -5.87 | 0.03584 |
| lmo2225 | LMRG_01607 | Fumarate hydratase class II (EC 4.2.1.2) | -4.74 | 0.03742 |
| lmo1611 | LMRG_01355 | Glutamyl aminopeptidase (EC 3.4.11.7) | -3.56 | 0.03742 |
| lmo0851 | LMRG_02274 | hypothetical protein | -3.31 | 0.03742 |
| lmo0366 | LMRG_00058 | Ferrous iron transport periplasmic protein EfeO, contains peptidase-M75 domain and (frequently) cupredoxin-like domain | -4.93 | 0.03958 |
| lmo1948 | LMRG_01095 | DNA-binding response regulator ResD | -6.06 | 0.04171 |
| lmos46 | LMRG_00701 | hypothetical protein | -5.18 | 0.04866 |
| lmo0743 | LMRG_00431 | ABC transporter, permease protein | -3.57 | 0.05068 |
| lmo2791 | LMRG_01906 | Chromosome (plasmid) partitioning protein ParA | -4.64 | 0.05266 |
| lmo0578 | LMRG_00260 | hypothetical protein | -3.7 | 0.05461 |
| lmo0179 | LMRG_02744 | N-acetyl-D-glucosamine ABC transporter, permease protein 1 | -4.43 | 0.05608 |
| lmo1129 | LMRG_00571 | Acetyltransferase, GNAT family | -3.42 | 0.05608 |
| lmo2451 | LMRG_01797 | Protein translocase membrane subunit SecG | -5.37 | 0.0575 |
| lmo0556 | LMRG_00238 | Phosphoglycerate mutase family, Lmo0556 homolog | -3.96 | 0.0575 |
| lmo0268 | LMRG_02608 | Phosphoglycerate mutase family protein | -2.87 | 0.06162 |
| lmo1726 | LMRG_02545 | Oxidoreductase, Gfo/Idh/MocA family | -3.31 | 0.06341 |
| lmo0125 | LMRG_02374 | hypothetical protein | -5.6 | 0.06742 |
| lmo0675 | LMRG_02874 | Flagellar motor switch protein FliN | -3.85 | 0.06914 |
| lmo2496 | LMRG_01752 | Phosphate ABC transporter, ATP-binding protein PstB (TC 3.A.1.7.1) | -3.84 | 0.06927 |
| lmo2686 | LMRG_02232 | hypothetical protein | -3.35 | 0.06927 |
| lmo1619 | LMRG_01347 | D-alanine aminotransferase (EC 2.6.1.21) | -3.23 | 0.06927 |
| lmo0203 | LMRG_02625 | Zinc metalloproteinase aureolysin (EC 3.4.24.29) | -3.08 | 0.06927 |
| lmo2127 | LMRG_01281 | CAAX amino terminal protease family protein | -3.34 | 0.07521 |
| lmo1587 | LMRG_01380 | Ornithine carbamoyltransferase (EC 2.1.3.3) | -3.15 | 0.0832 |
| lmo0662 | LMRG_00349 | Novel pyridoxal kinase, thiD family (EC 2.7.1.35) | -3.94 | 0.08472 |
| lmo1768 | LMRG_02503 | Amidophosphoribosyltransferase (EC 2.4.2.14) | -3.79 | 0.09883 |
| lmo2027 | LMRG_01176 | Internalin-like protein Lmo2027 homolog | -2.96 | 0.1044 |
| lmo1050 | LMRG_00511 | hypothetical protein | -3.73 | 0.10781 |
| lmo1521 | LMRG_01449 | N-acetylmuramoyl-L-alanine amidase (EC 3.5.1.28) | -2.96 | 0.11117 |
| lmo0100 | LMRG_02349 | hypothetical protein | -7.36 | 0.1311 |
| lmo0149 | LMRG_02392 | hypothetical protein | -7.21 | 0.1311 |
| lmo2683 | LMRG_02228 | PTS system, cellobiose-specific IIB component (EC 2.7.1.205) | -7.07 | 0.1311 |
| lmo0563 | LMRG_00245 | Imidazole glycerol phosphate synthase cyclase subunit | -6.98 | 0.1311 |
| lmo0784 | LMRG_00472 | PTS system, mannose-specific IIA component (EC 2.7.1.191) | -6.63 | 0.1311 |
| lmo1327 | LMRG_00777 | Ribosome-binding factor A | -6.56 | 0.1311 |
| lmo2812 | LMRG_01886 | D-alanyl-D-alanine carboxypeptidase (EC 3.4.16.4) | -6.29 | 0.1311 |
| lmo0660 | LMRG_00347 | transposase OrfA, IS3 family | -6.26 | 0.1311 |
| lmo0577 | LMRG_00259 | Membrane protein | -6.24 | 0.1311 |
| lmo2736 | LMRG_01960 | Glycerate kinase (EC 2.7.1.31) | -6.15 | 0.1311 |
| lmo0120 | LMRG_02369 | hypothetical protein | -6.12 | 0.1311 |
| lmo2402 | LMRG_01846 | Uncharacterized DUF1027 domain protein YutD | -6.08 | 0.1311 |
| lmo1046 | LMRG_00507 | Cyclic pyranopterin monophosphate synthase (EC 4.6.1.17) | -6.03 | 0.1311 |
| lmo0398 | LMRG_00091 | PTS system, IIA component | -5.96 | 0.1311 |
| lmo2746 | LMRG_01950 | hypothetical protein | -5.95 | 0.1311 |
| lmo1501 | LMRG_01469 | UPF0473 protein YrzB | -5.92 | 0.1311 |
| lmo0916 | LMRG_02016 | PTS system, cellobiose-specific IIA component (EC 2.7.1.205) | -5.91 | 0.1311 |
| lmo1172 | LMRG_00618 | Ethanolamine two-component response regulator | -5.85 | 0.1311 |
| lmo1094 | LMRG_00556 | hypothetical protein | -5.84 | 0.1311 |
| lmo0235 | LMRG_02671 | 2-C-methyl-D-erythritol 4-phosphate cytidylyltransferase (EC 2.7.7.60) | -5.82 | 0.1311 |
| lmo1140 | LMRG_00583 | hypothetical protein | -5.74 | 0.1311 |
| lmo2405 | LMRG_01843 | hypothetical protein | -5.71 | 0.1311 |
| lmo1680 | LMRG_01287 | Cystathionine gamma-synthase (EC 2.5.1.48) @ O-acetylhomoserine sulfhydrylase (EC 2.5.1.49) | -5.67 | 0.1311 |
| lmo2477 | LMRG_01771 | UDP-glucose 4-epimerase (EC 5.1.3.2) | -5.62 | 0.1311 |
| lmo0731 | LMRG_00419 | hypothetical protein | -5.6 | 0.1311 |
| lmo2170 | LMRG_01662 | Enoyl-[acyl-carrier-protein] reductase [FMN, NADH] (EC 1.3.1.9), FabK => refractory to triclosan | -5.58 | 0.1311 |
| lmo0512 | LMRG_00193 | hypothetical protein | -5.55 | 0.1311 |
| lmo0302 | LMRG_02566 | hypothetical protein | -5.51 | 0.1311 |
| lmo2664 | LMRG_02209 | Sorbitol dehydrogenase homologue (EC:1.1.1.14) | -5.5 | 0.1311 |
| lmo2438 | LMRG_01810 | hypothetical protein | -5.39 | 0.1311 |
| lmo1996 | LMRG_01144 | Deoxyribonucleoside regulator DeoR (transcriptional repressor) | -5.32 | 0.1311 |
| lmo0491 | LMRG_00172 | 3-dehydroquinate dehydratase I (EC 4.2.1.10) | -5.3 | 0.1311 |
| lmo0720 | LMRG_00409 | hypothetical protein | -5.18 | 0.1311 |
| lmo1818 | LMRG_00965 | Ribulose-phosphate 3-epimerase (EC 5.1.3.1) | -5.14 | 0.1311 |
| lmo2431 | LMRG_01817 | Ferrichrome-binding periplasmic protein precursor (TC 3.A.1.14.3) | -4.88 | 0.1311 |
| lmo0866 | LMRG_02289 | DEAD-box ATP-dependent RNA helicase DeaD (= CshA) (EC 3.6.4.13) | -4.85 | 0.1311 |
| lmo2117 | LMRG_01271 | Acetyltransferase, GNAT family | -4.79 | 0.1311 |
| lmo2324 | LMRG_02920 | Phage antirepressor protein | -4.79 | 0.1311 |
| lmo0328 | LMRG_00022 | hypothetical protein | -4.76 | 0.1311 |
| lmo0733 | LMRG_00421 | Transcriptional regulator, Xre family | -4.72 | 0.1311 |
| lmo1039 | LMRG_00500 | Molybdenum ABC transporter ATP-binding protein ModC | -4.65 | 0.1311 |
| lmo0414 | LMRG_00106 | Metal transporter, ZIP family | -4.58 | 0.1311 |
| lmo0325 | LMRG_00018 | Transcriptional regulator, MutR family | -4.5 | 0.1311 |
| lmo2236 | LMRG_01596 | Shikimate/quinate 5-dehydrogenase I beta (EC 1.1.1.282) | -4.5 | 0.1311 |
| lmo2696 | LMRG_02001 | Phosphoenolpyruvate-dihydroxyacetone phosphotransferase (EC 2.7.1.121), ADP-binding subunit DhaL | -4.44 | 0.1311 |
| lmo2662 | LMRG_02207 | Ribose-5-phosphate isomerase B (EC 5.3.1.6) | -4.41 | 0.1311 |
| lmo1070 | LMRG_02974 | UPF0358 protein YlaN | -4.4 | 0.1311 |
| lmo0586 | LMRG_00268 | cell surface protein precursor | -4.38 | 0.1311 |
| lmo1245 | LMRG_00691 | hypothetical protein | -4.37 | 0.1311 |
| lmo1910 | LMRG_01057 | Oxidoreductase, FAD-binding | -4.36 | 0.1311 |
| lmo2698 | LMRG_01999 | Transcriptional regulator, RpiR family | -4.31 | 0.1311 |
| lmo1478 | LMRG_00931 | Transcriptional regulator, MerR family | -4.25 | 0.1311 |
| lmo0984 | LMRG_02084 | hypothetical protein | -4.22 | 0.1311 |
| lmo0778 | LMRG_00466 | hypothetical protein | -4.21 | 0.1311 |
| lmo1430 | LMRG_00882 | hypothetical protein | -4.19 | 0.1311 |
| lmo1665 | LMRG_01302 | hypothetical protein | -4.19 | 0.1311 |
| lmo0060 | LMRG_02490 | Putative secretion system component EssB/YukC | -4.18 | 0.1311 |
| lmo1267 | LMRG_00716 | Cell division trigger factor (EC 5.2.1.8) | -4.1 | 0.1311 |
| lmo1777 | LMRG_02835 | HD domain protein | -4.09 | 0.1311 |
| lmo2234 | LMRG_01598 | Xylose isomerase domain protein TIM barrel | -4.09 | 0.1311 |
| lmo0564 | LMRG_00246 | Phosphoribosylformimino-5-aminoimidazole carboxamide ribotide isomerase (EC 5.3.1.16) | -4.06 | 0.1311 |
| lmo0793 | LMRG_00481 | Uncharacterized DUF554 membrane protein | -3.99 | 0.1311 |
| lmo2131 | LMRG_02809 | hypothetical protein | -3.99 | 0.1311 |
| lmo1789 | LMRG_02813 | flavodoxin-like fold domain protein | -3.97 | 0.1311 |
| lmo2427 | LMRG_01821 | Rod shape-determining protein RodA | -3.96 | 0.1311 |
| lmo2833 | LMRG_01865 | Uncharacterized glycosyl hydrolase YcjT | -3.92 | 0.1311 |
| lmo0871 | LMRG_02295 | Transcriptional regulator, HxlR family | -3.91 | 0.1311 |
| lmo0647 | LMRG_00334 | hypothetical protein | -3.9 | 0.1311 |
| lmo1498 | LMRG_01472 | FIG011945: O-methyltransferase family protein | -3.89 | 0.1311 |
| lmo1596a | LMRG_02946 | FIG00774215: hypothetical protein | -3.89 | 0.1311 |
| lmo0361 | LMRG_00053 | Twin-arginine translocation protein TatCy | -3.82 | 0.1311 |
| lmo1495 | LMRG_01475 | Hypothetical protein perhaps functionally coupled to transcription elongation factor GreA | -3.66 | 0.1311 |
| lmo0300 | LMRG_02568 | beta-glucosidase (EC 3.2.1.21) | -3.57 | 0.1311 |
| lmo1682 | LMRG_01285 | Uncharacterized MFS-type transporter | -3.57 | 0.1311 |
| lmo1227 | LMRG_00673 | Uracil-DNA glycosylase, family 1 (EC 3.2.2.27) | -3.55 | 0.1311 |
| lmo2300 | LMRG_01532 | putative terminase large subunit | -3.5 | 0.1311 |
| lmo1831 | LMRG_00978 | Orotate phosphoribosyltransferase (EC 2.4.2.10) | -3.48 | 0.1311 |
| lmo0477 | LMRG_00158 | putative secreted protein | -3.46 | 0.1311 |
| lmos45 | LMRG_00698 | hypothetical protein | -3.4 | 0.1311 |
| lmo1423 | LMRG_00875 | hypothetical protein | -3.34 | 0.1311 |
| lmo2173 | LMRG_01659 | sigma-54-dependent transcriptional regulator | -3.33 | 0.1311 |
| lmo0711 | LMRG_00400 | Flagellar basal-body rod protein FlgC | -3.3 | 0.1311 |
| lmo0915 | LMRG_02015 | PTS system, cellobiose-specific IIC component | -3.29 | 0.1311 |
| lmo1663 | LMRG_01304 | Asparagine synthetase [glutamine-hydrolyzing] (EC 6.3.5.4) AsnB | -3.26 | 0.1311 |
| lmo0719 | LMRG_00408 | Transcriptional regulator, PadR family | -3.24 | 0.1311 |
| lmo0678 | LMRG_00366 | Flagellar biosynthesis protein FliR | -3.12 | 0.1311 |
| lmo0380 | LMRG_00073 | hypothetical protein | -3.05 | 0.1311 |
| lmo2525 | LMRG_01723 | MreB-like protein (Mbl protein) | -3.04 | 0.1311 |
| lmo1811 | LMRG_00958 | ATP-dependent DNA helicase RecG (EC 3.6.4.12) | -2.95 | 0.1311 |
| #N/A | LMRG_02135 | CRISPR-associated protein, Csn2 family | -2.85 | 0.1311 |
| lmo1932 | LMRG_01079 | Heptaprenyl diphosphate synthase component I (EC 2.5.1.30) | -2.83 | 0.1311 |
| lmo2732 | LMRG_01964 | phosphosugar-binding protein | -2.76 | 0.1311 |
| lmo1371 | LMRG_00821 | Dihydrolipoamide dehydrogenase of branched-chain alpha-keto acid dehydrogenase (EC 1.8.1.4) | -2.74 | 0.1311 |
| lmo2805 | LMRG_01891 | putative secreted protein | -2.74 | 0.1311 |
| #N/A | LMRG_02895 | hypothetical protein | -2.74 | 0.1311 |
| lmo1633 | LMRG_01333 | Anthranilate synthase, aminase component (EC 4.1.3.27) | -2.66 | 0.1311 |
| lmo0956 | LMRG_02055 | N-acetylglucosamine-6-phosphate deacetylase (EC 3.5.1.25) | -2.64 | 0.1311 |
| lmo1363 | LMRG_00813 | (2E,6E)-farnesyl diphosphate synthase (EC 2.5.1.10) | -2.56 | 0.1311 |
| lmo0777 | LMRG_00465 | hypothetical protein | -2.55 | 0.1311 |
| lmo1365 | LMRG_00815 | 1-deoxy-D-xylulose 5-phosphate synthase (EC 2.2.1.7) | -2.53 | 0.1311 |
| lmo0157 | LMRG_02402 | DinG family ATP-dependent helicase CPE1197 | -2.46 | 0.1311 |
| lmo1434 | LMRG_00886 | Ribonuclease J2 (endoribonuclease in RNA processing) | -2.42 | 0.1311 |
| #N/A | LMRG_02138 | CRISPR-associated endonuclease Cas9 | -2.36 | 0.1311 |
| lmo1084 | LMRG_00546 | dTDP-4-dehydrorhamnose reductase (EC 1.1.1.133) | -2.35 | 0.1311 |
| lmo0930 | LMRG_02029 | Metal-dependent hydrolases of the beta-lactamase superfamily I | -2.34 | 0.1311 |
| lmo1325 | LMRG_00775 | Translation initiation factor 2 | -2.2 | 0.1311 |
| lmo1769 | LMRG_02502 | Phosphoribosylformylglycinamidine synthase, synthetase subunit (EC 6.3.5.3) | -2.14 | 0.1311 |
| lmo1909 | LMRG_01056 | Uncharacterized UPF0750 membrane protein YpjC | -2.12 | 0.1311 |
| lmo2005 | LMRG_01153 | Uncharacterized oxidoreductase, YajO family | -1.94 | 0.1311 |
| lmo0219 | LMRG_02641 | tRNA(Ile)-lysidine synthetase (EC 6.3.4.19) / Hypoxanthine-guanine phosphoribosyltransferase (EC 2.4.2.8) | -1.29 | 0.1311 |
| lmo2113 | LMRG_01267 | Coproheme decarboxylase HemQ (no EC) | -1.01 | 0.1311 |
| lmo1901 | LMRG_01048 | Pantoate--beta-alanine ligase (EC 6.3.2.1) | -0.45 | 0.1311 |
| lmo1294 | LMRG_00744 | tRNA dimethylallyltransferase (EC 2.5.1.75) | -4.82 | 0.13237 |
| lmo1878 | LMRG_01024 | Mn-dependent transcriptional regulator MntR | -3.43 | 0.13237 |
| lmo1429 | LMRG_00881 | Substrate-specific component ThiT of thiamin ECF transporter | -3.65 | 0.13248 |
| lmo1023 | LMRG_02123 | KtrAB potassium uptake system, peripheral membrane component KtrA | -3.41 | 0.13248 |
| lmo2352 | LMRG_01491 | LysR-family transcriptional regulator Bsu YtlI | -3.61 | 0.1331 |
| lmo1775 | LMRG_02496 | N5-carboxyaminoimidazole ribonucleotide mutase (EC 5.4.99.18) | -6.18 | 0.13331 |
| lmo0774 | LMRG_00462 | Diacylglycerol kinase-related protein | -3.85 | 0.13331 |
| lmo1180 | LMRG_00626 | Ethanolamine utilization protein similar to PduA/PduJ | -3.63 | 0.13331 |
| lmo1786 | LMRG_02825 | Internalin C | -3.23 | 0.13331 |
| lmo2692 | LMRG_02239 | protein from nitrogen regulatory protein P-II (GLNB) family, ortholog YAAQ B. subtilis | -3.82 | 0.14003 |
| lmo0124 | LMRG_02373 | hypothetical protein | -3.57 | 0.14003 |
| lmo0704 | LMRG_00393 | hypothetical protein | -3.29 | 0.1439 |
| lmo1447 | LMRG_00899 | Zinc ABC transporter, ATP-binding protein ZnuC | -3.47 | 0.14446 |
| lmo0714 | LMRG_00403 | Flagellar motor switch protein FliG | -3.28 | 0.14502 |
| lmo1299 | LMRG_00749 | Glutamine synthetase type I (EC 6.3.1.2) | -2.01 | 0.151 |
| lmo0943 | LMRG_02041 | DNA protection during starvation protein | -3.24 | 0.15478 |
| lmo2114 | LMRG_01268 | ABC transporter-like sensor ATP-binding protein Bsel_2651 | -3.07 | 0.16392 |
| lmo1082 | LMRG_00544 | dTDP-4-dehydrorhamnose 3,5-epimerase (EC 5.1.3.13) | -2.97 | 0.16439 |
| lmo1854 | LMRG_01001 | Repressor CsoR of the copZA operon | -2.83 | 0.16808 |
| lmo2701 | LMRG_01996 | Uncharacterized protein YaaL | -5.05 | 0.1743 |
| lmo2003 | LMRG_01151 | Transcriptional regulator, GntR family | -3.52 | 0.1743 |
| lmo1565 | LMRG_01402 | DNA polymerase I (EC 2.7.7.7) | -2.96 | 0.17791 |
| lmo1855 | LMRG_01002 | D-alanyl-D-alanine carboxypeptidase (EC 3.4.16.4) | -2.19 | 0.17833 |
| lmo2258 | LMRG_01573 | ribulose-phosphate 3-epimerase family protein | -3.19 | 0.18085 |
| lmo0895 | LMRG_02319 | RNA polymerase sigma factor SigB | -5.25 | 0.18165 |
| lmo1417 | LMRG_00869 | Uncharacterized MFS-type transporter YxiO | -1.16 | 0.18165 |
| lmo1906 | LMRG_01053 | Methylglyoxal synthase (EC 4.2.3.3) | -6.22 | 0.18413 |
| lmo2298 | LMRG_01534 | Minor capsid protein [Bacteriophage A118] / Phage minor capsid protein | -3.14 | 0.18594 |
| lmo2530 | LMRG_01718 | ATP synthase gamma chain (EC 3.6.3.14) | -2.97 | 0.18594 |
| lmo2437 | LMRG_01811 | hypothetical protein | -4.25 | 0.19249 |
| lmo2284 | LMRG_01548 | Putative tail or base plate protein gp19 [Bacteriophage A118] | -1.31 | 0.19387 |
| lmo2359 | LMRG_01483 | Cof-like hydrolase | -3.8 | 0.19524 |
| lmo1387 | LMRG_00839 | Pyrroline-5-carboxylate reductase (EC 1.5.1.2), ProG-like | -3.11 | 0.20068 |
| lmo2840 | LMRG_01858 | Uncharacterized glycosyltransferase YcjM | -3.19 | 0.20202 |
| lmo0015 | LMRG_02444 | Cytochrome aa3-600 menaquinol oxidase subunit III | -3.5 | 0.20335 |
| lmo0292 | LMRG_02579 | Serine protease, DegP/HtrA, do-like (EC 3.4.21.-) | -1.45 | 0.20366 |
| lmo2499 | LMRG_01749 | Phosphate ABC transporter, substrate-binding protein PstS (TC 3.A.1.7.1) | -2.85 | 0.20397 |
| #N/A | LMRG_02936 | FIG00775722: hypothetical protein | -4.73 | 0.20428 |
| lmo1564 | LMRG_01403 | Formamidopyrimidine-DNA glycosylase (EC 3.2.2.23) | -3 | 0.20458 |
| lmo0076 | LMRG_02327 | Methylphosphotriester-DNA--protein-cysteine S-methyltransferase (EC 2.1.1.n11) / ADA regulatory protein / Methylated-DNA--protein-cysteine methyltransferase (EC 2.1.1.63) | -3.26 | 0.20688 |
| lmo1805 | LMRG_00952 | Ribonuclease III (EC 3.1.26.3) | -2.65 | 0.23393 |
| lmo2598 | LMRG_02142 | tRNA pseudouridine(38-40) synthase (EC 5.4.99.12) | -2.9 | 0.2371 |
| lmo1570 | LMRG_01397 | Pyruvate kinase (EC 2.7.1.40) / Phosphohistidine swiveling domain | -2.48 | 0.24025 |
| lmo2807 | LMRG_01889 | putative secreted protein | -2.98 | 0.24043 |
| lmo0434 | LMRG_00127 | Internalin B (GW modules) | -2.2 | 0.25039 |
| lmo1753 | LMRG_02518 | Transcription regulator [contains diacylglycerol kinase catalytic domain] | -2.88 | 0.25053 |
| lmo2296 | LMRG_01536 | Phage capsid protein | -3.31 | 0.25663 |
| lmo2649 | LMRG_02194 | PTS system, IIC component, UlaA-type | -2.88 | 0.25663 |
| lmo0424 | LMRG_00116 | Sugar:proton symporter | -3.24 | 0.25772 |
| lmo2853 | LMRG_02424 | RNA-binding protein Jag | -2.64 | 0.25784 |
| lmo0506 | LMRG_00187 | Galactitol-1-phosphate 5-dehydrogenase (EC 1.1.1.251) | -3.3 | 0.26084 |
| lmo2253 | LMRG_01578 | Hydrolase, haloacid dehalogenase-like family | -3.38 | 0.26201 |
| lmo1494 | LMRG_01476 | 5'-methylthioadenosine nucleosidase (EC 3.2.2.16) @ S-adenosylhomocysteine nucleosidase (EC 3.2.2.9) | -3.17 | 0.26201 |
| lmo0691 | LMRG_00379 | Chemotaxis regulator - transmits chemoreceptor signals to flagellar motor components CheY | -2.53 | 0.26971 |
| lmo2021 | LMRG_01170 | Transcriptional repressor for NAD biosynthesis in gram-positives | -3.06 | 0.27547 |
| lmo1216 | LMRG_00662 | N-acetylmuramoyl-L-alanine amidase, family 4 | -2.07 | 0.28402 |
| lmo0400 | LMRG_00093 | PTS system, IIC component | -2.63 | 0.28688 |
| lmo2512 | LMRG_01736 | Competence protein F homolog, phosphoribosyltransferase domain | -2.64 | 0.29909 |
| lmo0296 | LMRG_02572 | Membrane protein | -2.9 | 0.30001 |
| lmo2204 | LMRG_01628 | hypothetical protein | -2.52 | 0.30278 |
| lmo1809 | LMRG_00956 | Phosphate:acyl-ACP acyltransferase PlsX (EC 2.3.1.n2) | -2.93 | 0.31297 |
| lmo2493 | LMRG_01755 | Transcriptional repressor CzrA, ArsR family | -2.97 | 0.31726 |
| lmo0352 | LMRG_00043 | Transcriptional regulator, DeoR family | -2.79 | 0.31726 |
| lmo2847 | LMRG_02418 | Rhamnulose-1-phosphate aldolase (EC 4.1.2.19) | -2.79 | 0.31726 |
| lmo1815 | LMRG_00962 | FIG001802: Putative alkaline-shock protein | -2.76 | 0.31726 |
| lmo1603 | LMRG_01364 | Aminopeptidase | -2.46 | 0.31726 |
| lmo0962 | LMRG_02061 | LemA protein | -2.71 | 0.32083 |
| lmo0589 | LMRG_00271 | hypothetical protein | -2.76 | 0.32257 |
| lmo2772 | LMRG_01923 | PTS system, beta-glucoside-specific IIB component / PTS system, beta-glucoside-specific IIC component / PTS system, beta-glucoside-specific IIA component | -1.6 | 0.32429 |
| lmo2094 | LMRG_01245 | class II aldolase/adducin domain protein | -2.35 | 0.33323 |
| lmo2641 | LMRG_02186 | Heptaprenyl diphosphate synthase component II (EC 2.5.1.30) | -2.25 | 0.33671 |
| lmo0189 | LMRG_02754 | Veg protein | -2.67 | 0.33748 |
| lmo0273 | LMRG_02603 | Acetyltransferase, GNAT family | -3.02 | 0.35614 |
| lmo2681 | LMRG_02226 | Potassium-transporting ATPase B chain (EC 3.6.3.12) (TC 3.A.3.7.1) | -2.95 | 0.39967 |
| lmo1636 | LMRG_01330 | Efflux ABC transporter, ATP-binding protein | -2.74 | 0.40736 |
| lmo0271 | LMRG_02605 | 6-phospho-beta-glucosidase (EC 3.2.1.86) | -1.97 | 0.41411 |
| lmo2513 | LMRG_01735 | ComF operon protein A, DNA transporter ATPase | -2.39 | 0.41994 |
| lmo2334 | LMRG_01509 | DNA-binding protein | -2.38 | 0.42007 |
| lmo1861 | LMRG_01008 | YfaA | -1.77 | 0.42007 |
| lmo0993 | LMRG_02093 | KtrCD potassium uptake system, integral membrane component KtrD | -2.26 | 0.42758 |
| lmo1136 | LMRG_00579 | Internalin-like protein (LPXTG motif) Lmo1136 homolog | -2.12 | 0.43376 |
| lmo2557 | LMRG_01690 | Transcription regulator [contains diacylglycerol kinase catalytic domain] | -1.68 | 0.43376 |
| lmo1913 | LMRG_01060 | hypothetical protein | -2.6 | 0.43422 |
| lmo2581 | LMRG_02686 | Heme efflux system permease HrtB | -2.32 | 0.44853 |
| lmo2060 | LMRG_01210 | hypothetical protein | -2.09 | 0.44894 |
| lmo0105 | LMRG_02354 | Chitinase (EC 3.2.1.14) | -3.04 | 0.45279 |
| lmo2546 | LMRG_01701 | Threonine synthase (EC 4.2.3.1) | -2.82 | 0.4592 |
| lmo0670 | LMRG_00358 | hypothetical protein | -8.67 | 0.45987 |
| lmot01 | LMRG_05001 | tRNA-Lys-CTT | -8.62 | 0.45987 |
| lmot02 | LMRG_05002 | tRNA-Val-TAC | -7.34 | 0.45987 |
| lmo2802a | LMRG_01894 | hypothetical protein | -7.22 | 0.45987 |
| lmo0693 | LMRG_00381 | Flagellar motor switch protein FliN | -7.15 | 0.45987 |
| lmot38 | LMRG_05038 | tRNA-Val-TAC | -7.12 | 0.45987 |
| lmot15 | LMRG_05015 | tRNA-Asp-GTC | -6.82 | 0.45987 |
| lmot25 | LMRG_05025 | tRNA-Asp-GTC | -6.59 | 0.45987 |
| lmo1986 | LMRG_01134 | Ketol-acid reductoisomerase (NADP(+)) (EC 1.1.1.86) | -6.58 | 0.45987 |
| lmo2574 | LMRG_02694 | hypothetical protein | -6.57 | 0.45987 |
| lmot44 | LMRG_05044 | tRNA-Gln-TTG | -6.48 | 0.45987 |
| lmo2272 | #N/A | hypothetical protein | -6.43 | 0.45987 |
| lmot54 | LMRG_05054 | tRNA-Asp-GTC | -6.37 | 0.45987 |
| lmo2651 | LMRG_02196 | PTS system, IIA component | -6.33 | 0.45987 |
| lmo1185 | LMRG_00631 | Ethanolamine utilization protein similar to PduT | -6.29 | 0.45987 |
| lmo0542 | LMRG_00224 | PTS system, glucitol/sorbitol-specific IIA component (EC 2.7.1.198) | -5.91 | 0.45987 |
| lmo2259 | LMRG_01572 | PTS system, beta-glucoside-specific, IIA component | -5.91 | 0.45987 |
| lmo2647 | LMRG_02192 | Creatinine amidohydrolase (EC 3.5.2.10) | -5.68 | 0.45987 |
| lmo1220 | LMRG_00666 | Transcriptional regulator YodB, HxlR family | -5.65 | 0.45987 |
| lmo0128 | LMRG_02377 | Holin | -5.62 | 0.45987 |
| lmos49 | #N/A | hypothetical protein | -5.6 | 0.45987 |
| lmo1469 | LMRG_00922 | SSU ribosomal protein S21p | -5.58 | 0.45987 |
| lmo1832 | LMRG_00979 | Orotidine 5'-phosphate decarboxylase (EC 4.1.1.23) | -5.52 | 0.45987 |
| lmo2532 | LMRG_01716 | ATP synthase delta chain (EC 3.6.3.14) | -5.5 | 0.45987 |
| lmo0351 | LMRG_00042 | Phosphoenolpyruvate-dihydroxyacetone phosphotransferase (EC 2.7.1.121), subunit DhaM | -5.44 | 0.45987 |
| lmo2832 | LMRG_01866 | Glycerate kinase (EC 2.7.1.31) | -5.43 | 0.45987 |
| lmo0759 | LMRG_00447 | Glyoxalase family protein | -5.4 | 0.45987 |
| #N/A | LMRG_02847 | hypothetical protein | -5.4 | 0.45987 |
| lmo0683 | LMRG_00371 | Chemotaxis protein methyltransferase CheR (EC 2.1.1.80) | -5.39 | 0.45987 |
| lmo0792 | LMRG_00480 | Hydrolase, carbon-nitrogen family | -5.25 | 0.45987 |
| lmo1944 | LMRG_01091 | Ferredoxin | -5.15 | 0.45987 |
| lmo1608 | LMRG_01358 | FIG002434: Uncharacterized protein YtpQ | -5.1 | 0.45987 |
| lmo2703 | LMRG_01994 | Nucleoid-associated protein YaaK | -5.09 | 0.45987 |
| lmo0844 | LMRG_02267 | RidA/YER057c/UK114 superfamily protein | -5.08 | 0.45987 |
| lmo1517 | LMRG_01453 | Nitrogen regulatory protein P-II | -5.02 | 0.45987 |
| lmo2098 | LMRG_01249 | PTS system, galactitol-specific IIA component (EC 2.7.1.200) | -4.96 | 0.45987 |
| lmo0131 | LMRG_02380 | hypothetical protein | -4.89 | 0.45987 |
| lmo2061 | LMRG_01211 | Conserved membrane protein in copper uptake, YcnI | -4.89 | 0.45987 |
| lmo2760a | LMRG_01935 | hypothetical protein | -4.83 | 0.45987 |
| lmo1133 | LMRG_00575 | hypothetical protein | -4.74 | 0.45987 |
| lmo1487 | LMRG_00940 | Hydrolase (HAD superfamily), YqeK | -4.71 | 0.45987 |
| lmo0350 | LMRG_00041 | hypothetical protein | -4.7 | 0.45987 |
| lmo2047 | LMRG_01197 | LSU ribosomal protein L32p @ LSU ribosomal protein L32p, zinc-dependent | -4.69 | 0.45987 |
| lmo1157 | LMRG_00600 | Propanediol dehydratase reactivation factor small subunit | -4.66 | 0.45987 |
| lmo2729 | LMRG_01967 | Transcriptional regulator | -4.64 | 0.45987 |
| lmo2151 | LMRG_01681 | hypothetical protein | -4.62 | 0.45987 |
| lmo0212 | LMRG_02634 | Acetyltransferase, GNAT family | -4.57 | 0.45987 |
| lmo2080 | LMRG_01231 | hypothetical protein | -4.56 | 0.45987 |
| lmo1194 | LMRG_00640 | Cobalt-precorrin-5B (C1)-methyltransferase (EC 2.1.1.195) | -4.52 | 0.45987 |
| lmo1304 | LMRG_00754 | UPF0291 protein YnzC | -4.52 | 0.45987 |
| lmo0638 | LMRG_00322 | Glutamyl endopeptidase precursor (EC 3.4.21.19), blaSE | -4.51 | 0.45987 |
| lmo0863 | LMRG_02286 | hypothetical protein | -4.44 | 0.45987 |
| lmo1358 | LMRG_00808 | Alkaline shock protein | -4.4 | 0.45987 |
| lmo1022 | LMRG_02122 | Cell envelope stress response system LiaFSR, response regulator LiaR(VraR) | -4.36 | 0.45987 |
| lmo1247 | LMRG_00693 | hypothetical protein | -4.28 | 0.45987 |
| lmo1181 | LMRG_00627 | ATP:Cob(I)alamin adenosyltransferase (EC 2.5.1.17) @ ATP:Cob(I)alamin adenosyltransferase (EC 2.5.1.17), ethanolamine utilization | -4.24 | 0.45987 |
| lmo1724 | LMRG_02547 | Efflux ABC transporter, ATP-binding protein | -4.22 | 0.45987 |
| lmo1236 | LMRG_00682 | Uncharacterized protein YslB | -4.21 | 0.45987 |
| lmo1779 | LMRG_02833 | hypothetical protein | -4.21 | 0.45987 |
| lmo0967 | LMRG_02066 | GTP pyrophosphokinase (EC 2.7.6.5) | -4.18 | 0.45987 |
| lmo2676 | LMRG_02221 | Putative UV-damage repair protein UvrX | -4.15 | 0.45987 |
| #N/A | LMRG_05001 | hypothetical protein | -4.12 | 0.45987 |
| lmo0710 | LMRG_00399 | Flagellar basal-body rod protein FlgB | -4.06 | 0.45987 |
| lmo1516 | LMRG_01454 | Ammonium transporter | -4.04 | 0.45987 |
| lmo2346 | LMRG_01497 | L-cystine ABC transporter, ATP-binding protein TcyN | -3.97 | 0.45987 |
| lmo0260 | LMRG_02649 | hypothetical protein | -3.87 | 0.45987 |
| lmo2280 | LMRG_01552 | Protein gp23 [Bacteriophage A118] | -3.84 | 0.45987 |
| lmo0316 | LMRG_00009 | Hydroxyethylthiazole kinase (EC 2.7.1.50) | -3.81 | 0.45987 |
| lmo0501 | LMRG_00182 | Predicted galactitol operon regulator (Transcriptional antiterminator), BglG family / PTS system, IIA component | -3.78 | 0.45987 |
| lmo0845 | LMRG_02268 | Methionine synthase II (cobalamin-independent) | -3.77 | 0.45987 |
| lmo1021 | LMRG_02121 | Cell envelope stress response system LiaFSR, sensor histidine kinase LiaS(VraS) | -3.76 | 0.45987 |
| lmo0117 | LMRG_02366 | Listeria protein LmaB, associated with virulence | -3.74 | 0.45987 |
| lmo0167 | LMRG_02412 | 16S rRNA (cytidine(1402)-2'-O)-methyltransferase (EC 2.1.1.198) | -3.74 | 0.45987 |
| lmo0657 | LMRG_00344 | hypothetical protein | -3.74 | 0.45987 |
| lmo1308 | LMRG_00758 | SAM-dependent methyltransferase | -3.74 | 0.45987 |
| lmo1395 | LMRG_00847 | Transcriptional regulator in cluster with unspecified monosaccharide ABC transport system | -3.72 | 0.45987 |
| lmo2099 | LMRG_01250 | Putative transcriptional antiterminator, BglG family / PTS system, IIA component | -3.72 | 0.45987 |
| lmo0150 | LMRG_02394 | FIG00774738: hypothetical protein | -3.69 | 0.45987 |
| lmo0953 | LMRG_02052 | hypothetical protein | -3.66 | 0.45987 |
| lmo1009 | LMRG_02109 | CBS domain-containing protein YkuL | -3.66 | 0.45987 |
| lmo2227 | LMRG_01605 | Efflux ABC transporter, ATP-binding protein | -3.66 | 0.45987 |
| lmo0905 | LMRG_02005 | hypothetical protein | -3.65 | 0.45987 |
| lmo1720 | LMRG_02551 | PTS system, cellobiose-specific IIB component (EC 2.7.1.205) | -3.65 | 0.45987 |
| lmo2191 | LMRG_01641 | Regulatory protein Spx | -3.65 | 0.45987 |
| lmo0966 | LMRG_02065 | hypothetical protein | -3.62 | 0.45987 |
| lmo1956 | LMRG_01103 | Ferric uptake regulation protein FUR | -3.6 | 0.45987 |
| lmo1348 | LMRG_00798 | Aminomethyltransferase (glycine cleavage system T protein) (EC 2.1.2.10) | -3.59 | 0.45987 |
| lmo0505 | LMRG_00186 | Ribulose-phosphate 3-epimerase (EC 5.1.3.1) | -3.58 | 0.45987 |
| lmo0413 | LMRG_00105 | hypothetical protein | -3.53 | 0.45987 |
| lmo1201 | LMRG_00647 | Uroporphyrinogen-III methyltransferase (EC 2.1.1.107) / Uroporphyrinogen-III synthase (EC 4.2.1.75) | -3.52 | 0.45987 |
| lmo0631 | LMRG_00314 | PTS system, fructose-specific IIA component (EC 2.7.1.202) | -3.45 | 0.45987 |
| lmo1324 | LMRG_00774 | Ribosomal protein L7Ae family protein YlxQ | -3.42 | 0.45987 |
| lmo1195 | LMRG_00641 | Cobalt-precorrin-7 (C5)-methyltransferase (EC 2.1.1.289) | -3.4 | 0.45987 |
| lmo1433 | LMRG_00885 | similar to glutathione reductase | -3.39 | 0.45987 |
| lmo1646 | LMRG_01320 | Exonuclease SbcD | -3.38 | 0.45987 |
| lmo1483 | LMRG_00936 | dCMP deaminase (EC 3.5.4.12) @ Late competence protein ComEB | -3.32 | 0.45987 |
| lmo2193 | LMRG_01639 | Oligopeptide ABC transporter, ATP-binding protein OppD (TC 3.A.1.5.1) | -3.31 | 0.45987 |
| lmo0561 | LMRG_00243 | Phosphoribosyl-ATP pyrophosphatase (EC 3.6.1.31) | -3.29 | 0.45987 |
| lmo1460 | LMRG_00912 | DNA recombination and repair protein RecO | -3.27 | 0.45987 |
| lmo2460 | LMRG_01788 | Central glycolytic genes regulator | -3.26 | 0.45987 |
| #N/A | LMRG_02137 | CRISPR-associated protein Cas1 | -3.24 | 0.45987 |
| lmo0562 | LMRG_00244 | Phosphoribosyl-AMP cyclohydrolase (EC 3.5.4.19) | -3.22 | 0.45987 |
| lmo0416 | LMRG_00108 | Transcriptional regulator, Xre family | -3.2 | 0.45987 |
| lmo2475 | LMRG_01773 | Phosphoglucomutase (EC 5.4.2.2) | -3.17 | 0.45987 |
| lmo2297 | LMRG_01535 | Phage capsid scaffolding protein | -3.13 | 0.45987 |
| lmo1336 | LMRG_00786 | 5-formyltetrahydrofolate cyclo-ligase (EC 6.3.3.2) | -3.1 | 0.45987 |
| lmo1269 | LMRG_00719 | Signal peptidase I (EC 3.4.21.89) | -3.08 | 0.45987 |
| lmo2799 | LMRG_01898 | PTS system, mannitol-specific IIC component / PTS system, mannitol-specific IIB component (EC 2.7.1.197) | -3.08 | 0.45987 |
| lmo1159 | LMRG_00602 | Propanediol utilization polyhedral body protein PduJ | -3.07 | 0.45987 |
| lmo2009 | LMRG_01157 | ABC transporter, permease protein 1 (cluster 1, maltose/g3p/polyamine/iron) | -3.07 | 0.45987 |
| lmo0996 | LMRG_02096 | Methylated-DNA--protein-cysteine methyltransferase (EC 2.1.1.63) | -3.04 | 0.45987 |
| lmo1653 | LMRG_01314 | Putative cellsurface protein | -3.04 | 0.45987 |
| lmo2749 | LMRG_01947 | Para-aminobenzoate synthase, amidotransferase component (EC 2.6.1.85) | -3.04 | 0.45987 |
| lmo2796 | LMRG_01901 | N-acetylmannosamine kinase (EC 2.7.1.60) | -3.04 | 0.45987 |
| lmo1708 | LMRG_02562 | weak similarity to aminoglycoside N(3)-acetyltransferase | -3.02 | 0.45987 |
| lmo1998 | LMRG_01146 | Putative glucosamine-fructose-6-phosphate aminotransferase | -3.02 | 0.45987 |
| lmo2182 | LMRG_01650 | Heme transporter analogous to IsdDEF, ATP-binding protein | -2.99 | 0.45987 |
| lmo1058 | LMRG_00520 | UPF0223 protein YktA | -2.97 | 0.45987 |
| lmo1150 | LMRG_00593 | Propanediol utilization transcriptional activator | -2.97 | 0.45987 |
| lmo2122 | LMRG_01276 | Maltodextrose utilization protein YvdJ | -2.97 | 0.45987 |
| lmo2515 | LMRG_01733 | Two-component transcriptional response regulator DegU, LuxR family | -2.97 | 0.45987 |
| lmo1935 | LMRG_01082 | Protein tyrosine phosphatase (EC 3.1.3.48) | -2.96 | 0.45987 |
| lmo2111 | LMRG_01265 | FMN reductase [NAD(P)H] (EC 1.5.1.39) | -2.96 | 0.45987 |
| lmo2271 | LMRG_01558 | hypothetical protein | -2.96 | 0.45987 |
| lmo1848 | LMRG_00995 | Manganese ABC transporter, inner membrane permease protein SitD | -2.95 | 0.45987 |
| lmo1425 | LMRG_00877 | Choline ABC transport system, permease protein OpuBD | -2.94 | 0.45987 |
| lmo1835 | LMRG_00982 | Carbamoyl-phosphate synthase large chain (EC 6.3.5.5) | -2.94 | 0.45987 |
| lmo1155 | LMRG_00598 | Propanediol dehydratase small subunit (EC 4.2.1.28) | -2.93 | 0.45987 |
| lmo1749 | LMRG_02522 | Shikimate kinase I (EC 2.7.1.71) | -2.91 | 0.45987 |
| lmo1342 | LMRG_00792 | Late competence protein ComGF, access of DNA to ComEA, FIG012620 | -2.9 | 0.45987 |
| lmo1512 | LMRG_01458 | tRNA-specific 2-thiouridylase MnmA (EC 2.8.1.13) | -2.89 | 0.45987 |
| lmo0595 | LMRG_00277 | O-acetylhomoserine sulfhydrylase (EC 2.5.1.49) @ O-succinylhomoserine sulfhydrylase (EC 2.5.1.48) | -2.87 | 0.45987 |
| lmo0716 | LMRG_00405 | Flagellum-specific ATP synthase FliI | -2.87 | 0.45987 |
| lmo0768 | LMRG_00456 | ABC transporter, substrate-binding protein (cluster 1, maltose/g3p/polyamine/iron) | -2.87 | 0.45987 |
| lmo0004 | LMRG_02432 | Uncharacterized S4 RNA-binding-domain protein YbcJ | -2.86 | 0.45987 |
| lmo1868 | LMRG_01015 | Lactoylglutathione lyase | -2.86 | 0.45987 |
| lmo2699 | LMRG_01998 | Cof-like hydrolase | -2.84 | 0.45987 |
| lmo1526 | LMRG_01444 | Uncharacterized membrane protein YrvD | -2.82 | 0.45987 |
| lmo2497 | LMRG_01751 | Phosphate ABC transporter, permease protein PstA (TC 3.A.1.7.1) | -2.81 | 0.45987 |
| lmo0797 | LMRG_00485 | Transcriptional regulator, MarR family | -2.79 | 0.45987 |
| lmo2382 | LMRG_02727 | Na(+) H(+) antiporter subunit E | -2.77 | 0.45987 |
| lmo2785 | LMRG_01912 | Catalase KatE (EC 1.11.1.6) | -2.77 | 0.45987 |
| lmo2309 | LMRG_01524 | Phage exonuclease (EC 3.1.11.3) | -2.76 | 0.45987 |
| #N/A | LMRG_02908 | FIG00775940: hypothetical protein | -2.76 | 0.45987 |
| lmo2602 | LMRG_02146 | Mg(2+)-transport-ATPase-associated protein MgtC | -2.75 | 0.45987 |
| lmo2074 | LMRG_01225 | hypothetical protein | -2.73 | 0.45987 |
| lmo2648 | LMRG_02193 | phosphotriesterase family protein | -2.71 | 0.45987 |
| lmo1945 | LMRG_01092 | Substrate-specific component RibU of riboflavin ECF transporter | -2.69 | 0.45987 |
| lmo2650 | LMRG_02195 | PTS system, lactose/cellobiose specific IIB subunit | -2.69 | 0.45987 |
| lmo1332 | LMRG_00782 | Probable GTPase related to EngC | -2.68 | 0.45987 |
| lmo1139 | LMRG_00582 | hypothetical protein | -2.67 | 0.45987 |
| lmo0344 | LMRG_00035 | Oxidoreductase, short-chain dehydrogenase/reductase family | -2.66 | 0.45987 |
| lmo1927 | LMRG_01074 | 3-dehydroquinate synthase (EC 4.2.3.4) | -2.66 | 0.45987 |
| lmo2613 | LMRG_02157 | LSU ribosomal protein L15p (L27Ae) | -2.66 | 0.45987 |
| lmo0886 | LMRG_02310 | Alanine racemase (EC 5.1.1.1) | -2.65 | 0.45987 |
| lmo1765 | LMRG_02506 | IMP cyclohydrolase (EC 3.5.4.10) / Phosphoribosylaminoimidazolecarboxamide formyltransferase (EC 2.1.2.3) | -2.64 | 0.45987 |
| lmo1177 | LMRG_00623 | Ethanolamine utilization polyhedral-body-like protein EutL | -2.61 | 0.45987 |
| lmo2029 | LMRG_01178 | Cell division integral membrane protein, YggT and half-length relatives | -2.61 | 0.45987 |
| lmo0640 | LMRG_00326 | Putative oxidoreductase SCO7655 | -2.59 | 0.45987 |
| lmo0903 | LMRG_02003 | OsmC/Ohr family protein | -2.59 | 0.45987 |
| lmo1258 | LMRG_00707 | Lysophospholipase (EC 3.1.1.5) | -2.57 | 0.45987 |
| lmo2491 | LMRG_01757 | Nucleotidase YfbR, HD superfamily | -2.57 | 0.45987 |
| lmo0053 | LMRG_02482 | LSU ribosomal protein L9p | -2.56 | 0.45987 |
| lmo0387 | LMRG_00080 | hypothetical protein | -2.56 | 0.45987 |
| lmo1553 | LMRG_01415 | Glutamate-1-semialdehyde 2,1-aminomutase (EC 5.4.3.8) | -2.56 | 0.45987 |
| lmo2509 | LMRG_01739 | Peptide chain release factor 2 @ programmed frameshift-containing | -2.55 | 0.45987 |
| lmo1745 | LMRG_02526 | Two-component transcriptional response regulator, LuxR family | -2.54 | 0.45987 |
| lmo2545 | LMRG_01702 | Homoserine kinase (EC 2.7.1.39) | -2.52 | 0.45987 |
| lmo0955 | LMRG_02054 | hypothetical protein | -2.51 | 0.45987 |
| lmo1612 | LMRG_01354 | Uncharacterized protein YtzB | -2.51 | 0.45987 |
| lmo1767 | LMRG_02504 | Phosphoribosylformylglycinamidine cyclo-ligase (EC 6.3.3.1) | -2.51 | 0.45987 |
| lmo0644 | LMRG_00331 | Lipoteichoic acid primase LtaP | -2.5 | 0.45987 |
| lmo0983 | LMRG_02083 | Glutathione peroxidase (EC 1.11.1.9) @ Thioredoxin peroxidase (EC 1.11.1.15) | -2.5 | 0.45987 |
| lmo1631 | LMRG_01335 | Anthranilate phosphoribosyltransferase (EC 2.4.2.18) | -2.5 | 0.45987 |
| lmo1929 | LMRG_01076 | Nucleoside diphosphate kinase (EC 2.7.4.6) | -2.49 | 0.45987 |
| lmo1048 | LMRG_00509 | Molybdenum cofactor biosynthesis protein MoaB | -2.48 | 0.45987 |
| lmo1450 | LMRG_00902 | DEAD-box ATP-dependent RNA helicase CshB (EC 3.6.4.13) | -2.48 | 0.45987 |
| lmo0104 | LMRG_02353 | hypothetical protein | -2.47 | 0.45987 |
| lmo2004 | LMRG_01152 | Transcriptional regulator, GntR family | -2.47 | 0.45987 |
| lmo1152 | LMRG_00595 | Propanediol utilization polyhedral body protein PduB | -2.46 | 0.45987 |
| lmo1427 | LMRG_00879 | Choline ABC transport system, permease protein OpuBB | -2.46 | 0.45987 |
| lmo1007 | LMRG_02107 | hypothetical protein | -2.45 | 0.45987 |
| lmo0600 | LMRG_00283 | hypothetical protein | -2.44 | 0.45987 |
| lmo2228 | LMRG_01604 | Negative regulatory protein YxlE | -2.42 | 0.45987 |
| lmo0964 | LMRG_02063 | FIG00002411: Thioredoxin-fold protein | -2.4 | 0.45987 |
| lmo0534 | LMRG_00216 | FIG00519347: Ribonucleotide reductase-like protein | -2.38 | 0.45987 |
| lmo1800 | LMRG_00947 | Protein tyrosine phosphatase (EC 3.1.3.48) | -2.38 | 0.45987 |
| lmo2564 | LMRG_02705 | 2-hydroxymuconate tautomerase-like protein YwhB (Bsu3751) | -2.38 | 0.45987 |
| lmo2449 | LMRG_01799 | 3'-to-5' exoribonuclease RNase R | -2.37 | 0.45987 |
| lmo0210 | LMRG_02632 | L-lactate dehydrogenase (EC 1.1.1.27) | -2.36 | 0.45987 |
| lmo2794 | LMRG_01903 | Chromosome (plasmid) partitioning protein ParB-2 | -2.36 | 0.45987 |
| lmo1847 | LMRG_00994 | Manganese ABC transporter, periplasmic-binding protein SitA | -2.35 | 0.45987 |
| lmo2535 | LMRG_01713 | ATP synthase F0 sector subunit a (EC 3.6.3.14) | -2.33 | 0.45987 |
| lmo0095 | LMRG_02344 | hypothetical protein | -2.31 | 0.45987 |
| lmo0616 | LMRG_00299 | membrane-anchoring domain / Glycerophosphoryl diester phosphodiesterase (EC 3.1.4.46) | -2.31 | 0.45987 |
| lmo0934 | LMRG_02033 | Epoxyqueuosine reductase (EC 1.17.99.6) QueG | -2.29 | 0.45987 |
| lmo0620 | LMRG_00303 | hypothetical protein | -2.28 | 0.45987 |
| lmo1759 | LMRG_02512 | ATP-dependent DNA helicase UvrD/PcrA (EC 3.6.4.12) | -2.25 | 0.45987 |
| lmo2559 | LMRG_02710 | CTP synthase (EC 6.3.4.2) | -2.25 | 0.45987 |
| lmo1983 | LMRG_01131 | Dihydroxy-acid dehydratase (EC 4.2.1.9) | -2.24 | 0.45987 |
| lmo2180 | LMRG_01652 | hypothetical protein | -2.23 | 0.45987 |
| lmo1154 | LMRG_00597 | Propanediol dehydratase medium subunit (EC 4.2.1.28) | -2.22 | 0.45987 |
| lmo1776 | LMRG_02495 | hypothetical protein | -2.19 | 0.45987 |
| lmo0177 | LMRG_02742 | Methionyl-tRNA synthetase (EC 6.1.1.10) | -2.18 | 0.45987 |
| lmo2388 | LMRG_02733 | hypothetical protein | -2.17 | 0.45987 |
| lmo2774 | LMRG_02899 | FIG00775122: hypothetical protein | -2.17 | 0.45987 |
| lmo1208 | LMRG_00654 | Cobyric acid synthase (EC 6.3.5.10) | -2.15 | 0.45987 |
| #N/A | LMRG_02591 | FIG00775102: hypothetical protein | -2.15 | 0.45987 |
| lmo1821 | LMRG_00968 | Protein serine/threonine phosphatase PrpC, regulation of stationary phase | -2.14 | 0.45987 |
| lmo2054 | LMRG_01204 | UPF0298 protein YlbG | -2.14 | 0.45987 |
| lmo2486 | LMRG_01762 | hypothetical protein | -2.14 | 0.45987 |
| lmo2213 | LMRG_01619 | Uncharacterized protein, homolog of B.subtilis yhgC | -2.13 | 0.45987 |
| lmo1739 | LMRG_02532 | ABC transporter, ATP-binding protein (cluster 3, basic aa/glutamine/opines) | -2.12 | 0.45987 |
| lmo2014 | LMRG_01162 | alpha-mannosidase (EC 3.2.1.24) | -2.12 | 0.45987 |
| lmo0121 | LMRG_02370 | Phage tail length tape-measure protein T | -2.1 | 0.45987 |
| lmo1075 | LMRG_00537 | Teichoic acid export ATP-binding protein TagH (EC 3.6.3.40) | -2.1 | 0.45987 |
| lmo0760 | LMRG_00448 | Carboxylesterase (EC 3.1.1.1) in BtlB locus | -2.09 | 0.45987 |
| lmo1814 | LMRG_00961 | Dihydroxyacetone kinase-like protein, phosphatase domain / Dihydroxyacetone kinase-like protein, kinase domain | -2.08 | 0.45987 |
| lmo1958 | LMRG_01105 | Ferrichrome transport system permease protein FhuB | -2.08 | 0.45987 |
| lmo2149 | LMRG_01683 | P26 | -2.08 | 0.45987 |
| lmo1942 | LMRG_01089 | ATP-dependent DNA helicase RecS (RecQ family) | -2.07 | 0.45987 |
| lmo0865 | LMRG_02288 | phosphoglucomutase/phosphomannomutase family protein | -2.06 | 0.45987 |
| lmo0869 | LMRG_02293 | 2-amino-3-carboxymuconate-6-semialdehyde decarboxylase (EC 4.1.1.45) | -2.04 | 0.45987 |
| lmo1031 | LMRG_00492 | Unknown pentose isomerase TM0951 | -2.04 | 0.45987 |
| lmo2474 | LMRG_01774 | RNase adapter protein RapZ | -2.04 | 0.45987 |
| lmo1262 | LMRG_00711 | Phage DNA binding protein | -2.03 | 0.45987 |
| lmo1025 | LMRG_02125 | hypothetical protein | -2.02 | 0.45987 |
| lmo2520 | LMRG_01728 | O-succinylbenzoate synthase (EC 4.2.1.113) | -2.02 | 0.45987 |
| lmo0767 | LMRG_00455 | ABC transporter, permease protein 2 (cluster 1, maltose/g3p/polyamine/iron) | -2.01 | 0.45987 |
| lmo0369 | LMRG_00061 | Transcriptional regulatory protein YeeN | -1.99 | 0.45987 |
| lmo0590 | LMRG_00272 | Dihydroxyacetone kinase-like protein, phosphatase domain / Dihydroxyacetone kinase-like protein, kinase domain | -1.98 | 0.45987 |
| lmo0859 | LMRG_02282 | Multiple sugar ABC transporter, substrate-binding protein MsmE | -1.98 | 0.45987 |
| lmo1675 | LMRG_01292 | 2-succinyl-5-enolpyruvyl-6-hydroxy-3-cyclohexene-1-carboxylic-acid synthase (EC 2.2.1.9) | -1.98 | 0.45987 |
| lmo2048 | LMRG_01198 | FIG01269488: protein, clustered with ribosomal protein L32p | -1.98 | 0.45987 |
| lmo0715 | LMRG_00404 | Flagellar assembly protein FliH | -1.97 | 0.45987 |
| lmo1047 | LMRG_00508 | GTP 3',8-cyclase (EC 4.1.99.22) | -1.97 | 0.45987 |
| lmo1924 | LMRG_01071 | Prephenate dehydrogenase (EC 1.3.1.12) | -1.96 | 0.45987 |
| lmo2523 | LMRG_01725 | Single-stranded DNA-binding protein | -1.94 | 0.45987 |
| lmo1466 | LMRG_00918 | Membrane protein containing HD superfamily hydrolase domain, YQFF ortholog | -1.93 | 0.45987 |
| lmo2162 | LMRG_01670 | Inosose dehydratase (EC 4.2.1.44) | -1.91 | 0.45987 |
| lmo0553 | LMRG_00235 | CBS domain protein | -1.9 | 0.45987 |
| lmo0489 | LMRG_00170 | 2,4-dienoyl-CoA reductase [NADPH] (EC 1.3.1.34) | -1.88 | 0.45987 |
| lmo0783 | LMRG_02869 | PTS system, mannose-specific IIA component (EC 2.7.1.191) / PTS system, mannose-specific IIB component (EC 2.7.1.191) | -1.87 | 0.45987 |
| #N/A | LMRG_00561 | hypothetical protein | -1.87 | 0.45987 |
| lmo0232 | LMRG_02674 | ATP-dependent Clp protease, ATP-binding subunit ClpC | -1.86 | 0.45987 |
| lmo0314 | LMRG_00007 | Cellobiose phosphotransferase system YdjC-like protein | -1.86 | 0.45987 |
| lmo2498 | LMRG_01750 | Phosphate ABC transporter, permease protein PstC (TC 3.A.1.7.1) | -1.85 | 0.45987 |
| lmo2339 | LMRG_01504 | Uncharacterized protein YbbK | -1.84 | 0.45987 |
| lmo1703 | LMRG_02777 | Uncharacterized RNA methyltransferase YfjO | -1.83 | 0.45987 |
| lmo2076 | LMRG_01227 | Ribosomal-protein-S18p-alanine acetyltransferase (EC 2.3.1.128) | -1.83 | 0.45987 |
| lmo0033 | LMRG_02462 | glycosyl hydrolase, family 9 | -1.8 | 0.45987 |
| lmo2419 | LMRG_01829 | Methionine ABC transporter ATP-binding protein | -1.8 | 0.45987 |
| lmo2279 | LMRG_01553 | holin | -1.79 | 0.45987 |
| lmo1661 | LMRG_01306 | [4Fe-4S]-AdoMet protein YtqA | -1.77 | 0.45987 |
| lmo1497 | LMRG_01473 | Uridine kinase (EC 2.7.1.48) | -1.75 | 0.45987 |
| lmo2372 | LMRG_02717 | ABC transporter, ATP-binding protein | -1.75 | 0.45987 |
| #N/A | LMRG_02593 | Lin0308 protein | -1.75 | 0.45987 |
| lmo1243 | LMRG_00689 | PhnB protein | -1.74 | 0.45987 |
| lmo1673 | LMRG_01294 | Naphthoate synthase (EC 4.1.3.36) | -1.74 | 0.45987 |
| lmo0929 | LMRG_02028 | Sortase A, LPXTG specific | -1.73 | 0.45987 |
| lmo2837 | LMRG_01861 | Inner membrane ABC transporter permease protein YcjP | -1.72 | 0.45987 |
| lmo0279 | LMRG_02596 | Ribonucleotide reductase of class III (anaerobic), large subunit (EC 1.17.4.2) | -1.71 | 0.45987 |
| lmo0478 | LMRG_02885 | putative secreted protein | -1.71 | 0.45987 |
| lmot19 | LMRG_05019 | tRNA-Ser-GCT | -1.71 | 0.45987 |
| lmo0154 | LMRG_02399 | Zinc ABC transporter, ATP-binding protein AdcC | -1.66 | 0.45987 |
| lmo0338 | LMRG_02932 | FIG00774315: hypothetical protein | -1.66 | 0.45987 |
| lmo0766 | LMRG_00454 | ABC transporter, permease protein 1 (cluster 1, maltose/g3p/polyamine/iron) | -1.66 | 0.45987 |
| lmo1232 | LMRG_00678 | Recombination inhibitory protein MutS2 | -1.66 | 0.45987 |
| lmo0687 | LMRG_00375 | hypothetical protein | -1.65 | 0.45987 |
| lmo1191 | LMRG_00637 | Cobyrinic acid a,c-diamide synthetase (EC 6.3.5.11) | -1.65 | 0.45987 |
| lmo2717 | LMRG_01979 | Cytochrome d ubiquinol oxidase subunit II (EC 1.10.3.-) | -1.65 | 0.45987 |
| lmo1384 | LMRG_00836 | Rhodanese domain protein UPF0176, Firmicutes subgroup | -1.64 | 0.45987 |
| lmo2090 | LMRG_01241 | Argininosuccinate synthase (EC 6.3.4.5) | -1.64 | 0.45987 |
| lmo2542 | LMRG_01705 | Peptide chain release factor N(5)-glutamine methyltransferase (EC 2.1.1.297) | -1.63 | 0.45987 |
| lmo1412 | LMRG_00864 | DNA topology modulation protein FlaR | -1.61 | 0.45987 |
| lmo0822 | LMRG_02247 | Transcriptional regulator, MerR family | -1.6 | 0.45987 |
| lmo1141 | LMRG_00584 | Precorrin-2 oxidase (EC 1.3.1.76) | -1.59 | 0.45987 |
| lmo1736 | LMRG_02535 | Acetyltransferase | -1.58 | 0.45987 |
| lmo2476 | LMRG_01772 | Aldose 1-epimerase (EC 5.1.3.3) | -1.58 | 0.45987 |
| lmo0340 | LMRG_00031 | hypothetical protein | -1.57 | 0.45987 |
| lmo0551 | LMRG_00233 | Extracellular protein | -1.56 | 0.45987 |
| lmo0721 | LMRG_00410 | elongation factor G-binding protein, putative | -1.56 | 0.45987 |
| lmo2123 | LMRG_01277 | Maltodextrin ABC transporter, permease protein MdxG | -1.56 | 0.45987 |
| lmo1439 | LMRG_00891 | Superoxide dismutase [Mn] (EC 1.15.1.1) | -1.55 | 0.45987 |
| lmo1606 | LMRG_01360 | DNA translocase FtsK | -1.54 | 0.45987 |
| lmo2244 | LMRG_01588 | Similar to ribosomal large subunit pseudouridine synthase D, Bacillus subtilis YhcT type | -1.53 | 0.45987 |
| lmo2140 | LMRG_02800 | ABC transporter, permease protein | -1.51 | 0.45987 |
| lmo1916 | LMRG_01063 | Beta-lactamase class C-like and penicillin binding proteins (PBPs) superfamily | -1.5 | 0.45987 |
| lmo2084 | LMRG_01235 | putative phosphotransferase | -1.49 | 0.45987 |
| lmo2307 | LMRG_01526 | Phage DNA replication protein O | -1.49 | 0.45987 |
| lmo0243 | LMRG_02663 | RNA polymerase sporulation specific sigma factor SigH | -1.47 | 0.45987 |
| lmo2804 | LMRG_01892 | hypothetical protein | -1.45 | 0.45987 |
| lmo2534 | LMRG_01714 | ATP synthase F0 sector subunit c (EC 3.6.3.14) | -1.43 | 0.45987 |
| lmo1953 | LMRG_01100 | Purine nucleoside phosphorylase (EC 2.4.2.1) @ N-Ribosylnicotinamide phosphorylase (EC 2.4.2.1) | -1.42 | 0.45987 |
| lmo0379 | LMRG_00071 | hypothetical protein | -1.41 | 0.45987 |
| lmo1470 | LMRG_00923 | 16S rRNA (uracil(1498)-N(3))-methyltransferase (EC 2.1.1.193) | -1.39 | 0.45987 |
| lmo0630 | LMRG_00313 | Putative transcriptional antiterminator, BglG family / PTS system, IIA component | -1.37 | 0.45987 |
| lmo2850 | LMRG_02421 | Predicted L-rhamnose permease RhaY | -1.37 | 0.45987 |
| lmo0061 | LMRG_02491 | FtsK/SpoIIIE family protein, putative EssC/YukB component of Type VII secretion system | -1.35 | 0.45987 |
| lmo1377 | LMRG_00829 | Two-component transcriptional response regulator, OmpR family | -1.35 | 0.45987 |
| lmo0712 | LMRG_00401 | Flagellar hook-basal body complex protein FliE | -1.34 | 0.45987 |
| lmo1126 | LMRG_00568 | Acetyltransferase, GNAT family | -1.33 | 0.45987 |
| lmo1137 | LMRG_00580 | hypothetical protein | -1.31 | 0.45987 |
| lmo1214 | LMRG_00660 | hypothetical protein | -1.31 | 0.45987 |
| lmo1614 | LMRG_01352 | Quorum-quenching lactonase YtnP | -1.3 | 0.45987 |
| lmo2392 | LMRG_02737 | DUF1450 superfamily protein | -1.3 | 0.45987 |
| lmo0546 | LMRG_00228 | Sorbitol-6-phosphate 2-dehydrogenase (EC 1.1.1.140) | -1.29 | 0.45987 |
| lmo1676 | LMRG_01291 | Isochorismate synthase (EC 5.4.4.2) @ Menaquinone-specific isochorismate synthase (EC 5.4.4.2) | -1.28 | 0.45987 |
| lmo2740 | LMRG_01956 | hypothetical protein | -1.28 | 0.45987 |
| lmo1218 | LMRG_00664 | Uncharacterized RNA methyltransferase YsgA | -1.27 | 0.45987 |
| lmo2104a | LMRG_01259 | hypothetical protein | -1.27 | 0.45987 |
| lmo0181 | LMRG_02746 | N-acetyl-D-glucosamine ABC transporter, substrate-binding protein | -1.26 | 0.45987 |
| lmo2685 | LMRG_02230 | PTS system, beta-glucoside-specific IIA component | -1.26 | 0.45987 |
| lmo1692 | LMRG_02766 | hypothetical protein | -1.25 | 0.45987 |
| lmo0126 | LMRG_02375 | hypothetical protein | -1.23 | 0.45987 |
| lmo1695 | LMRG_02769 | L-O-lysylphosphatidylglycerol synthase (EC 2.3.2.3) | -1.22 | 0.45987 |
| lmo2429 | LMRG_01819 | Ferrichrome transport ATP-binding protein FhuC | -1.22 | 0.45987 |
| lmo2150 | LMRG_01682 | hypothetical protein | -1.21 | 0.45987 |
| lmo0568 | LMRG_00250 | ATP phosphoribosyltransferase (EC 2.4.2.17) => HisGs | -1.17 | 0.45987 |
| lmo0682 | LMRG_00370 | Flagellar synthesis regulator FleN | -1.16 | 0.45987 |
| lmo1063 | LMRG_00525 | ABC-type antimicrobial peptide transport system, ATPase component | -1.11 | 0.45987 |
| lmo0958 | LMRG_02057 | Predicted transcriptional regulator of N-Acetylglucosamine utilization, GntR family | -1.05 | 0.45987 |
| lmo0007 | LMRG_02435 | DNA gyrase subunit A (EC 5.99.1.3) | -1 | 0.45987 |
| lmo0301 | LMRG_02567 | PTS system, beta-glucoside-specific, IIA component | -0.98 | 0.45987 |
| lmo1857 | LMRG_01004 | UPF0346 protein YozE | -0.93 | 0.45987 |
| lmo2299 | LMRG_01533 | putative portal protein | -0.89 | 0.45987 |
| lmo1955 | LMRG_01102 | Site-specific tyrosine recombinase XerD | -0.72 | 0.45987 |
| lmo2347 | LMRG_01496 | L-cystine ABC transporter, permease protein TcyM | -0.66 | 0.45987 |
| lmo1691 | LMRG_02765 | Deoxyuridine 5'-triphosphate nucleotidohydrolase (EC 3.6.1.23) | -0.62 | 0.45987 |
| lmo2024 | LMRG_01173 | Quinolinate phosphoribosyltransferase [decarboxylating] (EC 2.4.2.19) | -0.56 | 0.45987 |
| lmo1274 | LMRG_00724 | Rossmann fold nucleotide-binding protein Smf possibly involved in DNA uptake | -0.52 | 0.45987 |
| lmo2184 | LMRG_01648 | Heme transporter IsdDEF, lipoprotein IsdE | -0.47 | 0.45987 |
| lmo2521 | LMRG_01727 | N-acetylmannosaminyltransferase (EC 2.4.1.187) | -0.47 | 0.45987 |
| lmo1484 | LMRG_00937 | Late competence protein ComEA, DNA receptor | -0.44 | 0.45987 |
| lmo2348 | LMRG_01495 | L-cystine ABC transporter, permease protein TcyL | -0.44 | 0.45987 |
| lmo1542 | LMRG_01428 | LSU ribosomal protein L21p | -0.42 | 0.45987 |
| lmo1672 | LMRG_01295 | O-succinylbenzoic acid--CoA ligase (EC 6.2.1.26) | -0.42 | 0.45987 |
| lmo1338 | LMRG_00788 | Uncharacterized protein YqgQ | -0.41 | 0.45987 |
| lmo1334 | LMRG_00784 | Uncharacterized membrane anchored protein YqzD | -0.37 | 0.45987 |
| lmo2366 | LMRG_02711 | Transcriptional regulator, DeoR family | -0.36 | 0.45987 |
| lmo0288 | LMRG_02583 | Two-component sensor kinase SA14-24 | -0.33 | 0.45987 |
| lmo1838 | LMRG_00985 | Aspartate carbamoyltransferase (EC 2.1.3.2) | -0.33 | 0.45987 |
| lmo0969 | LMRG_02068 | Similar to ribosomal large subunit pseudouridine synthase D, Bacillus subtilis YjbO type | -0.29 | 0.45987 |
| lmo0079 | LMRG_02389 | hypothetical protein | -0.18 | 0.45987 |
| lmo1872 | LMRG_01019 | 23S rRNA (guanine(748)-N(1))-methyltransferase (EC 2.1.1.188) | -0.17 | 0.45987 |
| lmo1928 | LMRG_01075 | Chorismate synthase (EC 4.2.3.5) | -0.16 | 0.45987 |
| lmo1819 | LMRG_00966 | Ribosome small subunit biogenesis RbfA-release protein RsgA | -0.15 | 0.45987 |
| lmo1331 | LMRG_00781 | Polyribonucleotide nucleotidyltransferase (EC 2.7.7.8) | -0.14 | 0.45987 |
| lmo1923 | LMRG_01070 | 3-phosphoshikimate 1-carboxyvinyltransferase (EC 2.5.1.19) | -0.14 | 0.45987 |
| lmo1978 | LMRG_02980 | Glucose-6-phosphate 1-dehydrogenase (EC 1.1.1.49) | -0.11 | 0.45987 |
| lmo2704 | LMRG_01993 | DNA polymerase III subunits gamma and tau (EC 2.7.7.7) | -0.11 | 0.45987 |
| lmo2612 | LMRG_02156 | Protein translocase subunit SecY | -0.09 | 0.45987 |
| lmo2801 | LMRG_01896 | N-acetylmannosamine-6-phosphate 2-epimerase (EC 5.1.3.9) | -2.05 | 0.46103 |
| lmo1285 | LMRG_00735 | Uncharacterized CoA-binding protein YneT | -2 | 0.46103 |
| lmo2065 | LMRG_01215 | FIG007350: hypothetical protein co-occurring with bile hydrolase | -1.84 | 0.46103 |
| lmo2594 | LMRG_02134 | hypothetical protein | -1.69 | 0.46103 |
| lmo2031 | LMRG_01180 | Pyridoxal phosphate-containing protein YggS | -1.64 | 0.46103 |
| lmo2423 | LMRG_01825 | Cobalt-zinc-cadmium resistance protein | -1.6 | 0.46103 |
| lmo2846 | LMRG_02417 | L-rhamnose mutarotase (EC 5.1.3.32) | -1.46 | 0.46103 |
| lmo1679 | LMRG_01288 | Cystathionine beta-lyase (EC 4.4.1.8) | -1.38 | 0.46103 |
| lmo0834 | LMRG_RS04175 | hypothetical protein | -1.36 | 0.46103 |
| lmo0674 | LMRG_00362 | hypothetical protein | -1.31 | 0.46103 |
| lmo0476 | LMRG_00157 | dNTP triphosphohydrolase, broad substrate specificity | -1.15 | 0.46103 |
| lmo0882 | LMRG_02306 | transmembrane protein, distant homology with ydbS | -2.69 | 0.46135 |
| lmo0558 | LMRG_00240 | 6-phosphogluconolactonase (EC 3.1.1.31) | -2.18 | 0.46135 |
| lmo0685 | LMRG_00373 | Flagellar motor rotation protein MotA | -2.15 | 0.46135 |
| lmo2473 | LMRG_01775 | FIG002813: LPPG:FO 2-phospho-L-lactate transferase like, CofD-like | -2.02 | 0.46135 |
| lmo1870 | LMRG_01017 | hypothetical protein | -2.01 | 0.46135 |
| lmo1349 | LMRG_00799 | Glycine dehydrogenase [decarboxylating] (glycine cleavage system P1 protein) (EC 1.4.4.2) | -1.95 | 0.46135 |
| lmo2216 | LMRG_01616 | Histidine triad (HIT) nucleotide-binding protein, similarity with At5g48545 and yeast YDL125C (HNT1) | -1.85 | 0.46135 |
| lmo1489 | LMRG_00942 | RNA-binding protein YhbY | -1.83 | 0.46135 |
| lmo1662 | LMRG_01305 | Putative rRNA methylase YtqB | -1.32 | 0.46135 |
| lmot39 | LMRG_05039 | tRNA-Arg-CCT | -1.23 | 0.46135 |
| lmo1837 | LMRG_00984 | Dihydroorotase (EC 3.5.2.3) | -1.16 | 0.46135 |
| lmo2250 | LMRG_01581 | ABC transporter, substrate-binding protein (cluster 3, basic aa/glutamine/opines) / ABC transporter, permease protein (cluster 3, basic aa/glutamine/opines) | -1.21 | 0.46274 |
| lmo2289 | LMRG_01543 | Protein gp14 | -2.01 | 0.46546 |
| lmo0677 | LMRG_00365 | Flagellar biosynthesis protein FliQ | -1.88 | 0.46546 |
| lmo0494 | LMRG_00175 | lipase/acylhydrolase family protein | -1.55 | 0.46546 |
| lmo0843 | LMRG_02266 | Uncharacterized membrane protein YsdA | -1.2 | 0.46546 |
| lmo2387 | LMRG_02732 | hypothetical protein | -1.59 | 0.46603 |
| lmo1620 | LMRG_01346 | Putative dipeptidase YtjP | -1.6 | 0.46822 |
| lmo1830 | LMRG_00977 | Short-chain dehydrogenase | -2.01 | 0.46855 |
| lmo0463 | LMRG_00152 | FIG00775752: hypothetical protein | -1.69 | 0.46855 |
| lmo1784 | LMRG_02828 | LSU ribosomal protein L35p | -2.48 | 0.46944 |
| lmo1685 | LMRG_02759 | Glutamate-1-semialdehyde 2,1-aminomutase (EC 5.4.3.8) | -1.75 | 0.46944 |
| lmo0926 | LMRG_02025 | Transcriptional regulator, AcrR family | -1.52 | 0.46944 |
| lmo1557 | LMRG_02940 | Glutamyl-tRNA reductase (EC 1.2.1.70) | -0.52 | 0.47 |
| lmo2519 | LMRG_01729 | Undecaprenyl-phosphate alpha-N-acetylglucosaminyl 1-phosphate transferase (EC 2.7.8.33) | -1.17 | 0.47016 |
| lmo1167 | LMRG_00610 | Glycerol uptake facilitator protein @ Propanediol diffusion facilitator | -2.3 | 0.47024 |
| lmo0894 | LMRG_02318 | Serine-protein kinase RsbW (EC 2.7.11.1) | -2.15 | 0.47024 |
| lmo1534 | LMRG_01436 | L-lactate dehydrogenase (EC 1.1.1.27) | -1.7 | 0.47024 |
| lmo0091 | LMRG_02340 | ATP synthase gamma chain (EC 3.6.3.14) | -1.83 | 0.47413 |
| lmo0820 | LMRG_02245 | Acetyltransferase (GNAT family) SAS0976 | -1.37 | 0.47413 |
| lmo2572 | LMRG_02696 | Dihydrofolate reductase homolog | -1.49 | 0.47587 |
| lmo1366 | LMRG_00816 | RNA binding methyltransferase FtsJ like | -1.85 | 0.48117 |
| lmo2354 | LMRG_01489 | Exoenzymes regulatory protein AepA precursor | -1.99 | 0.48329 |
| lmo2125 | LMRG_01279 | Maltodextrin ABC transporter, substrate-binding protein MdxE | -1.49 | 0.48489 |
| lmo2585 | LMRG_02682 | Hypothetical protein YrhD | -1.49 | 0.48489 |
| lmo1170 | LMRG_00613 | L-threonine kinase in B12 biosynthesis (EC 2.7.1.177) | -1.16 | 0.48489 |
| lmo0821 | LMRG_02246 | hypothetical protein | -1.73 | 0.49263 |
| lmo2085 | LMRG_01236 | hypothetical protein | -1.52 | 0.49263 |
| lmo0800 | LMRG_00489 | Uncharacterized protein YqkB | -1.61 | 0.49864 |
| lmo2446 | LMRG_01802 | glycosyl hydrolase, family 31 | -1.34 | 0.49876 |
| lmo2371 | LMRG_02716 | ABC transporter, permease protein | -1.27 | 0.49888 |
| lmo2848 | LMRG_02419 | L-rhamnose isomerase (EC 5.3.1.14) | -1.58 | 0.50251 |
| lmo2838 | LMRG_01860 | Inner membrane ABC transporter permease protein YcjO | -1.73 | 0.50301 |
| lmo1230 | LMRG_00676 | Possible colicin V production protein | -1.4 | 0.50624 |
| lmo1883 | LMRG_01030 | Chitinase (EC 3.2.1.14) | -1.62 | 0.50869 |
| lmo0636 | LMRG_00319 | Rrf2 family transcriptional regulator | -1.25 | 0.51811 |
| lmo0979 | LMRG_02079 | Efflux ABC transporter, ATP-binding protein | -1.84 | 0.51839 |
| lmo0241 | LMRG_02665 | 23S rRNA (guanosine(2251)-2'-O)-methyltransferase (EC 2.1.1.185) | -1.53 | 0.51839 |
| lmo2568 | LMRG_02700 | hypothetical protein | -1.24 | 0.51839 |
| lmo0187 | LMRG_02752 | Ribonuclease M5 (EC 3.1.26.8) | -1.72 | 0.52582 |
| lmo1394 | LMRG_00846 | Oxidoreductase, short-chain dehydrogenase/reductase family | -1.52 | 0.52668 |
| lmo0236 | LMRG_02670 | 2-C-methyl-D-erythritol 2,4-cyclodiphosphate synthase (EC 4.6.1.12) | -1.42 | 0.53215 |
| lmo0949 | LMRG_02048 | UPF0118 membrane protein YdbI | -1.36 | 0.54993 |
| lmo2178 | LMRG_01654 | Putative peptidoglycan bound protein (LPXTG motif) Lmo2178 homolog | -1.23 | 0.55152 |
| lmo0621 | LMRG_00304 | hypothetical protein | -1.62 | 0.55157 |
| lmo2851 | LMRG_02422 | Transcriptional regulator of rhamnose utilization, AraC family | -1.63 | 0.55353 |
| lmo1083 | LMRG_00545 | dTDP-glucose 4,6-dehydratase (EC 4.2.1.46) | -1.35 | 0.55511 |
| lmo2164 | LMRG_01668 | Transcriptional regulator, AraC family | -1.65 | 0.5651 |
| lmo0429 | LMRG_00121 | glycosyl hydrolase, family 38 | -1.24 | 0.57085 |
| lmo2482 | LMRG_01766 | Prolipoprotein diacylglyceryl transferase | -1.47 | 0.57774 |
| lmo1428 | LMRG_00880 | Choline ABC transport system, ATP-binding protein OpuBA | -1.21 | 0.57966 |
| #N/A | LMRG_02851 | Internalin H (LPXTG motif) | -1.1 | 0.58233 |
| lmo0717 | LMRG_00406 | Soluble lytic murein transglycosylase (EC 4.2.2.n1) | -1.54 | 0.59032 |
| lmo0227 | LMRG_02837 | tRNA-dihydrouridine synthase DusB | -1.29 | 0.62446 |
| lmo0493 | LMRG_00174 | hydrolase, CocE/NonD family | -1.16 | 0.62858 |
| lmo2741 | LMRG_01955 | Multidrug-efflux transporter, major facilitator superfamily (MFS) | -1.39 | 0.65275 |
| lmo1368 | LMRG_00818 | DNA repair protein RecN | -1.31 | 0.68404 |
| lmo2677 | LMRG_02222 | Hydrolase, alpha/beta fold family | -1.49 | 0.6892 |
| lmo0543 | LMRG_00225 | PTS system, glucitol/sorbitol-specific IIB component (EC 2.7.1.198) / PTS system, glucitol/sorbitol-specific IIC component 2 | -2.13 | 0.69209 |
| lmo2293 | LMRG_01539 | Protein gp10 [Bacteriophage A118] | -1.58 | 0.69807 |
| lmo1148 | LMRG_00591 | Cobalamin synthase (EC 2.7.8.26) | -1.49 | 0.69807 |
| lmo0754 | LMRG_00442 | Bile acid 7-alpha dehydratase BaiE (EC 4.2.1.106) | -1.38 | 0.69807 |
| lmo0508 | LMRG_00189 | PTS system, galactitol-specific IIC component | -1.55 | 0.69817 |
| lmo0528 | LMRG_02879 | putative secreted protein | -1.38 | 0.69817 |
| lmo0791 | LMRG_00479 | hypothetical protein | -1.4 | 0.70065 |
| lmo0134 | LMRG_02383 | Uncharacterized protein YjdJ, possible GNAT acetyltransferase | -2.95 | 0.7009 |
| lmo2351 | LMRG_01492 | FMN reductase (NADPH) (EC 1.5.1.38) | -2.72 | 0.7009 |
| lmo0907 | LMRG_02007 | Phosphoglycerate mutase family, Lmo0907 homolog | -2.33 | 0.7009 |
| lmo1972 | LMRG_01119 | PTS system, IIB component | -1.81 | 0.7009 |
| lmo0474 | LMRG_00155 | hypothetical protein | -1.67 | 0.7009 |
| lmo0412 | LMRG_00104 | hypothetical protein | -1.48 | 0.7009 |
| lmo0950 | LMRG_02049 | Cell surface hydrolase, membrane-bound | -1.42 | 0.7009 |
| lmo2575 | LMRG_02693 | Cobalt/zinc/cadmium resistance protein CzcD | -1.39 | 0.7009 |
| lmo0965 | LMRG_02064 | Adenylate cyclase (EC 4.6.1.1) | -1.33 | 0.7009 |
| lmo0651 | LMRG_00338 | Transcriptional regulator, GntR family | -1.27 | 0.7009 |
| lmo0109 | LMRG_02358 | Transcriptional regulator, AraC family | -1.21 | 0.7009 |
| lmo1991 | LMRG_01139 | Threonine dehydratase biosynthetic (EC 4.3.1.19) | -1.18 | 0.7009 |
| lmo1310 | LMRG_00760 | Co-activator of prophage gene expression IbrA | -1.15 | 0.7009 |
| lmo2306 | LMRG_01527 | Protein gp32 [Listeria phage 2389] | -0.9 | 0.7009 |
| lmo2049 | LMRG_01199 | UPF0348 protein family | -1.46 | 0.70113 |
| lmo1908 | LMRG_01055 | MazG nucleotide pyrophosphohydrolase | -2.23 | 0.70422 |
| lmo2044 | LMRG_01193 | Oligopeptide ABC transporter, substrate-binding protein OppA (TC 3.A.1.5.1) | -1.61 | 0.70422 |
| lmo0432 | LMRG_00124 | Oxidoreductase, short-chain dehydrogenase/reductase family | -1.58 | 0.70422 |
| lmo0058 | LMRG_02488 | Putative secretion system component EssA | -1.49 | 0.70422 |
| lmo0727 | LMRG_00415 | Glutamine--fructose-6-phosphate aminotransferase [isomerizing] (EC 2.6.1.16) | -2.56 | 0.70474 |
| lmo0567 | LMRG_00249 | Histidinol dehydrogenase (EC 1.1.1.23) | -2.4 | 0.70474 |
| lmo0122 | LMRG_02371 | Phage tail fiber | -1.6 | 0.70474 |
| lmo0016 | LMRG_02445 | Cytochrome aa3-600 menaquinol oxidase subunit IV | -1.26 | 0.70474 |
| lmo0723 | LMRG_00412 | Methyl-accepting chemotaxis sensor/transducer protein | -1.34 | 0.70533 |
| lmo0390 | LMRG_00083 | Uracil-DNA glycosylase, family 1 (EC 3.2.2.27) | -1.78 | 0.70685 |
| lmo1790 | LMRG_02814 | Uncharacterized protein YobT | -1.32 | 0.70685 |
| lmo0730 | LMRG_00418 | hypothetical protein | -1.82 | 0.708 |
| lmo0795 | LMRG_00483 | Uncharacterized inner membrane protein RarD | -1.28 | 0.708 |
| lmo0862 | LMRG_02285 | Trehalose-6-phosphate hydrolase (EC 3.2.1.93) | -1.28 | 0.71168 |
| lmo1457 | LMRG_00909 | Phosphoenolpyruvate synthase regulatory protein | -1.19 | 0.71168 |
| lmo1593 | LMRG_01374 | Cysteine desulfurase (EC 2.8.1.7), associated with tRNA 4-thiouridine synthase | -1.98 | 0.71317 |
| lmo1462 | LMRG_00914 | GTP-binding protein Era | -1.22 | 0.71317 |
| lmo2830 | LMRG_01868 | Thioredoxin | -1.71 | 0.71753 |
| lmo0483 | LMRG_00164 | Transcriptional regulator, AraC family | -1.5 | 0.71753 |
| lmo1042 | LMRG_00503 | Molybdopterin molybdenumtransferase (EC 2.10.1.1) | -1.47 | 0.72203 |
| lmo0860 | LMRG_02283 | Multiple sugar ABC transporter, permease protein MsmF | -1.18 | 0.77494 |
| lmo2634 | LMRG_02179 | Transmembrane component of general energizing module of ECF transporters | -1.12 | 0.77494 |
| lmo1079 | LMRG_00541 | FIG028455: hypothetical protein | 5.15 | 0.81112 |
| lmo1688 | LMRG_02762 | Enoyl-[acyl-carrier-protein] reductase [NADPH] (EC 1.3.1.104), FabL | -0.31 | 0.96019 |
| lmo0690 | LMRG_00378 | Flagellin protein FlaA | -1.1 | 0.96721 |
| lmo0637 | LMRG_00320 | SAM-dependent methyltransferase | -1.29 | 0.98418 |
| lmo0047 | LMRG_02476 | putative lipoprotein | -1.15 | 1 |
| lmo2404 | LMRG_01844 | UPF0721 transmembrane protein YunE | -1.15 | 1 |
| lmo0538 | LMRG_00220 | N-acyl-L-amino acid amidohydrolase (EC 3.5.1.14) | -1.13 | 1 |
| lmo0763 | LMRG_00451 | Ser/Thr protein phosphatase family protein | -1.13 | 1 |
| lmo0921 | LMRG_02021 | UPF0703 protein YcgQ | -1.13 | 1 |
| lmo0945 | LMRG_02949 | Metallo-beta-lactamase superfamily | -1.13 | 1 |
| lmo1013 | LMRG_02113 | Potassium efflux system KefA protein / Small-conductance mechanosensitive channel | -1.13 | 1 |
| lmo0756 | LMRG_00444 | ABC transporter, ATP-binding protein in BtlB locus | -1.12 | 1 |
| lmo1260 | LMRG_00709 | Glutamate 5-kinase (EC 2.7.2.11) | -1.12 | 1 |
| lmo0519 | LMRG_00200 | Uncharacterized MFS-type transporter | -1.11 | 1 |
| lmo1175 | LMRG_00621 | Ethanolamine ammonia-lyase heavy chain (EC 4.3.1.7) | -1.11 | 1 |
| lmo1988 | LMRG_01136 | 3-isopropylmalate dehydrogenase (EC 1.1.1.85) | -1.11 | 1 |
| lmo2095 | LMRG_01246 | Tagatose-6-phosphate kinase (EC 2.7.1.144) / 1-phosphofructokinase (EC 2.7.1.56) | -1.11 | 1 |
| lmo2235 | LMRG_01597 | 2,4-dienoyl-CoA reductase [NADPH] (EC 1.3.1.34) | -1.1 | 1 |
| lmo2549 | LMRG_01698 | cell wall teichoic acid glycosylation protein GtcA | -1.1 | 1 |
| lmo2811 | LMRG_01887 | tRNA-5-carboxymethylaminomethyl-2-thiouridine(34) synthesis protein MnmE | -1.1 | 1 |
| lmo1036 | LMRG_02973 | hypothetical protein | -1.09 | 1 |
| lmo1524 | LMRG_01446 | Adenine phosphoribosyltransferase (EC 2.4.2.7) | -1.09 | 1 |
| lmo2058 | LMRG_01208 | Heme A synthase, cytochrome oxidase biogenesis protein Cox15-CtaA | -1.09 | 1 |
| lmo1001 | LMRG_02101 | hypothetical protein | -1.08 | 1 |
| lmo1165 | LMRG_00608 | CoA-acylating propionaldehyde dehydrogenase | -1.08 | 1 |
| lmo1725 | LMRG_02546 | Transcriptional regulator, GntR family | -1.08 | 1 |
| lmo2254 | LMRG_01577 | Guanine/hypoxanthine permease PbuO | -1.08 | 1 |
| lmo0770 | LMRG_00458 | Transcriptional regulator, GntR family | -1.07 | 1 |
| lmo0957 | LMRG_02056 | Glucosamine-6-phosphate deaminase (EC 3.5.99.6) | -1.07 | 1 |
| lmo1984 | LMRG_01132 | Acetolactate synthase large subunit (EC 2.2.1.6) | -1.07 | 1 |
| lmo2737 | LMRG_01959 | Bacillus subtilis catabolite repression transcription factor CcpB | -1.07 | 1 |
| lmo0017 | LMRG_02446 | Capsule biosynthesis protein capA | -1.06 | 1 |
| lmo0806 | LMRG_02791 | DNA-binding protein | -1.06 | 1 |
| lmo1125 | LMRG_00567 | hypothetical protein | -1.06 | 1 |
| lmo1369 | LMRG_00819 | Branched-chain phosphotransacylase (EC 2.3.1.- ) | -1.05 | 1 |
| lmo1711 | LMRG_02558 | Aminopeptidase S (Leu, Val, Phe, Tyr preference) (EC 3.4.11.24) | -1.05 | 1 |
| lmo0575 | LMRG_00257 | Glucomannan utilization operon transcriptional regulator, GmuR | -1.04 | 1 |
| lmo1525 | LMRG_01445 | Single-stranded-DNA-specific exonuclease RecJ | -1.04 | 1 |
| lmo2176 | LMRG_01656 | Transcriptional regulator, AcrR family | -1.04 | 1 |
| lmo2450 | LMRG_01798 | Carboxylesterase (EC 3.1.1.1) | -1.04 | 1 |
| lmo0346 | LMRG_00037 | Triosephosphate isomerase (EC 5.3.1.1) | -1.03 | 1 |
| lmo0355 | LMRG_00046 | redox proteins related to the succinate dehydrogenases and fumarate reductases | -1.03 | 1 |
| lmo0521 | LMRG_00202 | 6-phospho-beta-glucosidase (EC 3.2.1.86) | -1.03 | 1 |
| lmo1509 | LMRG_01461 | RecD-like DNA helicase YrrC | -1.03 | 1 |
| lmo1993 | LMRG_01141 | Pyrimidine-nucleoside phosphorylase (EC 2.4.2.2) | -1.03 | 1 |
| lmo0256 | LMRG_02653 | 16S rRNA (guanine(1207)-N(2))-methyltransferase (EC 2.1.1.172) | -1.02 | 1 |
| lmo0267 | LMRG_02609 | Putative ring-cleaving dioxygenase MhqA | -1.01 | 1 |
| lmo0960 | LMRG_02059 | peptidase, U32 family small subunit [C1] | -1.01 | 1 |
| lmo2490 | LMRG_01758 | Protein CsbA | -1.01 | 1 |
| lmo2691 | LMRG_02238 | N-acetylmuramoyl-L-alanine amidase family 4 (EC 3.5.1.28), needed for cell separation and autolysis | -1.01 | 1 |
| lmo2714 | LMRG_01982 | hypothetical protein | -1.01 | 1 |
| lmo2725 | LMRG_01971 | Multi antimicrobial extrusion protein (Na(+)/drug antiporter), MATE family of MDR efflux pumps | -1.01 | 1 |
| lmo0043 | LMRG_02472 | Arginine deiminase (EC 3.5.3.6) | -1 | 1 |
| lmo0707 | LMRG_00396 | Flagellar cap protein FliD | -1 | 1 |
| lmo0192 | LMRG_02614 | Pur operon repressor PurR | -0.99 | 1 |
| lmo0729 | LMRG_00417 | hypothetical protein | -0.99 | 1 |
| lmo2340 | LMRG_01503 | Pseudouridine 5'-phosphate glycosidase (EC 4.2.1.70) | -0.99 | 1 |
| lmo2194 | LMRG_01638 | Oligopeptide ABC transporter, permease protein OppC (TC 3.A.1.5.1) | -0.98 | 1 |
| lmo0180 | LMRG_02745 | N-acetyl-D-glucosamine ABC transporter, permease protein 2 | -0.97 | 1 |
| lmo0696 | LMRG_00385 | Flagellar basal-body rod modification protein FlgD | -0.97 | 1 |
| lmo2764 | LMRG_01931 | ROK family protein | -0.97 | 1 |
| lmo0574 | LMRG_00256 | 6-phospho-beta-glucosidase (EC 3.2.1.86) | -0.96 | 1 |
| lmo2398 | LMRG_01850 | Low temperature requirement C protein | -0.96 | 1 |
| lmo2471 | LMRG_01777 | NADH:flavin oxidoreductase | -0.96 | 1 |
| lmo0877 | LMRG_02301 | Glucosamine-6-phosphate deaminase (EC 3.5.99.6) | -0.95 | 1 |
| lmo2592 | LMRG_02132 | Oxidoreductase, aldo/keto reductase family | -0.95 | 1 |
| lmo1743 | LMRG_02528 | hypothetical protein | -0.94 | 1 |
| lmo0092 | LMRG_02341 | ATP synthase beta chain (EC 3.6.3.14) | -0.93 | 1 |
| lmo2670 | LMRG_02215 | hypothetical protein | -0.93 | 1 |
| lmo0702 | LMRG_00391 | hypothetical protein | -0.92 | 1 |
| lmo1915 | LMRG_01062 | Malolactic enzyme (EC 1.-.-.-) | -0.92 | 1 |
| lmo1989 | LMRG_01137 | 3-isopropylmalate dehydratase large subunit (EC 4.2.1.33) | -0.92 | 1 |
| lmo2784 | LMRG_01913 | Transcription antiterminator, BglG family | -0.92 | 1 |
| lmo0799 | LMRG_00488 | Blue-light photoreceptor | -0.91 | 1 |
| lmo2242 | LMRG_01590 | Methylated-DNA--protein-cysteine methyltransferase (EC 2.1.1.63) | -0.91 | 1 |
| lmo0426 | LMRG_00118 | PTS system, IIA component | -0.9 | 1 |
| lmo0659 | LMRG_00346 | Transcriptional regulator, MutR family | -0.9 | 1 |
| lmo2418 | LMRG_01830 | Methionine ABC transporter permease protein | -0.9 | 1 |
| lmo0485 | LMRG_00166 | Putative nitroreductase family protein SACOL0874 | -0.89 | 1 |
| lmo2057 | LMRG_01207 | Heme O synthase, protoheme IX farnesyltransferase, COX10-CtaB | -0.89 | 1 |
| lmo2554 | LMRG_01693 | Glycosyltransferase LafB, responsible for the formation of Gal-Glc-DAG | -0.89 | 1 |
| #N/A | LMRG_02494 | FIG00774984: hypothetical protein | -0.89 | 1 |
| lmo0127 | LMRG_02376 | hypothetical protein | -0.88 | 1 |
| lmo0402 | LMRG_00095 | PRD/PTS system IIA 2 domain protein | -0.88 | 1 |
| lmo0985 | LMRG_02085 | hypothetical protein | -0.88 | 1 |
| lmo2779 | LMRG_01918 | GTP-binding and nucleic acid-binding protein YchF | -0.88 | 1 |
| lmo1281 | LMRG_00731 | 4-hydroxybenzoyl-CoA thioesterase | -0.87 | 1 |
| lmo1856 | LMRG_01003 | Purine nucleoside phosphorylase (EC 2.4.2.1) | -0.87 | 1 |
| lmo1894 | LMRG_01041 | Endonuclease III (EC 4.2.99.18) | -0.87 | 1 |
| lmo2560 | LMRG_02709 | DNA-directed RNA polymerase delta subunit (EC 2.7.7.6) | -0.87 | 1 |
| lmo0147 | LMRG_02390 | hypothetical protein | -0.86 | 1 |
| lmo0242 | LMRG_02664 | Uncharacterized protein YacP, similar to C-terminal domain of ribosome protection-type Tc-resistance proteins | -0.86 | 1 |
| lmo0367 | LMRG_00059 | Ferrous iron transport peroxidase EfeB | -0.86 | 1 |
| lmo0681 | LMRG_00369 | Flagellar biosynthesis protein FlhF | -0.86 | 1 |
| lmo1912 | LMRG_01059 | GGDEF domain protein | -0.86 | 1 |
| lmo2221 | LMRG_01611 | Plasmid replication DNA element cmp binding-factor 1 | -0.86 | 1 |
| lmo2657 | LMRG_02202 | Deoxyguanosinetriphosphate triphosphohydrolase (EC 3.1.5.1) | -0.86 | 1 |
| lmo2682 | LMRG_02227 | Potassium-transporting ATPase A chain (EC 3.6.3.12) (TC 3.A.3.7.1) | -0.86 | 1 |
| lmo0375 | LMRG_00067 | hypothetical protein | -0.85 | 1 |
| lmo0910 | LMRG_02010 | hypothetical protein | -0.85 | 1 |
| lmo1092 | LMRG_00554 | Nicotinate phosphoribosyltransferase (EC 6.3.4.21) | -0.85 | 1 |
| lmo2222 | LMRG_02924 | DNA double-strand break repair protein Mre11 | -0.85 | 1 |
| lmo0018 | LMRG_02447 | 6-phospho-beta-glucosidase (EC 3.2.1.86) | -0.84 | 1 |
| lmo2536 | LMRG_01712 | ATP synthase protein I | -0.84 | 1 |
| lmo0530 | LMRG_00212 | hypothetical protein | -0.83 | 1 |
| lmo1326 | LMRG_00776 | YlxP-like protein | -0.83 | 1 |
| lmo1567 | LMRG_01400 | Citrate synthase (si) (EC 2.3.3.1) | -0.83 | 1 |
| lmo2439 | LMRG_01809 | hypothetical protein | -0.82 | 1 |
| lmo2440 | LMRG_01808 | hypothetical protein | -0.81 | 1 |
| lmo0596 | LMRG_00278 | hypothetical protein | -0.8 | 1 |
| lmo1422 | LMRG_00874 | Glycine betaine ABC transport system, permease protein OpuAB / Glycine betaine ABC transport system, glycine betaine-binding protein OpuAC | -0.8 | 1 |
| lmo2646 | LMRG_02191 | Protein of unknown function DUF1341 | -0.8 | 1 |
| lmo0425 | LMRG_00117 | Transcription antiterminator, BglG family | -0.79 | 1 |
| lmo1697 | LMRG_02771 | cation efflux family protein | -0.79 | 1 |
| lmo1840 | LMRG_00987 | Pyrimidine operon regulatory protein PyrR | -0.79 | 1 |
| lmo2539 | LMRG_01708 | Serine hydroxymethyltransferase (EC 2.1.2.1) | -0.79 | 1 |
| lmo0401 | LMRG_00094 | alpha-mannosidase (EC 3.2.1.24) | -0.78 | 1 |
| lmo0427 | LMRG_00119 | PTS system, IIB component | -0.78 | 1 |
| lmo0831 | LMRG_02254 | Auxin efflux carrier family protein | -0.78 | 1 |
| lmo1761 | LMRG_02510 | Sodium-dependent transporter, SNF family | -0.78 | 1 |
| lmo1961 | LMRG_01108 | Thioredoxin reductase (EC 1.8.1.9) | -0.78 | 1 |
| lmo2759 | LMRG_01937 | O-acetyl-ADP-ribose deacetylase | -0.78 | 1 |
| lmo0030 | LMRG_02459 | Hydrolase, haloacid dehalogenase-like family | -0.77 | 1 |
| lmo0298 | LMRG_02570 | PTS system, cellobiose-specific IIC component | -0.77 | 1 |
| lmo0798 | LMRG_02868 | Lysine-specific permease | -0.77 | 1 |
| lmo0935 | LMRG_02034 | tRNA (cytidine(34)-2'-O)-methyltransferase (EC 2.1.1.207) | -0.77 | 1 |
| lmo1921 | LMRG_01068 | UPF0302 protein YpiB | -0.77 | 1 |
| lmo1949 | LMRG_01096 | LSU rRNA pseudouridine(2605) synthase (EC 5.4.99.22) | -0.77 | 1 |
| lmo2158 | LMRG_01674 | hypothetical protein | -0.77 | 1 |
| lmo0354 | LMRG_00045 | CoA ligase | -0.76 | 1 |
| lmo0591 | LMRG_00273 | hypothetical protein | -0.76 | 1 |
| lmo0718 | LMRG_00407 | hypothetical protein | -0.76 | 1 |
| lmo1065 | LMRG_00527 | DUF1054 superfamily protein | -0.76 | 1 |
| lmo1378 | LMRG_00830 | Two-component system sensor histidine kinase | -0.76 | 1 |
| lmo1645 | LMRG_01321 | Exonuclease SbcC | -0.76 | 1 |
| lmo1810 | LMRG_00957 | Transcription factor FapR | -0.76 | 1 |
| lmo2200 | LMRG_01632 | Organic hydroperoxide resistance transcriptional regulator | -0.76 | 1 |
| lmo0441 | LMRG_00133 | Penicillin-binding protein 3 | -0.75 | 1 |
| lmo1256 | LMRG_00705 | hypothetical protein | -0.75 | 1 |
| lmo2226 | LMRG_01606 | Transmembrane protein YxlG | -0.75 | 1 |
| lmo2591 | LMRG_02131 | N-acetylmuramoyl-L-alanine amidase, family 4 | -0.75 | 1 |
| lmo2652 | LMRG_02904 | Putative transcriptional antiterminator, BglG family / PTS system, IIA component | -0.75 | 1 |
| lmo0119 | LMRG_02368 | hypothetical protein | -0.74 | 1 |
| lmo2373 | LMRG_02718 | PTS system, IIB component | -0.74 | 1 |
| lmo0286 | LMRG_02585 | Glutamine-dependent 2-keto-4-methylthiobutyrate transaminase | -0.73 | 1 |
| lmo0319 | LMRG_00012 | 6-phospho-beta-glucosidase (EC 3.2.1.86) | -0.73 | 1 |
| lmo0383 | LMRG_00076 | Malonate-semialdehyde dehydrogenase [inositol] (EC 1.2.1.18) | -0.73 | 1 |
| lmo0428 | LMRG_00120 | PTS system, IIC component | -0.73 | 1 |
| lmo1266 | LMRG_00715 | hypothetical protein | -0.73 | 1 |
| lmo1617 | LMRG_02976 | drug resistance transporter, EmrB/QacA family | -0.73 | 1 |
| lmo1853 | LMRG_01000 | Lead, cadmium, zinc and mercury transporting ATPase (EC 3.6.3.3) (EC 3.6.3.5) | -0.73 | 1 |
| lmo0086 | LMRG_02335 | hypothetical protein | -0.72 | 1 |
| lmo0573 | LMRG_00255 | Hypoxanthine/guanine permease PbuG | -0.72 | 1 |
| lmo0603 | LMRG_00286 | hypothetical protein | -0.72 | 1 |
| lmo1705 | LMRG_02779 | Deoxyadenosine kinase (EC 2.7.1.76) @ Deoxyguanosine kinase (EC 2.7.1.113) | -0.72 | 1 |
| lmo2386 | LMRG_02731 | Uncharacterized membrane protein YuiD | -0.72 | 1 |
| #N/A | LMRG_01255 | FIG00774476: hypothetical protein | -0.72 | 1 |
| lmo0130 | LMRG_02379 | 5'-nucleotidase (EC 3.1.3.5) | -0.71 | 1 |
| lmo2355 | LMRG_01488 | Multidrug resistance protein | -0.71 | 1 |
| lmo1305 | LMRG_00755 | Transketolase (EC 2.2.1.1) | -0.7 | 1 |
| lmo0457 | LMRG_00149 | Conservative hypothetical protein probably involved in hydantoin, pyrimidine utilization | -0.69 | 1 |
| lmo1774 | LMRG_02497 | N5-carboxyaminoimidazole ribonucleotide synthase (EC 6.3.4.18) | -0.69 | 1 |
| lmo1785 | LMRG_02827 | Translation initiation factor 3 | -0.69 | 1 |
| lmo2577 | LMRG_02691 | Cof-like hydrolase, HAD-superfamily, subfamily IIB | -0.69 | 1 |
| lmo2787 | LMRG_01910 | PTS system, beta-glucoside-specific IIB component / PTS system, beta-glucoside-specific IIC component / PTS system, beta-glucoside-specific IIA component | -0.69 | 1 |
| lmo0207 | LMRG_02629 | putative lipoprotein | -0.68 | 1 |
| lmo0818 | LMRG_02243 | Cation-transporting ATPase, E1-E2 family | -0.68 | 1 |
| lmo1401 | LMRG_00853 | 2',3'-cyclic-nucleotide 2'-phosphodiesterase, Bsub YmdB | -0.68 | 1 |
| lmo2573 | LMRG_02695 | Zinc-type alcohol dehydrogenase-like protein | -0.68 | 1 |
| lmo2824 | LMRG_02985 | D-3-phosphoglycerate dehydrogenase (EC 1.1.1.95) | -0.68 | 1 |
| lmo0305 | LMRG_00002 | Low-specificity L-threonine aldolase (EC 4.1.2.48) | -0.67 | 1 |
| lmo0611 | LMRG_00294 | FMN-dependent NADH-azoreductase (EC 1.7.1.6) | -0.67 | 1 |
| lmo0982 | LMRG_02082 | Deblocking aminopeptidase (EC 3.4.11.-) | -0.67 | 1 |
| lmo1865 | LMRG_01012 | Transcriptional repressor CcpN, MarR family | -0.67 | 1 |
| lmo2434 | LMRG_01814 | Glutamate decarboxylase (EC 4.1.1.15) | -0.67 | 1 |
| lmo2697 | LMRG_02000 | Phosphoenolpyruvate-dihydroxyacetone phosphotransferase (EC 2.7.1.121), subunit DhaM | -0.67 | 1 |
| lmo0713 | LMRG_00402 | Flagellar M-ring protein FliF | -0.66 | 1 |
| lmo0724 | LMRG_00413 | hypothetical protein | -0.66 | 1 |
| lmo0753 | LMRG_00441 | Transcriptional regulator in BltB locus | -0.66 | 1 |
| lmo2687 | LMRG_02233 | Similar to rod shape-determining protein RodA | -0.66 | 1 |
| lmo1291 | LMRG_00741 | Peptidoglycan O-acetyltransferase YrhL | -0.65 | 1 |
| lmo1967 | LMRG_01114 | Uncharacterized protein YaaN | -0.65 | 1 |
| lmo2462 | LMRG_01786 | Microsomal dipeptidase precursor (EC 3.4.13.19) | -0.65 | 1 |
| lmo2816 | LMRG_01882 | Uncharacterized MFS-type transporter | -0.65 | 1 |
| lmo0423 | LMRG_00115 | RNA polymerase ECF-type sigma factor | -0.64 | 1 |
| lmo1132 | LMRG_00574 | Heterodimeric efflux ABC transporter, permease/ATP-binding subunit 2 | -0.64 | 1 |
| lmo1621 | LMRG_01345 | MutT/Nudix family protein | -0.64 | 1 |
| lmo2604 | LMRG_02148 | hypothetical protein | -0.64 | 1 |
| lmo0234 | LMRG_02672 | Membrane-associated protein containing RNA-binding TRAM domain and ribonuclease PIN-domain, YacL B.subtilis ortholog | -0.63 | 1 |
| lmo0824 | LMRG_02249 | hypothetical protein | -0.63 | 1 |
| lmo1225 | LMRG_00671 | Transcriptional regulator, MarR family | -0.63 | 1 |
| lmo1652 | LMRG_01315 | Efflux ABC transporter, permease/ATP-binding protein MdlA | -0.63 | 1 |
| lmo2795 | LMRG_01902 | Sialic acid utilization regulator, RpiR family | -0.63 | 1 |
| lmor01 | LMRG_05501 | SSU rRNA ## 16S rRNA, small subunit ribosomal RNA | -0.63 | 1 |
| lmo0029 | LMRG_02458 | hypothetical protein | -0.62 | 1 |
| lmo0169 | LMRG_02414 | Glucose:proton symporter GlcU | -0.62 | 1 |
| lmo0615 | LMRG_00298 | hypothetical protein | -0.61 | 1 |
| lmo0998 | LMRG_02954 | CAAX amino terminal protease family protein | -0.61 | 1 |
| lmo0608 | LMRG_00291 | Efflux ABC transporter, permease/ATP-binding protein EF2592 | -0.6 | 1 |
| lmo1004 | LMRG_02104 | hypothetical protein | -0.6 | 1 |
| lmo1381 | LMRG_00833 | Acylphosphate phosphohydrolase (EC 3.6.1.7) | -0.6 | 1 |
| lmo2067 | LMRG_01217 | Choloylglycine hydrolase (EC 3.5.1.24) | -0.6 | 1 |
| lmo2056 | LMRG_01206 | Allergen V5/Tpx-1 related | -0.59 | 1 |
| lmo2416 | LMRG_01832 | hypothetical protein | -0.59 | 1 |
| lmo2661 | LMRG_02206 | Ribulose-phosphate 3-epimerase (EC 5.1.3.1) | -0.59 | 1 |
| lmor04 | LMRG_05504 | SSU rRNA ## 16S rRNA, small subunit ribosomal RNA | -0.59 | 1 |
| lmo0188 | LMRG_02753 | SSU rRNA (adenine(1518)-N(6)/adenine(1519)-N(6))-dimethyltransferase (EC 2.1.1.182) | -0.58 | 1 |
| lmo0839 | LMRG_02262 | Uncharacterized MFS-type transporter | -0.58 | 1 |
| lmo1674 | LMRG_01293 | 2-succinyl-6-hydroxy-2,4-cyclohexadiene-1-carboxylate synthase (EC 4.2.99.20) | -0.58 | 1 |
| lmo2558 | LMRG_02909 | Autolysin, amidase | -0.58 | 1 |
| lmo0035 | LMRG_02464 | putative glucosamine-fructose-6-phosphate aminotransferase | -0.57 | 1 |
| lmo0094 | LMRG_02343 | hypothetical protein | -0.57 | 1 |
| lmo0323 | LMRG_00015 | Endonuclease/exonuclease/phosphatase family protein | -0.57 | 1 |
| lmo0750 | LMRG_00438 | hypothetical protein | -0.57 | 1 |
| lmo1059 | LMRG_00521 | hypothetical protein | -0.57 | 1 |
| lmo1224 | LMRG_00670 | ABC transporter, coiled-coil domain and fused permease subunit | -0.57 | 1 |
| lmo1539 | LMRG_01431 | Glycerol uptake facilitator protein | -0.57 | 1 |
| lmo2245 | LMRG_01587 | Uncharacterized protein YqjT | -0.57 | 1 |
| lmo0133 | LMRG_02382 | Uncharacterized protein YjdI | -0.56 | 1 |
| lmo0904 | LMRG_02004 | hypothetical protein | -0.56 | 1 |
| lmo1292 | LMRG_00742 | Glycerophosphoryl diester phosphodiesterase (EC 3.1.4.46) | -0.56 | 1 |
| lmo1914 | LMRG_01061 | EAL domain protein | -0.56 | 1 |
| lmo2105 | LMRG_01258 | Ferrous iron transporter FeoB | -0.56 | 1 |
| lmo2393 | LMRG_02738 | hypothetical protein | -0.56 | 1 |
| lmo2407 | LMRG_01841 | hypothetical protein | -0.56 | 1 |
| lmo2464 | LMRG_01784 | Transcriptional regulator, AcrR family | -0.56 | 1 |
| lmo2745 | LMRG_01951 | Multidrug resistance ABC transporter permease/ATP-binding protein BmrA | -0.56 | 1 |
| lmo0313 | LMRG_02933 | hypothetical protein | -0.55 | 1 |
| lmo0906 | LMRG_02006 | Glutathione reductase (EC 1.8.1.7) | -0.55 | 1 |
| lmo0911 | LMRG_02011 | hypothetical protein | -0.55 | 1 |
| lmo1844 | LMRG_00991 | Lipoprotein signal peptidase (EC 3.4.23.36) | -0.55 | 1 |
| lmo2728 | LMRG_01968 | Transcriptional regulator AdhR, MerR family | -0.55 | 1 |
| lmo1388 | LMRG_00840 | Purine nucleoside ABC transporter, substrate-binding protein @ CD4+ T cell-stimulating antigen, lipoprotein | -0.54 | 1 |
| lmo1960 | LMRG_01107 | Ferrichrome transport ATP-binding protein FhuC | -0.54 | 1 |
| lmo2195 | LMRG_01637 | Oligopeptide ABC transporter, permease protein OppB (TC 3.A.1.5.1) | -0.54 | 1 |
| lmo0443 | LMRG_00135 | Cell envelope-associated transcriptional attenuator LytR-CpsA-Psr, subfamily F1 | -0.53 | 1 |
| lmo0665 | LMRG_00352 | hypothetical protein | -0.53 | 1 |
| lmo0981 | LMRG_02081 | Uncharacterized MFS-type transporter | -0.53 | 1 |
| lmo1061 | LMRG_00523 | Two-component system sensor histidine kinase | -0.53 | 1 |
| lmo1530 | LMRG_01440 | Queuine tRNA-ribosyltransferase (EC 2.4.2.29) | -0.53 | 1 |
| lmo0106 | LMRG_02355 | Sugar kinase and transcription regulator (EC 2.7.1.-) | -0.52 | 1 |
| lmo1713 | LMRG_02556 | MreB-like protein (Mbl protein) | -0.52 | 1 |
| lmo1762 | LMRG_02509 | hypothetical protein | -0.52 | 1 |
| lmo2738 | LMRG_01958 | Hemolysins and related proteins containing CBS domains | -0.52 | 1 |
| lmos79 | #N/A | hypothetical protein | -0.52 | 1 |
| lmo1134 | LMRG_00576 | Transcriptional regulator, MerR family | -0.51 | 1 |
| lmo1389 | LMRG_00841 | Purine nucleoside ABC transporter, ATP-binding protein | -0.51 | 1 |
| lmo1794 | LMRG_02818 | Uncharacterized protein YlqD | -0.51 | 1 |
| lmo2463 | LMRG_01785 | transporter, MMPL family | -0.51 | 1 |
| lmo0205 | LMRG_02627 | Broad-substrate range phospholipase C (EC 3.1.4.3) | -0.5 | 1 |
| lmo0554 | LMRG_00236 | NADH-dependent butanol dehydrogenase A (EC 1.1.1.-) | -0.5 | 1 |
| lmo1251 | LMRG_00697 | Transcriptional regulator, Crp/Fnr family | -0.5 | 1 |
| lmo1259 | LMRG_00708 | Gamma-glutamyl phosphate reductase (EC 1.2.1.41) | -0.5 | 1 |
| lmo1300 | LMRG_00750 | Arsenite/antimonite:H+ antiporter ArsB | -0.5 | 1 |
| lmo1523 | LMRG_01447 | Guanosine-3',5'-bis(diphosphate) 3'-pyrophosphohydrolase (EC 3.1.7.2) / GTP pyrophosphokinase (EC 2.7.6.5), (p)ppGpp synthetase II | -0.5 | 1 |
| lmo2675 | LMRG_02220 | hypothetical protein | -0.5 | 1 |
| lmo0584 | LMRG_00266 | UPF0118 membrane protein YubA | -0.49 | 1 |
| lmo2224 | LMRG_01608 | Hypothetical protein SAV1846 | -0.49 | 1 |
| lmo2389 | LMRG_02734 | NADH dehydrogenase (EC 1.6.99.3) | -0.49 | 1 |
| lmo0447 | LMRG_00139 | Glutamate decarboxylase (EC 4.1.1.15) | -0.48 | 1 |
| lmo0705 | LMRG_00394 | Flagellar hook-associated protein FlgK | -0.48 | 1 |
| lmo1976 | LMRG_01123 | Oxidoreductase, short-chain dehydrogenase/reductase family | -0.48 | 1 |
| lmo2501 | LMRG_01747 | Alkaline phosphatase synthesis transcriptional regulatory protein PhoP | -0.48 | 1 |
| lmo2190 | LMRG_01642 | ClpCP protease substrate adapter protein MecA | -0.47 | 1 |
| lmo2341 | LMRG_01502 | Pseudouridine kinase (EC 2.7.1.83) | -0.47 | 1 |
| lmo0664 | LMRG_00351 | Maltose O-acetyltransferase (EC 2.3.1.79) | -0.46 | 1 |
| lmo0110 | LMRG_02359 | Esterase/lipase | -0.45 | 1 |
| lmo0617 | LMRG_00300 | hypothetical protein | -0.45 | 1 |
| lmo0973 | LMRG_02072 | D-alanyl transfer protein DltB | -0.45 | 1 |
| lmo1781 | LMRG_02831 | hypothetical protein | -0.45 | 1 |
| lmo2203 | LMRG_01629 | N-acetylmuramoyl-L-alanine amidase, family 4 | -0.45 | 1 |
| lmo0206 | LMRG_02628 | Distant similarity with viral glycoprotein gp160 of HIV type 1 | -0.44 | 1 |
| lmo0221 | LMRG_02643 | Pantothenate kinase type III, CoaX-like (EC 2.7.1.33) | -0.44 | 1 |
| lmo0343 | LMRG_00034 | Transaldolase (EC 2.2.1.2) | -0.44 | 1 |
| lmo0191 | LMRG_02963 | Cellobiose phosphotransferase system YdjC-like protein | -0.43 | 1 |
| lmo0194 | LMRG_02616 | ABC-type antimicrobial peptide transport system, ATPase component | -0.43 | 1 |
| lmo0359 | LMRG_00050 | Fructose-bisphosphate aldolase class II (EC 4.1.2.13) homolog | -0.43 | 1 |
| lmo2356 | LMRG_01486 | hypothetical protein | -0.43 | 1 |
| lmo2680 | LMRG_02225 | Potassium-transporting ATPase C chain (EC 3.6.3.12) (TC 3.A.3.7.1) | -0.43 | 1 |
| lmo0257 | LMRG_02652 | RNA-2',3'-PO4:RNA-5'-OH ligase | -0.42 | 1 |
| lmo1396 | LMRG_00848 | CDP-diacylglycerol--glycerol-3-phosphate 3-phosphatidyltransferase (EC 2.7.8.5) | -0.42 | 1 |
| lmo2826 | LMRG_01872 | Uncharacterized MFS-type transporter | -0.42 | 1 |
| lmo0164 | LMRG_02409 | DNA replication intiation control protein YabA | -0.41 | 1 |
| lmo0178 | LMRG_02743 | ROK family sugar kinase or transcriptional regulator | -0.41 | 1 |
| lmo0384 | LMRG_00077 | 5-deoxy-glucuronate isomerase (EC 5.3.1.30) | -0.41 | 1 |
| lmo0634 | LMRG_00317 | hypothetical protein | -0.41 | 1 |
| lmo1408 | LMRG_00860 | Transcriptional regulator, PadR family | -0.41 | 1 |
| #N/A | LMRG_02850 | FIG00774088: hypothetical protein | -0.41 | 1 |
| lmo0214 | LMRG_02636 | Transcription-repair coupling factor | -0.4 | 1 |
| lmo1475 | LMRG_00928 | Heat-inducible transcription repressor HrcA | -0.4 | 1 |
| lmo1938 | LMRG_01085 | SSU ribosomal protein S1p | -0.4 | 1 |
| lmo2133 | LMRG_02807 | Fructose-bisphosphate aldolase class II (EC 4.1.2.13) homolog | -0.4 | 1 |
| lmo2839 | LMRG_01859 | ABC-type sugar transport system, periplasmic binding protein YcjN | -0.4 | 1 |
| lmo0341 | LMRG_00032 | hypothetical protein | -0.39 | 1 |
| lmo1317 | LMRG_00767 | 1-deoxy-D-xylulose 5-phosphate reductoisomerase (EC 1.1.1.267) | -0.39 | 1 |
| lmo2636 | LMRG_02181 | FAD:protein FMN transferase (EC 2.7.1.180) @ FAD:protein FMN transferase (EC 2.7.1.180), HepST-associated | -0.39 | 1 |
| lmo2695 | LMRG_02002 | Phosphoenolpyruvate-dihydroxyacetone phosphotransferase (EC 2.7.1.121), dihydroxyacetone binding subunit DhaK | -0.39 | 1 |
| lmo0008 | LMRG_02436 | Cardiolipin synthase, bacterial type ClsA | -0.38 | 1 |
| lmo0807 | LMRG_02790 | Spermidine/putrescine import ABC transporter ATP-binding protein PotA (TC 3.A.1.11.1) | -0.38 | 1 |
| lmo0879 | LMRG_02303 | Sugar phosphate isomerases/epimerases | -0.38 | 1 |
| lmo1479 | LMRG_00932 | Translation elongation factor LepA | -0.38 | 1 |
| lmo1678 | LMRG_01289 | 5,10-methylenetetrahydrofolate reductase (EC 1.5.1.20) / Homolog of homocysteine-binding domain | -0.38 | 1 |
| lmo2752 | LMRG_01944 | Heterodimeric efflux ABC transporter, multidrug resistance => LmrD subunit of LmrCD | -0.38 | 1 |
| #N/A | LMRG_02139 | FIG00774503: hypothetical protein | -0.38 | 1 |
| lmor12 | LMRG_05512 | SSU rRNA ## 16S rRNA, small subunit ribosomal RNA | -0.38 | 1 |
| lmor18 | LMRG_05518 | SSU rRNA ## 16S rRNA, small subunit ribosomal RNA | -0.38 | 1 |
| lmo0663 | LMRG_00350 | Cof-like hydrolase | -0.37 | 1 |
| lmo1744 | LMRG_02527 | Isoflavone_redu, Isoflavone reductase | -0.37 | 1 |
| lmo2589 | LMRG_02678 | Transcriptional regulator, AcrR family | -0.37 | 1 |
| lmo0162 | LMRG_02407 | DNA polymerase III delta prime subunit (EC 2.7.7.7) | -0.36 | 1 |
| lmo1795 | LMRG_02819 | Glutamine amidotransferase, class I | -0.36 | 1 |
| lmo2007 | LMRG_01155 | ABC transporter, substrate-binding protein (cluster 1, maltose/g3p/polyamine/iron) | -0.36 | 1 |
| lmo2454 | LMRG_01794 | hypothetical protein | -0.36 | 1 |
| lmo0633 | LMRG_00316 | PTS system, fructose-specific IIB component (EC 2.7.1.202) | -0.35 | 1 |
| lmo0661 | LMRG_00348 | carboxymuconolactone decarboxylase family protein | -0.35 | 1 |
| lmo0890 | LMRG_02314 | RsbS, negative regulator of sigma-B | -0.35 | 1 |
| lmo0974 | LMRG_02073 | D-alanine--poly(phosphoribitol) ligase subunit 1 (EC 6.1.1.13) | -0.35 | 1 |
| lmo1514 | LMRG_01456 | Replication-associated recombination protein RarA | -0.35 | 1 |
| lmo2115 | LMRG_01269 | ABC transporter-like sensor and permease protein | -0.35 | 1 |
| lmo2142 | LMRG_02798 | hypothetical protein | -0.35 | 1 |
| lmo0688 | LMRG_00376 | Glycosyl transferase, group 2 family | -0.34 | 1 |
| lmo1223 | LMRG_00669 | ABC transporter, ATP-binding protein | -0.34 | 1 |
| lmo1780 | LMRG_02832 | Tripeptide aminopeptidase (EC 3.4.11.4) | -0.34 | 1 |
| lmo1971 | LMRG_01118 | PTS system, IIC component, UlaA-type | -0.34 | 1 |
| lmo1987 | LMRG_01135 | 2-isopropylmalate synthase (EC 2.3.3.13) | -0.34 | 1 |
| lmo2255 | LMRG_01576 | hypothetical protein | -0.34 | 1 |
| lmo2721 | LMRG_01975 | Uncharacterized protein YieK | -0.34 | 1 |
| lmo0389 | LMRG_00082 | Low temperature requirement protein A | -0.33 | 1 |
| lmo0606 | LMRG_00289 | Transcriptional regulator, MarR family | -0.33 | 1 |
| lmo0686 | LMRG_00374 | Flagellar motor rotation protein MotB | -0.33 | 1 |
| lmo1160 | LMRG_00603 | Phosphate propanoyltransferase (EC 2.3.1.222) | -0.33 | 1 |
| lmo1718 | LMRG_02553 | Outer surface protein of unknown function, cellobiose operon | -0.33 | 1 |
| lmo1752 | LMRG_02519 | hypothetical protein | -0.33 | 1 |
| lmo2134 | LMRG_02806 | Fructose-bisphosphate aldolase class II (EC 4.1.2.13) homolog | -0.33 | 1 |
| lmo0385 | LMRG_00078 | 5-keto-2-deoxygluconokinase (EC 2.7.1.92) | -0.32 | 1 |
| lmo0671 | LMRG_00359 | hypothetical protein | -0.32 | 1 |
| lmo0781 | LMRG_00469 | PTS system, mannose-specific IID component | -0.32 | 1 |
| lmo1449 | LMRG_00901 | Endonuclease IV (EC 3.1.21.2) | -0.32 | 1 |
| lmo2342 | LMRG_01501 | SSU rRNA pseudouridine(516) synthase (EC 5.4.99.19) | -0.32 | 1 |
| lmo0084 | LMRG_02332 | Oxidoreductase, aldo/keto reductase family | -0.31 | 1 |
| lmo0694 | LMRG_02872 | hypothetical protein | -0.31 | 1 |
| lmo0876 | LMRG_02300 | PTS system, beta-glucoside-specific, IIC component | -0.31 | 1 |
| lmo1839 | LMRG_00986 | Uracil permease @ Uracil:proton symporter UraA | -0.31 | 1 |
| lmo1850 | LMRG_00997 | Transcriptional regulator, MarR family | -0.31 | 1 |
| lmo2724 | LMRG_01972 | PhnB protein | -0.31 | 1 |
| lmor09 | LMRG_05509 | SSU rRNA ## 16S rRNA, small subunit ribosomal RNA | -0.31 | 1 |
| lmo0223 | LMRG_02645 | Cysteine synthase (EC 2.5.1.47) | -0.3 | 1 |
| lmo0544 | LMRG_00226 | PTS system, glucitol/sorbitol-specific IIC component | -0.3 | 1 |
| lmo0673 | LMRG_00361 | hypothetical protein | -0.3 | 1 |
| lmo0028 | LMRG_02457 | Muramoyltetrapeptide carboxypeptidase (EC 3.4.17.13) | -0.29 | 1 |
| lmo0196 | LMRG_02618 | DNA-binding protein SpoVG | -0.29 | 1 |
| lmo0565 | LMRG_00247 | Imidazole glycerol phosphate synthase amidotransferase subunit HisH | -0.29 | 1 |
| lmo1702 | LMRG_02776 | Fosfomycin resistance protein FosX | -0.29 | 1 |
| lmo2051 | LMRG_01201 | Lon-like protease with PDZ domain | -0.29 | 1 |
| lmo2550 | LMRG_01697 | Dolichol-phosphate mannosyltransferase MtrA | -0.29 | 1 |
| lmo2765 | LMRG_01930 | PTS system, cellobiose-specific IIA component (EC 2.7.1.205) | -0.29 | 1 |
| #N/A | LMRG_02848 | hypothetical protein | -0.29 | 1 |
| lmo0082 | LMRG_02330 | hypothetical protein | -0.28 | 1 |
| lmo0282 | LMRG_02589 | Aliphatic amidase AmiE (EC 3.5.1.4) | -0.28 | 1 |
| lmo0672 | LMRG_00360 | Membrane protein | -0.28 | 1 |
| lmo0738 | LMRG_00426 | PTS system, beta-glucoside-specific IIB component / PTS system, beta-glucoside-specific IIC component / PTS system, beta-glucoside-specific IIA component | -0.28 | 1 |
| lmo1699 | LMRG_02773 | Methyl-accepting chemotaxis protein | -0.28 | 1 |
| lmo1995 | LMRG_01143 | Deoxyribose-phosphate aldolase (EC 4.1.2.4) | -0.28 | 1 |
| lmo1354 | LMRG_00804 | Aminopeptidase YpdF (MP-, MA-, MS-, AP-, NP- specific) | -0.27 | 1 |
| lmo2161 | LMRG_01671 | Trehalose utilization protein ThuA | -0.27 | 1 |
| lmo2163 | LMRG_01669 | Myo-inositol 2-dehydrogenase 1 (EC 1.1.1.18) | -0.27 | 1 |
| lmo2827 | LMRG_01871 | Transcriptional regulator, MarR family | -0.27 | 1 |
| lmor15 | LMRG_05515 | SSU rRNA ## 16S rRNA, small subunit ribosomal RNA | -0.27 | 1 |
| lmo0173 | N/A | Mobile element protein | -0.26 | 1 |
| lmo0995 | LMRG_02095 | Putative membrane-bound acyltransferase YkrP | -0.26 | 1 |
| lmo1990 | LMRG_01138 | 3-isopropylmalate dehydratase small subunit (EC 4.2.1.33) | -0.26 | 1 |
| lmo2735 | LMRG_01961 | Uncharacterized glycosyltransferase YcjM | -0.26 | 1 |
| lmo2818 | LMRG_01880 | Uncharacterized MFS-type transporter | -0.26 | 1 |
| lmo0833 | LMRG_02256 | transcriptional activator | -0.25 | 1 |
| lmo0838 | LMRG_02261 | Sugar phosphate transporter | -0.25 | 1 |
| lmo1168 | LMRG_00611 | Propionate kinase, propanediol utilization (EC 2.7.2.1) | -0.25 | 1 |
| lmo1238 | LMRG_00684 | Ribonuclease PH (EC 2.7.7.56) | -0.25 | 1 |
| lmo1511 | LMRG_01459 | Hydrolase, alpha/beta fold family | -0.25 | 1 |
| lmo1940 | LMRG_01087 | L-asparaginase (EC 3.5.1.1) | -0.25 | 1 |
| lmo2679 | LMRG_02224 | Osmosensitive K+ channel histidine kinase KdpD | -0.25 | 1 |
| lmo2842 | LMRG_01856 | Transcriptional regulator YcjW, LacI family, possibly involved in maltodextrin utilization pathway | -0.25 | 1 |
| lmo0830 | LMRG_02253 | Fructose-1,6-bisphosphatase, Bacillus type (EC 3.1.3.11) | -0.24 | 1 |
| lmo0988 | LMRG_02088 | Peptide chain release factor 3 | -0.24 | 1 |
| lmo1538 | LMRG_01432 | Glycerol kinase (EC 2.7.1.30) | -0.24 | 1 |
| lmo2823 | LMRG_01875 | FIG008208: hypothetical protein | -0.24 | 1 |
| lmo0295 | LMRG_02574 | Acetyltransferase, GNAT family | -0.23 | 1 |
| lmo0676 | LMRG_00364 | Flagellar biosynthesis protein FliP | -0.23 | 1 |
| lmo0980 | LMRG_02080 | Efflux ABC transporter, permease protein | -0.23 | 1 |
| lmo1005 | LMRG_02105 | 2-hydroxy-3-oxopropionate reductase (EC 1.1.1.60) | -0.23 | 1 |
| lmo2374 | LMRG_02719 | Aspartokinase (EC 2.7.2.4) | -0.23 | 1 |
| lmo0888 | LMRG_02312 | Programmed cell death toxin YdcE | -0.22 | 1 |
| lmo1409 | LMRG_00861 | Uncharacterized MFS-type transporter | -0.22 | 1 |
| lmo1727 | LMRG_02544 | Transcriptional regulator, LacI family | -0.22 | 1 |
| lmo2360 | LMRG_01482 | hypothetical protein | -0.22 | 1 |
| lmo0524 | LMRG_00205 | Sulfate transporter family protein | -0.21 | 1 |
| lmo0938 | LMRG_02037 | Low molecular weight protein tyrosine phosphatase (EC 3.1.3.48) | -0.21 | 1 |
| lmo1867 | LMRG_01014 | Pyruvate,phosphate dikinase (EC 2.7.9.1) | -0.21 | 1 |
| lmo2377 | LMRG_02722 | Uncharacterized MFS-type transporter YuxJ | -0.21 | 1 |
| lmo2754 | LMRG_01942 | D-alanyl-D-alanine carboxypeptidase (EC 3.4.16.4) | -0.21 | 1 |
| lmo0706 | LMRG_00395 | Flagellar hook-associated protein FlgL | -0.2 | 1 |
| lmo0847 | LMRG_02270 | Glutamine ABC transporter, substrate-binding protein GlnH / Glutamine ABC transporter, permease protein GlnP | -0.2 | 1 |
| lmo1293 | LMRG_00743 | Aerobic glycerol-3-phosphate dehydrogenase (EC 1.1.5.3) | -0.2 | 1 |
| lmo1407 | LMRG_00859 | Pyruvate formate-lyase activating enzyme (EC 1.97.1.4) | -0.2 | 1 |
| lmo1875 | LMRG_01022 | Bis-ABC ATPase Uup | -0.2 | 1 |
| lmo1941 | LMRG_01088 | hypothetical protein | -0.2 | 1 |
| lmo2364 | LMRG_02917 | hypothetical protein | -0.2 | 1 |
| lmo0123 | LMRG_02372 | Phage tail fiber | -0.19 | 1 |
| lmo0409 | LMRG_00102 | Internalin-like protein (LPXTG motif) Lmo0409 homolog | -0.19 | 1 |
| lmo0629 | LMRG_00312 | Isochorismatase (EC 3.3.2.1) | -0.19 | 1 |
| lmo1760 | LMRG_02511 | Heptaprenylglyceryl phosphate synthase (EC 2.5.1.n9) | -0.19 | 1 |
| lmo2590 | LMRG_02130 | ATP-binding protein, Mrp/Nbp35 family | -0.19 | 1 |
| lmo2829 | LMRG_01869 | Nitroreductase family protein | -0.19 | 1 |
| lmor11 | LMRG_05511 | LSU rRNA ## 23S rRNA, large subunit ribosomal RNA | -0.19 | 1 |
| lmor14 | LMRG_05514 | LSU rRNA ## 23S rRNA, large subunit ribosomal RNA | -0.19 | 1 |
| lmor17 | LMRG_05517 | LSU rRNA ## 23S rRNA, large subunit ribosomal RNA | -0.19 | 1 |
| lmo0031 | LMRG_02460 | Transcriptional regulator, LacI family | -0.18 | 1 |
| lmo0294 | LMRG_02573 | Transcriptional regulator, LysR family | -0.18 | 1 |
| lmo2263 | LMRG_01568 | Putative phosphatase YitU | -0.18 | 1 |
| lmo0397 | LMRG_00090 | hypothetical protein | -0.17 | 1 |
| lmo2403 | LMRG_01845 | 5'-nucleotidase family protein in cluster with NagD-like phosphatase | -0.17 | 1 |
| lmo2638 | LMRG_02183 | NADH dehydrogenase (EC 1.6.99.3) in cluster with putative pheromone precursor | -0.17 | 1 |
| lmo2822 | LMRG_01876 | hypothetical protein | -0.17 | 1 |
| lmo0514 | LMRG_00195 | Internalin-like protein (LPXTG motif) Lmo0514 homolog | -0.16 | 1 |
| lmo0773 | LMRG_00461 | Acryloyl-CoA reductase AcuI/YhdH (EC 1.3.1.84) | -0.16 | 1 |
| lmo1030 | LMRG_00491 | Unknown pentose utilization regulator, LacI family | -0.16 | 1 |
| lmo1188 | LMRG_00634 | Putative major teichoic acid biosynthesis protein C | -0.16 | 1 |
| lmo1742 | LMRG_02529 | Adenine deaminase (EC 3.5.4.2) | -0.16 | 1 |
| lmo2854 | LMRG_02425 | Inner membrane protein translocase and chaperone YidC, short form OxaI-like | -0.16 | 1 |
| lmo0864 | LMRG_02287 | FIG01423360: glycoside hydrolase | -0.15 | 1 |
| lmo0990 | LMRG_02090 | Multi antimicrobial extrusion protein (Na(+)/drug antiporter), MATE family of MDR efflux pumps | -0.15 | 1 |
| lmo2185 | LMRG_01647 | Cell surface protein IsdA, transfers heme from hemoglobin to apo-IsdC | -0.15 | 1 |
| lmo2852 | LMRG_02423 | hypothetical protein | -0.15 | 1 |
| lmor05 | LMRG_05505 | LSU rRNA ## 23S rRNA, large subunit ribosomal RNA | -0.15 | 1 |
| lmor08 | LMRG_05508 | LSU rRNA ## 23S rRNA, large subunit ribosomal RNA | -0.15 | 1 |
| lmot04 | LMRG_05004 | tRNA-Lys-TTT | -0.15 | 1 |
| lmo0852 | LMRG_02275 | Transcriptional regulator, AcrR family | -0.14 | 1 |
| lmo1081 | LMRG_00543 | Glucose-1-phosphate thymidylyltransferase (EC 2.7.7.24) | -0.14 | 1 |
| lmo1607 | LMRG_01359 | Phenylalanyl-tRNA synthetase domain protein (Bsu YtpR) | -0.14 | 1 |
| lmo1734 | LMRG_02537 | Glutamate synthase [NADPH] large chain (EC 1.4.1.13) | -0.14 | 1 |
| lmo2303 | LMRG_01529 | LmaC, associated with virulence in Listeria | -0.14 | 1 |
| lmo2445 | LMRG_01803 | Internalin-like protein Lmo2445 homolog | -0.14 | 1 |
| lmo2678 | LMRG_02223 | DNA-binding response regulator KdpE | -0.14 | 1 |
| lmo0585 | LMRG_00267 | Extracellular protein | -0.13 | 1 |
| lmo0841 | LMRG_02264 | Cation-transporting ATPase | -0.13 | 1 |
| lmo1393 | LMRG_00845 | Uncharacterized zinc protease YmfH | -0.13 | 1 |
| lmo2644 | LMRG_02189 | hypothetical protein | -0.13 | 1 |
| #N/A | LMRG_RS14955 | Cassette chromosome recombinase B | -0.13 | 1 |
| lmo1390 | LMRG_00842 | Purine nucleoside ABC transporter, permease protein 1 | -0.12 | 1 |
| lmo0529 | LMRG_00211 | Uncharacterized glycosyltransferase YdaM | -0.11 | 1 |
| lmo0758 | LMRG_00446 | Glyoxalase in BtlB locus | -0.11 | 1 |
| lmo1701 | LMRG_02775 | hypothetical protein | -0.11 | 1 |
| lmo1750 | LMRG_02521 | Molybdate metabolism regulator | -0.11 | 1 |
| lmo1952 | LMRG_01099 | Diaminopimelate decarboxylase (EC 4.1.1.20) | -0.11 | 1 |
| lmo2129 | LMRG_01283 | hypothetical protein | -0.11 | 1 |
| lmot45 | LMRG_05045 | tRNA-Lys-TTT | -0.11 | 1 |
| lmo0422 | LMRG_00114 | Transcriptional regulator, PadR family | -0.1 | 1 |
| lmo0481 | LMRG_00162 | Oleate hydratase (EC 4.2.1.53) | -0.1 | 1 |
| lmo0605 | LMRG_00288 | Na+-driven multidrug efflux pump | -0.1 | 1 |
| lmo1318 | LMRG_00768 | Intramembrane protease RasP/YluC, implicated in cell division based on FtsL cleavage | -0.1 | 1 |
| lmo1623 | LMRG_01343 | putative membrane-associated phospholipid phosphatase, PAP2 superfamily | -0.1 | 1 |
| lmo2767 | LMRG_01928 | hypothetical protein | -0.1 | 1 |
| lmor02 | LMRG_05502 | LSU rRNA ## 23S rRNA, large subunit ribosomal RNA | -0.1 | 1 |
| lmo0052 | LMRG_02481 | Cyclic-di-AMP phosphodiesterase GdpP | -0.09 | 1 |
| lmo1625 | LMRG_01341 | Probable cell division protein YtgP | -0.09 | 1 |
| lmo1638 | LMRG_01328 | Microcin C7 self-immunity protein mccF | -0.09 | 1 |
| lmo2015 | LMRG_01164 | alpha-mannosidase (EC 3.2.1.24) | -0.09 | 1 |
| lmo2781 | LMRG_01916 | beta-glucosidase (EC 3.2.1.21) | -0.09 | 1 |
| lmo0014 | LMRG_02443 | Cytochrome aa3-600 menaquinol oxidase subunit I | -0.08 | 1 |
| lmo0531 | LMRG_00213 | hypothetical protein | -0.08 | 1 |
| lmo1843 | LMRG_00990 | LSU rRNA pseudouridine(1911/1915/1917) synthase (EC 5.4.99.23) | -0.08 | 1 |
| lmo1899 | LMRG_01046 | DinG family ATP-dependent helicase YoaA | -0.08 | 1 |
| lmo2001 | LMRG_01149 | PTS system, mannose-specific IIC component | -0.08 | 1 |
| lmo2743 | LMRG_01953 | Transaldolase (EC 2.2.1.2) | -0.08 | 1 |
| lmo0653 | LMRG_00340 | hypothetical protein | -0.07 | 1 |
| lmo1273 | LMRG_00723 | Ribonuclease HII (EC 3.1.26.4) | -0.07 | 1 |
| lmo2081 | LMRG_01232 | Fluoride ion transporter CrcB | -0.07 | 1 |
| lmo2353 | LMRG_01490 | Sodium/hydrogen exchanger family protein | -0.07 | 1 |
| lmot36 | LMRG_05036 | tRNA-Lys-TTT | -0.07 | 1 |
| lmo0699 | LMRG_00388 | Flagellar motor switch protein FliM | -0.06 | 1 |
| lmo0788 | LMRG_00476 | Activator of (R)-2-hydroxyglutaryl-CoA dehydratase | -0.06 | 1 |
| lmo1376 | LMRG_00828 | 6-phosphogluconate dehydrogenase, decarboxylating (EC 1.1.1.44) | -0.06 | 1 |
| lmo1700 | LMRG_02774 | hypothetical protein | -0.06 | 1 |
| lmo1737 | LMRG_02534 | Glycerol dehydrogenase (EC 1.1.1.6) | -0.06 | 1 |
| lmo2453 | LMRG_01795 | Hypothetical protein formerly called MenX | -0.06 | 1 |
| lmo2599 | LMRG_02143 | Transmembrane component of general energizing module of ECF transporters | -0.06 | 1 |
| lmo2674 | LMRG_02219 | Ribose-5-phosphate isomerase B (EC 5.3.1.6) | -0.06 | 1 |
| lmo2771 | LMRG_01924 | 6-phospho-beta-glucosidase (EC 3.2.1.86) | -0.06 | 1 |
| lmo0240 | LMRG_02666 | Mini-ribonuclease III | -0.05 | 1 |
| lmo0739 | LMRG_00427 | 6-phospho-beta-glucosidase (EC 3.2.1.86) | -0.05 | 1 |
| lmo0878 | LMRG_02302 | Oxidoreductase, aldo/keto reductase family | -0.05 | 1 |
| lmo1506 | LMRG_01464 | ABC transporter, permease protein | -0.05 | 1 |
| lmo0009 | LMRG_02437 | Spermidine N1-acetyltransferase (EC 2.3.1.57) | -0.04 | 1 |
| lmo0622 | LMRG_00305 | hypothetical protein | -0.04 | 1 |
| lmo0650 | LMRG_00337 | phage infection protein | -0.04 | 1 |
| lmo1127 | LMRG_00569 | hypothetical protein | -0.04 | 1 |
| lmo1566 | LMRG_01401 | Isocitrate dehydrogenase [NADP] (EC 1.1.1.42) | -0.04 | 1 |
| lmo1936 | LMRG_01083 | Glycerol-3-phosphate dehydrogenase [NAD(P)+] (EC 1.1.1.94) | -0.04 | 1 |
| lmo1981 | LMRG_01129 | hypothetical protein | -0.04 | 1 |
| lmo2238 | LMRG_01594 | Inner membrane transport protein YdiM | -0.04 | 1 |
| lmo2815 | LMRG_01883 | Oxidoreductase, short-chain dehydrogenase/reductase family | -0.04 | 1 |
| lmo0056 | LMRG_02486 | 6 kDa early secretory antigenic target ESAT-6 (EsxA) | -0.03 | 1 |
| lmo0290 | LMRG_02581 | Two-component system YycFG regulatory protein YycI | -0.03 | 1 |
| lmo1351 | LMRG_00801 | Rhodanese-like domain protein | -0.03 | 1 |
| lmo1690 | LMRG_02764 | Uncharacterized membrane protein YfhP | -0.03 | 1 |
| lmo0051 | LMRG_02480 | Accessory gene regulator protein A | -0.02 | 1 |
| lmo0099 | LMRG_02348 | Putative regulator of the mannose operon, ManO | -0.02 | 1 |
| lmo0525 | LMRG_00206 | hypothetical protein | -0.02 | 1 |
| lmo1153 | LMRG_00596 | Propanediol dehydratase large subunit (EC 4.2.1.28) | -0.02 | 1 |
| lmo1886 | LMRG_01033 | Thermostable carboxypeptidase 1 (EC 3.4.17.19) | -0.02 | 1 |
| lmo2246 | LMRG_01586 | DNA alkylation repair enzyme | -0.02 | 1 |
| lmo2336 | LMRG_01507 | 1-phosphofructokinase (EC 2.7.1.56) | -0.02 | 1 |
| lmo1095 | LMRG_00557 | PTS system, IIB component | -0.01 | 1 |
| lmo1577 | LMRG_01390 | FIG002379: metal-dependent hydrolase | -0.01 | 1 |
| lmo1628 | LMRG_01338 | Tryptophan synthase beta chain (EC 4.2.1.20) | -0.01 | 1 |
| lmo1715 | LMRG_02555 | SAM-dependent methyltransferase | -0.01 | 1 |
| lmo2248 | LMRG_01583 | UPF0111 protein YkaA, likely to be a phosphate transport regulator | -0.01 | 1 |
| lmo2757 | LMRG_01939 | ATP-dependent DNA helicase RecQ | -0.01 | 1 |
| lmo0002 | LMRG_02430 | DNA polymerase III beta subunit (EC 2.7.7.7) | 0 | 1 |
| lmo0005 | LMRG_02433 | DNA recombination and repair protein RecF | 0 | 1 |
| lmo0010 | LMRG_02439 | Mevalonate kinase (EC 2.7.1.36) | 0 | 1 |
| lmo0012 | LMRG_02441 | Phosphomevalonate kinase (EC 2.7.4.2) | 0 | 1 |
| lmo0021 | LMRG_02450 | PTS system, IIA component | 0 | 1 |
| lmo0022 | LMRG_02451 | PTS system, mannose/fructose/sorbose family, IIB component | 0 | 1 |
| lmo0023 | LMRG_02452 | PTS system, mannose/fructose/sorbose family, IIC component | 0 | 1 |
| lmo0041 | LMRG_02470 | Transcriptional regulator, RpiR family | 0 | 1 |
| lmo0046 | LMRG_02475 | SSU ribosomal protein S18p @ SSU ribosomal protein S18p, zinc-independent | 0 | 1 |
| lmo0049 | LMRG_02478 | Accessory gene regulator protein D | 0 | 1 |
| lmo0059 | LMRG_02489 | Putative secretion accessory protein EsaB/YukD | 0 | 1 |
| lmo0078 | LMRG_02329 | Glyoxylate reductase (EC 1.1.1.79) @ Glyoxylate reductase (EC 1.1.1.26) @ Hydroxypyruvate reductase (EC 1.1.1.81) | 0 | 1 |
| lmo0088 | LMRG_02337 | ATP synthase F0 sector subunit c (EC 3.6.3.14) | 0 | 1 |
| lmo0102 | LMRG_02351 | hypothetical protein | 0 | 1 |
| lmo0114 | LMRG_02363 | repressor protein | 0 | 1 |
| lmo0115 | LMRG_02364 | Listeria protein LmaD, associated with virulence | 0 | 1 |
| lmo0136 | LMRG_02385 | Oligopeptide ABC transporter, permease protein OppB (TC 3.A.1.5.1) | 0 | 1 |
| lmo0137 | LMRG_02386 | Oligopeptide ABC transporter, permease protein OppC (TC 3.A.1.5.1) | 0 | 1 |
| lmo0138 | LMRG_02387 | hypothetical protein | 0 | 1 |
| lmo0197 | LMRG_02619 | DNA-binding protein SpoVG | 0 | 1 |
| lmo0208 | LMRG_02630 | UPF0145 protein YbjQ | 0 | 1 |
| lmo0213 | LMRG_02635 | Peptidyl-tRNA hydrolase (EC 3.1.1.29) | 0 | 1 |
| lmo0217 | LMRG_02639 | Cell division protein DivIC (FtsB), stabilizes FtsL against RasP cleavage | 0 | 1 |
| lmo0226 | LMRG_02838 | 2-amino-4-hydroxy-6-hydroxymethyldihydropteridine pyrophosphokinase (EC 2.7.6.3) | 0 | 1 |
| lmo0229 | LMRG_02677 | Transcriptional regulator CtsR | 0 | 1 |
| lmo0237 | LMRG_02669 | Glutamyl-tRNA synthetase (EC 6.1.1.17) @ Glutamyl-tRNA(Gln) synthetase (EC 6.1.1.24) | 0 | 1 |
| lmo0238 | LMRG_02938 | Serine acetyltransferase (EC 2.3.1.30) | 0 | 1 |
| lmo0245 | LMRG_02661 | Protein translocase subunit SecE | 0 | 1 |
| lmo0249 | LMRG_02657 | LSU ribosomal protein L1p (L10Ae) | 0 | 1 |
| lmo0251 | LMRG_02655 | LSU ribosomal protein L7p/L12p (P1/P2) | 0 | 1 |
| lmo0259 | LMRG_02650 | DNA-directed RNA polymerase beta' subunit (EC 2.7.7.6) | 0 | 1 |
| lmo0287 | LMRG_02584 | Two-component response regulator SA14-24 | 0 | 1 |
| lmo0293 | LMRG_02578 | 23S rRNA (pseudouridine(1915)-N(3))-methyltransferase (EC 2.1.1.177) | 0 | 1 |
| lmo0297 | LMRG_02571 | Transcriptional antiterminator of lichenan operon, BglG family / PTS system, cellobiose-specific IIA component (EC 2.7.1.205) | 0 | 1 |
| lmo0299 | LMRG_02569 | PTS system, cellobiose-specific IIB component (EC 2.7.1.205) | 0 | 1 |
| lmo0306 | LMRG_00003 | hypothetical protein | 0 | 1 |
| lmo0308 | LMRG_00005 | hypothetical protein | 0 | 1 |
| lmo0315 | LMRG_00008 | Thiaminase II (EC 3.5.99.2) involved in salvage of thiamin pyrimidine moiety | 0 | 1 |
| lmo0318 | LMRG_00011 | Thiamin-phosphate pyrophosphorylase (EC 2.5.1.3) | 0 | 1 |
| lmo0324 | LMRG_00016 | hypothetical protein | 0 | 1 |
| lmo0326 | LMRG_00019 | FIG011400: transcriptional activator of Lmo0327 homolog | 0 | 1 |
| lmo0345 | LMRG_00036 | Ribose-5-phosphate isomerase B (EC 5.3.1.6) | 0 | 1 |
| lmo0349 | LMRG_00040 | hypothetical protein | 0 | 1 |
| lmo0357 | LMRG_00048 | PTS system, IIA component | 0 | 1 |
| lmo0360 | LMRG_00051 | Transcriptional regulator, DeoR family | 0 | 1 |
| lmo0362 | LMRG_00054 | Twin-arginine translocation protein TatAy | 0 | 1 |
| lmo0399 | LMRG_00092 | PTS system, IIB component | 0 | 1 |
| lmo0406 | LMRG_00099 | Possible glyoxylase family protein (Lactoylglutathione lyase) (EC 4.4.1.5) | 0 | 1 |
| lmo0419 | LMRG_00111 | Membrane protein | 0 | 1 |
| lmo0442 | LMRG_00134 | hypothetical protein | 0 | 1 |
| lmo0446 | LMRG_00138 | Penicillin V amidase (Pva) not involved in bile hydrolysis | 0 | 1 |
| lmo0448a | LMRG_02888 | Probable glutamate/gamma-aminobutyrate antiporter | 0 | 1 |
| lmo0486 | LMRG_00167 | LSU ribosomal protein L32p @ LSU ribosomal protein L32p, zinc-independent | 0 | 1 |
| lmo0499 | LMRG_00180 | Ribulose-phosphate 3-epimerase (EC 5.1.3.1) | 0 | 1 |
| lmo0500 | LMRG_00181 | Transaldolase (EC 2.2.1.2) | 0 | 1 |
| lmo0504 | LMRG_00185 | hypothetical protein | 0 | 1 |
| lmo0532 | LMRG_00214 | hypothetical protein | 0 | 1 |
| lmo0533 | LMRG_00215 | ACT domain protein CAC_0478 | 0 | 1 |
| lmo0548 | LMRG_00230 | hypothetical protein | 0 | 1 |
| lmo0550 | LMRG_00232 | Putative peptidoglycan bound protein (LPXTG motif) Lmo0550 homolog | 0 | 1 |
| lmo0570 | LMRG_00252 | Histidinol-phosphatase (EC 3.1.3.15) | 0 | 1 |
| lmo0571 | LMRG_00253 | Methylated-DNA--protein-cysteine methyltransferase | 0 | 1 |
| lmo0579 | LMRG_00261 | bacterial seryl-tRNA synthetase related | 0 | 1 |
| lmo0597 | LMRG_00280 | cyclic nucleotide-binding protein | 0 | 1 |
| lmo0619 | LMRG_00302 | hypothetical protein | 0 | 1 |
| lmo0655 | LMRG_00342 | Serine/threonine protein phosphatase (EC 3.1.3.16) | 0 | 1 |
| lmo0658 | LMRG_00345 | endonuclease III domain protein | 0 | 1 |
| lmo0680 | LMRG_00368 | Flagellar biosynthesis protein FlhA | 0 | 1 |
| lmo0684 | LMRG_00372 | hypothetical protein | 0 | 1 |
| lmo0698 | LMRG_00387 | Flagellar motor switch protein FliN | 0 | 1 |
| lmo0709 | LMRG_00398 | hypothetical protein | 0 | 1 |
| lmo0726 | N/A | Hypothetical CDS | 0 | 1 |
| lmo0735 | LMRG_00423 | D-allulose-6-phosphate 3-epimerase (EC 5.1.3.-) | 0 | 1 |
| lmo0736 | LMRG_00424 | D-allose-6-phosphate isomerase (EC 5.3.1.-) @ Ribose-5-phosphate isomerase B (EC 5.3.1.6) | 0 | 1 |
| lmo0737 | LMRG_00425 | hypothetical protein | 0 | 1 |
| lmo0747 | LMRG_00435 | hypothetical protein | 0 | 1 |
| lmo0748 | LMRG_00436 | hypothetical protein | 0 | 1 |
| lmo0761 | LMRG_00449 | Flavin reductase like domain protein in BltB locus | 0 | 1 |
| lmo0780 | LMRG_00468 | hypothetical protein | 0 | 1 |
| lmo0802 | LMRG_02795 | GTP pyrophosphokinase (EC 2.7.6.5) | 0 | 1 |
| lmo0812 | LMRG_02785 | GTP pyrophosphokinase (EC 2.7.6.5) | 0 | 1 |
| lmo0817 | LMRG_02242 | PhnB protein | 0 | 1 |
| lmo0850 | LMRG_02273 | hypothetical protein | 0 | 1 |
| lmo0853 | LMRG_02276 | small multidrug resistance family (SMR) protein | 0 | 1 |
| lmo0855 | LMRG_02278 | D-alanine--D-alanine ligase (EC 6.3.2.4) | 0 | 1 |
| lmo0870 | LMRG_02294 | hypothetical protein | 0 | 1 |
| lmo0873 | LMRG_02297 | Putative transcriptional antiterminator, BglG family / PTS system, IIA component | 0 | 1 |
| lmo0874 | LMRG_02298 | PTS system, beta-glucoside-specific, IIA component | 0 | 1 |
| lmo0875 | LMRG_02299 | PTS system, IIB component | 0 | 1 |
| lmo0885 | LMRG_02309 | Holo-[acyl-carrier-protein] synthase (EC 2.7.8.7) | 0 | 1 |
| lmo0887 | LMRG_02311 | Programmed cell death antitoxin YdcD | 0 | 1 |
| lmo0914 | LMRG_02014 | PTS system, cellobiose-specific IIB component (EC 2.7.1.205) | 0 | 1 |
| lmo0968 | LMRG_02067 | NAD kinase (EC 2.7.1.23) | 0 | 1 |
| lmo0986 | LMRG_02086 | Efflux ABC transporter, ATP-binding protein | 0 | 1 |
| lmo1002 | LMRG_02102 | Phosphotransferase system, phosphocarrier protein HPr | 0 | 1 |
| lmo1012 | LMRG_02112 | N-acetyl-L,L-diaminopimelate deacetylase (EC 3.5.1.47) | 0 | 1 |
| lmo1020 | LMRG_02120 | Membrane protein LiaF(VraT), specific inhibitor of LiaRS(VraRS) signaling pathway | 0 | 1 |
| lmo1027 | LMRG_02127 | Ribonuclease J1 (endonuclease and 5' exonuclease) | 0 | 1 |
| lmo1028 | LMRG_02128 | Protein of unknown function DUF1447 | 0 | 1 |
| lmo1038 | LMRG_00499 | Molybdenum cofactor guanylyltransferase (EC 2.7.7.77) | 0 | 1 |
| lmo1043 | LMRG_00504 | Molybdopterin-guanine dinucleotide biosynthesis protein MobB | 0 | 1 |
| lmo1044 | LMRG_00505 | Molybdopterin synthase catalytic subunit MoaE (EC 2.8.1.12) | 0 | 1 |
| lmo1045 | LMRG_00506 | Molybdopterin synthase sulfur carrier subunit | 0 | 1 |
| lmo1074 | LMRG_00536 | Teichoic acid translocation permease protein TagG | 0 | 1 |
| lmo1078 | LMRG_00540 | UTP--glucose-1-phosphate uridylyltransferase (EC 2.7.7.9) | 0 | 1 |
| lmo1085 | LMRG_00547 | CDP-ribitol:poly(ribitol phosphate) ribitol phosphotransferase | 0 | 1 |
| lmo1086 | LMRG_00548 | Sugar-phosphate cytidylyltransferase | 0 | 1 |
| lmo1087 | LMRG_00549 | Xylitol dehydrogenase | 0 | 1 |
| lmo1090 | LMRG_00552 | Beta-1,3-glucosyltransferase | 0 | 1 |
| lmo1091 | LMRG_00553 | Beta-1,3-glucosyltransferase | 0 | 1 |
| lmo1121 | LMRG_00563 | hypothetical protein | 0 | 1 |
| lmo1122 | LMRG_00564 | hypothetical protein | 0 | 1 |
| lmo1124 | LMRG_00566 | hypothetical protein | 0 | 1 |
| lmo1130 | LMRG_00572 | hypothetical protein | 0 | 1 |
| lmo1144 | LMRG_00587 | Propanediol utilization polyhedral body protein PduU | 0 | 1 |
| lmo1145 | LMRG_00588 | Propanediol utilization protein PduV | 0 | 1 |
| lmo1146 | LMRG_00589 | Predicted alpha-ribazole-5-phosphate synthase CblS for cobalamin biosynthesis | 0 | 1 |
| lmo1147 | LMRG_00590 | Adenosylcobinamide kinase (EC 2.7.1.156) / Adenosylcobinamide-phosphate guanylyltransferase (EC 2.7.7.62) | 0 | 1 |
| lmo1149 | LMRG_00592 | Alpha-ribazole-5'-phosphate phosphatase (EC 3.1.3.73) | 0 | 1 |
| lmo1151 | LMRG_00594 | Propanediol utilization polyhedral body protein PduA | 0 | 1 |
| lmo1156 | LMRG_00599 | Propanediol dehydratase reactivation factor large subunit | 0 | 1 |
| lmo1163 | LMRG_00606 | Propanediol utilization polyhedral body protein PduN | 0 | 1 |
| lmo1166 | LMRG_00609 | Propanol dehydrogenase [NAD+] | 0 | 1 |
| lmo1169 | LMRG_00612 | L-threonine 3-O-phosphate decarboxylase (EC 4.1.1.81) | 0 | 1 |
| lmo1174 | LMRG_00620 | Ethanolamine utilization protein EutA | 0 | 1 |
| lmo1190 | LMRG_00636 | Substrate-specific component CblT of predicted B12-regulated ECF transporter for dimethylbenzimidazole | 0 | 1 |
| lmo1192 | LMRG_00638 | Adenosylcobinamide-phosphate synthase (EC 6.3.1.10) | 0 | 1 |
| lmo1196 | LMRG_00642 | Cobalt-precorrin-6B C15-methyltransferase [decarboxylating] (EC 2.1.1.196) | 0 | 1 |
| lmo1197 | LMRG_00643 | Cobalt-precorrin-4 C(11)-methyltransferase (EC 2.1.1.271) | 0 | 1 |
| lmo1199 | LMRG_00645 | Cobalt-precorrin-3 C(17)-methyltransferase (EC 2.1.1.272) | 0 | 1 |
| lmo1200 | LMRG_00646 | Cobalt-precorrin-6A reductase (EC 1.3.1.106) | 0 | 1 |
| lmo1203 | LMRG_00649 | Cobalt-precorrin-2 C(20)-methyltransferase (EC 2.1.1.151) | 0 | 1 |
| lmo1205 | LMRG_00651 | Cobalt ECF transporter, additional substrate-binding protein CbiN | 0 | 1 |
| lmo1206 | LMRG_00652 | Cobalt ECF transporter, transmembrane component of energizing module CbiQ | 0 | 1 |
| lmo1207 | LMRG_00653 | Cobalt ECF transporter, ATPase component of energizing module CbiO | 0 | 1 |
| lmo1212 | LMRG_00658 | hypothetical protein | 0 | 1 |
| lmo1222 | LMRG_00668 | Phenylalanyl-tRNA synthetase beta chain (EC 6.1.1.20) | 0 | 1 |
| lmo1228 | LMRG_00674 | Ribonuclease HIII (EC 3.1.26.4) | 0 | 1 |
| lmo1229 | LMRG_00675 | Z-ring-associated protein | 0 | 1 |
| lmo1237 | LMRG_00683 | Glutamate racemase (EC 5.1.1.3) | 0 | 1 |
| lmo1239 | LMRG_00685 | Nucleoside 5-triphosphatase RdgB (dHAPTP, dITP, XTP-specific) (EC 3.6.1.66) | 0 | 1 |
| lmo1248 | LMRG_00694 | MutT/Nudix family protein | 0 | 1 |
| lmo1249 | LMRG_00695 | hypothetical protein | 0 | 1 |
| lmo1263 | LMRG_00712 | DNA-binding protein | 0 | 1 |
| lmo1272 | LMRG_00722 | LSU ribosomal maturation GTPase RbgA (B. subtilis YlqF) | 0 | 1 |
| lmo1275 | LMRG_00725 | DNA topoisomerase I (EC 5.99.1.2) | 0 | 1 |
| lmo1277 | LMRG_00727 | Site-specific tyrosine recombinase XerC | 0 | 1 |
| lmo1282 | LMRG_00732 | Uncharacterized protein YneQ | 0 | 1 |
| lmo1284 | LMRG_00734 | Acyl-phosphate:glycerol-3-phosphate O-acyltransferase PlsY (EC 2.3.1.n3) | 0 | 1 |
| lmo1287 | LMRG_00737 | DNA topoisomerase IV subunit A (EC 5.99.1.3) | 0 | 1 |
| lmo1306 | LMRG_00756 | UPF0154 protein YneF | 0 | 1 |
| lmo1312 | LMRG_00762 | hypothetical protein | 0 | 1 |
| lmo1313 | LMRG_00763 | Uridylate kinase (EC 2.7.4.22) | 0 | 1 |
| lmo1314 | LMRG_00764 | Ribosome recycling factor | 0 | 1 |
| lmo1322 | LMRG_00772 | Transcription termination protein NusA | 0 | 1 |
| lmo1323 | LMRG_00773 | COG2740: Predicted nucleic-acid-binding protein implicated in transcription termination | 0 | 1 |
| lmo1329 | LMRG_00779 | FMN adenylyltransferase (EC 2.7.7.2) / Riboflavin kinase (EC 2.7.1.26) | 0 | 1 |
| lmo1330 | LMRG_00780 | SSU ribosomal protein S15p (S13e) | 0 | 1 |
| lmo1333 | LMRG_00783 | hypothetical protein | 0 | 1 |
| lmo1341 | LMRG_00791 | Late competence protein ComGG, FIG007920 | 0 | 1 |
| lmo1343 | LMRG_00793 | Late competence protein ComGE, FIG015564 | 0 | 1 |
| lmo1344 | LMRG_00794 | Late competence protein ComGD, access of DNA to ComEA, FIG012777 | 0 | 1 |
| lmo1346 | LMRG_00796 | Late competence protein ComGB, access of DNA to ComEA | 0 | 1 |
| lmo1347 | LMRG_00797 | Late competence protein ComGA, access of DNA to ComEA | 0 | 1 |
| lmo1352 | LMRG_00802 | hypothetical protein | 0 | 1 |
| lmo1356 | LMRG_00806 | Biotin carboxyl carrier protein of acetyl-CoA carboxylase | 0 | 1 |
| lmo1359 | LMRG_00809 | Transcription termination protein NusB | 0 | 1 |
| lmo1362 | LMRG_00812 | Exodeoxyribonuclease VII small subunit (EC 3.1.11.6) | 0 | 1 |
| lmo1364 | LMRG_00814 | Cold shock protein of CSP family | 0 | 1 |
| lmo1375 | LMRG_00826 | Peptidase T (EC 3.4.11.4) | 0 | 1 |
| lmo1382 | LMRG_00834 | FIG011684: hypothetical protein | 0 | 1 |
| lmo1398 | LMRG_00850 | RecA protein | 0 | 1 |
| lmo1402 | LMRG_00854 | YMCA protein | 0 | 1 |
| lmo1415 | LMRG_00867 | Hydroxymethylglutaryl-CoA synthase (EC 2.3.3.10) | 0 | 1 |
| lmo1418 | LMRG_00870 | Alkaline phosphodiesterase I (EC 3.1.4.1) / Nucleotide pyrophosphatase (EC 3.6.1.9) | 0 | 1 |
| lmo1420 | LMRG_00872 | UDP-N-acetylenolpyruvoylglucosamine reductase (EC 1.3.1.98) | 0 | 1 |
| lmo1436 | LMRG_00888 | Aspartokinase (EC 2.7.2.4) | 0 | 1 |
| lmo1437 | LMRG_00889 | Aspartate-semialdehyde dehydrogenase (EC 1.2.1.11) | 0 | 1 |
| lmo1441 | LMRG_00893 | (E)-4-hydroxy-3-methylbut-2-enyl-diphosphate synthase (flavodoxin) (EC 1.17.7.3) | 0 | 1 |
| lmo1453 | LMRG_00905 | tRNA (adenine(22)-N(1))-methyltransferase (EC 2.1.1.217) | 0 | 1 |
| lmo1454 | LMRG_00906 | RNA polymerase sigma factor RpoD | 0 | 1 |
| lmo1459 | LMRG_00911 | Glycyl-tRNA synthetase alpha chain (EC 6.1.1.14) | 0 | 1 |
| lmo1465 | LMRG_00917 | Metal-dependent hydrolase YbeY, involved in rRNA and/or ribosome maturation and assembly | 0 | 1 |
| lmo1472 | LMRG_00925 | Chaperone protein DnaJ | 0 | 1 |
| lmo1474 | LMRG_00927 | Heat shock protein GrpE | 0 | 1 |
| lmo1480 | LMRG_00933 | SSU ribosomal protein S20p | 0 | 1 |
| lmo1481 | LMRG_00934 | DNA polymerase III delta subunit (EC 2.7.7.7) | 0 | 1 |
| lmo1488 | LMRG_00941 | Nicotinate-nucleotide adenylyltransferase (EC 2.7.7.18) | 0 | 1 |
| lmo1490 | LMRG_00943 | Shikimate 5-dehydrogenase I alpha (EC 1.1.1.25) | 0 | 1 |
| lmo1491 | LMRG_00944 | GTP-binding protein YqeH, required for biogenesis of 30S ribosome subunit | 0 | 1 |
| lmo1492 | LMRG_00945 | FIG001553: Hydrolase, HAD subfamily IIIA | 0 | 1 |
| lmo1496 | LMRG_01474 | Transcription elongation factor GreA | 0 | 1 |
| lmo1513 | LMRG_01457 | Cysteine desulfurase (EC 2.8.1.7) | 0 | 1 |
| lmo1520 | LMRG_01450 | Histidyl-tRNA synthetase (EC 6.1.1.21) | 0 | 1 |
| lmo1529 | LMRG_01441 | Protein translocase subunit YajC | 0 | 1 |
| lmo1532 | LMRG_01438 | Holliday junction ATP-dependent DNA helicase RuvB (EC 3.6.4.12) | 0 | 1 |
| lmo1537 | LMRG_01433 | GTP-binding protein Obg | 0 | 1 |
| lmo1540 | LMRG_01430 | LSU ribosomal protein L27p | 0 | 1 |
| lmo1541 | LMRG_01429 | FIG139598: Potential ribosomal protein | 0 | 1 |
| lmo1543 | LMRG_01427 | Ribonuclease G | 0 | 1 |
| lmo1544 | LMRG_01426 | Septum site-determining protein MinD | 0 | 1 |
| lmo1545 | LMRG_01425 | Septum site-determining protein MinC | 0 | 1 |
| lmo1546 | LMRG_01424 | Rod shape-determining protein MreD | 0 | 1 |
| lmo1549 | LMRG_01420 | UPF0758 family protein | 0 | 1 |
| lmo1551 | LMRG_01417 | Dihydrofolate synthase (EC 6.3.2.12) @ Folylpolyglutamate synthase (EC 6.3.2.17) | 0 | 1 |
| lmo1555 | LMRG_01413 | Uroporphyrinogen-III synthase (EC 4.2.1.75) | 0 | 1 |
| lmo1556 | LMRG_01412 | Porphobilinogen deaminase (EC 2.5.1.61) | 0 | 1 |
| lmo1558 | LMRG_01409 | GTP-binding protein EngB | 0 | 1 |
| lmo1560 | LMRG_01407 | Helicase loader DnaI | 0 | 1 |
| lmo1561 | LMRG_01406 | Helicase loader DnaB | 0 | 1 |
| lmo1562 | LMRG_01405 | Ribonucleotide reductase transcriptional regulator NrdR | 0 | 1 |
| lmo1568 | LMRG_01399 | UPF0756 membrane protein YtwI | 0 | 1 |
| lmo1573 | LMRG_01394 | Acetyl-coenzyme A carboxyl transferase beta chain (EC 6.4.1.2) | 0 | 1 |
| lmo1594 | LMRG_01373 | Septation ring formation regulator EzrA | 0 | 1 |
| lmo1596 | LMRG_01371 | SSU ribosomal protein S4p (S9e) @ SSU ribosomal protein S4p (S9e), zinc-independent | 0 | 1 |
| lmo1598 | LMRG_01369 | Tyrosyl-tRNA synthetase (EC 6.1.1.1) | 0 | 1 |
| lmo1602 | LMRG_01365 | UPF0478 protein YtxG | 0 | 1 |
| lmo1610 | LMRG_01356 | hypothetical protein | 0 | 1 |
| lmo1629 | LMRG_01337 | Phosphoribosylanthranilate isomerase (EC 5.3.1.24) | 0 | 1 |
| lmo1630 | LMRG_01336 | Indole-3-glycerol phosphate synthase (EC 4.1.1.48) | 0 | 1 |
| lmo1632 | LMRG_01334 | Anthranilate synthase, amidotransferase component (EC 4.1.3.27) | 0 | 1 |
| lmo1635 | LMRG_01331 | PhnB protein | 0 | 1 |
| lmo1640 | LMRG_01326 | hypothetical protein | 0 | 1 |
| lmo1647 | LMRG_01319 | Acyl-ACP:1-acyl-sn-glycerol-3-phosphate acyltransferase (EC 2.3.1.n4) | 0 | 1 |
| lmo1654 | LMRG_01313 | Putative cellsurface protein | 0 | 1 |
| lmo1656 | LMRG_01311 | hypothetical protein | 0 | 1 |
| lmo1657 | LMRG_01310 | Translation elongation factor Ts | 0 | 1 |
| lmo1658 | LMRG_01309 | SSU ribosomal protein S2p (SAe) | 0 | 1 |
| lmo1664 | LMRG_01303 | S-adenosylmethionine synthetase (EC 2.5.1.6) | 0 | 1 |
| lmo1668 | LMRG_01299 | Uncharacterized protein YtmB | 0 | 1 |
| lmo1669 | LMRG_01298 | 8-oxo-dGTPase Bsu YtkD / 8-oxo-GTPase Bsu YtkD | 0 | 1 |
| lmo1670 | LMRG_01297 | Membrane protein insertion efficiency factor YidD | 0 | 1 |
| lmo1719 | LMRG_02552 | PTS system, beta-glucoside-specific IIA component | 0 | 1 |
| lmo1735 | LMRG_02536 | Glutamate synthase operon transcriptional activator GltC, LysR family | 0 | 1 |
| lmo1740 | LMRG_02531 | ABC transporter, permease protein (cluster 3, basic aa/glutamine/opines) | 0 | 1 |
| lmo1754 | LMRG_02517 | Aspartyl-tRNA(Asn) amidotransferase subunit B (EC 6.3.5.6) @ Glutamyl-tRNA(Gln) amidotransferase subunit B (EC 6.3.5.7) | 0 | 1 |
| lmo1756 | LMRG_02515 | Aspartyl-tRNA(Asn) amidotransferase subunit C (EC 6.3.5.6) @ Glutamyl-tRNA(Gln) amidotransferase subunit C (EC 6.3.5.7) | 0 | 1 |
| lmo1772 | LMRG_02499 | Phosphoribosylaminoimidazole-succinocarboxamide synthase (EC 6.3.2.6) | 0 | 1 |
| lmo1783 | LMRG_02829 | LSU ribosomal protein L20p | 0 | 1 |
| lmo1787 | LMRG_02811 | LSU ribosomal protein L19p | 0 | 1 |
| lmo1793 | LMRG_02817 | 16S rRNA processing protein RimM | 0 | 1 |
| lmo1796 | LMRG_02820 | KH domain RNA binding protein YlqC | 0 | 1 |
| lmo1797 | LMRG_02821 | SSU ribosomal protein S16p | 0 | 1 |
| lmo1801 | LMRG_00948 | Signal recognition particle protein Ffh | 0 | 1 |
| lmo1808 | LMRG_00955 | Malonyl CoA-acyl carrier protein transacylase (EC 2.3.1.39) | 0 | 1 |
| lmo1816 | LMRG_00963 | LSU ribosomal protein L28p @ LSU ribosomal protein L28p, zinc-independent | 0 | 1 |
| lmo1817 | LMRG_00964 | Thiamin pyrophosphokinase (EC 2.7.6.2) | 0 | 1 |
| lmo1820 | LMRG_00967 | Serine/threonine protein kinase PrkC, regulator of stationary phase | 0 | 1 |
| lmo1823 | LMRG_00970 | Methionyl-tRNA formyltransferase (EC 2.1.2.9) | 0 | 1 |
| lmo1825 | LMRG_00972 | Phosphopantothenoylcysteine decarboxylase (EC 4.1.1.36) / Phosphopantothenoylcysteine synthetase (EC 6.3.2.5) | 0 | 1 |
| lmo1827 | LMRG_00974 | Guanylate kinase (EC 2.7.4.8) | 0 | 1 |
| lmo1833 | LMRG_00980 | Dihydroorotate dehydrogenase (NAD(+)), catalytic subunit (EC 1.3.1.14) | 0 | 1 |
| lmo1836 | LMRG_00983 | Carbamoyl-phosphate synthase small chain (EC 6.3.5.5) | 0 | 1 |
| lmo1841 | LMRG_00988 | hypothetical protein | 0 | 1 |
| lmo1873 | LMRG_01020 | Dihydrofolate reductase (EC 1.5.1.3) | 0 | 1 |
| lmo1874 | LMRG_01021 | Thymidylate synthase (EC 2.1.1.45) | 0 | 1 |
| lmo1879 | LMRG_01026 | Cold shock protein of CSP family => CspD (naming convention as in B.subtlis) | 0 | 1 |
| lmo1880 | LMRG_01027 | Ribonuclease HI, Bacillus nonfunctional homolog | 0 | 1 |
| lmo1882 | LMRG_01029 | SSU ribosomal protein S14p (S29e) @ SSU ribosomal protein S14p (S29e), zinc-independent | 0 | 1 |
| lmo1885 | LMRG_01032 | Xanthine phosphoribosyltransferase (EC 2.4.2.22) | 0 | 1 |
| lmo1888 | LMRG_01035 | Cell division protein GpsB, coordinates the switch between cylindrical and septal cell wall synthesis by re-localization of PBP1 | 0 | 1 |
| lmo1891 | LMRG_01038 | RecU Holliday junction resolvase | 0 | 1 |
| lmo1897 | LMRG_01044 | Aspartate aminotransferase (EC 2.6.1.1) | 0 | 1 |
| lmo1903 | LMRG_01050 | bacteriocin transport accessory protein | 0 | 1 |
| lmo1904 | LMRG_01051 | Biotin operon repressor / Biotin--protein ligase (EC 6.3.4.9)(EC 6.3.4.10)(EC 6.3.4.11)(EC 6.3.4.15) | 0 | 1 |
| lmo1905 | LMRG_01052 | CCA tRNA nucleotidyltransferase (EC 2.7.7.72) | 0 | 1 |
| lmo1934 | LMRG_01081 | DNA-binding protein HBsu | 0 | 1 |
| lmo1950 | LMRG_01097 | Segregation and condensation protein B | 0 | 1 |
| lmo1951 | LMRG_01098 | Segregation and condensation protein A | 0 | 1 |
| lmo1969 | LMRG_01116 | 4-hydroxy-2-oxoglutarate aldolase (EC 4.1.3.16) @ 2-dehydro-3-deoxyphosphogluconate aldolase (EC 4.1.2.14) | 0 | 1 |
| lmo1973 | LMRG_01120 | PTS system, IIA component | 0 | 1 |
| lmo1980 | N/A | hypothetical protein | 0 | 1 |
| lmo2002 | LMRG_01150 | PTS system, mannose-specific IIB component (EC 2.7.1.191) | 0 | 1 |
| lmo2017 | LMRG_01166 | putative membrane-associated phospholipid phosphatase, PAP2 superfamily | 0 | 1 |
| lmo2018 | LMRG_01167 | Diaminopimelate epimerase (EC 5.1.1.7) | 0 | 1 |
| lmo2019 | LMRG_01168 | Isoleucyl-tRNA synthetase (EC 6.1.1.5) | 0 | 1 |
| lmo2022 | LMRG_01171 | Cysteine desulfurase (EC 2.8.1.7) | 0 | 1 |
| lmo2030 | LMRG_01179 | SepF, FtsZ-interacting protein related to cell division | 0 | 1 |
| lmo2035 | LMRG_01184 | UDP-N-acetylglucosamine--N-acetylmuramyl-(pentapeptide) pyrophosphoryl-undecaprenol N-acetylglucosamine transferase (EC 2.4.1.227) | 0 | 1 |
| lmo2040 | LMRG_01189 | Cell division protein FtsL | 0 | 1 |
| lmo2052 | LMRG_01202 | Phosphopantetheine adenylyltransferase (EC 2.7.7.3) | 0 | 1 |
| lmo2053 | LMRG_01203 | 16S rRNA (guanine(966)-N(2))-methyltransferase (EC 2.1.1.171) | 0 | 1 |
| lmo2063 | LMRG_01213 | hypothetical protein | 0 | 1 |
| lmo2066 | LMRG_01216 | FIG019766: hypothetical protein co-occurring with bile hydrolase | 0 | 1 |
| lmo2069 | LMRG_01219 | Heat shock protein 10 kDa family chaperone GroES | 0 | 1 |
| lmo2075 | LMRG_01226 | N(6)-L-threonylcarbamoyladenine synthase (EC 2.3.1.234) | 0 | 1 |
| lmo2077 | LMRG_01228 | tRNA threonylcarbamoyladenosine biosynthesis protein TsaB | 0 | 1 |
| lmo2078 | LMRG_01229 | tRNA threonylcarbamoyladenosine biosynthesis protein TsaE | 0 | 1 |
| lmo2093 | LMRG_01244 | hypothetical protein | 0 | 1 |
| lmo2097 | LMRG_01248 | PTS system, galactitol-specific IIB component (EC 2.7.1.200) | 0 | 1 |
| lmo2100 | LMRG_01251 | Transcriptional regulator of pyridoxine metabolism / Pyridoxamine phosphate aminotransferase (EC 2.6.1.54) | 0 | 1 |
| lmo2102 | LMRG_01253 | Pyridoxal 5'-phosphate synthase (glutamine hydrolyzing), glutaminase subunit (EC 4.3.3.6) | 0 | 1 |
| lmo2104 | LMRG_01257 | Ferrous iron transporter-associated protein FeoA | 0 | 1 |
| lmo2118 | LMRG_01272 | Phosphoglucosamine mutase (EC 5.4.2.10) | 0 | 1 |
| lmo2120 | LMRG_01274 | Diadenylate cyclase spyDAC | 0 | 1 |
| lmo2135 | LMRG_02805 | PTS system, IIC component | 0 | 1 |
| lmo2138 | LMRG_02802 | Transcription antiterminator, BglG family | 0 | 1 |
| lmo2141 | LMRG_02799 | Acetyltransferase, GNAT family | 0 | 1 |
| lmo2145 | LMRG_01687 | Predicted nicotinate-regulated transporter BH3254 | 0 | 1 |
| lmo2152 | LMRG_01680 | Putative thioredoxin | 0 | 1 |
| lmo2153 | LMRG_01679 | Flavodoxin | 0 | 1 |
| lmo2154 | LMRG_01678 | Ribonucleotide reductase of class Ia (aerobic), beta subunit (EC 1.17.4.1) | 0 | 1 |
| lmo2155 | LMRG_01677 | Ribonucleotide reductase of class Ia (aerobic), alpha subunit (EC 1.17.4.1) | 0 | 1 |
| lmo2192 | LMRG_01640 | Oligopeptide ABC transporter, ATP-binding protein OppF (TC 3.A.1.5.1) | 0 | 1 |
| lmo2202 | LMRG_01630 | 3-oxoacyl-[acyl-carrier-protein] synthase, KASIII (EC 2.3.1.180) | 0 | 1 |
| lmo2210 | LMRG_01622 | hypothetical protein | 0 | 1 |
| lmo2211 | LMRG_01621 | Coproporphyrin ferrochelatase (EC 4.99.1.9) | 0 | 1 |
| lmo2212 | LMRG_01620 | Uroporphyrinogen III decarboxylase (EC 4.1.1.37) | 0 | 1 |
| lmo2217 | LMRG_01615 | Hypothetical protein SAV1839 | 0 | 1 |
| lmo2230 | LMRG_01602 | Arsenate reductase (EC 1.20.4.4) thioredoxin-coupled, LMWP family | 0 | 1 |
| lmo2261 | LMRG_01570 | PaaD-like protein (DUF59) involved in Fe-S cluster assembly | 0 | 1 |
| lmo2267 | LMRG_02922 | ATP-dependent helicase/nuclease AddAB, subunit A | 0 | 1 |
| lmo2268 | LMRG_01562 | ATP-dependent helicase/nuclease AddAB, subunit B | 0 | 1 |
| lmo2269 | LMRG_01561 | hypothetical protein | 0 | 1 |
| lmo2281 | LMRG_01551 | gp22 | 0 | 1 |
| lmo2282 | LMRG_01550 | Putative short tail fibre [Bacteriophage A118] | 0 | 1 |
| lmo2304 | LMRG_01528 | Protein gp65 [Bacteriophage A118] | 0 | 1 |
| lmo2308 | LMRG_02474 | Phage recombination protein Bet | 0 | 1 |
| lmo2321 | LMRG_01523 | Protein gp45 [Bacteriophage A118] | 0 | 1 |
| lmo2322 | LMRG_01522 | Protein gp44 [Bacteriophage A118] | 0 | 1 |
| lmo2325 | LMRG_02984 | Phage protein | 0 | 1 |
| lmo2327 | LMRG_01517 | hypothetical protein | 0 | 1 |
| lmo2328 | LMRG_01516 | hypothetical protein | 0 | 1 |
| lmo2329 | LMRG_01514 | Repressor (CI-like) [Bacteriophage A118] | 0 | 1 |
| lmo2333 | LMRG_01510 | Competence transcription factor | 0 | 1 |
| lmo2345 | LMRG_01498 | Bacterial luciferase family protein YtmO, in cluster with L-cystine ABC transporter | 0 | 1 |
| lmo2350 | LMRG_01493 | Uncharacterized N-acetyltransferase YtmI | 0 | 1 |
| lmo2378 | LMRG_02723 | Na(+) H(+) antiporter subunit A | 0 | 1 |
| lmo2379 | LMRG_02724 | Na(+) H(+) antiporter subunit B | 0 | 1 |
| lmo2380 | LMRG_02725 | Na(+) H(+) antiporter subunit C | 0 | 1 |
| lmo2383 | LMRG_02728 | Na(+) H(+) antiporter subunit F | 0 | 1 |
| lmo2385 | LMRG_02730 | 1,4-dihydroxy-2-naphthoyl-CoA hydrolase (EC 3.1.2.28) in menaquinone biosynthesis | 0 | 1 |
| lmo2390 | LMRG_02735 | Thioredoxin reductase (EC 1.8.1.9) | 0 | 1 |
| lmo2391 | LMRG_02736 | oxidoreductase ylbE | 0 | 1 |
| lmo2394 | LMRG_02915 | hypothetical protein | 0 | 1 |
| lmo2397 | LMRG_01851 | NifU family protein | 0 | 1 |
| lmo2399 | LMRG_01849 | CBS-domain-containing protein | 0 | 1 |
| lmo2400 | LMRG_01848 | Acetyltransferase, GNAT family | 0 | 1 |
| lmo2412 | LMRG_01836 | Putative iron-sulfur cluster assembly scaffold protein for SUF system, SufE2 | 0 | 1 |
| lmo2413 | LMRG_01835 | Cysteine desulfurase (EC 2.8.1.7) => SufS | 0 | 1 |
| lmo2415 | LMRG_01833 | Iron-sulfur cluster assembly ATPase protein SufC | 0 | 1 |
| lmo2424 | LMRG_01824 | Thioredoxin | 0 | 1 |
| lmo2426 | LMRG_01822 | FIG138056: a glutathione-dependent thiol reductase | 0 | 1 |
| lmo2432 | LMRG_01816 | hypothetical protein | 0 | 1 |
| lmo2435 | LMRG_01813 | hypothetical protein | 0 | 1 |
| lmo2441 | LMRG_01807 | Transcriptional regulator, YafY family | 0 | 1 |
| lmo2442 | LMRG_01806 | hypothetical protein | 0 | 1 |
| lmo2448 | LMRG_01800 | tmRNA-binding protein SmpB | 0 | 1 |
| lmo2455 | LMRG_01793 | Enolase (EC 4.2.1.11) | 0 | 1 |
| lmo2458 | LMRG_01790 | Phosphoglycerate kinase (EC 2.7.2.3) | 0 | 1 |
| lmo2459 | LMRG_01789 | NAD-dependent glyceraldehyde-3-phosphate dehydrogenase (EC 1.2.1.12) | 0 | 1 |
| lmo2472 | LMRG_01776 | Sporulation transcription regulator WhiA | 0 | 1 |
| lmo2478 | LMRG_01770 | Thioredoxin reductase (EC 1.8.1.9) | 0 | 1 |
| lmo2487 | LMRG_01761 | Hypothetical protein, homolog of fig|393130.3.peg.2627 | 0 | 1 |
| lmo2507 | LMRG_01741 | Cell-division-associated, ABC-transporter-like signaling protein FtsE | 0 | 1 |
| lmo2524 | LMRG_01724 | 3-hydroxyacyl-[acyl-carrier-protein] dehydratase, FabZ form (EC 4.2.1.59) | 0 | 1 |
| lmo2528 | LMRG_01720 | ATP synthase epsilon chain (EC 3.6.3.14) | 0 | 1 |
| lmo2533 | LMRG_01715 | ATP synthase F0 sector subunit b (EC 3.6.3.14) | 0 | 1 |
| lmo2537 | LMRG_01710 | UDP-N-acetylglucosamine 2-epimerase (EC 5.1.3.14) | 0 | 1 |
| lmo2538 | LMRG_01709 | Uracil phosphoribosyltransferase (EC 2.4.2.9) | 0 | 1 |
| lmo2543 | LMRG_01704 | Peptide chain release factor 1 | 0 | 1 |
| lmo2548 | LMRG_01699 | LSU ribosomal protein L31p @ LSU ribosomal protein L31p, zinc-independent | 0 | 1 |
| lmo2556 | LMRG_01691 | Fructose-bisphosphate aldolase class II (EC 4.1.2.13) | 0 | 1 |
| lmo2562 | LMRG_02707 | Uncharacterized beta-barrel protein YwiB | 0 | 1 |
| lmo2578 | LMRG_02690 | cell surface hydrolase (putative) | 0 | 1 |
| lmo2584 | LMRG_02683 | Sulfur carrier protein FdhD | 0 | 1 |
| lmo2597 | LMRG_02141 | LSU ribosomal protein L13p (L13Ae) | 0 | 1 |
| lmo2600 | LMRG_02144 | ATPase component of general energizing module of ECF transporters | 0 | 1 |
| lmo2601 | LMRG_02145 | ATPase component of general energizing module of ECF transporters | 0 | 1 |
| lmo2605 | LMRG_02149 | LSU ribosomal protein L17p | 0 | 1 |
| lmo2606 | LMRG_02150 | DNA-directed RNA polymerase alpha subunit (EC 2.7.7.6) | 0 | 1 |
| lmo2607 | LMRG_02151 | SSU ribosomal protein S11p (S14e) | 0 | 1 |
| lmo2608 | LMRG_02152 | SSU ribosomal protein S13p (S18e) | 0 | 1 |
| lmo2609 | LMRG_02153 | LSU ribosomal protein L36p @ LSU ribosomal protein L36p, zinc-dependent | 0 | 1 |
| lmo2610 | LMRG_02906 | Translation initiation factor 1 | 0 | 1 |
| lmo2614 | LMRG_02158 | LSU ribosomal protein L30p (L7e) | 0 | 1 |
| lmo2615 | LMRG_02159 | SSU ribosomal protein S5p (S2e) | 0 | 1 |
| lmo2618 | LMRG_02162 | SSU ribosomal protein S8p (S15Ae) | 0 | 1 |
| lmo2619 | LMRG_02163 | SSU ribosomal protein S14p (S29e) @ SSU ribosomal protein S14p (S29e), zinc-dependent | 0 | 1 |
| lmo2620 | LMRG_02164 | LSU ribosomal protein L5p (L11e) | 0 | 1 |
| lmo2621 | LMRG_02165 | LSU ribosomal protein L24p (L26e) | 0 | 1 |
| lmo2622 | LMRG_02166 | LSU ribosomal protein L14p (L23e) | 0 | 1 |
| lmo2623 | LMRG_02167 | SSU ribosomal protein S17p (S11e) | 0 | 1 |
| lmo2624 | LMRG_02168 | LSU ribosomal protein L29p (L35e) | 0 | 1 |
| lmo2625 | LMRG_02169 | LSU ribosomal protein L16p (L10e) | 0 | 1 |
| lmo2626 | LMRG_02170 | SSU ribosomal protein S3p (S3e) | 0 | 1 |
| lmo2627 | LMRG_02171 | LSU ribosomal protein L22p (L17e) | 0 | 1 |
| lmo2628 | LMRG_02172 | SSU ribosomal protein S19p (S15e) | 0 | 1 |
| lmo2630 | LMRG_02174 | LSU ribosomal protein L23p (L23Ae) | 0 | 1 |
| lmo2631 | LMRG_02175 | LSU ribosomal protein L4p (L1e) | 0 | 1 |
| lmo2632 | LMRG_02176 | LSU ribosomal protein L3p (L3e) | 0 | 1 |
| lmo2633 | LMRG_02177 | SSU ribosomal protein S10p (S20e) | 0 | 1 |
| lmo2656 | LMRG_02201 | SSU ribosomal protein S12p (S23e) | 0 | 1 |
| lmo2663 | LMRG_02208 | Galactitol-1-phosphate 5-dehydrogenase (EC 1.1.1.251) | 0 | 1 |
| lmo2666 | LMRG_02211 | PTS system, galactitol-specific IIB component (EC 2.7.1.200) | 0 | 1 |
| lmo2671 | LMRG_02216 | hypothetical protein | 0 | 1 |
| lmo2672 | LMRG_02217 | Transcriptional regulator, AraC family | 0 | 1 |
| lmo2693 | LMRG_02240 | Thymidylate kinase (EC 2.7.4.9) | 0 | 1 |
| lmo2702 | LMRG_01995 | Recombination protein RecR | 0 | 1 |
| lmo2719 | LMRG_01977 | tRNA-specific adenosine-34 deaminase (EC 3.5.4.33) | 0 | 1 |
| lmo2789 | LMRG_01908 | Uncharacterized protein CAC3725 | 0 | 1 |
| lmo2790 | LMRG_01907 | Chromosome (plasmid) partitioning protein ParB | 0 | 1 |
| lmo2803 | LMRG_01893 | hypothetical protein | 0 | 1 |
| lmo2806 | LMRG_01890 | putative secreted protein | 0 | 1 |
| lmo2831 | LMRG_01867 | Beta-phosphoglucomutase (EC 5.4.2.6) | 0 | 1 |
| lmo2855 | LMRG_02426 | Ribonuclease P protein component (EC 3.1.26.5) | 0 | 1 |
| lmo2856 | LMRG_02427 | LSU ribosomal protein L34p | 0 | 1 |
| lmo2857 | LMRG_02428 | hypothetical protein | 0 | 1 |
| lmos73 | #N/A | hypothetical protein | 0 | 1 |
| #N/A | LMRG_02864 | hypothetical protein | 0 | 1 |
| #N/A | LMRG_02393 | hypothetical protein | 0 | 1 |
| #N/A | LMRG_02395 | FIG00774842: hypothetical protein | 0 | 1 |
| #N/A | LMRG_01515 | FIG00774055: hypothetical protein | 0 | 1 |
| #N/A | LMRG_02136 | CRISPR-associated protein Cas2 | 0 | 1 |
| #N/A | LMRG_02931 | hypothetical protein | 0 | 1 |
| #N/A | LMRG_02887 | hypothetical protein | 0 | 1 |
| #N/A | LMRG_00154 | hypothetical protein | 0 | 1 |
| #N/A | LMRG_02492 | Putative EsaC protein analog (Listeria type 3) | 0 | 1 |
| #N/A | LMRG_02849 | FIG00775292: hypothetical protein | 0 | 1 |
| #N/A | LMRG_02841 | Uncharacterized protein COG3236 | 0 | 1 |
| #N/A | LMRG_02074 | Component involved in D-alanylation of teichoic acids | 0 | 1 |
| lmo0270 | LMRG_02606 | hypothetical protein | 0.01 | 1 |
| lmo0439 | LMRG_00131 | Siderophore/Surfactin synthetase related protein | 0.01 | 1 |
| lmo0643 | LMRG_00330 | Transaldolase (EC 2.2.1.2) | 0.01 | 1 |
| lmo0872 | LMRG_02296 | Uncharacterized MFS-type transporter YtbD | 0.01 | 1 |
| lmo0936 | LMRG_02035 | FMN reductase (NADPH) (EC 1.5.1.38) | 0.01 | 1 |
| lmo1072 | LMRG_00534 | Pyruvate carboxylase (EC 6.4.1.1) | 0.01 | 1 |
| lmo1380 | LMRG_00832 | hypothetical protein | 0.01 | 1 |
| lmo2196 | LMRG_01636 | ABC transporter, substrate-binding protein (cluster 5, nickel/peptides/opines) | 0.01 | 1 |
| lmo0153 | LMRG_02398 | Zinc ABC transporter, substrate-binding lipoprotein AdcA | 0.02 | 1 |
| lmo0198 | LMRG_02620 | N-acetylglucosamine-1-phosphate uridyltransferase (EC 2.7.7.23) / Glucosamine-1-phosphate N-acetyltransferase (EC 2.3.1.157) | 0.02 | 1 |
| lmo0420 | LMRG_00112 | Cof-like hydrolase | 0.02 | 1 |
| lmo0509 | LMRG_00190 | Ribose-phosphate pyrophosphokinase (EC 2.7.6.1) | 0.02 | 1 |
| lmo1071 | LMRG_00533 | FtsW-like protein YlaO | 0.02 | 1 |
| lmo1319 | LMRG_00769 | Prolyl-tRNA synthetase (EC 6.1.1.15), bacterial type | 0.02 | 1 |
| lmo1320 | LMRG_00770 | DNA polymerase III polC-type (EC 2.7.7.7) | 0.02 | 1 |
| lmo1519 | LMRG_01451 | Aspartyl-tRNA synthetase (EC 6.1.1.12) | 0.02 | 1 |
| lmo1595 | LMRG_01372 | Free methionine-(R)-sulfoxide reductase, contains GAF domain | 0.02 | 1 |
| lmo1716 | LMRG_02554 | Transcriptional regulator, AcrR family | 0.02 | 1 |
| lmo1757 | LMRG_02514 | Putative pheromone cAM373 precursor lipoprotein CamS | 0.02 | 1 |
| lmo1824 | LMRG_00971 | Helicase PriA essential for oriC/DnaA-independent DNA replication | 0.02 | 1 |
| lmo1922 | LMRG_01069 | FIG009300: TPR-repeat-containing protein | 0.02 | 1 |
| lmo2504 | LMRG_01744 | SMC peptidase | 0.02 | 1 |
| lmo0165 | LMRG_02410 | tRNA(1)(Val) (adenine(37)-N(6))-methyltransferase (EC 2.1.1.223) | 0.03 | 1 |
| lmo0527 | LMRG_00208 | Membrane protein | 0.03 | 1 |
| lmo0697 | LMRG_00386 | Flagellar hook protein FlgE | 0.03 | 1 |
| lmo0782 | LMRG_00470 | PTS system, mannose-specific IIC component | 0.03 | 1 |
| lmo0884 | LMRG_02308 | Protoporphyrinogen IX oxidase, aerobic, HemY (EC 1.3.3.4) | 0.03 | 1 |
| lmo0961 | LMRG_02060 | Uncharacterized protease YrrO | 0.03 | 1 |
| lmo1276 | LMRG_00726 | Methylenetetrahydrofolate--tRNA-(uracil-5-)-methyltransferase TrmFO (EC 2.1.1.74) | 0.03 | 1 |
| lmo1438 | LMRG_00890 | Uncharacterized protein YqgF | 0.03 | 1 |
| lmo1448 | LMRG_00900 | Manganese-dependent inorganic pyrophosphatase (EC 3.6.1.1) | 0.03 | 1 |
| lmo1458 | LMRG_00910 | Glycyl-tRNA synthetase beta chain (EC 6.1.1.14) | 0.03 | 1 |
| lmo1738 | LMRG_02533 | ABC transporter, substrate-binding protein (cluster 3, basic aa/glutamine/opines) | 0.03 | 1 |
| lmo1755 | LMRG_02516 | Aspartyl-tRNA(Asn) amidotransferase subunit A (EC 6.3.5.6) @ Glutamyl-tRNA(Gln) amidotransferase subunit A (EC 6.3.5.7) | 0.03 | 1 |
| lmo2414 | LMRG_01834 | Iron-sulfur cluster assembly protein SufD | 0.03 | 1 |
| lmo2483 | LMRG_01765 | HPr kinase/phosphorylase | 0.03 | 1 |
| lmo2582 | LMRG_02685 | Sensor histidine kinase colocalized with HrtAB transporter | 0.03 | 1 |
| lmo0048 | LMRG_02477 | Accessory gene regulator protein B (EC 3.4.-.-) | 0.04 | 1 |
| lmo0925 | LMRG_02024 | Efflux ABC transporter, permease protein | 0.04 | 1 |
| lmo1591 | LMRG_01376 | N-acetyl-gamma-glutamyl-phosphate reductase (EC 1.2.1.38) | 0.04 | 1 |
| lmo2010 | LMRG_01158 | DNA-binding response regulator, AraC family | 0.04 | 1 |
| lmo2041 | LMRG_01190 | 16S rRNA (cytosine(1402)-N(4))-methyltransferase (EC 2.1.1.199) | 0.04 | 1 |
| lmo2629 | LMRG_02173 | LSU ribosomal protein L2p (L8e) | 0.04 | 1 |
| lmo2825 | LMRG_01873 | Phosphoserine aminotransferase (EC 2.6.1.52) | 0.04 | 1 |
| lmo0669 | LMRG_00357 | Oxidoreductase, short-chain dehydrogenase/reductase family | 0.05 | 1 |
| lmo0808 | LMRG_02789 | Spermidine/putrescine import ABC transporter permease protein PotB (TC 3.A.1.11.1) | 0.05 | 1 |
| lmo0823 | LMRG_02248 | oxidoreductase of aldo/keto reductase family, subgroup 1 | 0.05 | 1 |
| lmo1041 | LMRG_00502 | Molybdenum ABC transporter, substrate-binding protein ModA | 0.05 | 1 |
| lmo1093 | LMRG_00555 | NAD synthetase (EC 6.3.1.5) | 0.05 | 1 |
| lmo1257 | LMRG_00706 | hypothetical protein | 0.05 | 1 |
| lmo1552 | LMRG_01416 | Valyl-tRNA synthetase (EC 6.1.1.9) | 0.05 | 1 |
| lmo1860 | LMRG_01007 | Peptide-methionine (S)-S-oxide reductase MsrA (EC 1.8.4.11) | 0.05 | 1 |
| lmo1869 | LMRG_01016 | hypothetical protein | 0.05 | 1 |
| lmo1902 | LMRG_01049 | 3-methyl-2-oxobutanoate hydroxymethyltransferase (EC 2.1.2.11) | 0.05 | 1 |
| lmo2073 | LMRG_01224 | Bis-ABC ATPase YheS | 0.05 | 1 |
| lmo2468 | LMRG_01780 | ATP-dependent Clp protease proteolytic subunit ClpP (EC 3.4.21.92) | 0.05 | 1 |
| lmo2747 | LMRG_01949 | Seryl-tRNA synthetase (EC 6.1.1.11) | 0.05 | 1 |
| lmo0045 | LMRG_02474 | Single-stranded DNA-binding protein | 0.06 | 1 |
| lmo1655 | LMRG_01312 | Uncharacterized membrane spanning protein, contains VanZ-like domain | 0.06 | 1 |
| lmo1846 | LMRG_00993 | Multi antimicrobial extrusion protein (Na(+)/drug antiporter), MATE family of MDR efflux pumps | 0.06 | 1 |
| lmo2655 | LMRG_02200 | SSU ribosomal protein S7p (S5e) | 0.06 | 1 |
| lmo0054 | LMRG_02483 | Replicative DNA helicase (DnaB) (EC 3.6.4.12) | 0.07 | 1 |
| lmo0135 | LMRG_02384 | Oligopeptide ABC transporter, substrate-binding protein OppA (TC 3.A.1.5.1) | 0.07 | 1 |
| lmo0156 | LMRG_02401 | cyclic nucleotide-binding protein | 0.07 | 1 |
| lmo0258 | LMRG_02651 | DNA-directed RNA polymerase beta subunit (EC 2.7.7.6) | 0.07 | 1 |
| lmo0545 | LMRG_00227 | Glucitol operon activator protein | 0.07 | 1 |
| lmo1006 | LMRG_02106 | N-acetyl-L,L-diaminopimelate aminotransferase (EC 2.6.1.-) | 0.07 | 1 |
| lmo1077 | LMRG_00539 | Putative polyribitolphosphotransferase | 0.07 | 1 |
| lmo1414 | LMRG_00866 | 3-ketoacyl-CoA thiolase (EC 2.3.1.16) @ Acetyl-CoA acetyltransferase (EC 2.3.1.9) | 0.07 | 1 |
| lmo1600 | LMRG_01367 | Chorismate mutase I (EC 5.4.99.5) / 2-keto-3-deoxy-D-arabino-heptulosonate-7-phosphate synthase I beta (EC 2.5.1.54) | 0.07 | 1 |
| lmo1766 | LMRG_02505 | Phosphoribosylglycinamide formyltransferase (EC 2.1.2.2) | 0.07 | 1 |
| lmo2034 | LMRG_01183 | Cell division protein FtsQ | 0.07 | 1 |
| lmo2201 | LMRG_01631 | 3-oxoacyl-[acyl-carrier-protein] synthase, KASII (EC 2.3.1.179) | 0.07 | 1 |
| lmo2514 | LMRG_01734 | FIG005590: DegV family protein | 0.07 | 1 |
| #N/A | LMRG_02950 | hypothetical protein | 0.07 | 1 |
| lmo0218 | LMRG_02640 | RNA binding protein, contains ribosomal protein S1 domain | 0.08 | 1 |
| lmo0248 | LMRG_02658 | LSU ribosomal protein L11p (L12e) | 0.08 | 1 |
| lmo0266 | LMRG_02610 | Transcriptional regulator, MarR family | 0.08 | 1 |
| lmo0999 | LMRG_02099 | hypothetical protein | 0.08 | 1 |
| lmo1226 | LMRG_00672 | Transmembrane transport protein MmpL family | 0.08 | 1 |
| lmo1473 | LMRG_00926 | Chaperone protein DnaK | 0.08 | 1 |
| lmo1977 | LMRG_01124 | Ribonuclease Z (EC 3.1.26.11) | 0.08 | 1 |
| lmo2008 | LMRG_01156 | ABC transporter, permease protein 2 (cluster 1, maltose/g3p/polyamine/iron) | 0.08 | 1 |
| lmo2083 | LMRG_01234 | hypothetical protein | 0.08 | 1 |
| lmo2106 | LMRG_01260 | Ser/Thr protein phosphatase family protein | 0.08 | 1 |
| lmo0132 | LMRG_02381 | Inosine-5'-monophosphate dehydrogenase (EC 1.1.1.205) / CBS domain | 0.09 | 1 |
| lmo0627 | LMRG_00310 | hypothetical protein | 0.09 | 1 |
| lmo1574 | LMRG_02943 | DNA polymerase III alpha subunit (EC 2.7.7.7) | 0.09 | 1 |
| lmo1667 | LMRG_01300 | L-lactate dehydrogenase (EC 1.1.1.27) | 0.09 | 1 |
| lmo2367 | LMRG_02712 | Glucose-6-phosphate isomerase (EC 5.3.1.9) | 0.09 | 1 |
| lmo2734 | LMRG_01962 | glycosyl hydrolase, family 38 | 0.09 | 1 |
| lmo2810 | LMRG_01888 | tRNA-5-carboxymethylaminomethyl-2-thiouridine(34) synthesis protein MnmG | 0.09 | 1 |
| lmo0006 | LMRG_02434 | DNA gyrase subunit B (EC 5.99.1.3) | 0.1 | 1 |
| lmo0044 | LMRG_02473 | SSU ribosomal protein S6p | 0.1 | 1 |
| lmo0093 | LMRG_02342 | ATP synthase epsilon chain (EC 3.6.3.14) | 0.1 | 1 |
| lmo0445 | LMRG_00137 | hypothetical protein | 0.1 | 1 |
| lmo0994 | LMRG_02094 | hypothetical protein | 0.1 | 1 |
| lmo1051 | LMRG_00512 | Peptide deformylase (EC 3.5.1.88) | 0.1 | 1 |
| lmo1204 | LMRG_00650 | Cobalt ECF transporter, substrate-binding protein CbiM | 0.1 | 1 |
| lmo1504 | LMRG_01466 | Alanyl-tRNA synthetase (EC 6.1.1.7) | 0.1 | 1 |
| lmo2033 | LMRG_01182 | Cell division protein FtsA | 0.1 | 1 |
| lmo2042 | LMRG_01191 | Transcriptional regulator MraZ | 0.1 | 1 |
| lmo2301 | LMRG_01531 | putative terminase small subunit | 0.1 | 1 |
| lmo0280 | LMRG_02595 | Ribonucleotide reductase of class III (anaerobic), activating protein (EC 1.97.1.4) | 0.11 | 1 |
| lmo0284 | LMRG_02587 | Methionine ABC transporter ATP-binding protein | 0.11 | 1 |
| lmo1034 | LMRG_00495 | Unknown pentose kinase TM0952 | 0.11 | 1 |
| lmo1741 | LMRG_02530 | Two-component system sensor histidine kinase | 0.11 | 1 |
| lmo1842 | LMRG_00989 | STAS domain protein | 0.11 | 1 |
| lmo1919 | LMRG_01066 | Putative membrane protease YugP | 0.11 | 1 |
| lmo2198 | LMRG_01634 | Tryptophanyl-tRNA synthetase (EC 6.1.1.2) | 0.11 | 1 |
| lmo2508 | LMRG_01740 | UPF0750 membrane protein YvjA | 0.11 | 1 |
| lmo2565 | LMRG_02704 | dNTP triphosphohydrolase, broad substrate specificity | 0.11 | 1 |
| lmo2617 | LMRG_02161 | LSU ribosomal protein L6p (L9e) | 0.11 | 1 |
| lmo2793 | LMRG_01904 | hypothetical protein | 0.11 | 1 |
| lmo0848 | LMRG_02271 | Glutamine ABC transporter, ATP-binding protein GlnQ | 0.12 | 1 |
| lmo1178 | LMRG_00624 | Ethanolamine utilization polyhedral-body-like protein EutM | 0.12 | 1 |
| lmo1183 | LMRG_00629 | Protein clustered with ethanolamine utilization | 0.12 | 1 |
| lmo1193 | LMRG_00639 | Cobalt-precorrin-8 methylmutase (EC 5.4.99.60) | 0.12 | 1 |
| lmo1435 | LMRG_00887 | 4-hydroxy-tetrahydrodipicolinate synthase (EC 4.3.3.7) | 0.12 | 1 |
| lmo1834 | LMRG_00981 | Dihydroorotate dehydrogenase (NAD(+)), electron transfer subunit (EC 1.3.1.14) | 0.12 | 1 |
| lmo2096 | LMRG_01247 | PTS system, galactitol-specific IIC component | 0.12 | 1 |
| lmo1385 | LMRG_00837 | Uncharacterized UPF0750 membrane protein | 0.13 | 1 |
| lmo1686 | LMRG_02760 | Putative lipoprotein SAV1865 | 0.13 | 1 |
| lmo1889 | LMRG_01036 | UPF0398 protein YpsA | 0.13 | 1 |
| lmo1898 | LMRG_01045 | Uncharacterized protein YpmB | 0.13 | 1 |
| lmo2167 | LMRG_01665 | MBL-fold metallo-hydrolase superfamily | 0.13 | 1 |
| lmo2739 | LMRG_01957 | NAD-dependent protein deacetylase of SIR2 family | 0.13 | 1 |
| lmo2766 | LMRG_01929 | Transcriptional regulator, RpiR family | 0.13 | 1 |
| lmo0184 | LMRG_02970 | Oligo-1,6-glucosidase (EC 3.2.1.10) | 0.14 | 1 |
| lmo1316 | LMRG_00766 | Phosphatidate cytidylyltransferase (EC 2.7.7.41) | 0.14 | 1 |
| lmo1559 | LMRG_01408 | Threonyl-tRNA synthetase (EC 6.1.1.3) | 0.14 | 1 |
| lmo1778 | LMRG_02834 | ABC transporter, ATP-binding protein | 0.14 | 1 |
| lmo1804 | LMRG_00951 | Chromosome partition protein smc | 0.14 | 1 |
| lmo1887 | LMRG_01034 | 23S rRNA (guanine(2445)-N(2))-methyltransferase (EC 2.1.1.173) | 0.14 | 1 |
| lmo1917 | LMRG_01064 | Pyruvate formate-lyase (EC 2.3.1.54) | 0.14 | 1 |
| lmo2788 | LMRG_01909 | Beta-glucoside bgl operon antiterminator, BglG family | 0.14 | 1 |
| lmo0011 | LMRG_02440 | Diphosphomevalonate decarboxylase (EC 4.1.1.33) | 0.15 | 1 |
| lmo0228 | LMRG_02836 | Lysyl-tRNA synthetase (class II) (EC 6.1.1.6) | 0.15 | 1 |
| lmo0861 | LMRG_02284 | Sugar ABC transporter, permease protein precursor | 0.15 | 1 |
| lmo1138 | LMRG_00581 | ATP-dependent Clp protease proteolytic subunit ClpP2 (EC 3.4.21.92) | 0.15 | 1 |
| lmo2232 | LMRG_01600 | Hemolysins and related proteins containing CBS domains | 0.15 | 1 |
| lmo2540 | LMRG_01707 | Low molecular weight protein tyrosine phosphatase (EC 3.1.3.48) | 0.15 | 1 |
| lmo2551 | LMRG_01696 | Transcription termination factor Rho | 0.15 | 1 |
| lmo0027 | LMRG_02456 | PTS system, beta-glucoside-specific IIB component / PTS system, beta-glucoside-specific IIC component / PTS system, beta-glucoside-specific IIA component | 0.16 | 1 |
| lmo0433 | LMRG_00126 | Internalin A (LPXTG motif) | 0.16 | 1 |
| lmo1089 | LMRG_00551 | Glycerol-3-phosphate cytidylyltransferase (EC 2.7.7.39) | 0.16 | 1 |
| lmo1660 | LMRG_01307 | Leucyl-tRNA synthetase (EC 6.1.1.4) | 0.16 | 1 |
| lmo2206 | LMRG_01626 | Chaperone protein ClpB (ATP-dependent unfoldase) | 0.16 | 1 |
| lmo2344 | LMRG_01499 | Putative glutaredoxin YtnI | 0.16 | 1 |
| lmo2376 | LMRG_02721 | Peptidyl-prolyl cis-trans isomerase (EC 5.2.1.8) | 0.16 | 1 |
| lmo0225 | LMRG_02839 | Dihydroneopterin aldolase (EC 4.1.2.25) | 0.17 | 1 |
| lmo0264 | LMRG_02612 | Internalin E (LPXTG motif) | 0.17 | 1 |
| lmo0692 | LMRG_00380 | Signal transduction histidine kinase CheA | 0.17 | 1 |
| lmo0819 | LMRG_02244 | hypothetical protein | 0.17 | 1 |
| lmo1807 | LMRG_00954 | 3-oxoacyl-[acyl-carrier protein] reductase (EC 1.1.1.100), FadG | 0.17 | 1 |
| lmo2171 | LMRG_01661 | Oxalate/formate antiporter | 0.17 | 1 |
| lmo2249 | LMRG_01582 | Probable low-affinity inorganic phosphate transporter | 0.17 | 1 |
| lmo2381 | LMRG_02726 | Na(+) H(+) antiporter subunit D | 0.17 | 1 |
| lmo2596 | LMRG_02140 | SSU ribosomal protein S9p (S16e) | 0.17 | 1 |
| lmo0003 | LMRG_02431 | hypothetical protein | 0.18 | 1 |
| lmo0224 | LMRG_02645 | Dihydropteroate synthase (EC 2.5.1.15) | 0.18 | 1 |
| lmo2119 | LMRG_01273 | Uncharacterized secreted protein associated with spyDAC | 0.18 | 1 |
| lmo2417 | LMRG_01831 | Methionine ABC transporter substrate-binding protein | 0.18 | 1 |
| lmo0072 | LMRG_02324 | hypothetical protein | 0.19 | 1 |
| lmo0195 | LMRG_02617 | ABC-type antimicrobial peptide transport system, permease component | 0.19 | 1 |
| lmo0342 | LMRG_00033 | Transketolase (EC 2.2.1.1) | 0.19 | 1 |
| lmo0701 | LMRG_00390 | hypothetical protein | 0.19 | 1 |
| lmo1455 | LMRG_00907 | DNA primase DnaG | 0.19 | 1 |
| lmo1798 | LMRG_02822 | poly (glycerol-phosphate) alpha-glucosyltransferase | 0.19 | 1 |
| lmo1982 | LMRG_01130 | GNAT family acetyltransferase BA4593 | 0.19 | 1 |
| lmo2036 | LMRG_01185 | UDP-N-acetylmuramoyl-L-alanine--D-glutamate ligase (EC 6.3.2.9) | 0.19 | 1 |
| lmo2690 | LMRG_02237 | Transcriptional regulator, AcrR family | 0.19 | 1 |
| lmo0036 | LMRG_02465 | Putrescine carbamoyltransferase (EC 2.1.3.6) | 0.2 | 1 |
| lmo0829 | LMRG_02252 | Pyruvate-flavodoxin oxidoreductase | 0.2 | 1 |
| lmo1040 | LMRG_00501 | Molybdenum ABC transporter permease protein ModB | 0.2 | 1 |
| lmo1315 | LMRG_00765 | Undecaprenyl diphosphate synthase (EC 2.5.1.31) | 0.2 | 1 |
| lmo2587 | LMRG_02680 | hypothetical protein | 0.2 | 1 |
| lmo0175 | LMRG_02740 | Internalin-like protein (LPXTG motif) Lmo0610 homolog | 0.21 | 1 |
| lmo0645 | LMRG_00332 | Uncharacterized amino acid permease, GabP family | 0.21 | 1 |
| lmo1088 | LMRG_00550 | CDP-glycerol:glycerophosphate glycerophosphotransferase (tagB) | 0.21 | 1 |
| lmo1302 | LMRG_00752 | SOS-response repressor and protease LexA (EC 3.4.21.88) | 0.21 | 1 |
| lmo1485 | LMRG_00938 | FIG145533: Methyltransferase (EC 2.1.1.-) | 0.21 | 1 |
| lmo1926 | LMRG_01073 | Chorismate mutase II (EC 5.4.99.5) | 0.21 | 1 |
| lmo2011 | LMRG_01159 | Two-component system sensor histidine kinase | 0.21 | 1 |
| lmo2168 | LMRG_01664 | Lactoylglutathione lyase (EC 4.4.1.5) | 0.21 | 1 |
| lmo2332 | LMRG_01511 | Integrase [Bacteriophage A118] | 0.21 | 1 |
| lmo2561 | LMRG_02708 | Arginyl-tRNA synthetase (EC 6.1.1.19) | 0.21 | 1 |
| lmo0160 | LMRG_02405 | Putative peptidoglycan bound protein (LPXTG motif) Lmo0160 homolog | 0.22 | 1 |
| lmo0327 | LMRG_00021 | Internalin-like protein (LPXTG motif) Lmo0327 homolog / murein-hydrolysing domain | 0.22 | 1 |
| lmo0437 | LMRG_00129 | Oxidoreductase | 0.22 | 1 |
| lmo0480 | LMRG_02882 | Transcriptional regulator, AcrR family | 0.22 | 1 |
| lmo0569 | LMRG_00251 | ATP phosphoribosyltransferase regulatory subunit (EC 2.4.2.17) | 0.22 | 1 |
| lmo1011 | LMRG_02111 | 2,3,4,5-tetrahydropyridine-2,6-dicarboxylate N-acetyltransferase (EC 2.3.1.89) | 0.22 | 1 |
| lmo1143 | LMRG_00586 | Propanediol utilization polyhedral body protein PduT | 0.22 | 1 |
| lmo1547 | LMRG_01423 | Rod shape-determining protein MreC | 0.22 | 1 |
| lmo2144 | LMRG_01688 | Transcriptional regulator, GntR family | 0.22 | 1 |
| lmo2579 | LMRG_02689 | hypothetical protein | 0.22 | 1 |
| lmo2713 | LMRG_01983 | secreted protein with 1 GW repeat | 0.22 | 1 |
| lmo0289 | LMRG_02582 | Two-component system YycFG regulatory protein YycH | 0.23 | 1 |
| lmo0810 | LMRG_02787 | Spermidine/putrescine import ABC transporter substrate-binding protein PotD (TC 3.A.1.11.1) | 0.23 | 1 |
| lmo1618 | LMRG_01348 | Transcriptional regulator, MarR family | 0.23 | 1 |
| lmo1622 | LMRG_01344 | ADP-dependent (S)-NAD(P)H-hydrate dehydratase (EC 4.2.1.136) | 0.23 | 1 |
| lmo1859 | LMRG_02979 | Peptide-methionine (R)-S-oxide reductase MsrB (EC 1.8.4.12) | 0.23 | 1 |
| lmo1954 | LMRG_01101 | Phosphopentomutase (EC 5.4.2.7) | 0.23 | 1 |
| lmo2569 | LMRG_02699 | Oligopeptide ABC transporter, substrate-binding protein OppA (TC 3.A.1.5.1) | 0.23 | 1 |
| lmo0107 | LMRG_02356 | Heterodimeric efflux ABC transporter, permease/ATP-binding subunit 2 | 0.24 | 1 |
| lmo0250 | LMRG_02656 | LSU ribosomal protein L10p (P0) | 0.24 | 1 |
| lmo0856 | LMRG_02279 | UDP-N-acetylmuramoyl-tripeptide--D-alanyl-D-alanine ligase (EC 6.3.2.10) | 0.24 | 1 |
| lmo1010 | LMRG_02110 | LysR-family transcriptional regulator CcpC, catabolite repressor of CitB and CitZ expression | 0.24 | 1 |
| lmo1572 | LMRG_01395 | Acetyl-coenzyme A carboxyl transferase alpha chain (EC 6.4.1.2) | 0.24 | 1 |
| lmo1943 | LMRG_01090 | hypothetical protein YpbB | 0.24 | 1 |
| lmo2070 | LMRG_01221 | CAAX amino terminal protease family protein | 0.24 | 1 |
| lmo2505 | LMRG_01743 | D-glutamyl-L-m-Dpm peptidase P45 | 0.24 | 1 |
| lmo2611 | LMRG_02155 | Adenylate kinase (EC 2.7.4.3) | 0.24 | 1 |
| lmo2673 | LMRG_02218 | Universal stress protein family | 0.24 | 1 |
| lmo2814 | LMRG_01884 | Transcriptional regulator, AcrR family | 0.24 | 1 |
| lmo0262 | LMRG_02647 | Internalin G (LPXTG motif) | 0.25 | 1 |
| lmo0689 | LMRG_00377 | Chemotaxis protein CheV (EC 2.7.3.-) | 0.25 | 1 |
| lmo0765 | LMRG_00453 | hypothetical protein | 0.25 | 1 |
| lmo0814 | LMRG_02783 | Enoyl-[acyl-carrier-protein] reductase [FMN, NADH] (EC 1.3.1.9), FabK => refractory to triclosan | 0.25 | 1 |
| lmo1096 | LMRG_00558 | GMP synthase [glutamine-hydrolyzing], amidotransferase subunit (EC 6.3.5.2) / GMP synthase [glutamine-hydrolyzing], ATP pyrophosphatase subunit (EC 6.3.5.2) | 0.25 | 1 |
| lmo1117 | LMRG_00560 | Glyoxalase family protein | 0.25 | 1 |
| lmo1463 | LMRG_00915 | Cytidine deaminase (EC 3.5.4.5) | 0.25 | 1 |
| lmo1522 | LMRG_01448 | D-aminoacyl-tRNA deacylase (EC 3.1.1.96) | 0.25 | 1 |
| lmo2121 | LMRG_01275 | Maltose phosphorylase (EC 2.4.1.8) | 0.25 | 1 |
| lmo0220 | LMRG_02642 | Cell division-associated, ATP-dependent zinc metalloprotease FtsH | 0.26 | 1 |
| lmo0444 | LMRG_00136 | hypothetical protein | 0.26 | 1 |
| lmo0613 | LMRG_00296 | Zinc-type alcohol dehydrogenase-like protein | 0.26 | 1 |
| lmo0745 | LMRG_00433 | hypothetical protein | 0.26 | 1 |
| lmo0836 | LMRG_02259 | PsiE protein | 0.26 | 1 |
| lmo1221 | LMRG_00667 | Phenylalanyl-tRNA synthetase alpha chain (EC 6.1.1.20) | 0.26 | 1 |
| lmo1493 | LMRG_00946 | Oligoendopeptidase F-like protein | 0.26 | 1 |
| lmo1569 | LMRG_01398 | UPF0716 protein FxsA | 0.26 | 1 |
| lmo1615 | LMRG_01351 | tRNA (guanine(46)-N(7))-methyltransferase (EC 2.1.1.33) | 0.26 | 1 |
| lmo1722 | LMRG_02549 | ATP-dependent RNA helicase YfmL | 0.26 | 1 |
| lmo2186 | LMRG_01646 | NPQTN cell wall anchored protein IsdC | 0.26 | 1 |
| lmo0498 | LMRG_00179 | Ribose-5-phosphate isomerase B (EC 5.3.1.6) | 0.27 | 1 |
| lmo0594 | LMRG_00276 | Homoserine O-acetyltransferase (EC 2.3.1.31) | 0.27 | 1 |
| lmo0656 | LMRG_00343 | Terminal oxidase biogenesis protein CtaM, putative heme A, heme O chaperone | 0.27 | 1 |
| lmo1008 | LMRG_02108 | Uncharacterized protein YkuJ | 0.27 | 1 |
| lmo1507 | LMRG_01463 | Uncharacterized transcriptional response regulator YclJ | 0.27 | 1 |
| lmo1693 | LMRG_02767 | Regulatory protein RecX | 0.27 | 1 |
| lmo2043 | LMRG_01192 | Uncharacterized MFS-type transporter | 0.27 | 1 |
| lmo2553 | LMRG_01694 | Integral membrane protein LafC, accessory function in glycolipid and LTA synthesis | 0.27 | 1 |
| lmo0239 | LMRG_02667 | Cysteinyl-tRNA synthetase (EC 6.1.1.16) | 0.28 | 1 |
| lmo0339 | LMRG_00030 | Inorganic pyrophosphatase | 0.28 | 1 |
| lmo1283 | LMRG_00733 | aldose epimerase family protein | 0.28 | 1 |
| lmo1286 | LMRG_00736 | DNA topoisomerase IV subunit B (EC 5.99.1.3) | 0.28 | 1 |
| lmo1445 | LMRG_00897 | Zinc uptake regulation protein Zur | 0.28 | 1 |
| lmo1641 | LMRG_01325 | Aconitate hydratase (EC 4.2.1.3) | 0.28 | 1 |
| lmo1895 | LMRG_01042 | Chromosome replication initiation protein DnaD | 0.28 | 1 |
| lmo1939 | LMRG_01086 | Cytidylate kinase (EC 2.7.4.25) | 0.28 | 1 |
| lmo2209 | LMRG_01623 | Acetyltransferase, GNAT family | 0.28 | 1 |
| lmo2241 | LMRG_01591 | Transcriptional regulator, GntR family | 0.28 | 1 |
| lmo2295 | LMRG_01537 | Protein gp8 [Bacteriophage A118] | 0.28 | 1 |
| lmo2384 | LMRG_02729 | Na(+) H(+) antiporter subunit G | 0.28 | 1 |
| lmo2457 | LMRG_01791 | Triosephosphate isomerase (EC 5.3.1.1) | 0.28 | 1 |
| lmo0159 | LMRG_02404 | Putative peptidoglycan bound protein (LPXTG motif) Lmo0159 homolog | 0.29 | 1 |
| lmo0566 | LMRG_00248 | Imidazoleglycerol-phosphate dehydratase (EC 4.2.1.19) | 0.29 | 1 |
| lmo0776 | LMRG_00464 | Sugar kinase and transcription regulator (EC 2.7.1.-) | 0.29 | 1 |
| lmo2032 | LMRG_01181 | Cell division protein FtsZ | 0.29 | 1 |
| lmo2124 | LMRG_01278 | Maltodextrin ABC transporter, permease protein MdxF | 0.29 | 1 |
| lmo0255 | LMRG_02654 | putative lipoprotein | 0.3 | 1 |
| lmo2335 | LMRG_01508 | PTS system, fructose-specific IIA component (EC 2.7.1.202) / PTS system, fructose-specific IIB component (EC 2.7.1.202) / PTS system, fructose-specific IIC component | 0.3 | 1 |
| lmo0488 | LMRG_00169 | Transcriptional regulator, LysR family | 0.31 | 1 |
| lmo0614 | LMRG_00297 | Acetyltransferase, GNAT family | 0.31 | 1 |
| lmo0933 | LMRG_02032 | Uncharacterized glycosyltransferase YkcC | 0.31 | 1 |
| lmo1252 | LMRG_00699 | UPF0750 membrane protein YxkD | 0.31 | 1 |
| lmo1298 | LMRG_00748 | Transcriptional regulator GlnR | 0.31 | 1 |
| lmo0246 | LMRG_02660 | Transcription antitermination protein NusG | 0.32 | 1 |
| lmo0388 | LMRG_00081 | hypothetical protein | 0.32 | 1 |
| lmo1609 | LMRG_01357 | FIG009688: Thioredoxin | 0.32 | 1 |
| lmo1643 | LMRG_01323 | STAS domain protein | 0.32 | 1 |
| lmo1803 | LMRG_00950 | Signal recognition particle receptor FtsY | 0.32 | 1 |
| lmo2020 | LMRG_01169 | Cell division initiation protein DivIVA | 0.32 | 1 |
| lmo2071 | LMRG_01222 | hypothetical protein | 0.32 | 1 |
| lmo2091 | LMRG_01242 | Argininosuccinate lyase (EC 4.3.2.1) | 0.32 | 1 |
| lmo2761 | LMRG_01934 | 6-phospho-beta-glucosidase (EC 3.2.1.86) | 0.32 | 1 |
| lmo0883 | LMRG_02307 | transmembrane protein, distant homology with ydbT | 0.33 | 1 |
| lmo1233 | LMRG_00679 | Thioredoxin | 0.33 | 1 |
| lmo1829 | LMRG_00976 | Fibronectin/fibrinogen-binding protein | 0.33 | 1 |
| lmo2247 | LMRG_01585 | oxidoreductase of aldo/keto reductase family, subgroup 2 | 0.33 | 1 |
| lmo2406 | LMRG_01842 | UPF0759 protein YunF | 0.33 | 1 |
| lmo2563 | LMRG_02706 | Uncharacterized membrane zinc metalloprotease YwhC | 0.33 | 1 |
| lmo0635 | LMRG_00318 | 2-haloalkanoic acid dehalogenase (EC 3.8.1.2) | 0.34 | 1 |
| lmo0987 | LMRG_02087 | Efflux ABC transporter, permease protein | 0.34 | 1 |
| lmo2157 | LMRG_01675 | Alkyl sulfatase and related hydrolases, MBL-fold metallo-hydrolase superfamily | 0.34 | 1 |
| lmo0087 | LMRG_02336 | hypothetical protein | 0.35 | 1 |
| lmo0373 | LMRG_00065 | PTS system, cellobiose-specific IIC component | 0.35 | 1 |
| lmo0666 | LMRG_00353 | UPF0382 membrane protein YwdK | 0.35 | 1 |
| lmo0811 | LMRG_02786 | Carbonic anhydrase, alpha class (EC 4.2.1.1) | 0.35 | 1 |
| lmo1400 | LMRG_00852 | Acetyltransferase, GNAT family | 0.35 | 1 |
| lmo2064 | LMRG_01214 | Large-conductance mechanosensitive channel | 0.35 | 1 |
| lmo2654 | LMRG_02199 | Translation elongation factor G | 0.35 | 1 |
| lmo0139 | LMRG_02388 | hypothetical protein | 0.36 | 1 |
| lmo0436 | LMRG_00128 | Rrf2 family transcriptional regulator, group III | 0.36 | 1 |
| lmo0785 | LMRG_00473 | NtrC family Transcriptional regulator, ATPase domain | 0.36 | 1 |
| lmo1035 | LMRG_00496 | PTS system, beta-glucoside-specific IIB component / PTS system, beta-glucoside-specific IIC component / PTS system, beta-glucoside-specific IIA component | 0.36 | 1 |
| lmo1210 | LMRG_00656 | Membrane protein | 0.36 | 1 |
| lmo2667 | LMRG_02212 | PTS system, galactitol-specific IIA component (EC 2.7.1.200) | 0.36 | 1 |
| lmo0096 | LMRG_02345 | PTS system, mannose-specific IIA component (EC 2.7.1.191) / PTS system, mannose-specific IIB component (EC 2.7.1.191) | 0.37 | 1 |
| lmo0272 | LMRG_02604 | Sugar phosphatase YidA (EC 3.1.3.23) | 0.37 | 1 |
| lmo0511 | LMRG_00192 | Glutamine amidotransferase, class I | 0.37 | 1 |
| lmo0625 | LMRG_00308 | hypothetical protein | 0.37 | 1 |
| lmo0628 | LMRG_00311 | hypothetical protein | 0.37 | 1 |
| lmo1527 | LMRG_01443 | Protein translocase subunit SecD / Protein translocase subunit SecF | 0.37 | 1 |
| lmo2527 | LMRG_01721 | Uncharacterized membrane protein YwzB | 0.37 | 1 |
| lmo0172 | LMRG_02983 | Mobile element protein | 0.38 | 1 |
| lmo0371 | LMRG_00063 | Transcriptional regulator, GntR family | 0.38 | 1 |
| lmo0418 | LMRG_00110 | hypothetical protein | 0.38 | 1 |
| lmo1397 | LMRG_00849 | ADP-ribose pyrophosphatase of COG1058 family (EC 3.6.1.13) / Nicotinamide-nucleotide amidase (EC 3.5.1.42) | 0.38 | 1 |
| lmo1431 | LMRG_00883 | Bis-ABC ATPase YbiT | 0.38 | 1 |
| lmo1582 | LMRG_01385 | Adenine-specific methyltransferase (EC 2.1.1.72) | 0.38 | 1 |
| lmo2283 | LMRG_01549 | gp20 | 0.38 | 1 |
| lmo0813 | LMRG_02784 | Fructokinase (EC 2.7.1.4) | 0.39 | 1 |
| lmo2506 | LMRG_01742 | Cell-division-associated, ABC-transporter-like signaling protein FtsX | 0.39 | 1 |
| lmo1505 | LMRG_01465 | ABC-type antimicrobial peptide transport system, ATPase component | 0.4 | 1 |
| lmo1911 | LMRG_01058 | GGDEF domain protein | 0.4 | 1 |
| lmo2012 | LMRG_01160 | hypothetical protein | 0.4 | 1 |
| lmo2089 | LMRG_01240 | Esterase/lipase | 0.4 | 1 |
| lmo0581 | LMRG_00263 | Putative ribosomal RNA large subunit methyltransferase YwbD | 0.41 | 1 |
| lmo0840 | LMRG_02263 | Transcriptional regulator, MarR family | 0.41 | 1 |
| lmo1037 | LMRG_00498 | Uncharacterized membrane protein YoaT | 0.41 | 1 |
| lmo1907 | LMRG_01054 | 4-hydroxy-tetrahydrodipicolinate reductase (EC 1.17.1.8) | 0.41 | 1 |
| lmo2291 | LMRG_01541 | Phage major tail shaft protein | 0.41 | 1 |
| lmo2748 | LMRG_01948 | general stress protein 26 | 0.41 | 1 |
| lmo0090 | LMRG_02339 | ATP synthase alpha chain (EC 3.6.3.14) | 0.42 | 1 |
| lmo2252 | LMRG_01579 | Aspartate aminotransferase (EC 2.6.1.1) | 0.42 | 1 |
| lmo0077 | LMRG_02328 | Pentapeptide repeat family protein | 0.43 | 1 |
| lmo0503 | LMRG_00184 | PTS system, galactitol-specific IIA component (EC 2.7.1.200) | 0.43 | 1 |
| lmo0510 | LMRG_00191 | hypothetical protein | 0.43 | 1 |
| lmo0722 | LMRG_00411 | Pyruvate dehydrogenase (quinone) (EC 1.2.5.1) | 0.43 | 1 |
| lmo0732 | LMRG_00420 | Internalin-like protein (LPXTG motif) Lmo0732 homolog | 0.43 | 1 |
| lmo1289 | LMRG_00739 | Internalin-like protein (LPXTG motif) Lmo1289 homolog | 0.43 | 1 |
| lmo1813 | LMRG_00960 | L-serine dehydratase, beta subunit (EC 4.3.1.17) | 0.44 | 1 |
| lmo1992 | LMRG_01140 | Alpha-acetolactate decarboxylase (EC 4.1.1.5) | 0.44 | 1 |
| lmo2050 | LMRG_01200 | Excinuclease ABC subunit A paralog of unknown function | 0.44 | 1 |
| lmo2817 | LMRG_01881 | N-acyl-L-amino acid amidohydrolase (EC 3.5.1.14) | 0.44 | 1 |
| lmo0112 | LMRG_02361 | hypothetical protein | 0.45 | 1 |
| lmo0374 | LMRG_00066 | PTS system, cellobiose-specific IIB component (EC 2.7.1.205) | 0.45 | 1 |
| lmo0116 | LMRG_02365 | LmaC, associated with virulence in Listeria | 0.46 | 1 |
| lmo1383 | LMRG_00835 | Isopentenyl-diphosphate delta-isomerase, FMN-dependent (EC 5.3.3.2) | 0.46 | 1 |
| lmo1758 | LMRG_02513 | DNA ligase (NAD(+)) (EC 6.5.1.2) | 0.46 | 1 |
| lmo2660 | LMRG_02205 | Transketolase (EC 2.2.1.1) | 0.46 | 1 |
| lmo0976 | LMRG_02076 | GNAT family acetyltransferase YjcF | 0.47 | 1 |
| lmo1588 | LMRG_01379 | N-acetylornithine aminotransferase (EC 2.6.1.11) | 0.47 | 1 |
| lmo1613 | LMRG_01353 | hypothetical protein | 0.47 | 1 |
| lmo2522 | LMRG_01726 | Cell wall-binding protein YocH | 0.47 | 1 |
| lmo0703 | LMRG_00392 | UDP-N-acetylenolpyruvoylglucosamine reductase (EC 1.3.1.98) | 0.48 | 1 |
| lmo2668 | LMRG_02213 | Predicted galactitol operon regulator (Transcriptional antiterminator), BglG family / PTS system, IIA component | 0.48 | 1 |
| lmo2849 | LMRG_02420 | Rhamnulokinase (EC 2.7.1.5) | 0.48 | 1 |
| lmor13 | LMRG_05513 | 5S rRNA ## 5S ribosomal RNA | 0.48 | 1 |
| lmo0118 | LMRG_02367 | hypothetical protein | 0.49 | 1 |
| lmo2132 | LMRG_02808 | hypothetical protein | 0.49 | 1 |
| lmo2251 | LMRG_01580 | Glutamine ABC transporter, ATP-binding protein GlnQ | 0.49 | 1 |
| lmo2264 | LMRG_01567 | UPF0750 membrane protein YitT | 0.49 | 1 |
| lmo2337 | LMRG_01506 | Transcriptional repressor of the fructose operon, DeoR family | 0.49 | 1 |
| lmo2411 | LMRG_01837 | Iron-sulfur cluster assembly protein SufB | 0.49 | 1 |
| lmo2485 | LMRG_01763 | Uncharacterized protein CA_C2659 | 0.49 | 1 |
| #N/A | LMRG_02576 | Type I restriction-modification system, DNA-methyltransferase subunit M (EC 2.1.1.72) | 0.49 | 1 |
| lmo0482 | LMRG_00163 | 23S rRNA (adenine(2503)-C(2))-methyltransferase @ tRNA (adenine(37)-C(2))-methyltransferase (EC 2.1.1.192) | 0.5 | 1 |
| lmo0552 | LMRG_00234 | cell surface protein precursor | 0.5 | 1 |
| lmo0580 | LMRG_00262 | Phospholipase/carboxylesterase family protein | 0.5 | 1 |
| lmo0593 | LMRG_00275 | Formate/nitrite family of transporters | 0.5 | 1 |
| lmo1033 | LMRG_00494 | Transketolase, C-terminal section (EC 2.2.1.1) | 0.5 | 1 |
| lmo2128 | LMRG_01282 | Maltose operon transcriptional repressor MalR, LacI family | 0.5 | 1 |
| lmo2470 | LMRG_01778 | Internalin-like protein Lmo2470 homolog | 0.5 | 1 |
| lmo2570 | LMRG_02698 | hypothetical protein | 0.5 | 1 |
| lmo2756 | LMRG_01940 | DNA topoisomerase III (EC 5.99.1.2) | 0.5 | 1 |
| lmo0970 | LMRG_02069 | Enoyl-[acyl-carrier-protein] reductase [NADH] (EC 1.3.1.9) | 0.51 | 1 |
| lmo1822 | LMRG_00969 | 16S rRNA (cytosine(967)-C(5))-methyltransferase (EC 2.1.1.176) | 0.51 | 1 |
| lmo1920 | LMRG_01067 | Uncharacterized protein YpjA | 0.51 | 1 |
| lmo2055 | LMRG_01205 | ComK regulator YlbF | 0.51 | 1 |
| lmo2420 | LMRG_01828 | hypothetical protein | 0.51 | 1 |
| lmo2447 | LMRG_01801 | transcriptional activator | 0.51 | 1 |
| lmo0001 | LMRG_02429 | Chromosomal replication initiator protein DnaA | 0.52 | 1 |
| lmo0182 | LMRG_02747 | glycosyl hydrolase, family 31 | 0.52 | 1 |
| lmo0991 | LMRG_02091 | Membrane protein | 0.52 | 1 |
| lmo1066 | LMRG_00528 | Inositol-1-monophosphatase (EC 3.1.3.25) | 0.52 | 1 |
| lmo2013 | LMRG_01161 | hypothetical protein | 0.52 | 1 |
| lmo0331 | LMRG_00023 | Internalin-like protein (LPXTG motif) Lmo0331 homolog | 0.53 | 1 |
| lmo0520 | LMRG_00201 | N-acetylglucosamine kinase bacterial type predicted (EC 2.7.1.59) homolog / Transcriptional regulator | 0.53 | 1 |
| lmo0623 | LMRG_00306 | hypothetical protein | 0.53 | 1 |
| lmo2006 | LMRG_01154 | Acetolactate synthase, catabolic (EC 2.2.1.6) | 0.53 | 1 |
| lmo2510 | LMRG_01738 | Protein translocase subunit SecA | 0.53 | 1 |
| #N/A | LMRG_00279 | hypothetical protein | 0.53 | 1 |
| lmo0332 | LMRG_00024 | hypothetical protein | 0.54 | 1 |
| lmo0796 | LMRG_00484 | YceI like family protein | 0.54 | 1 |
| lmo0997 | LMRG_02097 | ATP-dependent Clp protease, ATP-binding subunit ClpE | 0.54 | 1 |
| lmo1321 | LMRG_00771 | Bacterial ribosome SSU maturation protein RimP | 0.54 | 1 |
| lmo1576 | LMRG_01391 | Uncharacterized protein YtoI | 0.54 | 1 |
| lmo2205 | LMRG_01627 | Phosphoglycerate mutase (EC 5.4.2.11) | 0.54 | 1 |
| lmor10 | LMRG_05510 | 5S rRNA ## 5S ribosomal RNA | 0.54 | 1 |
| lmo0333 | LMRG_00025 | Internalin-like protein (LPXTG motif) Lmo0333 homolog | 0.55 | 1 |
| lmo0335 | LMRG_00027 | hypothetical protein | 0.55 | 1 |
| lmo0391 | LMRG_00084 | hypothetical protein | 0.55 | 1 |
| lmo0880 | LMRG_02304 | hypothetical protein | 0.55 | 1 |
| lmo0922 | LMRG_02022 | Pantothenate kinase (EC 2.7.1.33) | 0.55 | 1 |
| lmo1405 | LMRG_00857 | Glycerol-3-phosphate responsive antiterminator (mRNA-binding) | 0.55 | 1 |
| lmo1563 | LMRG_01404 | Dephospho-CoA kinase (EC 2.7.1.24) | 0.55 | 1 |
| lmo1985 | LMRG_01133 | Acetolactate synthase small subunit (EC 2.2.1.6) | 0.55 | 1 |
| lmo2262 | LMRG_01569 | Putative esterase YitV | 0.55 | 1 |
| lmo2500 | LMRG_01748 | Phosphate regulon sensor protein PhoR (SphS) (EC 2.7.13.3) | 0.55 | 1 |
| lmo2637 | LMRG_02182 | Putative pheromone precursor lipoprotein, related to Cad | 0.55 | 1 |
| lmo0075 | LMRG_02326 | Probable carboxyvinyl-carboxyphosphonate phosphorylmutase (EC 2.7.8.23) | 0.56 | 1 |
| lmo0215 | LMRG_02637 | FIG006789: Stage V sporulation protein | 0.56 | 1 |
| lmo0363 | LMRG_00055 | Alpha-aspartyl dipeptidase Peptidase E (EC 3.4.13.21) | 0.56 | 1 |
| lmo0540 | LMRG_00222 | Putative penicillin-binding protein PbpX | 0.56 | 1 |
| lmo0604 | LMRG_00287 | hypothetical protein | 0.56 | 1 |
| lmo0639 | LMRG_00325 | Transcriptional regulator | 0.56 | 1 |
| lmo0762 | LMRG_00450 | GTP-binding protein related to HflX | 0.56 | 1 |
| lmo1296 | LMRG_02975 | Ribosome LSU-associated GTP-binding protein HflX | 0.56 | 1 |
| lmo1357 | LMRG_00807 | Biotin carboxylase of acetyl-CoA carboxylase (EC 6.3.4.14) | 0.56 | 1 |
| lmo1694 | LMRG_02768 | Cell division inhibitor Slr1223 (YfcH in EC), contains epimerase/dehydratase and DUF1731 domains | 0.56 | 1 |
| lmo2526 | LMRG_01722 | UDP-N-acetylglucosamine 1-carboxyvinyltransferase (EC 2.5.1.7) | 0.56 | 1 |
| lmo2845 | LMRG_01853 | Uncharacterized MFS-type transporter | 0.56 | 1 |
| lmo0484 | LMRG_00165 | Heme-degrading monooxygenase, staphylobilin-producing (EC 1.14.99.48) | 0.57 | 1 |
| lmo1709 | LMRG_02561 | Methionine aminopeptidase (EC 3.4.11.18) | 0.57 | 1 |
| lmo2062 | LMRG_01212 | Copper resistance protein CopC / Copper resistance protein CopD | 0.57 | 1 |
| lmo2529 | LMRG_01719 | ATP synthase beta chain (EC 3.6.3.14) | 0.57 | 1 |
| lmo0199 | LMRG_02621 | Ribose-phosphate pyrophosphokinase (EC 2.7.6.1) | 0.58 | 1 |
| lmo0360a | LMRG_00052 | hypothetical protein | 0.58 | 1 |
| lmo0599 | LMRG_00282 | Transcriptional regulator, PadR family | 0.58 | 1 |
| lmo0679 | LMRG_00367 | Flagellar biosynthesis protein FlhB | 0.58 | 1 |
| lmo0742 | LMRG_00430 | ABC transporter, ATP-binding protein | 0.58 | 1 |
| lmo1202 | LMRG_00648 | Sirohydrochlorin cobaltochelatase CbiK (EC 4.99.1.3) @ Sirohydrochlorin ferrochelatase activity of CbiK (EC 4.99.1.4) | 0.58 | 1 |
| lmo1687 | LMRG_02761 | DUF402 family nucleoside diphosphatase | 0.58 | 1 |
| lmo1712 | LMRG_02959 | hypothetical protein | 0.58 | 1 |
| lmo1890 | LMRG_01037 | hyothetical protein | 0.58 | 1 |
| lmor06 | LMRG_05506 | 5S rRNA ## 5S ribosomal RNA | 0.58 | 1 |
| lmo0152 | LMRG_02397 | Oligopeptide ABC transporter, substrate-binding protein OppA (TC 3.A.1.5.1) | 0.59 | 1 |
| lmo2101 | LMRG_01252 | Pyridoxal 5'-phosphate synthase (glutamine hydrolyzing), synthase subunit (EC 4.3.3.6) | 0.59 | 1 |
| lmo2516 | LMRG_01732 | FIG000605: protein co-occurring with transport systems (COG1739) | 0.59 | 1 |
| lmo2555 | LMRG_01692 | Glycosyltransferase LafA, responsible for the formation of Glc-DAG | 0.59 | 1 |
| lmo0667 | LMRG_00354 | Uncharacterized efflux ABC transporter, ATP-binding protein YadG | 0.6 | 1 |
| lmo1974 | LMRG_01121 | Transcriptional regulator, GntR family | 0.6 | 1 |
| lmo2107 | LMRG_01261 | Transcriptional repressor GlcR, DeoR family | 0.6 | 1 |
| lmo0440 | LMRG_00132 | hypothetical protein | 0.61 | 1 |
| lmo2110 | LMRG_01264 | Mannose-6-phosphate isomerase (EC 5.3.1.8) | 0.61 | 1 |
| lmo2160 | LMRG_01672 | Xylose isomerase-like TIM-barrel protein SAV0219 | 0.61 | 1 |
| lmo2694 | LMRG_02241 | Arginine decarboxylase (EC 4.1.1.19) | 0.61 | 1 |
| lmo0948 | LMRG_02047 | Transcriptional regulator, GntR family | 0.62 | 1 |
| lmo1578 | LMRG_01389 | Proline dipeptidase (EC 3.4.13.9) | 0.62 | 1 |
| lmo1864 | LMRG_01011 | FIG01964566: Predicted membrane protein, hemolysin III homolog | 0.62 | 1 |
| lmo2579a | LMRG_02688 | internalin H | 0.62 | 1 |
| lmo2762 | LMRG_01933 | PTS system, cellobiose-specific IIB component (EC 2.7.1.205) | 0.62 | 1 |
| lmo0448 | LMRG_00140 | Probable glutamate/gamma-aminobutyrate antiporter | 0.63 | 1 |
| lmo1337 | LMRG_00787 | FIG056164: rhomboid family serine protease | 0.63 | 1 |
| lmo1391 | LMRG_00843 | Purine nucleoside ABC transporter, permease protein 2 | 0.63 | 1 |
| lmo1728 | LMRG_02543 | 1,2-beta-oligoglucan phosphorylase (EC 2.4.1.333) | 0.63 | 1 |
| lmo2172 | LMRG_01660 | Coenzyme A transferase | 0.63 | 1 |
| lmo2231 | LMRG_01601 | Cobalt-zinc-cadmium resistance protein | 0.63 | 1 |
| lmo2669 | LMRG_02214 | Uncharacterized DUF1113 membrane protein family | 0.63 | 1 |
| lmo0269 | LMRG_02607 | Oligopeptide ABC transporter, permease protein OppC (TC 3.A.1.5.1) | 0.64 | 1 |
| lmo0370 | LMRG_00062 | Protein PhnA | 0.64 | 1 |
| lmo0430 | LMRG_00122 | Transcriptional regulator, LysR family | 0.64 | 1 |
| lmo0832 | LMRG_02255 | transposase OrfA, IS3 family | 0.64 | 1 |
| lmo1335 | LMRG_00785 | LSU ribosomal protein L33p @ LSU ribosomal protein L33p, zinc-dependent | 0.64 | 1 |
| lmo2375 | LMRG_02720 | hypothetical protein | 0.64 | 1 |
| lmor16 | LMRG_05516 | 5S rRNA ## 5S ribosomal RNA | 0.64 | 1 |
| lmo0334 | LMRG_00026 | hypothetical protein | 0.65 | 1 |
| lmo0495 | LMRG_00176 | Permease of the drug/metabolite transporter (DMT) superfamily | 0.65 | 1 |
| lmo1533 | LMRG_01437 | Holliday junction ATP-dependent DNA helicase RuvA (EC 3.6.4.12) | 0.65 | 1 |
| lmo1689 | LMRG_02763 | A/G-specific adenine glycosylase (EC 3.2.2.-) | 0.65 | 1 |
| lmo2146 | LMRG_01686 | Transcriptional activator CysL, LysR family | 0.65 | 1 |
| lmo2567 | LMRG_02701 | hypothetical protein | 0.65 | 1 |
| lmo0166 | LMRG_02411 | UPF0213 protein YazA | 0.66 | 1 |
| lmo0951 | LMRG_02050 | Cell surface hydrolase, membrane-bound | 0.66 | 1 |
| lmo1032 | LMRG_00493 | Transketolase, N-terminal section (EC 2.2.1.1) | 0.66 | 1 |
| lmo1452 | LMRG_00904 | GTP cyclohydrolase 1 type 2 homolog YbgI | 0.66 | 1 |
| lmo1666 | LMRG_01301 | hypothetical protein | 0.66 | 1 |
| lmo2422 | LMRG_01826 | Two-component transcriptional response regulator, OmpR family | 0.66 | 1 |
| lmo0560 | LMRG_00242 | NADP-specific glutamate dehydrogenase (EC 1.4.1.4) | 0.67 | 1 |
| lmo1579 | LMRG_01388 | Alanine dehydrogenase (EC 1.4.1.1) | 0.67 | 1 |
| lmo1746 | LMRG_02525 | ABC transporter-like sensor and permease protein | 0.67 | 1 |
| lmo1627 | LMRG_01339 | Tryptophan synthase alpha chain (EC 4.2.1.20) | 0.68 | 1 |
| lmo1876 | LMRG_01023 | Formate--tetrahydrofolate ligase (EC 6.3.4.3) | 0.68 | 1 |
| lmo2068 | LMRG_01218 | Heat shock protein 60 kDa family chaperone GroEL | 0.68 | 1 |
| lmo2240 | LMRG_01592 | Efflux ABC transporter, ATP-binding protein | 0.68 | 1 |
| lmo2338 | LMRG_01505 | Aminopeptidase C (EC 3.4.22.40) | 0.68 | 1 |
| lmo2362 | LMRG_01480 | Probable glutamate/gamma-aminobutyrate antiporter | 0.68 | 1 |
| lmot13 | LMRG_05013 | tRNA-Ser-GCT | 0.68 | 1 |
| lmo0209 | LMRG_02631 | virulence cluster protein A VclA | 0.7 | 1 |
| lmo1261 | LMRG_00710 | Membrane protein | 0.7 | 1 |
| lmo1406 | LMRG_00858 | Pyruvate formate-lyase (EC 2.3.1.54) | 0.7 | 1 |
| lmo1763 | LMRG_02508 | His repressor | 0.7 | 1 |
| lmo2288 | LMRG_01544 | Phage protein | 0.7 | 1 |
| lmo2452 | LMRG_01796 | Carboxylesterase (EC 3.1.1.1) | 0.7 | 1 |
| lmo2541 | LMRG_01706 | Threonylcarbamoyl-AMP synthase (EC 2.7.7.87) / SUA5 domain with internal deletion | 0.7 | 1 |
| lmo0348 | LMRG_00039 | Phosphoenolpyruvate-dihydroxyacetone phosphotransferase (EC 2.7.1.121), dihydroxyacetone binding subunit DhaK | 0.71 | 1 |
| lmo2197 | LMRG_01635 | hypothetical protein | 0.71 | 1 |
| lmo2731 | LMRG_01965 | Transcription regulator, RpiR family | 0.71 | 1 |
| lmo0050 | LMRG_02479 | Accessory gene regulator protein C | 0.72 | 1 |
| lmo0408 | LMRG_00101 | hypothetical protein | 0.72 | 1 |
| lmo1451 | LMRG_00903 | 4-hydroxy-3-methylbut-2-enyl diphosphate reductase (EC 1.17.7.4) | 0.72 | 1 |
| lmo2179 | LMRG_01653 | Putative peptidoglycan bound protein (LPXTG motif) Lmo2179 homolog | 0.72 | 1 |
| lmo0764 | LMRG_00452 | Lipoate-protein ligase A | 0.73 | 1 |
| lmo1158 | LMRG_00601 | Propanediol utilization polyhedral body protein PduK | 0.73 | 1 |
| lmo1184 | LMRG_00630 | Ethanolamine utilization polyhedral-body-like protein EutN | 0.73 | 1 |
| lmo1482 | LMRG_00935 | DNA internalization-related competence protein ComEC/Rec2 | 0.73 | 1 |
| lmo2436 | LMRG_01812 | Beta-glucoside bgl operon antiterminator, BglG family | 0.73 | 1 |
| lmo0168 | LMRG_02413 | Transition state regulatory protein AbrB | 0.74 | 1 |
| lmo0032 | LMRG_02461 | ROK family sugar kinase or transcriptional regulator | 0.75 | 1 |
| lmo0396 | LMRG_00089 | Pyrroline-5-carboxylate reductase (EC 1.5.1.2) | 0.75 | 1 |
| lmo1528 | LMRG_01442 | hypothetical protein | 0.75 | 1 |
| lmo1637 | LMRG_01329 | ABC transporter, permease protein | 0.75 | 1 |
| lmo2147 | LMRG_01685 | Putative membrane protein YeiH | 0.75 | 1 |
| lmo2511 | LMRG_01737 | Ribosome hibernation promoting factor Hpf | 0.75 | 1 |
| lmo0386 | LMRG_02971 | 3D-(3,5/4)-trihydroxycyclohexane-1,2-dione hydrolase (EC 3.7.1.22) | 0.76 | 1 |
| lmo0395 | LMRG_00088 | Acetyltransferase, GNAT family | 0.76 | 1 |
| lmo1604 | LMRG_01363 | Alkyl hydroperoxide reductase subunit C-like protein | 0.76 | 1 |
| lmo1649 | LMRG_01318 | hypothetical protein | 0.76 | 1 |
| lmo2265 | LMRG_01566 | Uncharacterized protein UPF0344 | 0.76 | 1 |
| lmo2401 | LMRG_01847 | Hypothetical NagD-like phosphatase | 0.76 | 1 |
| lmo1073 | LMRG_00535 | Vitamin B12 ABC transporter, substrate-binding protein BtuF | 0.77 | 1 |
| lmo1925 | LMRG_01072 | Biosynthetic Aromatic amino acid aminotransferase beta (EC 2.6.1.57) @ Histidinol-phosphate aminotransferase (EC 2.6.1.9) | 0.77 | 1 |
| lmo0492 | LMRG_00173 | Transcriptional regulator, LysR family | 0.78 | 1 |
| lmo0502 | LMRG_00183 | Polysialic acid capsule expression protein KpsF | 0.78 | 1 |
| lmo1171 | LMRG_00617 | Ethanol dehydrogenase [NAD+] | 0.78 | 1 |
| lmo1416 | LMRG_00868 | Uncharacterized membrane spanning protein, contains VanZ-like domain | 0.78 | 1 |
| lmo1698 | LMRG_02772 | Ribosomal-protein-S5p-alanine acetyltransferase (EC 2.3.1.128) | 0.78 | 1 |
| lmo0108 | LMRG_02357 | Heterodimeric efflux ABC transporter, permease/ATP-binding subunit 1 | 0.79 | 1 |
| lmo0304 | LMRG_00001 | hypothetical protein | 0.79 | 1 |
| lmo1297 | LMRG_02856 | Cystathionine beta-lyase family protein involved in aluminum resistance | 0.79 | 1 |
| lmo1419 | LMRG_00871 | UPF0118 membrane protein YdbI | 0.79 | 1 |
| lmo2000 | LMRG_01148 | PTS system, mannose-specific IID component | 0.79 | 1 |
| lmo2488 | LMRG_01760 | Excinuclease ABC subunit A | 0.79 | 1 |
| lmo1162 | LMRG_00605 | Propanediol utilization protein PduM | 0.8 | 1 |
| lmo1443 | LMRG_00895 | hypothetical protein | 0.8 | 1 |
| lmo2038 | LMRG_01187 | UDP-N-acetylmuramoyl-dipeptide--2,6-diaminopimelate ligase (EC 6.3.2.13) | 0.8 | 1 |
| lmo0927 | LMRG_02026 | Lipoteichoic acid synthase LtaS Type Ia | 0.81 | 1 |
| lmo1080 | LMRG_00542 | Glycosyl transferase | 0.81 | 1 |
| lmo1584 | LMRG_01383 | BH3197 unknown conserved protein | 0.81 | 1 |
| lmo1918 | LMRG_01065 | Uncharacterized flavin-containing protein YtfP | 0.81 | 1 |
| lmo0183 | LMRG_02748 | Alpha-glucosidases, family 31 of glycosyl hydrolases | 0.82 | 1 |
| lmo0610 | LMRG_00293 | Internalin-like protein (LPXTG motif) Lmo0610 homolog | 0.82 | 1 |
| lmo0646 | LMRG_00333 | Glyoxalase family protein | 0.82 | 1 |
| lmo1234 | LMRG_00680 | Excinuclease ABC subunit C | 0.82 | 1 |
| lmo2082 | LMRG_01233 | Fluoride ion transporter CrcB | 0.82 | 1 |
| lmo0024 | LMRG_02453 | PTS system, mannose/fructose/sorbose family, IID component | 0.83 | 1 |
| lmo0417 | LMRG_00109 | hypothetical protein | 0.83 | 1 |
| lmo0450 | LMRG_00142 | hypothetical protein | 0.83 | 1 |
| lmo1116 | LMRG_00559 | Transcriptional regulator, AraC family | 0.83 | 1 |
| lmo1392 | LMRG_00844 | FIG001621: Zinc protease | 0.83 | 1 |
| lmo1792 | LMRG_02816 | tRNA (guanine(37)-N(1))-methyltransferase (EC 2.1.1.228) | 0.83 | 1 |
| lmo2214 | LMRG_01618 | ABC transporter, permease protein EscB | 0.83 | 1 |
| lmo2503 | LMRG_01745 | Cardiolipin synthase, bacterial type ClsA | 0.83 | 1 |
| lmo0283 | LMRG_02588 | Methionine ABC transporter permease protein | 0.84 | 1 |
| lmo0992 | LMRG_02092 | Membrane protein | 0.84 | 1 |
| lmo1605 | LMRG_01361 | UDP-N-acetylmuramate--L-alanine ligase (EC 6.3.2.8) | 0.84 | 1 |
| lmo2370 | LMRG_02715 | Cystathionine beta-lyase (EC 4.4.1.8) | 0.84 | 1 |
| lmo2552 | LMRG_01695 | UDP-N-acetylglucosamine 1-carboxyvinyltransferase (EC 2.5.1.7) | 0.84 | 1 |
| lmo2665 | LMRG_02210 | PTS system, galactitol-specific IIC component | 0.84 | 1 |
| lmo0431 | LMRG_00123 | Acetyltransferase (isoleucine patch superfamily) | 0.85 | 1 |
| lmo0535 | LMRG_00217 | Transcriptional regulator, LacI family | 0.85 | 1 |
| lmo2109 | LMRG_01263 | Hydrolase, alpha/beta fold family | 0.85 | 1 |
| lmo2166 | LMRG_01666 | cyclic nucleotide-binding protein | 0.85 | 1 |
| lmo2286 | LMRG_01546 | Putative tail or base plate protein gp17 [Bacteriophage A118] | 0.85 | 1 |
| lmo2518 | LMRG_01730 | Cell envelope-associated transcriptional attenuator LytR-CpsA-Psr, subfamily F2 | 0.85 | 1 |
| lmos20 | #N/A | FIG00774325: hypothetical protein | 0.85 | 1 |
| lmo0261 | LMRG_02648 | 6-phospho-beta-glucosidase (EC 3.2.1.86) | 0.86 | 1 |
| lmo1413 | LMRG_00865 | Putative peptidoglycan bound protein (LPXTG motif) Lmo1413 homolog | 0.86 | 1 |
| lmo2037 | LMRG_01186 | Phospho-N-acetylmuramoyl-pentapeptide-transferase (EC 2.7.8.13) | 0.87 | 1 |
| #N/A | LMRG_01254 | FIG00774739: hypothetical protein | 0.87 | 1 |
| lmo1684 | LMRG_02758 | Phosphoglycerate dehydrogenase and related dehydrogenases | 0.88 | 1 |
| lmo2396 | LMRG_01852 | Internalin-like protein (LPXTG motif) Lmo2396 homolog | 0.88 | 1 |
| lmo0918 | LMRG_02018 | PRD/PTS system IIA 2 domain protein | 0.89 | 1 |
| lmo2026 | LMRG_01175 | Internalin-like protein (LPXTG motif) Lmo2026 homolog | 0.89 | 1 |
| lmo2323 | LMRG_01521 | Protein gp43 [Bacteriophage A118] | 0.89 | 1 |
| lmo2479 | LMRG_01769 | TPR-repeat-containing protein | 0.89 | 1 |
| lmo0303 | LMRG_02565 | Lipoprotein | 0.9 | 1 |
| lmo0547 | LMRG_00229 | Sorbitol operon transcription regulator | 0.9 | 1 |
| lmo0932 | LMRG_02031 | Uncharacterized membrane protein YhjE | 0.9 | 1 |
| lmo1597 | LMRG_01370 | hypothetical protein | 0.9 | 1 |
| lmo1616 | LMRG_01350 | Putative phosphotransferase YtmP | 0.9 | 1 |
| lmo1706 | LMRG_02958 | Inner membrane protein YihY, formerly thought to be RNase BN | 0.9 | 1 |
| lmo2039 | LMRG_01188 | Cell division protein FtsI [Peptidoglycan synthetase] (EC 2.4.1.129) | 0.9 | 1 |
| lmo2148 | LMRG_01684 | hypothetical protein | 0.9 | 1 |
| lmo0158 | LMRG_02403 | Sugar phosphatase YidA (EC 3.1.3.23) | 0.91 | 1 |
| lmo0618 | LMRG_00301 | Probable serine/threonine-protein kinase pknB (EC 2.7.11.1) | 0.91 | 1 |
| lmo0626 | LMRG_00309 | Uncharacterized membrane protein YkoS | 0.91 | 1 |
| lmo1403 | LMRG_00855 | DNA mismatch repair protein MutS | 0.91 | 1 |
| lmo1639 | LMRG_01327 | DNA-3-methyladenine glycosylase (EC 3.2.2.20) | 0.91 | 1 |
| lmo2433 | LMRG_01815 | tributyrin esterase | 0.91 | 1 |
| lmo1067 | LMRG_00529 | GTP-binding protein TypA/BipA | 0.92 | 1 |
| lmo2640 | LMRG_02185 | Heptaprenyl diphosphate synthase component I (EC 2.5.1.30) | 0.92 | 1 |
| lmo0211 | LMRG_02633 | LSU ribosomal protein L25p | 0.93 | 1 |
| lmo1311 | LMRG_00761 | hypothetical protein | 0.93 | 1 |
| lmo1508 | LMRG_01462 | Sensor histidine kinase YclK | 0.93 | 1 |
| lmo2484 | LMRG_01764 | Uncharacterized membrane protein YvlD | 0.93 | 1 |
| lmo0912 | LMRG_02012 | Formate efflux transporter FocA | 0.94 | 1 |
| lmo1626 | LMRG_01340 | hypothetical protein | 0.94 | 1 |
| lmo0356 | LMRG_00047 | Uncharacterized oxidoreductase YhhX | 0.95 | 1 |
| lmo1345 | LMRG_00795 | Late competence protein ComGC, access of DNA to ComEA, FIG007487 | 0.95 | 1 |
| lmo2059 | LMRG_01209 | Potassium voltage-gated channel subfamily KQT | 0.95 | 1 |
| lmo0113 | LMRG_02362 | Phage protein | 0.96 | 1 |
| lmo0900 | LMRG_02843 | hypothetical protein | 0.96 | 1 |
| lmo1290 | LMRG_00740 | Internalin-like protein (LPXTG motif) Lmo1290 homolog | 0.96 | 1 |
| lmo1733 | LMRG_02538 | Glutamate synthase [NADPH] small chain (EC 1.4.1.13) | 0.96 | 1 |
| lmo0601 | LMRG_00284 | hypothetical protein | 0.97 | 1 |
| lmo0826 | LMRG_02251 | Sodium-dependent phosphate transporter | 0.97 | 1 |
| lmo1019 | LMRG_02119 | Uncharacterized S1 RNA binding domain protein YitL | 0.97 | 1 |
| lmo1707 | LMRG_02563 | Hypothetical protein YfkK | 0.97 | 1 |
| lmo2361 | LMRG_01481 | Rrf2 family transcriptional regulator | 0.97 | 1 |
| lmos68 | #N/A | hypothetical protein | 0.97 | 1 |
| lmo0612 | LMRG_00295 | Transcriptional regulator, MarR family | 0.98 | 1 |
| lmo0708 | LMRG_00397 | Flagellar biosynthesis protein FliS | 0.98 | 1 |
| lmo0837 | LMRG_02260 | Efflux ABC transporter, permease/ATP-binding protein YwjA | 0.98 | 1 |
| lmo0449 | LMRG_00141 | Membrane protein | 0.99 | 1 |
| lmo1937 | LMRG_01084 | GTP-binding protein EngA | 0.99 | 1 |
| lmo2108 | LMRG_01262 | N-acetylglucosamine-6-phosphate deacetylase (EC 3.5.1.25) | 0.99 | 1 |
| lmo2270 | LMRG_01560 | Competence transcription factor | 0.99 | 1 |
| lmo0185 | LMRG_02750 | Uncharacterized metal-dependent hydrolase YcfH | 1 | 1 |
| lmo1231 | LMRG_00677 | DNA polymerase X family | 1 | 1 |
| lmo1367 | LMRG_00817 | Arginine pathway regulatory protein ArgR, repressor of arg regulon | 1 | 1 |
| lmo1863 | LMRG_01010 | DegV family protein | 1 | 1 |
| lmo0263 | LMRG_02646 | Internalin H (LPXTG motif) | 1.02 | 1 |
| lmo0497 | LMRG_00178 | Glycosyl transferase | 1.02 | 1 |
| lmo1241 | LMRG_00687 | hypothetical protein | 1.02 | 1 |
| lmo1355 | LMRG_00805 | Translation elongation factor P | 1.02 | 1 |
| lmo1399 | LMRG_00851 | Ribonuclease Y | 1.02 | 1 |
| lmo2583 | LMRG_02684 | Two-component response regulator colocalized with HrtAB transporter | 1.02 | 1 |
| lmo0576 | LMRG_00258 | Cell wall surface anchor family protein | 1.03 | 1 |
| lmo0695 | LMRG_00384 | hypothetical protein | 1.03 | 1 |
| lmo1026 | LMRG_02126 | Cell envelope-associated transcriptional attenuator LytR-CpsA-Psr, subfamily F2 | 1.03 | 1 |
| lmo1634 | LMRG_01332 | Acetaldehyde dehydrogenase (EC 1.2.1.10) / Alcohol dehydrogenase (EC 1.1.1.1) | 1.03 | 1 |
| lmo1979 | LMRG_01127 | Uncharacterized DUF1694 domain-containing protein YueI | 1.03 | 1 |
| lmo2710 | LMRG_01987 | hypothetical protein | 1.03 | 1 |
| lmo0740 | LMRG_00428 | hypothetical protein | 1.04 | 1 |
| lmo0752 | LMRG_00440 | Hydrolase/acyltransferase in BltB locus | 1.04 | 1 |
| lmo1440 | LMRG_00892 | Uncharacterized membrane protein YqgB | 1.04 | 1 |
| lmo1581 | LMRG_01386 | Acetate kinase (EC 2.7.2.1) | 1.04 | 1 |
| lmo2802 | LMRG_01895 | 16S rRNA (guanine(527)-N(7))-methyltransferase (EC 2.1.1.170) | 1.04 | 1 |
| lmo0186 | LMRG_02751 | Uncharacterized protein YabE | 1.05 | 1 |
| lmo0276 | LMRG_02599 | Hydrolase, haloacid dehalogenase-like family | 1.05 | 1 |
| lmo0337 | LMRG_00029 | hypothetical protein | 1.05 | 1 |
| lmo0376 | LMRG_00068 | Transcriptional regulator, YafY family | 1.05 | 1 |
| lmo2239 | LMRG_01593 | Membrane protein | 1.05 | 1 |
| lmo0277 | LMRG_02598 | Oxidoreductase, Gfo/Idh/MocA family | 1.06 | 1 |
| lmo0336 | LMRG_00028 | hypothetical protein | 1.06 | 1 |
| lmo1060 | LMRG_00522 | Two-component transcriptional response regulator, OmpR family | 1.06 | 1 |
| lmo1270 | LMRG_00720 | Signal peptidase I (EC 3.4.21.89) | 1.06 | 1 |
| lmo1307 | LMRG_00757 | hypothetical protein | 1.06 | 1 |
| lmo1946 | LMRG_01093 | Acyl-CoA hydrolase (EC 3.1.2.20) | 1.06 | 1 |
| lmo2430 | LMRG_01818 | Ferrichrome transport system permease protein FhuB | 1.06 | 1 |
| lmo0097 | LMRG_02346 | PTS system, mannose-specific IIC component | 1.07 | 1 |
| lmo1211 | LMRG_00657 | Membrane protein | 1.07 | 1 |
| lmo1535 | LMRG_01435 | Probable transcriptional regulatory protein YebC | 1.07 | 1 |
| lmo2782 | LMRG_01915 | PTS system, cellobiose-specific IIB component (EC 2.7.1.205) | 1.07 | 1 |
| lmo1182 | LMRG_00628 | Ethanolamine utilization protein similar to PduL | 1.08 | 1 |
| lmo1444 | LMRG_00896 | Foldase protein PrsA precursor (EC 5.2.1.8) | 1.09 | 1 |
| lmo2294 | LMRG_01538 | gp9 | 1.09 | 1 |
| lmo2644a | LMRG_02190 | hypothetical protein | 1.09 | 1 |
| #N/A | LMRG_02594 | ABC transporter, ATP-binding protein | 1.09 | 1 |
| lmo1123 | LMRG_00565 | hypothetical protein | 1.1 | 1 |
| lmo0265 | LMRG_02611 | Uncharacterized deacetylase | 1.11 | 1 |
| lmo0909 | LMRG_02009 | Transcriptional regulator, GntR family | 1.11 | 1 |
| lmo2819 | LMRG_01879 | N-acyl-L-amino acid amidohydrolase (EC 3.5.1.14) | 1.11 | 1 |
| lmo0607 | LMRG_00290 | Efflux ABC transporter, permease/ATP-binding protein EF2593 | 1.12 | 1 |
| lmo0757 | LMRG_00445 | ABC transporter permease YhcI | 1.12 | 1 |
| lmo1244 | LMRG_00690 | Phosphoglycerate mutase family protein | 1.12 | 1 |
| lmo1288 | LMRG_00738 | S-ribosylhomocysteine lyase (EC 4.4.1.21) @ Autoinducer-2 production protein LuxS | 1.12 | 1 |
| lmo2189 | LMRG_01643 | Competence protein CoiA | 1.12 | 1 |
| lmo1340 | LMRG_00790 | Lipoprotein | 1.13 | 1 |
| lmo1583 | LMRG_01384 | Thiol peroxidase, Tpx-type (EC 1.11.1.15) | 1.13 | 1 |
| lmo1791 | LMRG_02815 | hypothetical protein | 1.13 | 1 |
| lmo1828 | LMRG_00975 | UPF0701 protein YicC | 1.13 | 1 |
| lmo1896 | LMRG_01043 | Asparaginyl-tRNA synthetase (EC 6.1.1.22) | 1.13 | 1 |
| lmo0083 | LMRG_02331 | Transcriptional regulator, MerR family | 1.14 | 1 |
| lmo0170 | LMRG_02415 | hypothetical protein | 1.14 | 1 |
| lmo1235 | LMRG_00681 | Aspartokinase (EC 2.7.2.4) | 1.14 | 1 |
| lmo1265 | LMRG_00714 | hypothetical protein | 1.14 | 1 |
| lmo2753 | LMRG_01943 | Lin2896 protein | 1.14 | 1 |
| lmo0176 | LMRG_02741 | Glucose:proton symporter GlcU | 1.15 | 1 |
| lmo1131 | LMRG_00573 | Heterodimeric efflux ABC transporter, permease/ATP-binding subunit 1 | 1.15 | 1 |
| lmo0171 | LMRG_02416 | Internalin-like protein (LPXTG motif) Lmo0171 homolog | 1.16 | 1 |
| lmo0541 | LMRG_00223 | ABC transporter, substrate-binding protein (cluster 8, B12/iron complex) | 1.16 | 1 |
| lmo0772 | LMRG_00460 | Transcriptional regulator, GntR family | 1.16 | 1 |
| lmo0857 | LMRG_02280 | Hydrolase, alpha/beta fold family | 1.16 | 1 |
| lmo1900 | LMRG_01047 | Aspartate 1-decarboxylase (EC 4.1.1.11) | 1.16 | 1 |
| lmo1964 | LMRG_01111 | ABC transporter, ATP-binding protein | 1.16 | 1 |
| lmo2835 | LMRG_01863 | Sugar phosphate isomerases/epimerases family protein YcjR | 1.16 | 1 |
| lmo0382 | LMRG_00075 | Transcriptional repressor of the myo-inositol catabolic operon DeoR family | 1.17 | 1 |
| lmo1076 | LMRG_00538 | N-acetylglucosaminidase (EC 3.2.1.96) Auto | 1.17 | 1 |
| lmo1271 | LMRG_00721 | Signal peptidase I (EC 3.4.21.89) | 1.17 | 1 |
| lmo1642 | LMRG_01324 | STAS domain protein | 1.17 | 1 |
| lmo2274 | LMRG_01556 | Phage protein | 1.17 | 1 |
| lmo2798 | LMRG_01899 | Hydrolase, haloacid dehalogenase-like family | 1.17 | 1 |
| lmo0744 | LMRG_00432 | Macrolide export ATP-binding/permease protein | 1.18 | 1 |
| lmo1947 | LMRG_01094 | Sensor histidine kinase ResE (EC 2.7.13.3) | 1.18 | 1 |
| lmo0908 | LMRG_02008 | UPF0118 membrane protein YueF | 1.19 | 1 |
| lmo2658 | LMRG_02203 | Diamine acetyltransferase (EC 2.3.1.57) | 1.19 | 1 |
| lmo0939 | LMRG_02038 | hypothetical protein | 1.2 | 1 |
| lmo2706 | LMRG_01991 | hypothetical protein | 1.2 | 1 |
| lmo0825 | LMRG_02250 | Hydroxymethylglutaryl-CoA reductase (EC 1.1.1.34) | 1.21 | 1 |
| lmo1250 | LMRG_00696 | Uncharacterized MFS-type transporter | 1.21 | 1 |
| lmo2016 | LMRG_01165 | Cold shock protein of CSP family | 1.21 | 1 |
| lmo1510 | LMRG_01460 | TPR domain protein | 1.22 | 1 |
| lmo0151 | LMRG_02396 | FIG00775619: hypothetical protein | 1.23 | 1 |
| lmo0803 | LMRG_02794 | Na+/H+ antiporter | 1.23 | 1 |
| lmo1432 | LMRG_00884 | hypothetical protein | 1.23 | 1 |
| lmo0321 | LMRG_00013 | Membrane protein | 1.24 | 1 |
| lmo0889 | LMRG_02313 | RsbT co-antagonist protein RsbRA | 1.24 | 1 |
| lmo1852 | LMRG_00999 | Copper(I) chaperone CopZ | 1.24 | 1 |
| lmo2343 | LMRG_01500 | Putative monooxygenase MoxC | 1.24 | 1 |
| lmo0037 | LMRG_02466 | Agmatine/putrescine antiporter, associated with agmatine catabolism | 1.25 | 1 |
| lmo0278 | LMRG_02597 | Maltodextrin ABC transporter, ATP-binding protein MsmX | 1.25 | 1 |
| lmo1186 | LMRG_00632 | Ethanolamine permease | 1.25 | 1 |
| lmo1970 | LMRG_01117 | hypothetical protein | 1.25 | 1 |
| lmo2709 | LMRG_01988 | hypothetical protein | 1.25 | 1 |
| lmo0098 | LMRG_02347 | PTS system, mannose-specific IID component | 1.26 | 1 |
| lmo0372 | LMRG_00064 | 6-phospho-beta-glucosidase (EC 3.2.1.86) | 1.26 | 1 |
| lmo0515 | LMRG_00196 | Universal stress protein family | 1.26 | 1 |
| lmo1379 | LMRG_00831 | Inner membrane protein translocase and chaperone YidC, OxaA protein | 1.26 | 1 |
| lmo1651 | LMRG_01316 | Efflux ABC transporter, permease/ATP-binding protein MdlB | 1.26 | 1 |
| lmo2156 | LMRG_01676 | hypothetical protein | 1.26 | 1 |
| lmo2466 | LMRG_01782 | hypothetical protein | 1.26 | 1 |
| lmo2786 | LMRG_01911 | ADP-ribosylglycohydrolase | 1.26 | 1 |
| lmo0019 | LMRG_02448 | hypothetical protein | 1.27 | 1 |
| lmo0274 | LMRG_02602 | hypothetical protein | 1.27 | 1 |
| lmo0407 | LMRG_00100 | hypothetical protein | 1.27 | 1 |
| lmo1029 | LMRG_02129 | Hydrolase (HAD superfamily) in cluster with DUF1447 | 1.27 | 1 |
| lmo1049 | LMRG_00510 | Molybdopterin-synthase adenylyltransferase (EC 2.7.7.80) | 1.27 | 1 |
| lmo1518 | LMRG_01452 | hypothetical protein | 1.27 | 1 |
| lmo1866 | LMRG_01013 | Phosphoenolpyruvate synthase regulatory protein | 1.27 | 1 |
| lmo2797 | LMRG_01900 | PTS system, mannitol-specific IIA component (EC 2.7.1.197) | 1.27 | 1 |
| lmo0858 | LMRG_02281 | Transcriptional regulator, LacI family | 1.28 | 1 |
| lmo0917 | LMRG_02017 | 6-phospho-beta-glucosidase (EC 3.2.1.86) | 1.28 | 1 |
| lmo1788 | LMRG_02812 | Transcriptional regulator, MerR family | 1.28 | 1 |
| lmo2643 | LMRG_02188 | hypothetical protein | 1.28 | 1 |
| lmo0190 | LMRG_02755 | 4-diphosphocytidyl-2-C-methyl-D-erythritol kinase (EC 2.7.1.148) | 1.29 | 1 |
| lmo1278 | LMRG_00728 | ATP-dependent protease subunit HslV (EC 3.4.25.2) | 1.29 | 1 |
| lmo2215 | LMRG_01617 | ABC transporter, ATP-binding protein EcsA | 1.3 | 1 |
| lmo0517 | LMRG_00198 | Phosphoglycerate mutase family protein | 1.31 | 1 |
| lmo2639 | LMRG_02184 | hypothetical protein | 1.31 | 1 |
| lmo0222 | LMRG_02644 | 33 kDa chaperonin HslO | 1.32 | 1 |
| lmo1671 | LMRG_01296 | Zinc ABC transporter, substrate-binding protein ZnuA | 1.32 | 1 |
| lmo2603 | LMRG_02147 | acetamidase/formamidase family protein | 1.32 | 1 |
| lmo1062 | LMRG_00524 | ABC transporter, permease protein | 1.33 | 1 |
| lmo2642 | LMRG_02187 | Serine/threonine protein phosphatase family protein | 1.33 | 1 |
| lmo0700 | LMRG_00389 | Flagellar motor switch protein FliN | 1.34 | 1 |
| lmo0794 | LMRG_00482 | Rrf2-linked NADH-flavin reductase | 1.34 | 1 |
| lmo1751 | LMRG_02977 | 23S rRNA (uracil(1939)-C(5))-methyltransferase (EC 2.1.1.190) | 1.34 | 1 |
| lmo2028 | LMRG_01177 | Putative RNA-binding protein YlmH | 1.34 | 1 |
| lmo0557 | LMRG_00239 | Phosphoglycerate mutase family 1 | 1.35 | 1 |
| lmo2357 | LMRG_01485 | Membrane protein | 1.35 | 1 |
| lmo0537 | LMRG_00219 | N-carbamoyl-L-amino acid hydrolase (EC 3.5.1.87) | 1.36 | 1 |
| lmo1862 | LMRG_01009 | FIG006988: Lipase/Acylhydrolase with GDSL-like motif | 1.36 | 1 |
| lmo2207 | LMRG_01625 | Uncharacterized protein YetJ | 1.36 | 1 |
| lmo1056 | LMRG_00518 | hypothetical protein | 1.37 | 1 |
| lmo1069 | LMRG_00531 | Uncharacterized protein YlaI | 1.37 | 1 |
| lmo2087 | LMRG_01238 | Multi antimicrobial extrusion protein (Na(+)/drug antiporter), MATE family of MDR efflux pumps | 1.37 | 1 |
| lmo2116 | LMRG_01270 | hypothetical protein | 1.37 | 1 |
| lmo0835 | LMRG_02258 | Putative peptidoglycan bound protein (LPXTG motif) Lmo0835 homolog | 1.38 | 1 |
| lmo0899 | LMRG_02323 | Putative metallopeptidase (Zinc) SprT family | 1.38 | 1 |
| lmo2181 | LMRG_01651 | NPQTN specific sortase B | 1.38 | 1 |
| lmo2502 | LMRG_01746 | Cell division topological determinant MinJ | 1.39 | 1 |
| lmo1015 | LMRG_02115 | Glycine betaine ABC transport system, permease protein OpuAB | 1.4 | 1 |
| lmo1064 | LMRG_00526 | Magnesium and cobalt transport protein CorA | 1.4 | 1 |
| lmo1279 | LMRG_00729 | ATP-dependent hsl protease ATP-binding subunit HslU | 1.4 | 1 |
| lmo0901 | LMRG_02844 | PTS system, oligo-beta-mannoside-specific IIC component | 1.41 | 1 |
| lmo1515 | LMRG_01455 | Iron-sulfur cluster regulator IscR | 1.41 | 1 |
| lmo0026 | LMRG_02455 | Cytoplasmic copper homeostasis protein CutC | 1.43 | 1 |
| lmo0609 | LMRG_00292 | Rhodanese-like domain protein | 1.43 | 1 |
| lmo2233 | LMRG_01599 | Transcriptional regulator, LysR family | 1.43 | 1 |
| lmo0148 | LMRG_02391 | hypothetical protein | 1.44 | 1 |
| lmo0458 | LMRG_00150 | Hydantoinase/oxoprolinase family protein | 1.44 | 1 |
| lmo0518 | LMRG_00199 | Protein of unknown function DUF1304 | 1.44 | 1 |
| lmo2726 | LMRG_01970 | Transcriptional regulator, MarR family | 1.44 | 1 |
| lmo0347 | LMRG_00038 | Phosphoenolpyruvate-dihydroxyacetone phosphotransferase (EC 2.7.1.121), ADP-binding subunit DhaL | 1.45 | 1 |
| lmo0892 | LMRG_02316 | Serine phosphatase RsbU, regulator of sigma subunit | 1.45 | 1 |
| lmo1851 | LMRG_00998 | Carboxyl-terminal protease (EC 3.4.21.102) | 1.45 | 1 |
| lmo0902 | LMRG_02845 | Transcriptional regulator, GntR family | 1.46 | 1 |
| lmo2517 | LMRG_01731 | FIG009148: Acetyltransferase, GNAT family | 1.46 | 1 |
| lmo0403 | LMRG_00096 | hypothetical protein | 1.47 | 1 |
| lmo1057 | LMRG_00519 | L-lactate dehydrogenase (EC 1.1.1.27) | 1.47 | 1 |
| lmo2586 | LMRG_02681 | Putative formate dehydrogenase YrhE | 1.47 | 1 |
| lmo2588 | LMRG_02679 | Uncharacterized MFS-type transporter | 1.47 | 1 |
| lmo0946 | LMRG_02045 | hypothetical protein | 1.48 | 1 |
| lmo1802 | LMRG_00949 | Signal recognition particle associated protein | 1.48 | 1 |
| lmo0025 | LMRG_02454 | hypothetical protein | 1.49 | 1 |
| lmo0404 | LMRG_00097 | hypothetical protein | 1.49 | 1 |
| lmo1142 | LMRG_00585 | Cob(III)alamin reductase @ Cob(II)alamin reductase | 1.49 | 1 |
| lmo2428 | LMRG_01820 | Rod shape-determining protein RodA | 1.49 | 1 |
| lmo0281 | LMRG_02590 | cyclic nucleotide-binding protein | 1.5 | 1 |
| lmo2465 | LMRG_01783 | hypothetical protein | 1.5 | 1 |
| lmo0516 | LMRG_00197 | Capsule biosynthesis protein capA | 1.51 | 1 |
| lmo0786 | LMRG_00474 | FMN-dependent NADH-azoreductase (EC 1.7.1.6) | 1.51 | 1 |
| lmo2275 | LMRG_01559 | Phage protein | 1.51 | 1 |
| lmo2720 | LMRG_01976 | Acyl-coenzyme A synthetases/AMP-(fatty) acid ligases, YtcI homolog | 1.51 | 1 |
| lmo0642 | LMRG_02878 | Membrane protein | 1.52 | 1 |
| lmo1264 | LMRG_00713 | hypothetical protein | 1.52 | 1 |
| lmo1309 | LMRG_00759 | Co-activator of prophage gene expression IbrB | 1.52 | 1 |
| lmo1353 | LMRG_00803 | Uncharacterized protein YqhQ | 1.52 | 1 |
| lmo2092 | LMRG_01243 | Glycine betaine transporter OpuD | 1.52 | 1 |
| lmo0057 | LMRG_02487 | Putative secretion accessory protein EsaA/YueB @ Bacteriophage SPP1 receptor | 1.53 | 1 |
| lmo1128 | LMRG_00570 | hypothetical protein | 1.53 | 1 |
| lmor07 | LMRG_05507 | 5S rRNA ## 5S ribosomal RNA | 1.53 | 1 |
| lmo0034 | LMRG_02463 | PTS system, cellobiose-specific IIC component | 1.54 | 1 |
| lmo0602 | LMRG_00285 | Acetyltransferase, GNAT family | 1.55 | 1 |
| lmo2408 | LMRG_01840 | Transcriptional regulator, Xre family | 1.55 | 1 |
| lmo2544 | LMRG_01703 | Thymidine kinase (EC 2.7.1.21) | 1.55 | 1 |
| lmo0937 | LMRG_02036 | Uncharacterized protein Metme_2479 | 1.56 | 1 |
| lmo1782 | LMRG_02830 | Exodeoxyribonuclease III (EC 3.1.11.2) | 1.56 | 1 |
| lmo2260 | LMRG_01571 | Acetyltransferase, GNAT family, potentially associated with YqeK | 1.56 | 1 |
| lmo0649 | LMRG_00336 | Transcriptional regulator, GntR family | 1.57 | 1 |
| lmo1215 | LMRG_00661 | N-acetylmuramoyl-L-alanine amidase, family 4 | 1.57 | 1 |
| lmo1240 | LMRG_00686 | FIG009886: phosphoesterase | 1.57 | 1 |
| #N/A | LMRG_00323 | Lmo2594 protein | 1.58 | 1 |
| lmo0942 | LMRG_02040 | HtpG-like protein | 1.59 | 1 |
| lmo0963 | LMRG_02062 | Heat shock protein HtpX | 1.59 | 1 |
| lmo1014 | LMRG_02114 | Glycine betaine ABC transport system, ATP-binding protein OpuAA (EC 3.6.3.32) | 1.59 | 1 |
| lmo0452 | LMRG_00144 | FIG001454: Transglutaminase-like enzymes, putative cysteine proteases | 1.6 | 1 |
| lmo0891 | LMRG_02315 | Anti-sigma B factor RsbT | 1.6 | 1 |
| lmo1446 | LMRG_00898 | Zinc ABC transporter, permease protein ZnuB | 1.6 | 1 |
| lmo1502 | LMRG_01468 | Putative pre-16S rRNA nuclease YqgF | 1.6 | 1 |
| lmo1721 | LMRG_02550 | NtrC family Transcriptional regulator, ATPase domain | 1.61 | 1 |
| lmo0216 | LMRG_02638 | Ribosome-associated heat shock protein implicated in the recycling of the 50S subunit (S4 paralog) | 1.62 | 1 |
| lmo0787 | LMRG_00475 | Amino-acid permease AapA | 1.62 | 1 |
| lmo2481 | LMRG_01767 | Inorganic pyrophospatase PpaX (EC 3.1.3.18) | 1.62 | 1 |
| lmo0632 | LMRG_00315 | PTS system, fructose-specific IIC component | 1.63 | 1 |
| lmo2183 | LMRG_01649 | Heme transporter IsdDEF, permease component IsdF | 1.63 | 1 |
| lmo2705 | LMRG_01992 | hypothetical protein | 1.63 | 1 |
| lmo1723 | LMRG_02548 | hypothetical protein | 1.64 | 1 |
| lmo2188 | LMRG_01644 | Oligoendopeptidase F | 1.64 | 1 |
| lmo2616 | LMRG_02160 | LSU ribosomal protein L18p (L5e) | 1.64 | 1 |
| lmo0042 | LMRG_02471 | DedA protein | 1.65 | 1 |
| lmo0317 | LMRG_00010 | Hydroxymethylpyrimidine kinase (EC 2.7.1.49) @ Hydroxymethylpyrimidine phosphate kinase ThiD (EC 2.7.4.7) | 1.65 | 1 |
| lmo2369 | LMRG_02714 | General stress protein 13 | 1.65 | 1 |
| lmo2330 | LMRG_01513 | hypothetical protein | 1.66 | 1 |
| lmo2744 | LMRG_01952 | cyclic nucleotide-binding protein | 1.66 | 1 |
| lmo2834 | LMRG_01864 | Putative oxidoreductase YcjS (EC 1.-.-.-), NADH-binding | 1.66 | 1 |
| lmo0539 | LMRG_00221 | Tagatose 1,6-bisphosphate aldolase (EC 4.1.2.40) | 1.67 | 1 |
| lmo1198 | LMRG_00644 | Cobalt-precorrin 5A hydrolase (EC 3.7.1.12) | 1.67 | 1 |
| lmo2169 | LMRG_01663 | hypothetical protein | 1.67 | 1 |
| lmo0624 | LMRG_00307 | Acetyltransferase, GNAT family | 1.68 | 1 |
| lmo1968 | LMRG_01115 | Creatinine amidohydrolase (EC 3.5.2.10) | 1.68 | 1 |
| lmo2444 | LMRG_01804 | alpha-glucosidase | 1.68 | 1 |
| lmo0161 | LMRG_02406 | STAS domain protein | 1.69 | 1 |
| lmo2635 | LMRG_02180 | Putative prenyltransferase, contains 1,4-dihydroxy-2-naphthoate octaprenyltransferase domain | 1.69 | 1 |
| lmo0868 | LMRG_02292 | hypothetical protein | 1.71 | 1 |
| lmo1295 | LMRG_00745 | RNA-binding protein Hfq | 1.71 | 1 |
| lmo2187 | LMRG_01645 | hypothetical protein | 1.71 | 1 |
| lmo2088 | LMRG_01239 | Transcriptional regulator, AcrR family | 1.72 | 1 |
| lmo2136 | LMRG_02804 | PTS system, IIB component | 1.72 | 1 |
| lmo0536 | LMRG_00218 | glycosyl hydrolase, family 4 | 1.73 | 1 |
| lmo0725 | LMRG_00414 | putative peptidoglycan bound protein (LPXTG motif) | 1.73 | 1 |
| lmo1729 | LMRG_02542 | beta-glucosidase (EC 3.2.1.21) | 1.73 | 1 |
| lmo1748 | LMRG_02523 | hypothetical protein | 1.73 | 1 |
| lmo2421 | LMRG_01827 | Two-component system sensor histidine kinase | 1.73 | 1 |
| lmo2489 | LMRG_01759 | Excinuclease ABC subunit B | 1.73 | 1 |
| lmor03 | LMRG_05503 | 5S rRNA ## 5S ribosomal RNA | 1.73 | 1 |
| lmo1209 | LMRG_00655 | ATP:Cob(I)alamin adenosyltransferase (EC 2.5.1.17) @ ATP:Cob(I)alamin adenosyltransferase (EC 2.5.1.17), cobalamin synthesis | 1.74 | 1 |
| lmo0089 | LMRG_02969 | ATP synthase delta chain (EC 3.6.3.14) | 1.75 | 1 |
| lmo0559 | LMRG_00241 | Magnesium and cobalt transport protein CorA | 1.75 | 1 |
| lmo2409 | LMRG_01839 | hypothetical protein | 1.75 | 1 |
| lmo1536 | LMRG_01434 | Prephenate dehydratase (EC 4.2.1.51) | 1.76 | 1 |
| lmo2302 | LMRG_01530 | Protein gp68 | 1.76 | 1 |
| lmo2536a | #N/A | hypothetical protein | 1.76 | 1 |
| lmo2836 | LMRG_01862 | Zinc-type alcohol dehydrogenase YcjQ | 1.76 | 1 |
| lmo0513 | LMRG_00194 | Transcriptional regulator, MarR family | 1.77 | 1 |
| lmo0881 | LMRG_02305 | Lmo0881 homolog, only in Listeria | 1.78 | 1 |
| lmo0928 | LMRG_02027 | DNA-3-methyladenine glycosylase II (EC 3.2.2.21) | 1.78 | 1 |
| lmo0959 | LMRG_02058 | Undecaprenyl-phosphate alpha-N-acetylglucosaminyl 1-phosphate transferase (EC 2.7.8.33) | 1.78 | 1 |
| lmo2688 | LMRG_02234 | FtsW-like protein YlaO | 1.78 | 1 |
| lmo0854 | LMRG_02277 | small multidrug resistance family (SMR) protein | 1.79 | 1 |
| lmo1681 | LMRG_01286 | 5-methyltetrahydropteroyltriglutamate--homocysteine methyltransferase (EC 2.1.1.14) | 1.79 | 1 |
| lmo2159 | LMRG_01673 | Myo-inositol 2-dehydrogenase (EC 1.1.1.18) | 1.79 | 1 |
| lmo2708 | LMRG_01989 | PTS system, cellobiose-specific IIC component | 1.79 | 1 |
| lmo0163 | LMRG_02408 | Stage 0 sporulation protein YaaT | 1.8 | 1 |
| lmo0555 | LMRG_00237 | Di-tripeptide/cation symporter DtpT | 1.8 | 1 |
| lmo1799 | LMRG_02823 | Putative peptidoglycan bound protein (LPXTG motif) Lmo1799 homolog | 1.81 | 1 |
| lmo2443 | LMRG_01805 | hypothetical protein | 1.81 | 1 |
| lmo0867 | LMRG_02291 | Substrate-specific component QueT (COG4708) of predicted queuosine-regulated ECF transporter | 1.82 | 1 |
| lmo1731 | LMRG_02540 | N-acetyl-D-glucosamine ABC transporter, permease protein 1 | 1.82 | 1 |
| lmo0230 | LMRG_02676 | Protein-arginine kinase activator protein McsA | 1.83 | 1 |
| lmo2727 | LMRG_01969 | leucine-rich protein | 1.84 | 1 |
| lmo1016 | LMRG_02116 | Glycine betaine ABC transport system, glycine betaine-binding protein OpuAC | 1.86 | 1 |
| lmo2208 | LMRG_01624 | Hydrolase, haloacid dehalogenase-like family | 1.86 | 1 |
| lmo0801 | LMRG_02867 | Internalin-like protein (LPXTG motif) Lmo0801 homolog | 1.87 | 1 |
| lmo1339 | LMRG_00789 | Glucokinase (EC 2.7.1.2) | 1.87 | 1 |
| lmo1370 | LMRG_00820 | Branched-chain acyl kinase | 1.87 | 1 |
| lmo0405 | LMRG_00098 | Probable low-affinity inorganic phosphate transporter | 1.88 | 1 |
| lmo2820 | LMRG_01878 | Transcriptional regulator, AraC family / Glycoside hydrolase | 1.89 | 1 |
| lmo0971 | LMRG_02070 | Poly(glycerophosphate chain) D-alanine transfer protein DltD | 1.9 | 1 |
| lmo0507 | LMRG_00188 | PTS system, galactitol-specific IIB component (EC 2.7.1.200) | 1.91 | 1 |
| lmo0920 | LMRG_02020 | UPF0718 protein YcgR | 1.91 | 1 |
| lmo0285 | LMRG_02586 | Methionine ABC transporter substrate-binding protein | 1.93 | 1 |
| lmo1424 | LMRG_00876 | Manganese transport protein MntH | 1.94 | 1 |
| lmo2778 | LMRG_01919 | hypothetical protein | 1.94 | 1 |
| lmo0815 | LMRG_02782 | Uncharacterized transcriptional regulator YybA, MarR family | 1.95 | 1 |
| lmo2783 | LMRG_01914 | PTS system, beta-glucoside-specific, IIC component | 1.95 | 1 |
| #N/A | LMRG_02575 | Type I restriction-modification system, restriction subunit R (EC 3.1.21.3) | 1.95 | 1 |
| lmo0475 | LMRG_00156 | hypothetical protein | 1.97 | 1 |
| lmo1893 | LMRG_01040 | Hypothetical protein in cluster with penicillin-binding protein PBP1, Listerial type | 1.97 | 1 |
| lmo0459 | LMRG_00151 | hypothetical protein | 1.98 | 1 |
| lmo0805 | LMRG_02792 | hypothetical protein | 1.98 | 1 |
| lmo2722 | LMRG_01974 | multidrug efflux transporter transcriptional regulatory protein | 1.98 | 1 |
| lmo2742 | LMRG_01954 | hypothetical protein | 1.98 | 1 |
| lmo0101 | LMRG_02350 | Transcriptional regulator, ArsR family | 2 | 1 |
| lmo0749 | LMRG_00437 | Transcriptional regulator, Xre family | 2 | 1 |
| lmo0804 | LMRG_02793 | hypothetical protein | 2 | 1 |
| lmo2780 | LMRG_01917 | PTS system, beta-glucoside-specific, IIA component | 2 | 1 |
| lmo1135 | LMRG_00577 | hypothetical protein | 2.01 | 1 |
| lmo2712 | LMRG_01984 | Gluconokinase (EC 2.7.1.12) | 2.01 | 1 |
| lmo0487 | LMRG_00168 | ADP-ribose pyrophosphatase (EC 3.6.1.13) | 2.02 | 1 |
| lmo2079 | LMRG_01230 | hypothetical protein | 2.02 | 1 |
| lmo2659 | LMRG_02204 | Ribulose-phosphate 3-epimerase (EC 5.1.3.1) | 2.02 | 1 |
| lmo1468 | LMRG_00921 | Transamidase GatB domain protein | 2.03 | 1 |
| lmo2223 | LMRG_01609 | UPF0342 protein YheA | 2.03 | 1 |
| lmo2813 | LMRG_01885 | hypothetical protein | 2.03 | 1 |
| lmo0309 | LMRG_00006 | RloF | 2.04 | 1 |
| lmo1962 | LMRG_01109 | Transcriptional regulator, AcrR family | 2.04 | 1 |
| lmo2278 | LMRG_01554 | Endolysin, L-alanyl-D-glutamate peptidase (EC 3.4.-.-) [Bacteriophage A118] | 2.04 | 1 |
| lmo0275 | LMRG_02600 | ComEC like protein FIG012334 | 2.06 | 1 |
| lmo1624 | LMRG_01342 | Probable cell division protein YtgP | 2.06 | 1 |
| lmo1965 | LMRG_01112 | ADP-ribose pyrophosphatase (EC 3.6.1.13) | 2.07 | 1 |
| lmo1499 | LMRG_01471 | Murein endolytic transglycosylase MltG | 2.08 | 1 |
| lmo2287 | LMRG_01545 | Tail tape-measure protein [Bacteriophage A118] | 2.08 | 1 |
| lmo1173 | LMRG_00619 | Ethanolamine sensory transduction histidine kinase | 2.09 | 1 |
| lmo1476 | LMRG_00929 | Oxygen-independent coproporphyrinogen-III oxidase-like protein YggW | 2.09 | 1 |
| lmo0490 | LMRG_00171 | Shikimate/quinate 5-dehydrogenase I beta (EC 1.1.1.282) | 2.1 | 1 |
| lmo0944 | LMRG_02042 | hypothetical protein | 2.1 | 1 |
| lmo1055 | LMRG_00517 | Dihydrolipoamide dehydrogenase of pyruvate dehydrogenase complex (EC 1.8.1.4) | 2.12 | 1 |
| lmo1421 | LMRG_00873 | Glycine betaine ABC transport system, ATP-binding protein OpuAA (EC 3.6.3.32) | 2.12 | 1 |
| lmo1585 | LMRG_01382 | Protease IV | 2.12 | 1 |
| lmo0771 | LMRG_00459 | hypothetical protein | 2.13 | 1 |
| lmo1704 | LMRG_02778 | OsmC/Ohr family protein | 2.13 | 1 |
| lmo1994 | LMRG_01142 | Transcriptional regulator, LacI family | 2.14 | 1 |
| lmo1471 | LMRG_00924 | Ribosomal protein L11 methyltransferase | 2.15 | 1 |
| lmo0455 | LMRG_00147 | Regulator of polyketide synthase expression | 2.16 | 1 |
| #N/A | LMRG_02577 | Type I restriction-modification system, specificity subunit S (EC 3.1.21.3) | 2.16 | 1 |
| lmo2358 | LMRG_01484 | Glucosamine-6-phosphate deaminase (EC 3.5.99.6) | 2.18 | 1 |
| lmo1933 | LMRG_01080 | GTP cyclohydrolase I (EC 3.5.4.16) type 1 | 2.19 | 1 |
| lmo2733 | LMRG_01963 | PTS system, IIA component / PTS system, IIB component / PTS system, IIC component | 2.19 | 1 |
| lmo2199 | LMRG_01633 | Organic hydroperoxide resistance protein | 2.2 | 1 |
| lmo0111 | LMRG_02360 | hypothetical protein | 2.21 | 1 |
| lmo1999 | LMRG_01147 | putative glucosamine-fructose-6-phosphate aminotransferase | 2.21 | 1 |
| lmo2331 | LMRG_01512 | hypothetical protein | 2.22 | 1 |
| lmo2707 | LMRG_01990 | hypothetical protein | 2.23 | 1 |
| lmo1997 | LMRG_01145 | PTS system, mannose-specific IIA component (EC 2.7.1.191) | 2.26 | 1 |
| lmo2256 | LMRG_01575 | Intracellular protease | 2.27 | 1 |
| lmo0421 | LMRG_00113 | Rod shape-determining protein RodA | 2.29 | 1 |
| lmo0549 | LMRG_00231 | Internalin-like protein Lmo0549 homolog | 2.34 | 1 |
| lmo1404 | LMRG_00856 | DNA mismatch repair protein MutL | 2.34 | 1 |
| lmo2326 | LMRG_01518 | Protein gp41 [Bacteriophage A118] | 2.34 | 1 |
| lmo0438 | LMRG_00130 | hypothetical protein | 2.36 | 1 |
| lmo2580 | LMRG_02687 | Heme efflux system ATPase HrtA | 2.36 | 1 |
| lmo1477 | LMRG_00930 | NAD-dependent, hopanoid-associated epimerase/dehydratase family acting on nucleotide-sugars | 2.37 | 1 |
| lmo0923 | LMRG_02023 | Efflux ABC transporter, ATP-binding protein | 2.38 | 1 |
| #N/A | LMRG_02846 | hypothetical protein | 2.39 | 1 |
| lmo2653 | LMRG_02198 | Translation elongation factor Tu | 2.4 | 1 |
| lmo0039 | LMRG_02468 | Carbamate kinase (EC 2.7.2.2) | 2.41 | 1 |
| lmo2469 | LMRG_01779 | Uncharacterized amino acid permease, GabP family | 2.41 | 1 |
| lmo0411 | LMRG_00103 | Pyruvate-utilizing enzyme, similar to phosphoenolpyruvate synthase | 2.42 | 1 |
| lmo0453 | LMRG_00145 | FIG002343: hypothetical protein | 2.45 | 1 |
| lmo0952 | LMRG_02051 | hypothetical protein | 2.45 | 1 |
| lmo0775 | LMRG_00463 | hypothetical protein | 2.46 | 1 |
| lmo1730 | LMRG_02541 | N-acetyl-D-glucosamine ABC transporter, substrate-binding protein | 2.48 | 1 |
| lmo1858 | LMRG_01005 | Oxidoreductase, Gfo/Idh/MocA family | 2.48 | 1 |
| lmo0381 | LMRG_00074 | hypothetical protein | 2.49 | 1 |
| lmo0977 | LMRG_02077 | Putative acetyl esterase YjcH (EC 3.1.1.-) | 2.49 | 1 |
| lmo2165 | LMRG_01667 | hypothetical protein | 2.49 | 1 |
| lmo0898 | LMRG_02322 | Transcription accessory protein (S1 RNA-binding domain) | 2.5 | 1 |
| lmo2576 | LMRG_02692 | Putative peptidoglycan bound protein (LPXTG motif) Lmo2576 homolog | 2.5 | 1 |
| lmo2363 | LMRG_01479 | Glutamate decarboxylase (EC 4.1.1.15) | 2.52 | 1 |
| lmo2760 | LMRG_01936 | Bis-ABC ATPase BA0228 | 2.53 | 1 |
| lmo2218 | LMRG_01614 | Hypothetical protein SAV1840 | 2.55 | 1 |
| lmo0040 | LMRG_02469 | Agmatine deiminase (EC 3.5.3.12) | 2.59 | 1 |
| lmo1644 | LMRG_01322 | helicase, Snf2 family | 2.6 | 1 |
| lmo1601 | LMRG_01366 | General stress protein | 2.61 | 1 |
| lmo0846 | LMRG_02269 | Excinuclease ABC subunit C | 2.62 | 1 |
| lmo0368 | LMRG_00060 | Nudix hydrolase-like protein | 2.63 | 1 |
| lmo1963 | LMRG_01110 | ABC transporter, permease protein | 2.64 | 1 |
| lmo2763 | LMRG_01932 | PTS system, cellobiose-specific IIC component | 2.64 | 1 |
| lmo0913 | LMRG_02013 | Succinate-semialdehyde dehydrogenase [NAD(P)+] (EC 1.2.1.16) | 2.65 | 1 |
| lmo2467 | LMRG_01781 | Chitin binding protein | 2.66 | 1 |
| lmo1411 | LMRG_00863 | Uncharacterized transcriptional regulator YozG, Cro/CI family | 2.7 | 1 |
| lmo2689 | LMRG_02235 | Mg(2+) transport ATPase, P-type (EC 3.6.3.2) | 2.7 | 1 |
| lmo0377 | LMRG_00069 | hypothetical protein | 2.71 | 1 |
| lmo2751 | LMRG_01945 | Heterodimeric efflux ABC transporter, multidrug resistance => LmrC subunit of LmrCD | 2.74 | 1 |
| lmo2843 | LMRG_01855 | hypothetical protein | 2.77 | 1 |
| lmo0849 | LMRG_02272 | Amidase family protein | 2.81 | 1 |
| lmo0174 | LMRG_02982 | Mobile element protein | 2.83 | 1 |
| lmo2461 | LMRG_01787 | RNA polymerase sigma-54 factor RpoN | 2.83 | 1 |
| lmo0103 | LMRG_02352 | Nitroreductase family protein | 2.89 | 1 |
| lmo2410 | LMRG_01838 | hypothetical protein | 2.89 | 1 |
| lmo0654 | LMRG_00341 | hypothetical protein | 2.91 | 1 |
| lmo0816 | LMRG_02781 | N1-spermidine/spermine acetyltransferase PaiA | 2.94 | 1 |
| lmo2492 | LMRG_01756 | hypothetical protein | 3.01 | 1 |
| lmo0790 | LMRG_00478 | Cys-tRNA(Pro) deacylase YbaK | 3.02 | 1 |
| lmo2175 | LMRG_01657 | Oxidoreductase, short-chain dehydrogenase/reductase family | 3.03 | 1 |
| lmo2220 | LMRG_01612 | 3'->5' exoribonuclease Bsu YhaM | 3.05 | 1 |
| lmo2700 | LMRG_01997 | oxidoreductase of aldo/keto reductase family, subgroup 2 | 3.06 | 1 |
| lmo1957 | LMRG_01104 | Ferrichrome transport system permease protein FhuG | 3.07 | 1 |
| lmo1017 | LMRG_02117 | PTS system, glucose-specific IIA component (EC 2.7.1.199) | 3.17 | 1 |
| lmo0307 | LMRG_00004 | hypothetical protein | 3.18 | 1 |
| lmo2593 | LMRG_02133 | Transcriptional regulator, MerR family | 3.18 | 1 |
| lmo0456 | LMRG_00148 | Cytosine/purine/uracil/thiamine/allantoin permease family protein | 3.2 | 1 |
| lmo0896 | LMRG_02320 | Phosphoserine phosphatase RsbX (EC 3.1.3.3) | 3.21 | 1 |
| lmo1826 | LMRG_00973 | DNA-directed RNA polymerase omega subunit (EC 2.7.7.6) | 3.21 | 1 |
| lmo0378 | LMRG_00070 | hypothetical protein | 3.23 | 1 |
| lmo1975 | LMRG_01122 | DNA polymerase IV (EC 2.7.7.7) | 3.23 | 1 |
| lmo1467 | LMRG_00919 | Phosphate starvation-inducible protein PhoH, predicted ATPase | 3.24 | 1 |
| #N/A | LMRG_02842 | hypothetical protein | 3.24 | 1 |
| lmo0364 | LMRG_00056 | Transcriptional regulator, YafY family | 3.25 | 1 |
| lmo0394 | LMRG_00087 | Peptidase Lmo0394 homolog | 3.3 | 1 |
| lmo1806 | LMRG_00953 | Acyl carrier protein | 3.3 | 1 |
| lmo0526 | LMRG_00207 | Transcriptional regulator, MerR family | 3.31 | 1 |
| lmo0572 | LMRG_00254 | hypothetical protein | 3.31 | 1 |
| lmo1255 | LMRG_00704 | PTS system, trehalose-specific IIB component (EC 2.7.1.201) / PTS system, trehalose-specific IIC component | 3.31 | 1 |
| lmo2773 | LMRG_02900 | Beta-glucoside bgl operon antiterminator, BglG family | 3.34 | 1 |
| lmo0062 | LMRG_02493 | FIG00776004: hypothetical protein | 3.39 | 1 |
| lmo1000 | LMRG_02100 | Dehydrosqualene desaturase (EC 1.3.8.2) @ 4,4'-diapolycopene oxidase | 3.39 | 1 |
| lmo0809 | LMRG_02788 | Spermidine/putrescine import ABC transporter permease protein PotC (TC 3.A.1.11.1) | 3.4 | 1 |
| lmo1589 | LMRG_01378 | N-acetylglutamate kinase (EC 2.7.2.8) | 3.4 | 1 |
| lmo1881 | LMRG_01028 | DNA polymerase I 5'-3' exonuclease domain | 3.43 | 1 |
| lmo1217 | LMRG_00663 | Putative aminopeptidase YsdC | 3.46 | 1 |
| lmo0789 | LMRG_00477 | Phenazine biosynthesis protein PhzF like | 3.49 | 1 |
| lmo1554 | LMRG_01414 | Porphobilinogen synthase (EC 4.2.1.24) | 3.5 | 1 |
| lmo1456 | LMRG_00908 | hypothetical protein | 3.54 | 1 |
| lmo0155 | LMRG_02400 | Zinc ABC transporter, permease protein AdcB | 3.55 | 1 |
| lmo2368 | LMRG_02713 | MutT/Nudix family protein | 3.55 | 1 |
| lmo1586 | LMRG_01381 | NAD kinase (EC 2.7.1.23) homolog | 3.59 | 1 |
| lmo0975 | LMRG_02075 | Ribose-5-phosphate isomerase A (EC 5.3.1.6) | 3.61 | 1 |
| lmo1068 | LMRG_00530 | hypothetical protein | 3.62 | 1 |
| lmo1242 | LMRG_00688 | DUF1801 domain-containing protein | 3.65 | 1 |
| lmo2174 | LMRG_01658 | GGDEF domain protein | 3.65 | 1 |
| lmo0322 | LMRG_00014 | hypothetical protein | 3.66 | 1 |
| lmo0451 | LMRG_00143 | hypothetical protein | 3.7 | 1 |
| lmo0842 | LMRG_02265 | hypothetical protein | 3.7 | 1 |
| lmo2821 | LMRG_01877 | Internalin-like protein (LPXTG motif) Lmo2821 homolog | 3.7 | 1 |
| lmo1966 | LMRG_01113 | Protein XpaC | 3.71 | 1 |
| lmo1246 | LMRG_00692 | ATP-dependent RNA helicase YxiN | 3.75 | 1 |
| lmo2305 | LMRG_02921 | hypothetical protein | 3.75 | 1 |
| lmo1548 | LMRG_01422 | Rod shape-determining protein MreB | 3.79 | 1 |
| lmo0454 | LMRG_00146 | FIG022979: MoxR-like ATPases | 3.82 | 1 |
| lmo2023 | LMRG_01172 | L-aspartate oxidase (EC 1.4.3.16) | 3.82 | 1 |
| #N/A | LMRG_02937 | FIG00775722: hypothetical protein | 3.9 | 1 |
| lmo2290 | LMRG_01542 | Protein gp13 [Bacteriophage A118] | 3.95 | 1 |
| lmo1187 | LMRG_00633 | Ethanolamine utilization protein EutQ | 3.97 | 1 |
| lmo2137 | LMRG_02803 | PTS system, IIA component | 4.04 | 1 |
| lmo0592 | LMRG_00274 | hypothetical protein | 4.08 | 1 |
| lmo2143 | LMRG_02797 | mannose-6-phosphate isomerase, class I | 4.08 | 1 |
| lmo1024 | LMRG_02124 | hypothetical protein | 4.14 | 1 |
| lmo2273 | LMRG_01557 | hypothetical protein | 4.15 | 1 |
| lmo2292 | LMRG_01540 | Protein gp11 [Bacteriophage A118] | 4.15 | 1 |
| lmo0587 | LMRG_00269 | cell surface protein precursor | 4.17 | 1 |
| lmo0668 | LMRG_00355 | Uncharacterized efflux ABC transporter, permease protein YadH | 4.21 | 1 |
| lmo1500 | LMRG_01470 | DedA family protein | 4.21 | 1 |
| lmo2755 | LMRG_01941 | hydrolase, CocE/NonD family | 4.22 | 1 |
| lmo0193 | LMRG_02615 | possible membrane fusion protein | 4.25 | 1 |
| lmo1189 | LMRG_00635 | Transcriptional regulator, AraC family | 4.25 | 1 |
| lmo0393 | LMRG_00086 | hypothetical protein | 4.27 | 1 |
| lmo1461 | LMRG_00913 | hypothetical protein | 4.34 | 1 |
| lmo2349 | LMRG_01494 | L-cystine ABC transporter, substrate-binding protein TcyK | 4.34 | 1 |
| lmo0940 | LMRG_02950 | hypothetical protein | 4.54 | 1 |
| lmo0522 | LMRG_00203 | Transcriptional regulator, RpiR family | 4.55 | 1 |
| lmo1410 | LMRG_00862 | hypothetical protein | 4.65 | 1 |
| lmo1732 | LMRG_02539 | ABC transporter, permease protein 2 (cluster 1, maltose/g3p/polyamine/iron) | 4.65 | 1 |
| lmo0751 | LMRG_00439 | hypothetical protein | 4.68 | 1 |
| lmo0085 | LMRG_02334 | hypothetical protein | 4.71 | 1 |
| lmo0652 | LMRG_00339 | Acetyltransferase, GNAT family | 4.72 | 1 |
| lmo1254 | LMRG_00703 | Trehalose-6-phosphate hydrolase (EC 3.2.1.93) | 4.74 | 1 |
| lmo2130 | LMRG_01284 | Uncharacterized amino acid permease YdaO | 4.75 | 1 |
| lmo1812 | LMRG_00959 | L-serine dehydratase, alpha subunit (EC 4.3.1.17) | 4.78 | 1 |
| lmo1018 | LMRG_02118 | Cytoplasmic copper homeostasis protein CutC | 4.87 | 1 |
| lmo1696 | LMRG_02770 | Uncharacterized membrane spanning protein, contains VanZ-like domain | 4.96 | 1 |
| lmo1871 | LMRG_01018 | Phosphoglucomutase | 4.96 | 1 |
| lmo2237 | LMRG_01595 | Transport protein | 4.99 | 1 |
| lmo0919 | LMRG_02019 | Bis-ABC ATPase | 5.03 | 1 |
| lmo1659 | #N/A | FIG00774304: hypothetical protein | 5.15 | 1 |
| lmo0013 | LMRG_02442 | Cytochrome aa3-600 menaquinol oxidase subunit II | 5.19 | 1 |
| lmo0479 | LMRG_02884 | putative secreted protein | 5.31 | 1 |
| lmo1683 | LMRG_02757 | Peroxide stress regulator PerR, FUR family | 5.38 | 1 |
| lmo1464 | LMRG_00916 | Diacylglycerol kinase (EC 2.7.1.107) | 5.39 | 1 |
| lmo1845 | LMRG_00992 | xanthine/uracil permease family protein | 5.69 | 1 |
| lmo0129 | LMRG_02378 | N-acetylmuramoyl-L-alanine amidase (EC 3.5.1.28) | 5.72 | 1 |
| lmo2730 | LMRG_01966 | Haloacid dehalogenase-like hydrolase | 5.88 | 1 |
| lmo1164 | LMRG_00607 | ATP:Cob(I)alamin adenosyltransferase (EC 2.5.1.17) @ ATP:Cob(I)alamin adenosyltransferase (EC 2.5.1.17), propanediol utilization / Uncharacterized conserved protein GlcG | 6.2 | 1 |
| lmo2684 | LMRG_02229 | PTS system, cellobiose-specific IIC component | 6.26 | 1 |
| lmo0648 | LMRG_00335 | Magnesium and cobalt transport protein CorA | 6.3 | 1 |
| lmo2480 | LMRG_01768 | Putative acetyltransferase YvoF | 6.34 | 1 |
| lmo0353 | LMRG_00044 | Acetyltransferase, GNAT family | 6.49 | 1 |
| lmo0779 | LMRG_00467 | UPF0266 membrane protein YobD | 6.49 | 1 |
| lmo1303 | LMRG_00753 | Cell division suppressor protein YneA | 6.63 | 1 |
| lmo2025 | LMRG_01174 | Quinolinate synthetase (EC 2.5.1.72) | 6.8 | 1 |
| lmo0365 | LMRG_00057 | Ferrous iron transport permease EfeU | 6.86 | 1 |
| lmo0392 | LMRG_00085 | UPF0365 protein YqfA | 7.03 | 1 |
| lmo0866a | LMRG_02290 | FIG00774648: hypothetical protein | 7.64 | 1 |
| lmo1884 | LMRG_01031 | Xanthine permease | 8.92 | 1 |
| lmo0496 | LMRG_00177 | hypothetical protein | 9.69 | 1 |
| lmo0893 | LMRG_02317 | Anti-sigma B factor antagonist RsbV | 10.34 | 1 |
| lmo1590 | LMRG_01377 | Glutamate N-acetyltransferase (EC 2.3.1.35) @ N-acetylglutamate synthase (EC 2.3.1.1) | 10.44 | 1 |
| lmo1213 | LMRG_00659 | Transcriptional regulator, PadR family | 12.11 | 1 |

Supplemental Table 6. All mapped reads for spleen samples.

| **EGDe locus** | **10403S locus** | **Description** | **log2FC** | **Adj. p-value** |
| --- | --- | --- | --- | --- |
| lmo2425 | LMRG_01823 | Glycine cleavage system H protein | -8.43 | 0 |
| lmo2770 | LMRG_01925 | Glutathione biosynthesis bifunctional protein gshF (EC 6.3.2.2)(EC 6.3.2.3) | -8.36 | 0 |
| lmo0202 | LMRG_02624 | Thiol-activated cytolysin | -8.13 | 0 |
| lmo2219 | LMRG_01613 | Foldase protein PrsA precursor (EC 5.2.1.8) @ Foldase clustered with pyrimidine conversion | -7.94 | 0 |
| lmo0200 | LMRG_02622 | Virulence regulatory factor PrfA / Transcriptional regulator, Crp/Fnr family | -7.9 | 0 |
| lmo2474 | LMRG_01774 | RNase adapter protein RapZ | -7.66 | 0 |
| lmo2566 | LMRG_02703 | Lipoyl-[GcvH]:protein N-lipoyltransferase (EC 2.3.1.200) | -7.62 | 0 |
| lmo1052 | LMRG_00514 | Pyruvate dehydrogenase E1 component alpha subunit (EC 1.2.4.1) | -7.39 | 0 |
| lmo1773 | LMRG_02498 | Adenylosuccinate lyase (EC 4.3.2.2) @ SAICAR lyase (EC 4.3.2.2) | -6.88 | 0 |
| lmo0598 | LMRG_00281 | Substrate-specific component BioY of biotin ECF transporter | -6.88 | 0 |
| lmo2718 | LMRG_01978 | Cytochrome d ubiquinol oxidase subunit I (EC 1.10.3.-) | -6.65 | 0 |
| lmo1054 | LMRG_00516 | Dihydrolipoamide acetyltransferase component of pyruvate dehydrogenase complex (EC 2.3.1.12) | -6.41 | 0 |
| lmo2769 | LMRG_01926 | Efflux ABC transporter, ATP-binding protein | -6.34 | 0 |
| lmo0055 | LMRG_02485 | Adenylosuccinate synthetase (EC 6.3.4.4) | -6.03 | 0 |
| lmo0583 | LMRG_00265 | Protein translocase subunit SecA paralog 1 | -6.02 | 0 |
| lmo2103 | LMRG_01256 | Phosphate acetyltransferase (EC 2.3.1.8) | -5.66 | 0 |
| lmo0734 | LMRG_00422 | Transcriptional regulator, LacI family | -5.51 | 0 |
| lmo0641 | LMRG_00327 | Lead, cadmium, zinc and mercury transporting ATPase (EC 3.6.3.3) (EC 3.6.3.5) | -5.46 | 0 |
| lmo2716 | LMRG_01980 | Efflux ABC transporter for glutathione/L-cysteine, essential for assembly of bd-type respiratory oxidases => CydC subunit | -5.21 | 0 |
| lmo2715 | LMRG_01981 | Efflux ABC transporter for glutathione/L-cysteine, essential for assembly of bd-type respiratory oxidases => CydD subunit | -5.08 | 0 |
| lmo0931 | LMRG_02030 | Lipoate-protein ligase A | -4.94 | 0 |
| lmo2456 | LMRG_01792 | 2,3-bisphosphoglycerate-independent phosphoglycerate mutase (EC 5.4.2.12) | -4.72 | 0 |
| lmo2750 | LMRG_01946 | Para-aminobenzoate synthase, aminase component (EC 2.6.1.85) / Aminodeoxychorismate lyase (EC 4.1.3.38) | -4.39 | 0 |
| lmo0031 | LMRG_02460 | Transcriptional regulator, LacI family | -4.21 | 0 |
| lmo2072 | LMRG_01223 | Redox-sensing transcriptional repressor Rex | -4.17 | 0 |
| lmo1930 | LMRG_01077 | Heptaprenyl diphosphate synthase component II (EC 2.5.1.30) | -4 | 0 |
| lmo1053 | LMRG_00515 | Pyruvate dehydrogenase E1 component beta subunit (EC 1.2.4.1) | -3.71 | 0 |
| lmo1386 | LMRG_00838 | DNA translocase FtsK | -3.7 | 0 |
| lmo0205 | LMRG_02627 | Broad-substrate range phospholipase C (EC 3.1.4.3) | -3.52 | 0 |
| lmo2046 | LMRG_01195 | 2-dehydropantoate 2-reductase (EC 1.1.1.169) | -3.28 | 0 |
| lmo1599 | LMRG_01368 | Catabolite control protein A | -3.22 | 0 |
| lmo0214 | LMRG_02636 | Transcription-repair coupling factor | -2.1 | 0 |
| lmo0204 | LMRG_02626 | Actin-assembly inducing protein ActA precursor | -6.95 | 0.00841 |
| lmo1581 | LMRG_01386 | Acetate kinase (EC 2.7.2.1) | -3.84 | 0.00841 |
| lmo1892 | LMRG_01039 | Penicillin-binding protein 1A/1B @ Multimodular transpeptidase-transglycosylase | -3.03 | 0.00841 |
| lmo0964 | LMRG_02063 | FIG00002411: Thioredoxin-fold protein | -5.11 | 0.01591 |
| lmo2546 | LMRG_01701 | Threonine synthase (EC 4.2.3.1) | -2.89 | 0.01591 |
| lmo0978 | LMRG_02078 | Branched-chain amino acid aminotransferase (EC 2.6.1.42) | -2.69 | 0.02324 |
| lmo2772 | LMRG_01923 | PTS system, beta-glucoside-specific IIB component / PTS system, beta-glucoside-specific IIC component / PTS system, beta-glucoside-specific IIA component | -1.91 | 0.03019 |
| lmo1429 | LMRG_00881 | Substrate-specific component ThiT of thiamin ECF transporter | -4.71 | 0.0359 |
| lmo2054 | LMRG_01204 | UPF0298 protein YlbG | -3.46 | 0.0359 |
| lmo0741 | LMRG_00429 | Transcriptional regulator, GntR family | -4.19 | 0.04684 |
| lmo1571 | LMRG_01396 | 6-phosphofructokinase (EC 2.7.1.11) | -3.57 | 0.04684 |
| lmo0203 | LMRG_02625 | Zinc metalloproteinase aureolysin (EC 3.4.24.29) | -2.97 | 0.04684 |
| #N/A | LMRG_02892 | hypothetical protein | -1.51 | 0.05234 |
| lmo0154 | LMRG_02399 | Zinc ABC transporter, ATP-binding protein AdcC | -6.11 | 0.06264 |
| lmo1677 | LMRG_01290 | 1,4-dihydroxy-2-naphthoate polyprenyltransferase (EC 2.5.1.74) | -3.07 | 0.06264 |
| lmo0897 | LMRG_02321 | Sulfate permease | -4.88 | 0.06477 |
| lmo0358 | LMRG_00049 | PTS system, IIB component / PTS system, IIC component | -4.14 | 0.06477 |
| lmo1354 | LMRG_00804 | Aminopeptidase YpdF (MP-, MA-, MS-, AP-, NP- specific) | -2.59 | 0.06477 |
| lmo1771 | LMRG_02500 | Phosphoribosylformylglycinamidine synthase, PurS subunit (EC 6.3.5.3) | -5.23 | 0.06794 |
| lmo2768 | LMRG_01927 | Membrane protein | -5.16 | 0.06794 |
| lmo0712 | LMRG_00401 | Flagellar hook-basal body complex protein FliE | -6.14 | 0.07221 |
| lmo0728 | LMRG_00416 | FMN adenylyltransferase (EC 2.7.7.2) / Riboflavin kinase (EC 2.7.1.26) | -5.07 | 0.07494 |
| lmo0415 | LMRG_00107 | Peptidoglycan N-acetylglucosamine deacetylase (EC 3.5.1.-) | -3.8 | 0.07494 |
| lmo2451 | LMRG_01797 | Protein translocase membrane subunit SecG | -5.92 | 0.08937 |
| lmo0427 | LMRG_00119 | PTS system, IIB component | -5.18 | 0.09297 |
| lmo1372 | LMRG_00822 | Branched-chain alpha-keto acid dehydrogenase, E1 component, alpha subunit (EC 1.2.4.4) | -2.54 | 0.11167 |
| lmo0838 | LMRG_02261 | Sugar phosphate transporter | -2.77 | 0.11477 |
| lmo2136 | LMRG_02804 | PTS system, IIB component | -4.49 | 0.12267 |
| lmo2749 | LMRG_01947 | Para-aminobenzoate synthase, amidotransferase component (EC 2.6.1.85) | -3.99 | 0.13031 |
| lmo1401 | LMRG_00853 | 2',3'-cyclic-nucleotide 2'-phosphodiesterase, Bsub YmdB | -3.9 | 0.14245 |
| lmo0851 | LMRG_02274 | hypothetical protein | -3.1 | 0.14486 |
| lmo2560 | LMRG_02709 | DNA-directed RNA polymerase delta subunit (EC 2.7.7.6) | -3.48 | 0.1472 |
| lmo2791 | LMRG_01906 | Chromosome (plasmid) partitioning protein ParA | -4.6 | 0.15852 |
| lmo1849 | LMRG_00996 | Manganese ABC transporter, ATP-binding protein SitB | -3.94 | 0.16058 |
| lmo2055 | LMRG_01205 | ComK regulator YlbF | -4.07 | 0.18894 |
| lmo1587 | LMRG_01380 | Ornithine carbamoyltransferase (EC 2.1.3.3) | -2.93 | 0.20348 |
| lmo1503 | LMRG_01467 | UPF0297 protein YrzL | -3.5 | 0.22187 |
| lmo1805 | LMRG_00952 | Ribonuclease III (EC 3.1.26.3) | -2.48 | 0.22711 |
| lmo2679 | LMRG_02224 | Osmosensitive K+ channel histidine kinase KdpD | -2.14 | 0.23635 |
| lmo2853 | LMRG_02424 | RNA-binding protein Jag | -2.33 | 0.24124 |
| lmor09 | LMRG_05509 | SSU rRNA ## 16S rRNA, small subunit ribosomal RNA | -1.37 | 0.24601 |
| lmo1374 | LMRG_00824 | Dihydrolipoamide acyltransferase component of branched-chain alpha-keto acid dehydrogenase complex (EC 2.3.1.168) | -2.27 | 0.25064 |
| lmor12 | LMRG_05512 | SSU rRNA ## 16S rRNA, small subunit ribosomal RNA | -1.39 | 0.25515 |
| lmo0192 | LMRG_02614 | Pur operon repressor PurR | -2.43 | 0.25954 |
| lmo0956 | LMRG_02055 | N-acetylglucosamine-6-phosphate deacetylase (EC 3.5.1.25) | -1.96 | 0.26764 |
| lmo1291 | LMRG_00741 | Peptidoglycan O-acetyltransferase YrhL | -2.47 | 0.26798 |
| lmo1527 | LMRG_01443 | Protein translocase subunit SecD / Protein translocase subunit SecF | -2.63 | 0.29067 |
| lmor18 | LMRG_05518 | SSU rRNA ## 16S rRNA, small subunit ribosomal RNA | -1.37 | 0.30544 |
| lmor04 | LMRG_05504 | SSU rRNA ## 16S rRNA, small subunit ribosomal RNA | -1.43 | 0.30894 |
| lmor01 | LMRG_05501 | SSU rRNA ## 16S rRNA, small subunit ribosomal RNA | -1.41 | 0.31235 |
| lmo2449 | LMRG_01799 | 3'-to-5' exoribonuclease RNase R | -2.87 | 0.32244 |
| lmor15 | LMRG_05515 | SSU rRNA ## 16S rRNA, small subunit ribosomal RNA | -1.34 | 0.32244 |
| lmo0100 | LMRG_02349 | hypothetical protein | -6.93 | 0.32289 |
| lmo0577 | LMRG_00259 | Membrane protein | -6.01 | 0.32289 |
| lmo0398 | LMRG_00091 | PTS system, IIA component | -5.96 | 0.32289 |
| lmo2746 | LMRG_01950 | hypothetical protein | -5.95 | 0.32289 |
| lmo0916 | LMRG_02016 | PTS system, cellobiose-specific IIA component (EC 2.7.1.205) | -5.87 | 0.32289 |
| lmo1140 | LMRG_00583 | hypothetical protein | -5.74 | 0.32289 |
| lmo2170 | LMRG_01662 | Enoyl-[acyl-carrier-protein] reductase [FMN, NADH] (EC 1.3.1.9), FabK => refractory to triclosan | -5.58 | 0.32289 |
| lmo2473 | LMRG_01775 | FIG002813: LPPG:FO 2-phospho-L-lactate transferase like, CofD-like | -5.36 | 0.32289 |
| lmo0491 | LMRG_00172 | 3-dehydroquinate dehydratase I (EC 4.2.1.10) | -5.3 | 0.32289 |
| lmo0039 | LMRG_02468 | Carbamate kinase (EC 2.7.2.2) | -5.22 | 0.32289 |
| lmo0866 | LMRG_02289 | DEAD-box ATP-dependent RNA helicase DeaD (= CshA) (EC 3.6.4.13) | -4.85 | 0.32289 |
| lmo0586 | LMRG_00268 | cell surface protein precursor | -4.38 | 0.32289 |
| lmo1327 | LMRG_00777 | Ribosome-binding factor A | -4.34 | 0.32289 |
| lmo0720 | LMRG_00409 | hypothetical protein | -4.23 | 0.32289 |
| lmo0060 | LMRG_02490 | Putative secretion system component EssB/YukC | -4.2 | 0.32289 |
| lmo1777 | LMRG_02835 | HD domain protein | -4.09 | 0.32289 |
| lmo2833 | LMRG_01865 | Uncharacterized glycosyl hydrolase YcjT | -3.96 | 0.32289 |
| lmo1443 | LMRG_00895 | hypothetical protein | -3.94 | 0.32289 |
| lmo0647 | LMRG_00334 | hypothetical protein | -3.84 | 0.32289 |
| lmo0361 | LMRG_00053 | Twin-arginine translocation protein TatCy | -3.82 | 0.32289 |
| lmo0365 | LMRG_00057 | Ferrous iron transport permease EfeU | -3.55 | 0.32289 |
| lmo2387 | LMRG_02732 | hypothetical protein | -3.32 | 0.32289 |
| lmo1620 | LMRG_01346 | Putative dipeptidase YtjP | -3.24 | 0.32289 |
| lmo2499 | LMRG_01749 | Phosphate ABC transporter, substrate-binding protein PstS (TC 3.A.1.7.1) | -3.21 | 0.32289 |
| lmo1932 | LMRG_01079 | Heptaprenyl diphosphate synthase component I (EC 2.5.1.30) | -2.83 | 0.32289 |
| lmo1633 | LMRG_01333 | Anthranilate synthase, aminase component (EC 4.1.3.27) | -2.72 | 0.32289 |
| lmo1847 | LMRG_00994 | Manganese ABC transporter, periplasmic-binding protein SitA | -2.72 | 0.32289 |
| lmo0777 | LMRG_00465 | hypothetical protein | -2.55 | 0.32289 |
| lmo1363 | LMRG_00813 | (2E,6E)-farnesyl diphosphate synthase (EC 2.5.1.10) | -2.52 | 0.32289 |
| lmo2643 | LMRG_02188 | hypothetical protein | -2.47 | 0.32289 |
| lmo1434 | LMRG_00886 | Ribonuclease J2 (endoribonuclease in RNA processing) | -2.41 | 0.32289 |
| lmo2547 | LMRG_01700 | Homoserine dehydrogenase (EC 1.1.1.3) | -2.33 | 0.32289 |
| lmo1325 | LMRG_00775 | Translation initiation factor 2 | -2.25 | 0.32289 |
| lmo2777 | LMRG_01920 | Multidrug resistance transporter, Bcr/CflA family | -2.14 | 0.32289 |
| lmo1299 | LMRG_00749 | Glutamine synthetase type I (EC 6.3.1.2) | -1.95 | 0.32289 |
| lmo2005 | LMRG_01153 | Uncharacterized oxidoreductase, YajO family | -1.94 | 0.32289 |
| lmo2337 | LMRG_01506 | Transcriptional repressor of the fructose operon, DeoR family | -1.53 | 0.32289 |
| lmo2284 | LMRG_01548 | Putative tail or base plate protein gp19 [Bacteriophage A118] | -1.47 | 0.32289 |
| lmo1417 | LMRG_00869 | Uncharacterized MFS-type transporter YxiO | -1.21 | 0.32289 |
| lmo1557 | LMRG_02940 | Glutamyl-tRNA reductase (EC 1.2.1.70) | -0.73 | 0.32289 |
| lmo1878 | LMRG_01024 | Mn-dependent transcriptional regulator MntR | -3.14 | 0.32711 |
| lmo0219 | LMRG_02641 | tRNA(Ile)-lysidine synthetase (EC 6.3.4.19) / Hypoxanthine-guanine phosphoribosyltransferase (EC 2.4.2.8) | -1.26 | 0.32711 |
| lmo2800 | LMRG_01897 | Oxidoreductase, Gfo/Idh/MocA family | -3.42 | 0.33844 |
| lmo2194 | LMRG_01638 | Oligopeptide ABC transporter, permease protein OppC (TC 3.A.1.5.1) | -1.79 | 0.3427 |
| lmo2690 | LMRG_02237 | Transcriptional regulator, AcrR family | -1.84 | 0.41536 |
| lmo2847 | LMRG_02418 | Rhamnulose-1-phosphate aldolase (EC 4.1.2.19) | -2.02 | 0.42575 |
| lmo0582 | LMRG_00264 | P60 extracellular protein, invasion associated protein Iap | -2.15 | 0.42924 |
| lmo0300 | LMRG_02568 | beta-glucosidase (EC 3.2.1.21) | -2.87 | 0.43268 |
| lmo2493 | LMRG_01755 | Transcriptional repressor CzrA, ArsR family | -3.58 | 0.44049 |
| lmo0189 | LMRG_02754 | Veg protein | -2.75 | 0.44051 |
| lmo2250 | LMRG_01581 | ABC transporter, substrate-binding protein (cluster 3, basic aa/glutamine/opines) / ABC transporter, permease protein (cluster 3, basic aa/glutamine/opines) | -1.87 | 0.44051 |
| lmo2732 | LMRG_01964 | phosphosugar-binding protein | -2.72 | 0.47191 |
| lmo1172 | LMRG_00618 | Ethanolamine two-component response regulator | -3.38 | 0.4835 |
| lmo1710 | LMRG_02559 | Flavodoxin | -3.05 | 0.4838 |
| lmo0766 | LMRG_00454 | ABC transporter, permease protein 1 (cluster 1, maltose/g3p/polyamine/iron) | -2.65 | 0.4838 |
| lmo0558 | LMRG_00240 | 6-phosphogluconolactonase (EC 3.1.1.31) | -2.33 | 0.4838 |
| lmo1811 | LMRG_00958 | ATP-dependent DNA helicase RecG (EC 3.6.4.12) | -2.29 | 0.4838 |
| lmo2113 | LMRG_01267 | Coproheme decarboxylase HemQ (no EC) | -1 | 0.4838 |
| lmo1079 | LMRG_00541 | FIG028455: hypothetical protein | 1.41 | 0.4838 |
| lmo1665 | LMRG_01302 | hypothetical protein | -3.07 | 0.48525 |
| lmo0232 | LMRG_02674 | ATP-dependent Clp protease, ATP-binding subunit ClpC | -1.04 | 0.48525 |
| lmo0972 | LMRG_02071 | D-alanine--poly(phosphoribitol) ligase ACP subunit (EC 6.1.1.13) | -3.15 | 0.48999 |
| lmo2300 | LMRG_01532 | putative terminase large subunit | -3.48 | 0.5187 |
| lmo2048 | LMRG_01198 | FIG01269488: protein, clustered with ribosomal protein L32p | -1.97 | 0.52714 |
| lmo1494 | LMRG_01476 | 5'-methylthioadenosine nucleosidase (EC 3.2.2.16) @ S-adenosylhomocysteine nucleosidase (EC 3.2.2.9) | -2.3 | 0.55521 |
| lmo2162 | LMRG_01670 | Inosose dehydratase (EC 4.2.1.44) | -2.61 | 0.56132 |
| lmo1055 | LMRG_00517 | Dihydrolipoamide dehydrogenase of pyruvate dehydrogenase complex (EC 1.8.1.4) | -2.35 | 0.56345 |
| lmo0719 | LMRG_00408 | Transcriptional regulator, PadR family | -2.46 | 0.56571 |
| lmo1475 | LMRG_00928 | Heat-inducible transcription repressor HrcA | -1.46 | 0.56571 |
| lmo0674 | LMRG_00362 | hypothetical protein | -2.03 | 0.61174 |
| lmo2805 | LMRG_01891 | putative secreted protein | -2.26 | 0.61729 |
| lmo2001 | LMRG_01149 | PTS system, mannose-specific IIC component | -1.07 | 0.62088 |
| lmo0934 | LMRG_02033 | Epoxyqueuosine reductase (EC 1.17.99.6) QueG | -3.42 | 0.62255 |
| lmo2811 | LMRG_01887 | tRNA-5-carboxymethylaminomethyl-2-thiouridine(34) synthesis protein MnmE | -2.86 | 0.63034 |
| lmo2402 | LMRG_01846 | Uncharacterized DUF1027 domain protein YutD | -2.8 | 0.63034 |
| lmo1810 | LMRG_00957 | Transcription factor FapR | -2.76 | 0.63034 |
| lmo2169 | LMRG_01663 | hypothetical protein | -2.72 | 0.63034 |
| lmo2495 | LMRG_01753 | Phosphate ABC transporter, ATP-binding protein PstB (TC 3.A.1.7.1) | -2.69 | 0.63034 |
| lmo0740 | LMRG_00428 | hypothetical protein | -2.63 | 0.63034 |
| lmo0242 | LMRG_02664 | Uncharacterized protein YacP, similar to C-terminal domain of ribosome protection-type Tc-resistance proteins | -3.16 | 0.63188 |
| lmo1745 | LMRG_02526 | Two-component transcriptional response regulator, LuxR family | -1.96 | 0.63519 |
| lmo2515 | LMRG_01733 | Two-component transcriptional response regulator DegU, LuxR family | -2.61 | 0.66151 |
| lmo2525 | LMRG_01723 | MreB-like protein (Mbl protein) | -2.16 | 0.66637 |
| lmo2531 | LMRG_01717 | ATP synthase alpha chain (EC 3.6.3.14) | -2.59 | 0.69394 |
| lmo2398 | LMRG_01850 | Low temperature requirement C protein | -2.91 | 0.70726 |
| lmo2638 | LMRG_02183 | NADH dehydrogenase (EC 1.6.99.3) in cluster with putative pheromone precursor | -1.62 | 0.71522 |
| lmo0926 | LMRG_02025 | Transcriptional regulator, AcrR family | -1.9 | 0.72309 |
| lmo0371 | LMRG_00063 | Transcriptional regulator, GntR family | -1.4 | 0.72402 |
| lmo2545 | LMRG_01702 | Homoserine kinase (EC 2.7.1.39) | -2.38 | 0.75727 |
| lmo0163 | LMRG_02408 | Stage 0 sporulation protein YaaT | -2.73 | 0.76138 |
| lmo1377 | LMRG_00829 | Two-component transcriptional response regulator, OmpR family | -2.05 | 0.88656 |
| lmo1786 | LMRG_02825 | Internalin C | -1.98 | 0.90996 |
| lmo1854 | LMRG_01001 | Repressor CsoR of the copZA operon | -1.64 | 0.92124 |
| lmo1954 | LMRG_01101 | Phosphopentomutase (EC 5.4.2.7) | -1.6 | 0.92124 |
| lmo1848 | LMRG_00995 | Manganese ABC transporter, inner membrane permease protein SitD | -2.43 | 0.92432 |
| lmo2047 | LMRG_01197 | LSU ribosomal protein L32p @ LSU ribosomal protein L32p, zinc-dependent | -7.88 | 0.93367 |
| lmot02 | LMRG_05002 | tRNA-Val-TAC | -7.34 | 0.93367 |
| lmo0964 | LMRG_02063 | Flagellar motor switch protein FliN | -7.15 | 0.93367 |
| lmot38 | LMRG_05038 | tRNA-Val-TAC | -7.12 | 0.93367 |
| lmo2651 | LMRG_02196 | PTS system, IIA component | -6.33 | 0.93367 |
| lmo0888 | LMRG_02312 | Programmed cell death toxin YdcE | -6.04 | 0.93367 |
| lmo2259 | LMRG_01572 | PTS system, beta-glucoside-specific, IIA component | -5.91 | 0.93367 |
| lmo1220 | LMRG_00666 | Transcriptional regulator YodB, HxlR family | -5.6 | 0.93367 |
| lmo1469 | LMRG_00922 | SSU ribosomal protein S21p | -5.58 | 0.93367 |
| lmo0754 | LMRG_00442 | Bile acid 7-alpha dehydratase BaiE (EC 4.2.1.106) | -5.46 | 0.93367 |
| lmo0128 | LMRG_02377 | Holin | -5.06 | 0.93367 |
| lmo0131 | LMRG_02380 | hypothetical protein | -4.89 | 0.93367 |
| lmo0710 | LMRG_00399 | Flagellar basal-body rod protein FlgB | -4.85 | 0.93367 |
| lmo0953 | LMRG_02052 | hypothetical protein | -4.85 | 0.93367 |
| lmo2798 | LMRG_01899 | Hydrolase, haloacid dehalogenase-like family | -4.85 | 0.93367 |
| lmo0350 | LMRG_00041 | hypothetical protein | -4.7 | 0.93367 |
| lmo1157 | LMRG_00600 | Propanediol dehydratase reactivation factor small subunit | -4.66 | 0.93367 |
| lmo2729 | LMRG_01967 | Transcriptional regulator | -4.64 | 0.93367 |
| lmo1183 | LMRG_00629 | Protein clustered with ethanolamine utilization | -4.62 | 0.93367 |
| lmo2151 | LMRG_01681 | hypothetical protein | -4.62 | 0.93367 |
| lmo0212 | LMRG_02634 | Acetyltransferase, GNAT family | -4.57 | 0.93367 |
| lmo2080 | LMRG_01231 | hypothetical protein | -4.54 | 0.93367 |
| lmot44 | LMRG_05044 | tRNA-Gln-TTG | -4.53 | 0.93367 |
| lmo1358 | LMRG_00808 | Alkaline shock protein | -4.4 | 0.93367 |
| lmo0322 | LMRG_00014 | hypothetical protein | -4.17 | 0.93367 |
| lmo0877 | LMRG_02301 | Glucosamine-6-phosphate deaminase (EC 3.5.99.6) | -4.01 | 0.93367 |
| lmo2346 | LMRG_01497 | L-cystine ABC transporter, ATP-binding protein TcyN | -3.97 | 0.93367 |
| lmo0316 | LMRG_00009 | Hydroxyethylthiazole kinase (EC 2.7.1.50) | -3.87 | 0.93367 |
| lmo2369 | LMRG_02714 | General stress protein 13 | -3.78 | 0.93367 |
| lmo0117 | LMRG_02366 | Listeria protein LmaB, associated with virulence | -3.74 | 0.93367 |
| lmo0657 | LMRG_00344 | hypothetical protein | -3.74 | 0.93367 |
| lmo1308 | LMRG_00758 | SAM-dependent methyltransferase | -3.74 | 0.93367 |
| lmo0167 | LMRG_02412 | 16S rRNA (cytidine(1402)-2'-O)-methyltransferase (EC 2.1.1.198) | -3.72 | 0.93367 |
| lmot25 | LMRG_05025 | tRNA-Asp-GTC | -3.67 | 0.93367 |
| lmo2227 | LMRG_01605 | Efflux ABC transporter, ATP-binding protein | -3.66 | 0.93367 |
| lmot15 | LMRG_05015 | tRNA-Asp-GTC | -3.66 | 0.93367 |
| lmo0905 | LMRG_02005 | hypothetical protein | -3.65 | 0.93367 |
| lmo0542 | LMRG_00224 | PTS system, glucitol/sorbitol-specific IIA component (EC 2.7.1.198) | -3.62 | 0.93367 |
| lmo1201 | LMRG_00647 | Uroporphyrinogen-III methyltransferase (EC 2.1.1.107) / Uroporphyrinogen-III synthase (EC 4.2.1.75) | -3.6 | 0.93367 |
| lmo1317 | LMRG_00767 | 1-deoxy-D-xylulose 5-phosphate reductoisomerase (EC 1.1.1.267) | -3.54 | 0.93367 |
| lmo0413 | LMRG_00105 | hypothetical protein | -3.53 | 0.93367 |
| lmo2009 | LMRG_01157 | ABC transporter, permease protein 1 (cluster 1, maltose/g3p/polyamine/iron) | -3.46 | 0.93367 |
| lmo0631 | LMRG_00314 | PTS system, fructose-specific IIA component (EC 2.7.1.202) | -3.45 | 0.93367 |
| lmot54 | LMRG_05054 | tRNA-Asp-GTC | -3.44 | 0.93367 |
| lmo1195 | LMRG_00641 | Cobalt-precorrin-7 (C5)-methyltransferase (EC 2.1.1.289) | -3.4 | 0.93367 |
| lmo2491 | LMRG_01757 | Nucleotidase YfbR, HD superfamily | -3.4 | 0.93367 |
| lmo1324 | LMRG_00774 | Ribosomal protein L7Ae family protein YlxQ | -3.35 | 0.93367 |
| lmo0474 | LMRG_00155 | hypothetical protein | -3.33 | 0.93367 |
| lmo1483 | LMRG_00936 | dCMP deaminase (EC 3.5.4.12) @ Late competence protein ComEB | -3.32 | 0.93367 |
| lmo1998 | LMRG_01146 | Putative glucosamine-fructose-6-phosphate aminotransferase | -3.27 | 0.93367 |
| lmo0188 | LMRG_02753 | SSU rRNA (adenine(1518)-N(6)/adenine(1519)-N(6))-dimethyltransferase (EC 2.1.1.182) | -3.24 | 0.93367 |
| lmo0562 | LMRG_00244 | Phosphoribosyl-AMP cyclohydrolase (EC 3.5.4.19) | -3.22 | 0.93367 |
| lmot01 | LMRG_05001 | tRNA-Lys-CTT | -3.21 | 0.93367 |
| lmo0451 | LMRG_00143 | hypothetical protein | -3.2 | 0.93367 |
| lmo2297 | LMRG_01535 | Phage capsid scaffolding protein | -3.2 | 0.93367 |
| lmo1336 | LMRG_00786 | 5-formyltetrahydrofolate cyclo-ligase (EC 6.3.3.2) | -3.1 | 0.93367 |
| lmo1269 | LMRG_00719 | Signal peptidase I (EC 3.4.21.89) | -3.08 | 0.93367 |
| lmo1653 | LMRG_01314 | Putative cellsurface protein | -3.04 | 0.93367 |
| lmo2182 | LMRG_01650 | Heme transporter analogous to IsdDEF, ATP-binding protein | -2.99 | 0.93367 |
| lmo2271 | LMRG_01558 | hypothetical protein | -2.96 | 0.93367 |
| lmo1927 | LMRG_01074 | 3-dehydroquinate synthase (EC 4.2.3.4) | -2.95 | 0.93367 |
| lmo2193 | LMRG_01639 | Oligopeptide ABC transporter, ATP-binding protein OppD (TC 3.A.1.5.1) | -2.93 | 0.93367 |
| lmo1608 | LMRG_01358 | FIG002434: Uncharacterized protein YtpQ | -2.92 | 0.93367 |
| lmo0004 | LMRG_02432 | Uncharacterized S4 RNA-binding-domain protein YbcJ | -2.86 | 0.93367 |
| lmo2699 | LMRG_01998 | Cof-like hydrolase | -2.84 | 0.93367 |
| lmo1155 | LMRG_00598 | Propanediol dehydratase small subunit (EC 4.2.1.28) | -2.84 | 0.93367 |
| lmo1526 | LMRG_01444 | Uncharacterized membrane protein YrvD | -2.82 | 0.93367 |
| lmo2647 | LMRG_02192 | Creatinine amidohydrolase (EC 3.5.2.10) | -2.74 | 0.93367 |
| lmo0971 | LMRG_02070 | Poly(glycerophosphate chain) D-alanine transfer protein DltD | -2.73 | 0.93367 |
| lmo2309 | LMRG_01524 | Phage exonuclease (EC 3.1.11.3) | -2.7 | 0.93367 |
| lmo1139 | LMRG_00582 | hypothetical protein | -2.67 | 0.93367 |
| lmo1553 | LMRG_01415 | Glutamate-1-semialdehyde 2,1-aminomutase (EC 5.4.3.8) | -2.66 | 0.93367 |
| lmo2613 | LMRG_02157 | LSU ribosomal protein L15p (L27Ae) | -2.66 | 0.93367 |
| lmo2648 | LMRG_02193 | phosphotriesterase family protein | -2.65 | 0.93367 |
| lmo1956 | LMRG_01103 | Ferric uptake regulation protein FUR | -2.63 | 0.93367 |
| lmo2029 | LMRG_01178 | Cell division integral membrane protein, YggT and half-length relatives | -2.61 | 0.93367 |
| lmo1627 | LMRG_01339 | Tryptophan synthase alpha chain (EC 4.2.1.20) | -2.58 | 0.93367 |
| #N/A | LMRG_02137 | CRISPR-associated protein Cas1 | -2.55 | 0.93367 |
| lmo2509 | LMRG_01739 | Peptide chain release factor 2 @ programmed frameshift-containing | -2.52 | 0.93367 |
| lmo1152 | LMRG_00595 | Propanediol utilization polyhedral body protein PduB | -2.51 | 0.93367 |
| lmo1929 | LMRG_01076 | Nucleoside diphosphate kinase (EC 2.7.4.6) | -2.49 | 0.93367 |
| lmo0104 | LMRG_02353 | hypothetical protein | -2.47 | 0.93367 |
| lmo1972 | LMRG_01119 | PTS system, IIB component | -2.47 | 0.93367 |
| lmo1958 | LMRG_01105 | Ferrichrome transport system permease protein FhuB | -2.41 | 0.93367 |
| lmo1948 | LMRG_01095 | DNA-binding response regulator ResD | -2.38 | 0.93367 |
| lmo2180 | LMRG_01652 | hypothetical protein | -2.35 | 0.93367 |
| lmo0534 | LMRG_00216 | FIG00519347: Ribonucleotide reductase-like protein | -2.31 | 0.93367 |
| lmo0961 | LMRG_02060 | Uncharacterized protease YrrO | -2.31 | 0.93367 |
| lmo2644a | LMRG_02190 | hypothetical protein | -2.31 | 0.93367 |
| lmo0620 | LMRG_00303 | hypothetical protein | -2.28 | 0.93367 |
| lmo2359 | LMRG_01483 | Cof-like hydrolase | -2.28 | 0.93367 |
| lmo0561 | LMRG_00243 | Phosphoribosyl-ATP pyrophosphatase (EC 3.6.1.31) | -2.27 | 0.93367 |
| lmo1501 | LMRG_01469 | UPF0473 protein YrzB | -2.27 | 0.93367 |
| lmo0463 | LMRG_00152 | FIG00775752: hypothetical protein | -2.27 | 0.93367 |
| lmo2598 | LMRG_02142 | tRNA pseudouridine(38-40) synthase (EC 5.4.99.12) | -2.24 | 0.93367 |
| lmo2717 | LMRG_01979 | Cytochrome d ubiquinol oxidase subunit II (EC 1.10.3.-) | -2.16 | 0.93367 |
| lmo0337 | LMRG_00029 | hypothetical protein | -2.14 | 0.93367 |
| lmo0422 | LMRG_00114 | Transcriptional regulator, PadR family | -2.14 | 0.93367 |
| lmo0750 | LMRG_00438 | hypothetical protein | -2.14 | 0.93367 |
| lmo1821 | LMRG_00968 | Protein serine/threonine phosphatase PrpC, regulation of stationary phase | -2.14 | 0.93367 |
| lmo1747 | LMRG_02524 | ABC transporter-like sensor ATP-binding protein | -2.1 | 0.93367 |
| lmo1294 | LMRG_00744 | tRNA dimethylallyltransferase (EC 2.5.1.75) | -2.05 | 0.93367 |
| lmo1675 | LMRG_01292 | 2-succinyl-5-enolpyruvyl-6-hydroxy-3-cyclohexene-1-carboxylic-acid synthase (EC 2.2.1.9) | -2.05 | 0.93367 |
| lmo2386 | LMRG_02731 | Uncharacterized membrane protein YuiD | -2.02 | 0.93367 |
| lmo2520 | LMRG_01728 | O-succinylbenzoate synthase (EC 4.2.1.113) | -2 | 0.93367 |
| lmo1208 | LMRG_00654 | Cobyric acid synthase (EC 6.3.5.10) | -1.98 | 0.93367 |
| lmo0767 | LMRG_00455 | ABC transporter, permease protein 2 (cluster 1, maltose/g3p/polyamine/iron) | -1.96 | 0.93367 |
| lmo0859 | LMRG_02282 | Multiple sugar ABC transporter, substrate-binding protein MsmE | -1.96 | 0.93367 |
| lmo2498 | LMRG_01750 | Phosphate ABC transporter, permease protein PstC (TC 3.A.1.7.1) | -1.94 | 0.93367 |
| lmo2523 | LMRG_01725 | Single-stranded DNA-binding protein | -1.94 | 0.93367 |
| lmo0965 | LMRG_02064 | Adenylate cyclase (EC 4.6.1.1) | -1.87 | 0.93367 |
| #N/A | LMRG_00561 | hypothetical protein | -1.87 | 0.93367 |
| lmo0338 | LMRG_02932 | FIG00774315: hypothetical protein | -1.87 | 0.93367 |
| lmot04 | LMRG_05004 | tRNA-Lys-TTT | -1.86 | 0.93367 |
| lmot36 | LMRG_05036 | tRNA-Lys-TTT | -1.84 | 0.93367 |
| lmor07 | LMRG_05507 | 5S rRNA ## 5S ribosomal RNA | -1.82 | 0.93367 |
| lmo1575 | LMRG_01392 | 3'-to-5' oligoribonuclease A, Bacillus type | -1.81 | 0.93367 |
| lmo1673 | LMRG_01294 | Naphthoate synthase (EC 4.1.3.36) | -1.77 | 0.93367 |
| lmo1439 | LMRG_00891 | Superoxide dismutase [Mn] (EC 1.15.1.1) | -1.76 | 0.93367 |
| lmo0015 | LMRG_02444 | Cytochrome aa3-600 menaquinol oxidase subunit III | -1.75 | 0.93367 |
| lmo1746 | LMRG_02525 | ABC transporter-like sensor and permease protein | -1.75 | 0.93367 |
| lmot45 | LMRG_05045 | tRNA-Lys-TTT | -1.75 | 0.93367 |
| lmo2477 | LMRG_01771 | UDP-glucose 4-epimerase (EC 5.1.3.2) | -1.73 | 0.93367 |
| lmo0556 | LMRG_00238 | Phosphoglycerate mutase family, Lmo0556 homolog | -1.72 | 0.93367 |
| lmo1030 | LMRG_00491 | Unknown pentose utilization regulator, LacI family | -1.71 | 0.93367 |
| lmo0915 | LMRG_02015 | PTS system, cellobiose-specific IIC component | -1.7 | 0.93367 |
| lmo1931 | LMRG_01078 | Demethylmenaquinone methyltransferase (EC 2.1.1.163) | -1.7 | 0.93367 |
| lmo1425 | LMRG_00877 | Choline ABC transport system, permease protein OpuBD | -1.69 | 0.93367 |
| lmo0352 | LMRG_00043 | Transcriptional regulator, DeoR family | -1.68 | 0.93367 |
| lmo1462 | LMRG_00914 | GTP-binding protein Era | -1.67 | 0.93367 |
| lmo2575 | LMRG_02693 | Cobalt/zinc/cadmium resistance protein CzcD | -1.66 | 0.93367 |
| lmo0652 | LMRG_00339 | Acetyltransferase, GNAT family | -1.65 | 0.93367 |
| lmo2794 | LMRG_01903 | Chromosome (plasmid) partitioning protein ParB-2 | -1.65 | 0.93367 |
| lmo1497 | LMRG_01473 | Uridine kinase (EC 2.7.1.48) | -1.62 | 0.93367 |
| lmo0038 | LMRG_02467 | Agmatine deiminase (EC 3.5.3.12) | -1.61 | 0.93367 |
| lmo0682 | LMRG_00370 | Flagellar synthesis regulator FleN | -1.59 | 0.93367 |
| lmo1727 | LMRG_02544 | Transcriptional regulator, LacI family | -1.59 | 0.93367 |
| lmo2840 | LMRG_01858 | Uncharacterized glycosyltransferase YcjM | -1.59 | 0.93367 |
| lmo1373 | LMRG_00823 | Branched-chain alpha-keto acid dehydrogenase, E1 component, beta subunit (EC 1.2.4.4) | -1.58 | 0.93367 |
| lmo2263 | LMRG_01568 | Putative phosphatase YitU | -1.58 | 0.93367 |
| lmo2804 | LMRG_01892 | hypothetical protein | -1.57 | 0.93367 |
| lmor03 | LMRG_05503 | 5S rRNA ## 5S ribosomal RNA | -1.56 | 0.93367 |
| lmo2779 | LMRG_01918 | GTP-binding and nucleic acid-binding protein YchF | -1.55 | 0.93367 |
| lmo2031 | LMRG_01180 | Pyridoxal phosphate-containing protein YggS | -1.53 | 0.93367 |
| lmo2438 | LMRG_01810 | hypothetical protein | -1.53 | 0.93367 |
| lmo2307 | LMRG_01526 | Phage DNA replication protein O | -1.51 | 0.93367 |
| lmo2306 | LMRG_01527 | Protein gp32 [Listeria phage 2389] | -1.47 | 0.93367 |
| lmo1676 | LMRG_01291 | Isochorismate synthase (EC 5.4.4.2) @ Menaquinone-specific isochorismate synthase (EC 5.4.4.2) | -1.46 | 0.93367 |
| lmo2049 | LMRG_01199 | UPF0348 protein family | -1.46 | 0.93367 |
| lmo0840 | LMRG_02263 | Transcriptional regulator, MarR family | -1.45 | 0.93367 |
| lmo0985 | LMRG_02085 | hypothetical protein | -1.45 | 0.93367 |
| lmo0769 | LMRG_00457 | putative alpha-1,6-mannanase | -1.44 | 0.93367 |
| lmo1674 | LMRG_01293 | 2-succinyl-6-hydroxy-2,4-cyclohexadiene-1-carboxylate synthase (EC 4.2.99.20) | -1.44 | 0.93367 |
| lmo2534 | LMRG_01714 | ATP synthase F0 sector subunit c (EC 3.6.3.14) | -1.43 | 0.93367 |
| lmot19 | LMRG_05019 | tRNA-Ser-GCT | -1.42 | 0.93367 |
| lmo1525 | LMRG_01445 | Single-stranded-DNA-specific exonuclease RecJ | -1.37 | 0.93367 |
| lmo0630 | LMRG_00313 | Putative transcriptional antiterminator, BglG family / PTS system, IIA component | -1.36 | 0.93367 |
| lmo1214 | LMRG_00660 | hypothetical protein | -1.36 | 0.93367 |
| lmo0328 | LMRG_00022 | hypothetical protein | -1.32 | 0.93367 |
| lmo0599 | LMRG_00282 | Transcriptional regulator, PadR family | -1.31 | 0.93367 |
| lmo1160 | LMRG_00603 | Phosphate propanoyltransferase (EC 2.3.1.222) | -1.3 | 0.93367 |
| lmo2848 | LMRG_02419 | L-rhamnose isomerase (EC 5.3.1.14) | -1.3 | 0.93367 |
| lmo1741 | LMRG_02530 | Two-component system sensor histidine kinase | -1.29 | 0.93367 |
| lmo0894 | LMRG_02318 | Serine-protein kinase RsbW (EC 2.7.11.1) | -1.27 | 0.93367 |
| lmo1143 | LMRG_00586 | Propanediol utilization polyhedral body protein PduT | -1.26 | 0.93367 |
| lmo1387 | LMRG_00839 | Pyrroline-5-carboxylate reductase (EC 1.5.1.2), ProG-like | -1.23 | 0.93367 |
| lmo0547 | LMRG_00229 | Sorbitol operon transcription regulator | -1.18 | 0.93367 |
| lmo2486 | LMRG_01762 | hypothetical protein | -1.18 | 0.93367 |
| lmo1003 | LMRG_02103 | Phosphoenolpyruvate-protein phosphotransferase of PTS system (EC 2.7.3.9) | -1.17 | 0.93367 |
| lmo2392 | LMRG_02737 | DUF1450 superfamily protein | -1.14 | 0.93367 |
| lmo0958 | LMRG_02057 | Predicted transcriptional regulator of N-Acetylglucosamine utilization, GntR family | -1.13 | 0.93367 |
| lmo0584 | LMRG_00266 | UPF0118 membrane protein YubA | -1.12 | 0.93367 |
| lmo1063 | LMRG_00525 | ABC-type antimicrobial peptide transport system, ATPase component | -1.07 | 0.93367 |
| lmo1084 | LMRG_00546 | dTDP-4-dehydrorhamnose reductase (EC 1.1.1.133) | -1.05 | 0.93367 |
| lmo2196 | LMRG_01636 | ABC transporter, substrate-binding protein (cluster 5, nickel/peptides/opines) | -1.02 | 0.93367 |
| lmo0007 | LMRG_02435 | DNA gyrase subunit A (EC 5.99.1.3) | -1.01 | 0.93367 |
| lmo0301 | LMRG_02567 | PTS system, beta-glucoside-specific, IIA component | -0.98 | 0.93367 |
| lmo0676 | LMRG_00364 | Flagellar biosynthesis protein FliP | -0.95 | 0.93367 |
| lmo1857 | LMRG_01004 | UPF0346 protein YozE | -0.93 | 0.93367 |
| lmo2299 | LMRG_01533 | putative portal protein | -0.89 | 0.93367 |
| lmo1695 | LMRG_02769 | L-O-lysylphosphatidylglycerol synthase (EC 2.3.2.3) | -0.85 | 0.93367 |
| lmo1955 | LMRG_01102 | Site-specific tyrosine recombinase XerD | -0.81 | 0.93367 |
| lmo2024 | LMRG_01173 | Quinolinate phosphoribosyltransferase [decarboxylating] (EC 2.4.2.19) | -0.56 | 0.93367 |
| lmo1672 | LMRG_01295 | O-succinylbenzoic acid--CoA ligase (EC 6.2.1.26) | -0.48 | 0.93367 |
| lmo2184 | LMRG_01648 | Heme transporter IsdDEF, lipoprotein IsdE | -0.47 | 0.93367 |
| lmo2521 | LMRG_01727 | N-acetylmannosaminyltransferase (EC 2.4.1.187) | -0.47 | 0.93367 |
| lmo2348 | LMRG_01495 | L-cystine ABC transporter, permease protein TcyL | -0.44 | 0.93367 |
| lmo1484 | LMRG_00937 | Late competence protein ComEA, DNA receptor | -0.42 | 0.93367 |
| lmo1338 | LMRG_00788 | Uncharacterized protein YqgQ | -0.41 | 0.93367 |
| lmo1901 | LMRG_01048 | Pantoate--beta-alanine ligase (EC 6.3.2.1) | -0.36 | 0.93367 |
| lmo2366 | LMRG_02711 | Transcriptional regulator, DeoR family | -0.36 | 0.93367 |
| lmo1376 | LMRG_00828 | 6-phosphogluconate dehydrogenase, decarboxylating (EC 1.1.1.44) | -0.32 | 0.93367 |
| lmo0969 | LMRG_02068 | Similar to ribosomal large subunit pseudouridine synthase D, Bacillus subtilis YjbO type | -0.29 | 0.93367 |
| lmo1872 | LMRG_01019 | 23S rRNA (guanine(748)-N(1))-methyltransferase (EC 2.1.1.188) | -0.17 | 0.93367 |
| lmo1819 | LMRG_00966 | Ribosome small subunit biogenesis RbfA-release protein RsgA | -0.15 | 0.93367 |
| lmo1331 | LMRG_00781 | Polyribonucleotide nucleotidyltransferase (EC 2.7.7.8) | -0.14 | 0.93367 |
| lmo1928 | LMRG_01075 | Chorismate synthase (EC 4.2.3.5) | -0.14 | 0.93367 |
| lmo1923 | LMRG_01070 | 3-phosphoshikimate 1-carboxyvinyltransferase (EC 2.5.1.19) | -0.13 | 0.93367 |
| lmo2336 | LMRG_01507 | 1-phosphofructokinase (EC 2.7.1.56) | -0.12 | 0.93367 |
| lmo1096 | LMRG_00558 | GMP synthase [glutamine-hydrolyzing], amidotransferase subunit (EC 6.3.5.2) / GMP synthase [glutamine-hydrolyzing], ATP pyrophosphatase subunit (EC 6.3.5.2) | -0.11 | 0.93367 |
| lmo1978 | LMRG_02980 | Glucose-6-phosphate 1-dehydrogenase (EC 1.1.1.49) | -0.1 | 0.93367 |
| lmo2704 | LMRG_01993 | DNA polymerase III subunits gamma and tau (EC 2.7.7.7) | -0.1 | 0.93367 |
| lmo1473 | LMRG_00926 | Chaperone protein DnaK | -0.06 | 0.93367 |
| lmo0784 | LMRG_00472 | PTS system, mannose-specific IIA component (EC 2.7.1.191) | -2.88 | 0.9343 |
| lmo2698 | LMRG_01999 | Transcriptional regulator, RpiR family | -1.56 | 0.93816 |
| lmo1262 | LMRG_00711 | Phage DNA binding protein | -1.41 | 0.93816 |
| lmo0921 | LMRG_02021 | UPF0703 protein YcgQ | -1.32 | 0.93816 |
| lmo1365 | LMRG_00815 | 1-deoxy-D-xylulose 5-phosphate synthase (EC 2.2.1.7) | -1.32 | 0.93816 |
| lmo1423 | LMRG_00875 | hypothetical protein | -1.17 | 0.93816 |
| lmo2685 | LMRG_02230 | PTS system, beta-glucoside-specific IIA component | -1.85 | 0.94283 |
| lmo0243 | LMRG_02663 | RNA polymerase sporulation specific sigma factor SigH | -1.69 | 0.94283 |
| lmo1050 | LMRG_00511 | hypothetical protein | -1.56 | 0.94283 |
| lmo0615 | LMRG_00298 | hypothetical protein | -1.33 | 0.94283 |
| lmo1062 | LMRG_00524 | ABC transporter, permease protein | -1.53 | 0.94342 |
| lmo2530 | LMRG_01718 | ATP synthase gamma chain (EC 3.6.3.14) | -1.27 | 0.94475 |
| lmo1267 | LMRG_00716 | Cell division trigger factor (EC 5.2.1.8) | -1.93 | 0.94592 |
| lmo1567 | LMRG_01400 | Citrate synthase (si) (EC 2.3.3.1) | -1.19 | 0.94592 |
| lmo0681 | LMRG_00369 | Flagellar biosynthesis protein FlhF | -1.57 | 0.95515 |
| lmo2683 | LMRG_02228 | PTS system, cellobiose-specific IIB component (EC 2.7.1.205) | -1.42 | 0.95515 |
| lmo2405 | LMRG_01843 | hypothetical protein | -1.6 | 0.96356 |
| lmo1148 | LMRG_00591 | Cobalamin synthase (EC 2.7.8.26) | -2.01 | 0.96804 |
| lmo0957 | LMRG_02056 | Glucosamine-6-phosphate deaminase (EC 3.5.99.6) | -1.45 | 0.96804 |
| lmo2774 | LMRG_02899 | FIG00775122: hypothetical protein | -1.31 | 0.96804 |
| lmo1818 | LMRG_00965 | Ribulose-phosphate 3-epimerase (EC 5.1.3.1) | -1.82 | 0.96979 |
| lmo0235 | LMRG_02671 | 2-C-methyl-D-erythritol 4-phosphate cytidylyltransferase (EC 2.7.7.60) | -1.61 | 0.96979 |
| lmo0506 | LMRG_00187 | Galactitol-1-phosphate 5-dehydrogenase (EC 1.1.1.251) | -1.32 | 0.9945 |
| lmo0270 | LMRG_02606 | hypothetical protein | -1.24 | 0.9945 |
| lmo0164 | LMRG_02409 | DNA replication intiation control protein YabA | -1.29 | 0.99709 |
| lmos49 | #N/A | hypothetical protein | -2.79 | 1 |
| lmo1181 | LMRG_00627 | ATP:Cob(I)alamin adenosyltransferase (EC 2.5.1.17) @ ATP:Cob(I)alamin adenosyltransferase (EC 2.5.1.17), ethanolamine utilization | -2.66 | 1 |
| lmo1213 | LMRG_00659 | Transcriptional regulator, PadR family | -2.37 | 1 |
| lmo2098 | LMRG_01249 | PTS system, galactitol-specific IIA component (EC 2.7.1.200) | -2.35 | 1 |
| lmo0026 | LMRG_02455 | Cytoplasmic copper homeostasis protein CutC | -2.05 | 1 |
| lmo2760a | LMRG_01935 | hypothetical protein | -2.04 | 1 |
| lmo1133 | LMRG_00575 | hypothetical protein | -2.02 | 1 |
| lmo1395 | LMRG_00847 | Transcriptional regulator in cluster with unspecified monosaccharide ABC transport system | -1.97 | 1 |
| lmo1022 | LMRG_02122 | Cell envelope stress response system LiaFSR, response regulator LiaR(VraR) | -1.92 | 1 |
| lmo2016 | LMRG_01165 | Cold shock protein of CSP family | -1.92 | 1 |
| lmo1517 | LMRG_01453 | Nitrogen regulatory protein P-II | -1.9 | 1 |
| lmo2829 | LMRG_01869 | Nitroreductase family protein | -1.84 | 1 |
| lmo1785 | LMRG_02827 | Translation initiation factor 3 | -1.81 | 1 |
| lmo0058 | LMRG_02488 | Putative secretion system component EssA | -1.74 | 1 |
| lmo1604 | LMRG_01363 | Alkyl hydroperoxide reductase subunit C-like protein | -1.72 | 1 |
| lmo1749 | LMRG_02522 | Shikimate kinase I (EC 2.7.1.71) | -1.7 | 1 |
| lmo2074 | LMRG_01225 | hypothetical protein | -1.69 | 1 |
| lmo2703 | LMRG_01994 | Nucleoid-associated protein YaaK | -1.66 | 1 |
| lmo1137 | LMRG_00580 | hypothetical protein | -1.65 | 1 |
| lmo0162 | LMRG_02407 | DNA polymerase III delta prime subunit (EC 2.7.7.7) | -1.63 | 1 |
| lmo0662 | LMRG_00349 | Novel pyridoxal kinase, thiD family (EC 2.7.1.35) | -1.63 | 1 |
| lmo0075 | LMRG_02326 | Probable carboxyvinyl-carboxyphosphonate phosphorylmutase (EC 2.7.8.23) | -1.62 | 1 |
| lmo1809 | LMRG_00956 | Phosphate:acyl-ACP acyltransferase PlsX (EC 2.3.1.n2) | -1.56 | 1 |
| lmo0260 | LMRG_02649 | hypothetical protein | -1.55 | 1 |
| lmo0563 | LMRG_00245 | Imidazole glycerol phosphate synthase cyclase subunit | -1.55 | 1 |
| lmo1292 | LMRG_00742 | Glycerophosphoryl diester phosphodiesterase (EC 3.1.4.46) | -1.54 | 1 |
| lmo2004 | LMRG_01152 | Transcriptional regulator, GntR family | -1.53 | 1 |
| lmo0635 | LMRG_00318 | 2-haloalkanoic acid dehalogenase (EC 3.8.1.2) | -1.5 | 1 |
| lmo2158 | LMRG_01674 | hypothetical protein | -1.49 | 1 |
| lmo0317 | LMRG_00010 | Hydroxymethylpyrimidine kinase (EC 2.7.1.49) @ Hydroxymethylpyrimidine phosphate kinase ThiD (EC 2.7.4.7) | -1.48 | 1 |
| lmo0091 | LMRG_02340 | ATP synthase gamma chain (EC 3.6.3.14) | -1.45 | 1 |
| lmo1348 | LMRG_00798 | Aminomethyltransferase (glycine cleavage system T protein) (EC 2.1.2.10) | -1.45 | 1 |
| lmo1075 | LMRG_00537 | Teichoic acid export ATP-binding protein TagH (EC 3.6.3.40) | -1.44 | 1 |
| lmo0485 | LMRG_00166 | Putative nitroreductase family protein SACOL0874 | -1.43 | 1 |
| lmo2764 | LMRG_01931 | ROK family protein | -1.43 | 1 |
| lmo2091 | LMRG_01242 | Argininosuccinate lyase (EC 4.3.2.1) | -1.41 | 1 |
| lmo1025 | LMRG_02125 | hypothetical protein | -1.4 | 1 |
| lmo0119 | LMRG_02368 | hypothetical protein | -1.36 | 1 |
| lmo0546 | LMRG_00228 | Sorbitol-6-phosphate 2-dehydrogenase (EC 1.1.1.140) | -1.36 | 1 |
| lmo1798 | LMRG_02822 | poly (glycerol-phosphate) alpha-glucosyltransferase | -1.36 | 1 |
| lmo0133 | LMRG_02382 | Uncharacterized protein YjdI | -1.35 | 1 |
| lmo2510 | LMRG_01738 | Protein translocase subunit SecA | -1.35 | 1 |
| lmo0307 | LMRG_00004 | hypothetical protein | -1.35 | 1 |
| lmo1095 | LMRG_00557 | PTS system, IIB component | -1.33 | 1 |
| lmo0395 | LMRG_00088 | Acetyltransferase, GNAT family | -1.29 | 1 |
| lmo0731 | LMRG_00419 | hypothetical protein | -1.29 | 1 |
| lmo0418 | LMRG_00110 | hypothetical protein | -1.27 | 1 |
| lmo0967 | LMRG_02066 | GTP pyrophosphokinase (EC 2.7.6.5) | -1.27 | 1 |
| lmo0670 | LMRG_00358 | hypothetical protein | -1.26 | 1 |
| lmo0651 | LMRG_00338 | Transcriptional regulator, GntR family | -1.25 | 1 |
| lmo0527 | LMRG_00208 | Membrane protein | -1.24 | 1 |
| lmo1524 | LMRG_01446 | Adenine phosphoribosyltransferase (EC 2.4.2.7) | -1.24 | 1 |
| lmo1863 | LMRG_01010 | DegV family protein | -1.24 | 1 |
| lmo2471 | LMRG_01777 | NADH:flavin oxidoreductase | -1.24 | 1 |
| lmo2822 | LMRG_01876 | hypothetical protein | -1.24 | 1 |
| lmo2419 | LMRG_01829 | Methionine ABC transporter ATP-binding protein | -1.23 | 1 |
| lmo0247 | LMRG_02659 | hypothetical protein | -1.22 | 1 |
| lmo1881 | LMRG_01028 | DNA polymerase I 5'-3' exonuclease domain | -1.22 | 1 |
| lmo0273 | LMRG_02603 | Acetyltransferase, GNAT family | -1.2 | 1 |
| lmo1832 | LMRG_00979 | Orotidine 5'-phosphate decarboxylase (EC 4.1.1.23) | -1.2 | 1 |
| lmo1396 | LMRG_00848 | CDP-diacylglycerol--glycerol-3-phosphate 3-phosphatidyltransferase (EC 2.7.8.5) | -1.19 | 1 |
| lmo1994 | LMRG_01142 | Transcriptional regulator, LacI family | -1.19 | 1 |
| lmo2190 | LMRG_01642 | ClpCP protease substrate adapter protein MecA | -1.18 | 1 |
| lmo1753 | LMRG_02518 | Transcription regulator [contains diacylglycerol kinase catalytic domain] | -1.17 | 1 |
| lmo2701 | LMRG_01996 | Uncharacterized protein YaaL | -1.17 | 1 |
| lmo0436 | LMRG_00128 | Rrf2 family transcriptional regulator, group III | -1.16 | 1 |
| lmo0886 | LMRG_02310 | Alanine racemase (EC 5.1.1.1) | -1.16 | 1 |
| lmo1776 | LMRG_02495 | hypothetical protein | -1.16 | 1 |
| lmo2604 | LMRG_02148 | hypothetical protein | -1.16 | 1 |
| lmo2849 | LMRG_02420 | Rhamnulokinase (EC 2.7.1.5) | -1.16 | 1 |
| lmo1489 | LMRG_00942 | RNA-binding protein YhbY | -1.15 | 1 |
| lmo1628 | LMRG_01338 | Tryptophan synthase beta chain (EC 4.2.1.20) | -1.15 | 1 |
| lmo1900 | LMRG_01047 | Aspartate 1-decarboxylase (EC 4.1.1.11) | -1.15 | 1 |
| lmo1247 | LMRG_00693 | hypothetical protein | -1.14 | 1 |
| lmo1293 | LMRG_00743 | Aerobic glycerol-3-phosphate dehydrogenase (EC 1.1.5.3) | -1.14 | 1 |
| lmo0267 | LMRG_02609 | Putative ring-cleaving dioxygenase MhqA | -1.13 | 1 |
| lmo0808 | LMRG_02789 | Spermidine/putrescine import ABC transporter permease protein PotB (TC 3.A.1.11.1) | -1.13 | 1 |
| lmo1295 | LMRG_00745 | RNA-binding protein Hfq | -1.13 | 1 |
| lmo1723 | LMRG_02548 | hypothetical protein | -1.12 | 1 |
| lmo2532 | LMRG_01716 | ATP synthase delta chain (EC 3.6.3.14) | -1.12 | 1 |
| lmo0824 | LMRG_02249 | hypothetical protein | -1.11 | 1 |
| lmo1394 | LMRG_00846 | Oxidoreductase, short-chain dehydrogenase/reductase family | -1.11 | 1 |
| lmo0218 | LMRG_02640 | RNA binding protein, contains ribosomal protein S1 domain | -1.09 | 1 |
| lmo2792 | LMRG_01905 | DNA-binding protein | -1.09 | 1 |
| lmo0291 | LMRG_02580 | Zn-dependent hydrolase YycJ/WalJ, required for cell wall metabolism and coordination of cell division with DNA replication | -1.08 | 1 |
| lmo1047 | LMRG_00508 | GTP 3',8-cyclase (EC 4.1.99.22) | -1.08 | 1 |
| lmo2689 | LMRG_02235 | Mg(2+) transport ATPase, P-type (EC 3.6.3.2) | -1.08 | 1 |
| lmo1360 | LMRG_00810 | Methenyltetrahydrofolate cyclohydrolase (EC 3.5.4.9) / Methylenetetrahydrofolate dehydrogenase (NADP+) (EC 1.5.1.5) | -1.07 | 1 |
| lmo1814 | LMRG_00961 | Dihydroxyacetone kinase-like protein, phosphatase domain / Dihydroxyacetone kinase-like protein, kinase domain | -1.07 | 1 |
| lmo2137 | LMRG_02803 | PTS system, IIA component | -1.07 | 1 |
| lmo1179 | LMRG_00625 | Acetaldehyde dehydrogenase (EC 1.2.1.10) @ Acetaldehyde dehydrogenase (EC 1.2.1.10), ethanolamine utilization cluster | -1.06 | 1 |
| lmo1613 | LMRG_01353 | hypothetical protein | -1.05 | 1 |
| lmo2593 | LMRG_02133 | Transcriptional regulator, MerR family | -1.05 | 1 |
| lmo0397 | LMRG_00090 | hypothetical protein | -1.04 | 1 |
| lmo0932 | LMRG_02031 | Uncharacterized membrane protein YhjE | -1.04 | 1 |
| lmo1135 | LMRG_00577 | hypothetical protein | -1.04 | 1 |
| lmo1800 | LMRG_00947 | Protein tyrosine phosphatase (EC 3.1.3.48) | -1.04 | 1 |
| lmo2205 | LMRG_01627 | Phosphoglycerate mutase (EC 5.4.2.11) | -1.04 | 1 |
| lmo0483 | LMRG_00164 | Transcriptional regulator, AraC family | -1.03 | 1 |
| lmo0704 | LMRG_00393 | hypothetical protein | -1.03 | 1 |
| lmo1297 | LMRG_02856 | Cystathionine beta-lyase family protein involved in aluminum resistance | -1.03 | 1 |
| lmo1703 | LMRG_02777 | Uncharacterized RNA methyltransferase YfjO | -1.03 | 1 |
| lmo1831 | LMRG_00978 | Orotate phosphoribosyltransferase (EC 2.4.2.10) | -1.03 | 1 |
| lmo1866 | LMRG_01013 | Phosphoenolpyruvate synthase regulatory protein | -1.03 | 1 |
| lmo0367 | LMRG_00059 | Ferrous iron transport peroxidase EfeB | -1.02 | 1 |
| lmo0943 | LMRG_02041 | DNA protection during starvation protein | -1.02 | 1 |
| lmo1593 | LMRG_01374 | Cysteine desulfurase (EC 2.8.1.7), associated with tRNA 4-thiouridine synthase | -1.02 | 1 |
| lmo1736 | LMRG_02535 | Acetyltransferase | -1.02 | 1 |
| lmo0241 | LMRG_02665 | 23S rRNA (guanosine(2251)-2'-O)-methyltransferase (EC 2.1.1.185) | -1.01 | 1 |
| lmo0347 | LMRG_00038 | Phosphoenolpyruvate-dihydroxyacetone phosphotransferase (EC 2.7.1.121), ADP-binding subunit DhaL | -1.01 | 1 |
| lmo0644 | LMRG_00331 | Lipoteichoic acid primase LtaP | -1.01 | 1 |
| lmo1067 | LMRG_00529 | GTP-binding protein TypA/BipA | -1.01 | 1 |
| lmo1935 | LMRG_01082 | Protein tyrosine phosphatase (EC 3.1.3.48) | -1.01 | 1 |
| lmo2236 | LMRG_01596 | Shikimate/quinate 5-dehydrogenase I beta (EC 1.1.1.282) | -1.01 | 1 |
| lmo2529 | LMRG_01719 | ATP synthase beta chain (EC 3.6.3.14) | -1.01 | 1 |
| lmo0590 | LMRG_00272 | Dihydroxyacetone kinase-like protein, phosphatase domain / Dihydroxyacetone kinase-like protein, kinase domain | -1.01 | 1 |
| lmo0190 | LMRG_02755 | 4-diphosphocytidyl-2-C-methyl-D-erythritol kinase (EC 2.7.1.148) | -1 | 1 |
| lmo1707 | LMRG_02563 | Hypothetical protein YfkK | -1 | 1 |
| lmo1759 | LMRG_02512 | ATP-dependent DNA helicase UvrD/PcrA (EC 3.6.4.12) | -1 | 1 |
| lmo2475 | LMRG_01773 | Phosphoglucomutase (EC 5.4.2.2) | -1 | 1 |
| lmo0207 | LMRG_02629 | putative lipoprotein | -0.99 | 1 |
| lmo1013 | LMRG_02113 | Potassium efflux system KefA protein / Small-conductance mechanosensitive channel | -0.99 | 1 |
| lmo1584 | LMRG_01383 | BH3197 unknown conserved protein | -0.99 | 1 |
| lmo2354 | LMRG_01489 | Exoenzymes regulatory protein AepA precursor | -0.99 | 1 |
| lmo2580 | LMRG_02687 | Heme efflux system ATPase HrtA | -0.99 | 1 |
| lmo2594 | LMRG_02134 | hypothetical protein | -0.99 | 1 |
| lmo2723 | LMRG_01973 | Acetyltransferase, GNAT family | -0.99 | 1 |
| lmo2272 | #N/A | hypothetical protein | -0.99 | 1 |
| lmo0013 | LMRG_02442 | Cytochrome aa3-600 menaquinol oxidase subunit II | -0.98 | 1 |
| lmo0028 | LMRG_02457 | Muramoyltetrapeptide carboxypeptidase (EC 3.4.17.13) | -0.98 | 1 |
| lmo0424 | LMRG_00116 | Sugar:proton symporter | -0.98 | 1 |
| lmo0501 | LMRG_00182 | Predicted galactitol operon regulator (Transcriptional antiterminator), BglG family / PTS system, IIA component | -0.98 | 1 |
| lmo2696 | LMRG_02001 | Phosphoenolpyruvate-dihydroxyacetone phosphotransferase (EC 2.7.1.121), ADP-binding subunit DhaL | -0.98 | 1 |
| lmo2766 | LMRG_01929 | Transcriptional regulator, RpiR family | -0.98 | 1 |
| lmo0669 | LMRG_00357 | Oxidoreductase, short-chain dehydrogenase/reductase family | -0.98 | 1 |
| lmo0543 | LMRG_00225 | PTS system, glucitol/sorbitol-specific IIB component (EC 2.7.1.198) / PTS system, glucitol/sorbitol-specific IIC component 2 | -0.97 | 1 |
| lmo0733 | LMRG_00421 | Transcriptional regulator, Xre family | -0.97 | 1 |
| lmo2177 | LMRG_01655 | Membrane protein | -0.96 | 1 |
| lmo0567 | LMRG_00249 | Histidinol dehydrogenase (EC 1.1.1.23) | -0.95 | 1 |
| lmo2787 | LMRG_01910 | PTS system, beta-glucoside-specific IIB component / PTS system, beta-glucoside-specific IIC component / PTS system, beta-glucoside-specific IIA component | -0.95 | 1 |
| #N/A | LMRG_02593 | Lin0308 protein | -0.95 | 1 |
| lmo0437 | LMRG_00129 | Oxidoreductase | -0.94 | 1 |
| lmo0685 | LMRG_00373 | Flagellar motor rotation protein MotA | -0.94 | 1 |
| lmo1318 | LMRG_00768 | Intramembrane protease RasP/YluC, implicated in cell division based on FtsL cleavage | -0.94 | 1 |
| lmo1577 | LMRG_01390 | FIG002379: metal-dependent hydrolase | -0.94 | 1 |
| lmo1908 | LMRG_01055 | MazG nucleotide pyrophosphohydrolase | -0.94 | 1 |
| lmo1185 | LMRG_00631 | Ethanolamine utilization protein similar to PduT | -0.94 | 1 |
| lmo0387 | LMRG_00080 | hypothetical protein | -0.93 | 1 |
| lmo2404 | LMRG_01844 | UPF0721 transmembrane protein YunE | -0.93 | 1 |
| lmo2446 | LMRG_01802 | glycosyl hydrolase, family 31 | -0.93 | 1 |
| lmo2496 | LMRG_01752 | Phosphate ABC transporter, ATP-binding protein PstB (TC 3.A.1.7.1) | -0.93 | 1 |
| lmo2555 | LMRG_01692 | Glycosyltransferase LafA, responsible for the formation of Glc-DAG | -0.93 | 1 |
| lmos46 | #N/A | hypothetical protein | -0.93 | 1 |
| lmo2215 | LMRG_01617 | ABC transporter, ATP-binding protein EcsA | -0.92 | 1 |
| lmo2758 | LMRG_01938 | Inosine-5'-monophosphate dehydrogenase (EC 1.1.1.205) / CBS domain | -0.92 | 1 |
| lmot13 | LMRG_05013 | tRNA-Ser-GCT | -0.92 | 1 |
| lmo1538 | LMRG_01432 | Glycerol kinase (EC 2.7.1.30) | -0.91 | 1 |
| lmo1631 | LMRG_01335 | Anthranilate phosphoribosyltransferase (EC 2.4.2.18) | -0.91 | 1 |
| lmo1945 | LMRG_01092 | Substrate-specific component RibU of riboflavin ECF transporter | -0.91 | 1 |
| lmo2057 | LMRG_01207 | Heme O synthase, protoheme IX farnesyltransferase, COX10-CtaB | -0.91 | 1 |
| lmo2195 | LMRG_01637 | Oligopeptide ABC transporter, permease protein OppB (TC 3.A.1.5.1) | -0.91 | 1 |
| lmo2251 | LMRG_01580 | Glutamine ABC transporter, ATP-binding protein GlnQ | -0.91 | 1 |
| lmo0882 | LMRG_02306 | transmembrane protein, distant homology with ydbS | -0.9 | 1 |
| lmo1305 | LMRG_00755 | Transketolase (EC 2.2.1.1) | -0.9 | 1 |
| lmo1609 | LMRG_01357 | FIG009688: Thioredoxin | -0.9 | 1 |
| lmo2752 | LMRG_01944 | Heterodimeric efflux ABC transporter, multidrug resistance => LmrD subunit of LmrCD | -0.9 | 1 |
| lmo2827 | LMRG_01871 | Transcriptional regulator, MarR family | -0.9 | 1 |
| lmo0481 | LMRG_00162 | Oleate hydratase (EC 4.2.1.53) | -0.89 | 1 |
| lmo0763 | LMRG_00451 | Ser/Thr protein phosphatase family protein | -0.89 | 1 |
| lmo1611 | LMRG_01355 | Glutamyl aminopeptidase (EC 3.4.11.7) | -0.89 | 1 |
| lmo1896 | LMRG_01043 | Asparaginyl-tRNA synthetase (EC 6.1.1.22) | -0.89 | 1 |
| lmo0076 | LMRG_02327 | Methylphosphotriester-DNA--protein-cysteine S-methyltransferase (EC 2.1.1.n11) / ADA regulatory protein / Methylated-DNA--protein-cysteine methyltransferase (EC 2.1.1.63) | -0.88 | 1 |
| lmo0156 | LMRG_02401 | cyclic nucleotide-binding protein | -0.88 | 1 |
| lmo0177 | LMRG_02742 | Methionyl-tRNA synthetase (EC 6.1.1.10) | -0.88 | 1 |
| lmo0296 | LMRG_02572 | Membrane protein | -0.88 | 1 |
| lmo1919 | LMRG_01066 | Putative membrane protease YugP | -0.88 | 1 |
| lmo2695 | LMRG_02002 | Phosphoenolpyruvate-dihydroxyacetone phosphotransferase (EC 2.7.1.121), dihydroxyacetone binding subunit DhaK | -0.88 | 1 |
| lmo2579a | LMRG_02688 | internalin H | -0.88 | 1 |
| lmo0364 | LMRG_00056 | Transcriptional regulator, YafY family | -0.87 | 1 |
| lmo0637 | LMRG_00320 | SAM-dependent methyltransferase | -0.87 | 1 |
| lmo0983 | LMRG_02083 | Glutathione peroxidase (EC 1.11.1.9) @ Thioredoxin peroxidase (EC 1.11.1.15) | -0.87 | 1 |
| lmo0996 | LMRG_02096 | Methylated-DNA--protein-cysteine methyltransferase (EC 2.1.1.63) | -0.87 | 1 |
| lmo1725 | LMRG_02546 | Transcriptional regulator, GntR family | -0.87 | 1 |
| lmo1242 | LMRG_00688 | DUF1801 domain-containing protein | -0.86 | 1 |
| lmo1867 | LMRG_01014 | Pyruvate,phosphate dikinase (EC 2.7.9.1) | -0.86 | 1 |
| lmo2706 | LMRG_01991 | hypothetical protein | -0.86 | 1 |
| lmo0902 | LMRG_02845 | Transcriptional regulator, GntR family | -0.85 | 1 |
| lmo1238 | LMRG_00684 | Ribonuclease PH (EC 2.7.7.56) | -0.85 | 1 |
| lmo1268 | LMRG_00718 | ATP-dependent Clp protease ATP-binding subunit ClpX | -0.85 | 1 |
| lmo1974 | LMRG_01121 | Transcriptional regulator, GntR family | -0.85 | 1 |
| lmo0014 | LMRG_02443 | Cytochrome aa3-600 menaquinol oxidase subunit I | -0.84 | 1 |
| lmo0016 | LMRG_02445 | Cytochrome aa3-600 menaquinol oxidase subunit IV | -0.84 | 1 |
| lmo0715 | LMRG_00404 | Flagellar assembly protein FliH | -0.84 | 1 |
| lmo0981 | LMRG_02081 | Uncharacterized MFS-type transporter | -0.84 | 1 |
| lmo0431 | LMRG_00123 | Acetyltransferase (isoleucine patch superfamily) | -0.83 | 1 |
| lmo0564 | LMRG_00246 | Phosphoribosylformimino-5-aminoimidazole carboxamide ribotide isomerase (EC 5.3.1.16) | -0.83 | 1 |
| lmo1856 | LMRG_01003 | Purine nucleoside phosphorylase (EC 2.4.2.1) | -0.83 | 1 |
| lmo1961 | LMRG_01108 | Thioredoxin reductase (EC 1.8.1.9) | -0.83 | 1 |
| lmo2058 | LMRG_01208 | Heme A synthase, cytochrome oxidase biogenesis protein Cox15-CtaA | -0.83 | 1 |
| lmo2793 | LMRG_01904 | hypothetical protein | -0.83 | 1 |
| lmo0383 | LMRG_00076 | Malonate-semialdehyde dehydrogenase [inositol] (EC 1.2.1.18) | -0.82 | 1 |
| lmo0702 | LMRG_00391 | hypothetical protein | -0.82 | 1 |
| lmo1619 | LMRG_01347 | D-alanine aminotransferase (EC 2.6.1.21) | -0.82 | 1 |
| lmo2673 | LMRG_02218 | Universal stress protein family | -0.82 | 1 |
| lmo1993 | LMRG_01141 | Pyrimidine-nucleoside phosphorylase (EC 2.4.2.2) | -0.82 | 1 |
| lmo0295 | LMRG_02574 | Acetyltransferase, GNAT family | -0.82 | 1 |
| lmo0574 | LMRG_00256 | 6-phospho-beta-glucosidase (EC 3.2.1.86) | -0.81 | 1 |
| lmo0962 | LMRG_02061 | LemA protein | -0.81 | 1 |
| lmo0989 | LMRG_02089 | Transcriptional regulator, MarR family | -0.81 | 1 |
| lmo2161 | LMRG_01671 | Trehalose utilization protein ThuA | -0.81 | 1 |
| lmos79 | #N/A | hypothetical protein | -0.81 | 1 |
| lmo0591 | LMRG_00273 | hypothetical protein | -0.81 | 1 |
| lmo0871 | LMRG_02295 | Transcriptional regulator, HxlR family | -0.8 | 1 |
| lmo1301 | LMRG_00751 | Acetyltransferase, GNAT family | -0.8 | 1 |
| lmo2469 | LMRG_01779 | Uncharacterized amino acid permease, GabP family | -0.8 | 1 |
| lmo0606 | LMRG_00289 | Transcriptional regulator, MarR family | -0.8 | 1 |
| lmo0456 | LMRG_00148 | Cytosine/purine/uracil/thiamine/allantoin permease family protein | -0.79 | 1 |
| lmo0703 | LMRG_00392 | UDP-N-acetylenolpyruvoylglucosamine reductase (EC 1.3.1.98) | -0.79 | 1 |
| lmo1782 | LMRG_02830 | Exodeoxyribonuclease III (EC 3.1.11.2) | -0.79 | 1 |
| lmo2489 | LMRG_01759 | Excinuclease ABC subunit B | -0.79 | 1 |
| lmo2602 | LMRG_02146 | Mg(2+)-transport-ATPase-associated protein MgtC | -0.79 | 1 |
| lmo2692 | LMRG_02239 | protein from nitrogen regulatory protein P-II (GLNB) family, ortholog YAAQ B. subtilis | -0.79 | 1 |
| lmo0106 | LMRG_02355 | Sugar kinase and transcription regulator (EC 2.7.1.-) | -0.78 | 1 |
| lmo0687 | LMRG_00375 | hypothetical protein | -0.78 | 1 |
| lmo0505 | LMRG_00186 | Ribulose-phosphate 3-epimerase (EC 5.1.3.1) | -0.77 | 1 |
| lmo2466 | LMRG_01782 | hypothetical protein | -0.77 | 1 |
| lmo2514 | LMRG_01734 | FIG005590: DegV family protein | -0.77 | 1 |
| lmo0233 | LMRG_02673 | DNA repair protein RadA | -0.76 | 1 |
| lmo0416 | LMRG_00108 | Transcriptional regulator, Xre family | -0.76 | 1 |
| lmo0677 | LMRG_00365 | Flagellar biosynthesis protein FliQ | -0.76 | 1 |
| lmo1407 | LMRG_00859 | Pyruvate formate-lyase activating enzyme (EC 1.97.1.4) | -0.76 | 1 |
| lmo2591 | LMRG_02131 | N-acetylmuramoyl-L-alanine amidase, family 4 | -0.76 | 1 |
| lmo2665 | LMRG_02210 | PTS system, galactitol-specific IIC component | -0.76 | 1 |
| lmo2801 | LMRG_01896 | N-acetylmannosamine-6-phosphate 2-epimerase (EC 5.1.3.9) | -0.76 | 1 |
| lmo0513 | LMRG_00194 | Transcriptional regulator, MarR family | -0.75 | 1 |
| lmo0636 | LMRG_00319 | Rrf2 family transcriptional regulator | -0.75 | 1 |
| lmo1271 | LMRG_00721 | Signal peptidase I (EC 3.4.21.89) | -0.75 | 1 |
| lmo1389 | LMRG_00841 | Purine nucleoside ABC transporter, ATP-binding protein | -0.75 | 1 |
| lmo1795 | LMRG_02819 | Glutamine amidotransferase, class I | -0.75 | 1 |
| lmo1845 | LMRG_00992 | xanthine/uracil permease family protein | -0.75 | 1 |
| lmo1846 | LMRG_00993 | Multi antimicrobial extrusion protein (Na(+)/drug antiporter), MATE family of MDR efflux pumps | -0.75 | 1 |
| lmo2652 | LMRG_02904 | Putative transcriptional antiterminator, BglG family / PTS system, IIA component | -0.75 | 1 |
| lmo2712 | LMRG_01984 | Gluconokinase (EC 2.7.1.12) | -0.75 | 1 |
| lmo0024 | LMRG_02453 | PTS system, mannose/fructose/sorbose family, IID component | -0.75 | 1 |
| lmo0110 | LMRG_02359 | Esterase/lipase | -0.74 | 1 |
| lmo0157 | LMRG_02402 | DinG family ATP-dependent helicase CPE1197 | -0.74 | 1 |
| lmo1607 | LMRG_01359 | Phenylalanyl-tRNA synthetase domain protein (Bsu YtpR) | -0.74 | 1 |
| lmo1724 | LMRG_02547 | Efflux ABC transporter, ATP-binding protein | -0.74 | 1 |
| lmo0391 | LMRG_00084 | hypothetical protein | -0.73 | 1 |
| lmo0661 | LMRG_00348 | carboxymuconolactone decarboxylase family protein | -0.73 | 1 |
| lmo0690 | LMRG_00378 | Flagellin protein FlaA | -0.73 | 1 |
| lmo0705 | LMRG_00394 | Flagellar hook-associated protein FlgK | -0.73 | 1 |
| lmo1449 | LMRG_00901 | Endonuclease IV (EC 3.1.21.2) | -0.73 | 1 |
| lmo2231 | LMRG_01601 | Cobalt-zinc-cadmium resistance protein | -0.73 | 1 |
| lmo0112 | LMRG_02361 | hypothetical protein | -0.72 | 1 |
| lmo0314 | LMRG_00007 | Cellobiose phosphotransferase system YdjC-like protein | -0.72 | 1 |
| lmo1328 | LMRG_00778 | tRNA pseudouridine(55) synthase (EC 5.4.99.25) | -0.72 | 1 |
| lmo1349 | LMRG_00799 | Glycine dehydrogenase [decarboxylating] (glycine cleavage system P1 protein) (EC 1.4.4.2) | -0.72 | 1 |
| lmo1617 | LMRG_02976 | drug resistance transporter, EmrB/QacA family | -0.72 | 1 |
| lmo1886 | LMRG_01033 | Thermostable carboxypeptidase 1 (EC 3.4.17.19) | -0.72 | 1 |
| lmo2722 | LMRG_01974 | multidrug efflux transporter transcriptional regulatory protein | -0.72 | 1 |
| lmo2738 | LMRG_01958 | Hemolysins and related proteins containing CBS domains | -0.72 | 1 |
| lmo2785 | LMRG_01912 | Catalase KatE (EC 1.11.1.6) | -0.72 | 1 |
| lmo0757 | LMRG_00445 | ABC transporter permease YhcI | -0.71 | 1 |
| lmo1379 | LMRG_00831 | Inner membrane protein translocase and chaperone YidC, OxaA protein | -0.71 | 1 |
| lmo1531 | LMRG_01439 | S-adenosylmethionine:tRNA ribosyltransferase-isomerase (EC 2.4.99.17) | -0.71 | 1 |
| lmo2013 | LMRG_01161 | hypothetical protein | -0.71 | 1 |
| lmo2234 | LMRG_01598 | Xylose isomerase domain protein TIM barrel | -0.71 | 1 |
| lmo0231 | LMRG_02675 | Protein-arginine kinase McsB (EC 2.7.14.1) | -0.7 | 1 |
| lmo0749 | LMRG_00437 | Transcriptional regulator, Xre family | -0.7 | 1 |
| lmo1223 | LMRG_00669 | ABC transporter, ATP-binding protein | -0.7 | 1 |
| lmo1835 | LMRG_00982 | Carbamoyl-phosphate synthase large chain (EC 6.3.5.5) | -0.7 | 1 |
| lmo1887 | LMRG_01034 | 23S rRNA (guanine(2445)-N(2))-methyltransferase (EC 2.1.1.173) | -0.7 | 1 |
| lmo1957 | LMRG_01104 | Ferrichrome transport system permease protein FhuG | -0.7 | 1 |
| lmo0580 | LMRG_00262 | Phospholipase/carboxylesterase family protein | -0.7 | 1 |
| lmo2324 | LMRG_02920 | Phage antirepressor protein | -0.69 | 1 |
| lmo2649 | LMRG_02194 | PTS system, IIC component, UlaA-type | -0.69 | 1 |
| lmo2796 | LMRG_01901 | N-acetylmannosamine kinase (EC 2.7.1.60) | -0.69 | 1 |
| lmo0696 | LMRG_00385 | Flagellar basal-body rod modification protein FlgD | -0.69 | 1 |
| lmo0094 | LMRG_02343 | hypothetical protein | -0.68 | 1 |
| lmo0227 | LMRG_02837 | tRNA-dihydrouridine synthase DusB | -0.68 | 1 |
| lmo0525 | LMRG_00206 | hypothetical protein | -0.68 | 1 |
| lmo0792 | LMRG_00480 | Hydrolase, carbon-nitrogen family | -0.68 | 1 |
| lmo1138 | LMRG_00581 | ATP-dependent Clp protease proteolytic subunit ClpP2 (EC 3.4.21.92) | -0.68 | 1 |
| lmo2104a | LMRG_01259 | hypothetical protein | -0.68 | 1 |
| lmo0298 | LMRG_02570 | PTS system, cellobiose-specific IIC component | -0.67 | 1 |
| lmo0707 | LMRG_00396 | Flagellar cap protein FliD | -0.67 | 1 |
| lmo0760 | LMRG_00448 | Carboxylesterase (EC 3.1.1.1) in BtlB locus | -0.67 | 1 |
| lmo2403 | LMRG_01845 | 5'-nucleotidase family protein in cluster with NagD-like phosphatase | -0.67 | 1 |
| lmo2659 | LMRG_02204 | Ribulose-phosphate 3-epimerase (EC 5.1.3.1) | -0.67 | 1 |
| lmo2661 | LMRG_02206 | Ribulose-phosphate 3-epimerase (EC 5.1.3.1) | -0.67 | 1 |
| lmo0201 | LMRG_02623 | Phosphatidylinositol-specific phospholipase C (EC 4.6.1.13) | -0.66 | 1 |
| lmo0551 | LMRG_00233 | Extracellular protein | -0.66 | 1 |
| lmo0691 | LMRG_00379 | Chemotaxis regulator - transmits chemoreceptor signals to flagellar motor components CheY | -0.66 | 1 |
| lmo1029 | LMRG_02129 | Hydrolase (HAD superfamily) in cluster with DUF1447 | -0.66 | 1 |
| lmo1471 | LMRG_00924 | Ribosomal protein L11 methyltransferase | -0.66 | 1 |
| lmo1992 | LMRG_01140 | Alpha-acetolactate decarboxylase (EC 4.1.1.5) | -0.66 | 1 |
| lmo2123 | LMRG_01277 | Maltodextrin ABC transporter, permease protein MdxG | -0.66 | 1 |
| lmo2222 | LMRG_02924 | DNA double-strand break repair protein Mre11 | -0.66 | 1 |
| lmo2229 | LMRG_01603 | Multimodular transpeptidase-transglycosylase (EC 2.4.1.129) (EC 3.4.-.-) | -0.66 | 1 |
| lmo0341 | LMRG_00032 | hypothetical protein | -0.65 | 1 |
| lmo0429 | LMRG_00121 | glycosyl hydrolase, family 38 | -0.65 | 1 |
| lmo2131 | LMRG_02809 | hypothetical protein | -0.65 | 1 |
| lmo2246 | LMRG_01586 | DNA alkylation repair enzyme | -0.65 | 1 |
| lmo2740 | LMRG_01956 | hypothetical protein | -0.65 | 1 |
| lmo2751 | LMRG_01945 | Heterodimeric efflux ABC transporter, multidrug resistance => LmrC subunit of LmrCD | -0.65 | 1 |
| lmo2842 | LMRG_01856 | Transcriptional regulator YcjW, LacI family, possibly involved in maltodextrin utilization pathway | -0.65 | 1 |
| #N/A | LMRG_00279 | hypothetical protein | -0.65 | 1 |
| lmo0195 | LMRG_02617 | ABC-type antimicrobial peptide transport system, permease component | -0.64 | 1 |
| lmo0667 | LMRG_00354 | Uncharacterized efflux ABC transporter, ATP-binding protein YadG | -0.64 | 1 |
| lmo0818 | LMRG_02243 | Cation-transporting ATPase, E1-E2 family | -0.64 | 1 |
| lmo1035 | LMRG_00496 | PTS system, beta-glucoside-specific IIB component / PTS system, beta-glucoside-specific IIC component / PTS system, beta-glucoside-specific IIA component | -0.64 | 1 |
| lmo1794 | LMRG_02818 | Uncharacterized protein YlqD | -0.64 | 1 |
| lmo2150 | LMRG_01682 | hypothetical protein | -0.64 | 1 |
| lmo0029 | LMRG_02458 | hypothetical protein | -0.63 | 1 |
| lmo0090 | LMRG_02339 | ATP synthase alpha chain (EC 3.6.3.14) | -0.63 | 1 |
| lmo0772 | LMRG_00460 | Transcriptional regulator, GntR family | -0.63 | 1 |
| lmo1521 | LMRG_01449 | N-acetylmuramoyl-L-alanine amidase (EC 3.5.1.28) | -0.63 | 1 |
| lmo0165 | LMRG_02410 | tRNA(1)(Val) (adenine(37)-N(6))-methyltransferase (EC 2.1.1.223) | -0.63 | 1 |
| lmo0297 | LMRG_02571 | Transcriptional antiterminator of lichenan operon, BglG family / PTS system, cellobiose-specific IIA component (EC 2.7.1.205) | -0.62 | 1 |
| lmo1487 | LMRG_00940 | Hydrolase (HAD superfamily), YqeK | -0.62 | 1 |
| lmo1523 | LMRG_01447 | Guanosine-3',5'-bis(diphosphate) 3'-pyrophosphohydrolase (EC 3.1.7.2) / GTP pyrophosphokinase (EC 2.7.6.5), (p)ppGpp synthetase II | -0.62 | 1 |
| lmo1944 | LMRG_01091 | Ferredoxin | -0.62 | 1 |
| lmo1991 | LMRG_01139 | Threonine dehydratase biosynthetic (EC 4.3.1.19) | -0.62 | 1 |
| lmo2542 | LMRG_01705 | Peptide chain release factor N(5)-glutamine methyltransferase (EC 2.1.1.297) | -0.62 | 1 |
| lmo0105 | LMRG_02354 | Chitinase (EC 3.2.1.14) | -0.61 | 1 |
| lmo0194 | LMRG_02616 | ABC-type antimicrobial peptide transport system, ATPase component | -0.61 | 1 |
| lmo0484 | LMRG_00165 | Heme-degrading monooxygenase, staphylobilin-producing (EC 1.14.99.48) | -0.61 | 1 |
| lmo0845 | LMRG_02268 | Methionine synthase II (cobalamin-independent) | -0.61 | 1 |
| lmo0947 | LMRG_02046 | Uncharacterized MFS-type transporter YycB | -0.61 | 1 |
| lmo1392 | LMRG_00844 | FIG001621: Zinc protease | -0.61 | 1 |
| lmo1570 | LMRG_01397 | Pyruvate kinase (EC 2.7.1.40) / Phosphohistidine swiveling domain | -0.61 | 1 |
| lmo1760 | LMRG_02511 | Heptaprenylglyceryl phosphate synthase (EC 2.5.1.n9) | -0.61 | 1 |
| lmo1840 | LMRG_00987 | Pyrimidine operon regulatory protein PyrR | -0.61 | 1 |
| lmo2293 | LMRG_01539 | Protein gp10 [Bacteriophage A118] | -0.61 | 1 |
| lmo2778 | LMRG_01919 | hypothetical protein | -0.61 | 1 |
| lmo1876 | LMRG_01023 | Formate--tetrahydrofolate ligase (EC 6.3.4.3) | -0.6 | 1 |
| lmo0285 | LMRG_02586 | Methionine ABC transporter substrate-binding protein | -0.6 | 1 |
| lmo0721 | LMRG_00410 | elongation factor G-binding protein, putative | -0.6 | 1 |
| lmo1368 | LMRG_00818 | DNA repair protein RecN | -0.6 | 1 |
| lmo2755 | LMRG_01941 | hydrolase, CocE/NonD family | -0.6 | 1 |
| lmo0098 | LMRG_02347 | PTS system, mannose-specific IID component | -0.59 | 1 |
| lmo0134 | LMRG_02383 | Uncharacterized protein YjdJ, possible GNAT acetyltransferase | -0.59 | 1 |
| lmo0773 | LMRG_00461 | Acryloyl-CoA reductase AcuI/YhdH (EC 1.3.1.84) | -0.59 | 1 |
| lmo1170 | LMRG_00613 | L-threonine kinase in B12 biosynthesis (EC 2.7.1.177) | -0.59 | 1 |
| lmo1176 | LMRG_00622 | Ethanolamine ammonia-lyase light chain (EC 4.3.1.7) | -0.59 | 1 |
| lmo1662 | LMRG_01305 | Putative rRNA methylase YtqB | -0.59 | 1 |
| lmo2558 | LMRG_02909 | Autolysin, amidase | -0.59 | 1 |
| lmo2599 | LMRG_02143 | Transmembrane component of general energizing module of ECF transporters | -0.59 | 1 |
| lmo0420 | LMRG_00112 | Cof-like hydrolase | -0.59 | 1 |
| lmo1427 | LMRG_00879 | Choline ABC transport system, permease protein OpuBB | -0.58 | 1 |
| lmo1522 | LMRG_01448 | D-aminoacyl-tRNA deacylase (EC 3.1.1.96) | -0.58 | 1 |
| lmo1995 | LMRG_01143 | Deoxyribose-phosphate aldolase (EC 4.1.2.4) | -0.58 | 1 |
| lmo2660 | LMRG_02205 | Transketolase (EC 2.2.1.1) | -0.58 | 1 |
| lmo2536a | #N/A | hypothetical protein | -0.58 | 1 |
| lmo0097 | LMRG_02346 | PTS system, mannose-specific IIC component | -0.57 | 1 |
| lmo0835 | LMRG_02258 | Putative peptidoglycan bound protein (LPXTG motif) Lmo0835 homolog | -0.57 | 1 |
| lmo0878 | LMRG_02302 | Oxidoreductase, aldo/keto reductase family | -0.57 | 1 |
| lmo2430 | LMRG_01818 | Ferrichrome transport system permease protein FhuB | -0.57 | 1 |
| lmo0600 | LMRG_00283 | hypothetical protein | -0.56 | 1 |
| lmo0694 | LMRG_02872 | hypothetical protein | -0.56 | 1 |
| lmo1371 | LMRG_00821 | Dihydrolipoamide dehydrogenase of branched-chain alpha-keto acid dehydrogenase (EC 1.8.1.4) | -0.56 | 1 |
| lmo1844 | LMRG_00991 | Lipoprotein signal peptidase (EC 3.4.23.36) | -0.56 | 1 |
| lmo2114 | LMRG_01268 | ABC transporter-like sensor ATP-binding protein Bsel_2651 | -0.56 | 1 |
| lmo2757 | LMRG_01939 | ATP-dependent DNA helicase RecQ | -0.56 | 1 |
| lmo0053 | LMRG_02482 | LSU ribosomal protein L9p | -0.55 | 1 |
| lmo0087 | LMRG_02336 | hypothetical protein | -0.55 | 1 |
| lmo0092 | LMRG_02341 | ATP synthase beta chain (EC 3.6.3.14) | -0.55 | 1 |
| lmo0439 | LMRG_00131 | Siderophore/Surfactin synthetase related protein | -0.55 | 1 |
| lmo1789 | LMRG_02813 | flavodoxin-like fold domain protein | -0.55 | 1 |
| lmo1933 | LMRG_01080 | GTP cyclohydrolase I (EC 3.5.4.16) type 1 | -0.55 | 1 |
| lmo2427 | LMRG_01821 | Rod shape-determining protein RodA | -0.55 | 1 |
| lmo2571 | LMRG_02697 | Nicotinamidase (EC 3.5.1.19) | -0.55 | 1 |
| lmo0027 | LMRG_02456 | PTS system, beta-glucoside-specific IIB component / PTS system, beta-glucoside-specific IIC component / PTS system, beta-glucoside-specific IIA component | -0.54 | 1 |
| lmo0372 | LMRG_00064 | 6-phospho-beta-glucosidase (EC 3.2.1.86) | -0.54 | 1 |
| lmo0426 | LMRG_00118 | PTS system, IIA component | -0.54 | 1 |
| lmo0776 | LMRG_00464 | Sugar kinase and transcription regulator (EC 2.7.1.-) | -0.54 | 1 |
| lmo1663 | LMRG_01304 | Asparagine synthetase [glutamine-hydrolyzing] (EC 6.3.5.4) AsnB | -0.54 | 1 |
| lmo2410 | LMRG_01838 | hypothetical protein | -0.54 | 1 |
| lmo2761 | LMRG_01934 | 6-phospho-beta-glucosidase (EC 3.2.1.86) | -0.54 | 1 |
| lmo0030 | LMRG_02459 | Hydrolase, haloacid dehalogenase-like family | -0.53 | 1 |
| lmo0366 | LMRG_00058 | Ferrous iron transport periplasmic protein EfeO, contains peptidase-M75 domain and (frequently) cupredoxin-like domain | -0.53 | 1 |
| lmo0908 | LMRG_02008 | UPF0118 membrane protein YueF | -0.53 | 1 |
| lmo1636 | LMRG_01330 | Efflux ABC transporter, ATP-binding protein | -0.53 | 1 |
| lmo1784 | LMRG_02828 | LSU ribosomal protein L35p | -0.53 | 1 |
| lmo1915 | LMRG_01062 | Malolactic enzyme (EC 1.-.-.-) | -0.53 | 1 |
| lmo1959 | LMRG_01106 | Ferrichrome-binding periplasmic protein precursor (TC 3.A.1.14.3) | -0.53 | 1 |
| lmo2488 | LMRG_01760 | Excinuclease ABC subunit A | -0.53 | 1 |
| lmo2674 | LMRG_02219 | Ribose-5-phosphate isomerase B (EC 5.3.1.6) | -0.53 | 1 |
| lmo0176 | LMRG_02741 | Glucose:proton symporter GlcU | -0.52 | 1 |
| lmo0713 | LMRG_00402 | Flagellar M-ring protein FliF | -0.52 | 1 |
| lmo0724 | LMRG_00413 | hypothetical protein | -0.52 | 1 |
| lmo0807 | LMRG_02790 | Spermidine/putrescine import ABC transporter ATP-binding protein PotA (TC 3.A.1.11.1) | -0.52 | 1 |
| lmo2341 | LMRG_01502 | Pseudouridine kinase (EC 2.7.1.83) | -0.52 | 1 |
| lmo2372 | LMRG_02717 | ABC transporter, ATP-binding protein | -0.52 | 1 |
| lmo2554 | LMRG_01693 | Glycosyltransferase LafB, responsible for the formation of Gal-Glc-DAG | -0.52 | 1 |
| #N/A | LMRG_02591 | FIG00775102: hypothetical protein | -0.52 | 1 |
| lmo0507 | LMRG_00188 | PTS system, galactitol-specific IIB component (EC 2.7.1.200) | -0.51 | 1 |
| lmo1159 | LMRG_00602 | Propanediol utilization polyhedral body protein PduJ | -0.51 | 1 |
| lmo1865 | LMRG_01012 | Transcriptional repressor CcpN, MarR family | -0.51 | 1 |
| lmo2014 | LMRG_01162 | alpha-mannosidase (EC 3.2.1.24) | -0.51 | 1 |
| lmo2064 | LMRG_01214 | Large-conductance mechanosensitive channel | -0.51 | 1 |
| lmo2214 | LMRG_01618 | ABC transporter, permease protein EscB | -0.51 | 1 |
| lmo2724 | LMRG_01972 | PhnB protein | -0.51 | 1 |
| lmo2815 | LMRG_01883 | Oxidoreductase, short-chain dehydrogenase/reductase family | -0.51 | 1 |
| lmo0124 | LMRG_02373 | hypothetical protein | -0.5 | 1 |
| lmo0445 | LMRG_00137 | hypothetical protein | -0.5 | 1 |
| lmo0646 | LMRG_00333 | Glyoxalase family protein | -0.5 | 1 |
| lmo0813 | LMRG_02784 | Fructokinase (EC 2.7.1.4) | -0.5 | 1 |
| lmo1256 | LMRG_00705 | hypothetical protein | -0.5 | 1 |
| lmo1303 | LMRG_00753 | Cell division suppressor protein YneA | -0.5 | 1 |
| lmo1510 | LMRG_01460 | TPR domain protein | -0.5 | 1 |
| lmo2759 | LMRG_01937 | O-acetyl-ADP-ribose deacetylase | -0.5 | 1 |
| lmo2204 | LMRG_01628 | hypothetical protein | -0.5 | 1 |
| lmo0051 | LMRG_02480 | Accessory gene regulator protein A | -0.5 | 1 |
| lmo0929 | LMRG_02028 | Sortase A, LPXTG specific | -0.49 | 1 |
| lmo1592 | LMRG_01375 | tRNA 4-thiouridine synthase (EC 2.8.1.4) | -0.49 | 1 |
| lmo0121 | LMRG_02370 | Phage tail length tape-measure protein T | -0.48 | 1 |
| lmo0234 | LMRG_02672 | Membrane-associated protein containing RNA-binding TRAM domain and ribonuclease PIN-domain, YacL B.subtilis ortholog | -0.48 | 1 |
| lmo0279 | LMRG_02596 | Ribonucleotide reductase of class III (anaerobic), large subunit (EC 1.17.4.2) | -0.48 | 1 |
| lmo0284 | LMRG_02587 | Methionine ABC transporter ATP-binding protein | -0.48 | 1 |
| lmo0369 | LMRG_00061 | Transcriptional regulatory protein YeeN | -0.48 | 1 |
| lmo0454 | LMRG_00146 | FIG022979: MoxR-like ATPases | -0.48 | 1 |
| lmo0517 | LMRG_00198 | Phosphoglycerate mutase family protein | -0.48 | 1 |
| lmo0788 | LMRG_00476 | Activator of (R)-2-hydroxyglutaryl-CoA dehydratase | -0.48 | 1 |
| lmo2006 | LMRG_01154 | Acetolactate synthase, catabolic (EC 2.2.1.6) | -0.48 | 1 |
| lmo2174 | LMRG_01658 | GGDEF domain protein | -0.48 | 1 |
| lmo2188 | LMRG_01644 | Oligoendopeptidase F | -0.48 | 1 |
| lmo2266 | LMRG_01565 | Fumarylacetoacetate hydrolase family protein | -0.48 | 1 |
| lmo2298 | LMRG_01534 | Minor capsid protein [Bacteriophage A118] / Phage minor capsid protein | -0.48 | 1 |
| lmo0522 | LMRG_00203 | Transcriptional regulator, RpiR family | -0.47 | 1 |
| lmo0755 | LMRG_00443 | Lipase/Acylhydrolase with GDSL-like motif in BtlB locus | -0.47 | 1 |
| lmo0890 | LMRG_02314 | RsbS, negative regulator of sigma-B | -0.47 | 1 |
| lmo1082 | LMRG_00544 | dTDP-4-dehydrorhamnose 3,5-epimerase (EC 5.1.3.13) | -0.47 | 1 |
| lmo1997 | LMRG_01145 | PTS system, mannose-specific IIA component (EC 2.7.1.191) | -0.47 | 1 |
| lmo2783 | LMRG_01914 | PTS system, beta-glucoside-specific, IIC component | -0.47 | 1 |
| lmo0122 | LMRG_02371 | Phage tail fiber | -0.46 | 1 |
| lmo0480 | LMRG_02882 | Transcriptional regulator, AcrR family | -0.46 | 1 |
| lmo0592 | LMRG_00274 | hypothetical protein | -0.46 | 1 |
| lmo1243 | LMRG_00689 | PhnB protein | -0.46 | 1 |
| lmo1276 | LMRG_00726 | Methylenetetrahydrofolate--tRNA-(uracil-5-)-methyltransferase TrmFO (EC 2.1.1.74) | -0.46 | 1 |
| lmo1826 | LMRG_00973 | DNA-directed RNA polymerase omega subunit (EC 2.7.7.6) | -0.46 | 1 |
| lmo1953 | LMRG_01100 | Purine nucleoside phosphorylase (EC 2.4.2.1) @ N-Ribosylnicotinamide phosphorylase (EC 2.4.2.1) | -0.46 | 1 |
| lmo1964 | LMRG_01111 | ABC transporter, ATP-binding protein | -0.46 | 1 |
| lmo2497 | LMRG_01751 | Phosphate ABC transporter, permease protein PstA (TC 3.A.1.7.1) | -0.46 | 1 |
| lmo2544 | LMRG_01703 | Thymidine kinase (EC 2.7.1.21) | -0.46 | 1 |
| lmo0891 | LMRG_02315 | Anti-sigma B factor RsbT | -0.46 | 1 |
| lmo0276 | LMRG_02599 | Hydrolase, haloacid dehalogenase-like family | -0.45 | 1 |
| lmo0375 | LMRG_00067 | hypothetical protein | -0.45 | 1 |
| lmo1015 | LMRG_02115 | Glycine betaine ABC transport system, permease protein OpuAB | -0.45 | 1 |
| lmo1688 | LMRG_02762 | Enoyl-[acyl-carrier-protein] reductase [NADPH] (EC 1.3.1.104), FabL | -0.45 | 1 |
| lmo2393 | LMRG_02738 | hypothetical protein | -0.45 | 1 |
| lmo2748 | LMRG_01948 | general stress protein 26 | -0.45 | 1 |
| lmo2767 | LMRG_01928 | hypothetical protein | -0.45 | 1 |
| lmo0020 | LMRG_02449 | Transcriptional regulator, GntR family | -0.44 | 1 |
| lmo0025 | LMRG_02454 | hypothetical protein | -0.44 | 1 |
| lmo0414 | LMRG_00106 | Metal transporter, ZIP family | -0.44 | 1 |
| lmo0800 | LMRG_00489 | Uncharacterized protein YqkB | -0.44 | 1 |
| lmo1400 | LMRG_00852 | Acetyltransferase, GNAT family | -0.44 | 1 |
| lmo2721 | LMRG_01975 | Uncharacterized protein YieK | -0.44 | 1 |
| lmo2781 | LMRG_01916 | beta-glucosidase (EC 3.2.1.21) | -0.44 | 1 |
| lmo0129 | LMRG_02378 | N-acetylmuramoyl-L-alanine amidase (EC 3.5.1.28) | -0.43 | 1 |
| lmo0490 | LMRG_00171 | Shikimate/quinate 5-dehydrogenase I beta (EC 1.1.1.282) | -0.43 | 1 |
| lmo0862 | LMRG_02285 | Trehalose-6-phosphate hydrolase (EC 3.2.1.93) | -0.43 | 1 |
| lmo1014 | LMRG_02114 | Glycine betaine ABC transport system, ATP-binding protein OpuAA (EC 3.6.3.32) | -0.43 | 1 |
| lmo1595 | LMRG_01372 | Free methionine-(R)-sulfoxide reductase, contains GAF domain | -0.43 | 1 |
| lmo1616 | LMRG_01350 | Putative phosphotransferase YtmP | -0.43 | 1 |
| lmo2373 | LMRG_02718 | PTS system, IIB component | -0.43 | 1 |
| lmo2508 | LMRG_01740 | UPF0750 membrane protein YvjA | -0.43 | 1 |
| lmo0342 | LMRG_00033 | Transketolase (EC 2.2.1.1) | -0.42 | 1 |
| lmo2051 | LMRG_01201 | Lon-like protease with PDZ domain | -0.42 | 1 |
| lmo2183 | LMRG_01649 | Heme transporter IsdDEF, permease component IsdF | -0.42 | 1 |
| lmo1142 | LMRG_00585 | Cob(III)alamin reductase @ Cob(II)alamin reductase | -0.42 | 1 |
| lmo0401 | LMRG_00094 | alpha-mannosidase (EC 3.2.1.24) | -0.42 | 1 |
| #N/A | LMRG_02950 | hypothetical protein | -0.42 | 1 |
| lmo1366 | LMRG_00816 | RNA binding methyltransferase FtsJ like | -0.41 | 1 |
| lmo1691 | LMRG_02765 | Deoxyuridine 5'-triphosphate nucleotidohydrolase (EC 3.6.1.23) | -0.41 | 1 |
| lmo1952 | LMRG_01099 | Diaminopimelate decarboxylase (EC 4.1.1.20) | -0.41 | 1 |
| lmo2148 | LMRG_01684 | hypothetical protein | -0.41 | 1 |
| lmo2454 | LMRG_01794 | hypothetical protein | -0.41 | 1 |
| lmo2854 | LMRG_02425 | Inner membrane protein translocase and chaperone YidC, short form OxaI-like | -0.41 | 1 |
| lmo0126 | LMRG_02375 | hypothetical protein | -0.4 | 1 |
| lmo0382 | LMRG_00075 | Transcriptional repressor of the myo-inositol catabolic operon DeoR family | -0.4 | 1 |
| lmo0494 | LMRG_00175 | lipase/acylhydrolase family protein | -0.4 | 1 |
| lmo0739 | LMRG_00427 | 6-phospho-beta-glucosidase (EC 3.2.1.86) | -0.4 | 1 |
| lmo1250 | LMRG_00696 | Uncharacterized MFS-type transporter | -0.4 | 1 |
| lmo1393 | LMRG_00845 | Uncharacterized zinc protease YmfH | -0.4 | 1 |
| lmo1986 | LMRG_01134 | Ketol-acid reductoisomerase (NADP(+)) (EC 1.1.1.86) | -0.4 | 1 |
| lmo1989 | LMRG_01137 | 3-isopropylmalate dehydratase large subunit (EC 4.2.1.33) | -0.4 | 1 |
| lmo2389 | LMRG_02734 | NADH dehydrogenase (EC 1.6.99.3) | -0.4 | 1 |
| lmo2592 | LMRG_02132 | Oxidoreductase, aldo/keto reductase family | -0.4 | 1 |
| lmo0272 | LMRG_02604 | Sugar phosphatase YidA (EC 3.1.3.23) | -0.39 | 1 |
| lmo0520 | LMRG_00201 | N-acetylglucosamine kinase bacterial type predicted (EC 2.7.1.59) homolog / Transcriptional regulator | -0.39 | 1 |
| lmo0528 | LMRG_02879 | putative secreted protein | -0.39 | 1 |
| lmo0799 | LMRG_00488 | Blue-light photoreceptor | -0.39 | 1 |
| lmo0946 | LMRG_02045 | hypothetical protein | -0.39 | 1 |
| lmo1606 | LMRG_01360 | DNA translocase FtsK | -0.39 | 1 |
| lmo2094 | LMRG_01245 | class II aldolase/adducin domain protein | -0.39 | 1 |
| lmo2586 | LMRG_02681 | Putative formate dehydrogenase YrhE | -0.39 | 1 |
| lmo0155 | LMRG_02400 | Zinc ABC transporter, permease protein AdcB | -0.39 | 1 |
| lmo0096 | LMRG_02345 | PTS system, mannose-specific IIA component (EC 2.7.1.191) / PTS system, mannose-specific IIB component (EC 2.7.1.191) | -0.38 | 1 |
| lmo0949 | LMRG_02048 | UPF0118 membrane protein YdbI | -0.38 | 1 |
| lmo1566 | LMRG_01401 | Isocitrate dehydrogenase [NADP] (EC 1.1.1.42) | -0.38 | 1 |
| lmo1671 | LMRG_01296 | Zinc ABC transporter, substrate-binding protein ZnuA | -0.38 | 1 |
| lmo1916 | LMRG_01063 | Beta-lactamase class C-like and penicillin binding proteins (PBPs) superfamily | -0.38 | 1 |
| lmo2235 | LMRG_01597 | 2,4-dienoyl-CoA reductase [NADPH] (EC 1.3.1.34) | -0.38 | 1 |
| lmo2357 | LMRG_01485 | Membrane protein | -0.38 | 1 |
| lmo0257 | LMRG_02652 | RNA-2',3'-PO4:RNA-5'-OH ligase | -0.37 | 1 |
| lmo0278 | LMRG_02597 | Maltodextrin ABC transporter, ATP-binding protein MsmX | -0.37 | 1 |
| lmo0319 | LMRG_00012 | 6-phospho-beta-glucosidase (EC 3.2.1.86) | -0.37 | 1 |
| lmo0553 | LMRG_00235 | CBS domain protein | -0.37 | 1 |
| lmo1943 | LMRG_01090 | hypothetical protein YpbB | -0.37 | 1 |
| lmo2429 | LMRG_01819 | Ferrichrome transport ATP-binding protein FhuC | -0.37 | 1 |
| lmo2513 | LMRG_01735 | ComF operon protein A, DNA transporter ATPase | -0.37 | 1 |
| lmo0716 | LMRG_00405 | Flagellum-specific ATP synthase FliI | -0.36 | 1 |
| lmo1713 | LMRG_02556 | MreB-like protein (Mbl protein) | -0.36 | 1 |
| lmo2119 | LMRG_01273 | Uncharacterized secreted protein associated with spyDAC | -0.36 | 1 |
| lmo2139 | LMRG_02801 | Efflux ABC transporter, ATP-binding protein | -0.36 | 1 |
| lmo2258 | LMRG_01573 | ribulose-phosphate 3-epimerase family protein | -0.36 | 1 |
| lmo2741 | LMRG_01955 | Multidrug-efflux transporter, major facilitator superfamily (MFS) | -0.36 | 1 |
| lmo1216 | LMRG_00662 | N-acetylmuramoyl-L-alanine amidase, family 4 | -0.36 | 1 |
| lmo0993 | LMRG_02093 | KtrCD potassium uptake system, integral membrane component KtrD | -0.36 | 1 |
| lmo0018 | LMRG_02447 | 6-phospho-beta-glucosidase (EC 3.2.1.86) | -0.35 | 1 |
| lmo0286 | LMRG_02585 | Glutamine-dependent 2-keto-4-methylthiobutyrate transaminase | -0.35 | 1 |
| lmo0679 | LMRG_00367 | Flagellar biosynthesis protein FlhB | -0.35 | 1 |
| lmo0725 | LMRG_00414 | putative peptidoglycan bound protein (LPXTG motif) | -0.35 | 1 |
| lmo0951 | LMRG_02050 | Cell surface hydrolase, membrane-bound | -0.35 | 1 |
| lmo1682 | LMRG_01285 | Uncharacterized MFS-type transporter | -0.35 | 1 |
| lmo1712 | LMRG_02959 | hypothetical protein | -0.35 | 1 |
| lmo2339 | LMRG_01504 | Uncharacterized protein YbbK | -0.35 | 1 |
| lmo2734 | LMRG_01962 | glycosyl hydrolase, family 38 | -0.35 | 1 |
| lmo0008 | LMRG_02436 | Cardiolipin synthase, bacterial type ClsA | -0.34 | 1 |
| lmo0123 | LMRG_02372 | Phage tail fiber | -0.34 | 1 |
| lmo0268 | LMRG_02608 | Phosphoglycerate mutase family protein | -0.34 | 1 |
| lmo0819 | LMRG_02244 | hypothetical protein | -0.34 | 1 |
| lmo0923 | LMRG_02023 | Efflux ABC transporter, ATP-binding protein | -0.34 | 1 |
| lmo1234 | LMRG_00680 | Excinuclease ABC subunit C | -0.34 | 1 |
| lmo1859 | LMRG_02979 | Peptide-methionine (R)-S-oxide reductase MsrB (EC 1.8.4.12) | -0.34 | 1 |
| lmo1942 | LMRG_01089 | ATP-dependent DNA helicase RecS (RecQ family) | -0.34 | 1 |
| lmo2340 | LMRG_01503 | Pseudouridine 5'-phosphate glycosidase (EC 4.2.1.70) | -0.34 | 1 |
| lmo2711 | LMRG_02864 | hypothetical protein | -0.34 | 1 |
| lmo1889 | LMRG_01036 | UPF0398 protein YpsA | -0.33 | 1 |
| lmo2581 | LMRG_02686 | Heme efflux system permease HrtB | -0.33 | 1 |
| lmo2838 | LMRG_01860 | Inner membrane ABC transporter permease protein YcjO | -0.33 | 1 |
| lmo0009 | LMRG_02437 | Spermidine N1-acetyltransferase (EC 2.3.1.57) | -0.32 | 1 |
| lmo0061 | LMRG_02491 | FtsK/SpoIIIE family protein, putative EssC/YukB component of Type VII secretion system | -0.32 | 1 |
| lmo0697 | LMRG_00386 | Flagellar hook protein FlgE | -0.32 | 1 |
| lmo1189 | LMRG_00635 | Transcriptional regulator, AraC family | -0.32 | 1 |
| lmo1391 | LMRG_00843 | Purine nucleoside ABC transporter, permease protein 2 | -0.32 | 1 |
| lmo1650 | LMRG_01317 | CcdC protein | -0.32 | 1 |
| lmo1780 | LMRG_02832 | Tripeptide aminopeptidase (EC 3.4.11.4) | -0.32 | 1 |
| #N/A | LMRG_05001 | hypothetical protein | -0.32 | 1 |
| lmo0184 | LMRG_02970 | Oligo-1,6-glucosidase (EC 3.2.1.10) | -0.31 | 1 |
| lmo0711 | LMRG_00400 | Flagellar basal-body rod protein FlgC | -0.31 | 1 |
| lmo1254 | LMRG_00703 | Trehalose-6-phosphate hydrolase (EC 3.2.1.93) | -0.31 | 1 |
| lmo1514 | LMRG_01456 | Replication-associated recombination protein RarA | -0.31 | 1 |
| lmo1733 | LMRG_02538 | Glutamate synthase [NADPH] small chain (EC 1.4.1.13) | -0.31 | 1 |
| lmo2025 | LMRG_01174 | Quinolinate synthetase (EC 2.5.1.72) | -0.31 | 1 |
| lmo2511 | LMRG_01737 | Ribosome hibernation promoting factor Hpf | -0.31 | 1 |
| lmo1061 | LMRG_00523 | Two-component system sensor histidine kinase | -0.31 | 1 |
| lmo0290 | LMRG_02581 | Two-component system YycFG regulatory protein YycI | -0.3 | 1 |
| lmo0588 | LMRG_00270 | Deoxyribodipyrimidine photolyase (EC 4.1.99.3) | -0.3 | 1 |
| lmo0994 | LMRG_02094 | hypothetical protein | -0.3 | 1 |
| lmo1232 | LMRG_00678 | Recombination inhibitory protein MutS2 | -0.3 | 1 |
| lmo2129 | LMRG_01283 | hypothetical protein | -0.3 | 1 |
| lmo2221 | LMRG_01611 | Plasmid replication DNA element cmp binding-factor 1 | -0.3 | 1 |
| lmo2291 | LMRG_01541 | Phage major tail shaft protein | -0.3 | 1 |
| lmo2559 | LMRG_02710 | CTP synthase (EC 6.3.4.2) | -0.3 | 1 |
| lmo2802 | LMRG_01895 | 16S rRNA (guanine(527)-N(7))-methyltransferase (EC 2.1.1.170) | -0.3 | 1 |
| lmo1588 | LMRG_01379 | N-acetylornithine aminotransferase (EC 2.6.1.11) | -0.3 | 1 |
| lmor17 | LMRG_05517 | LSU rRNA ## 23S rRNA, large subunit ribosomal RNA | -0.3 | 1 |
| lmo0052 | LMRG_02481 | Cyclic-di-AMP phosphodiesterase GdpP | -0.29 | 1 |
| lmo0610 | LMRG_00293 | Internalin-like protein (LPXTG motif) Lmo0610 homolog | -0.29 | 1 |
| lmo0689 | LMRG_00377 | Chemotaxis protein CheV (EC 2.7.3.-) | -0.29 | 1 |
| lmo0727 | LMRG_00415 | Glutamine--fructose-6-phosphate aminotransferase [isomerizing] (EC 2.6.1.16) | -0.29 | 1 |
| lmo0864 | LMRG_02287 | FIG01423360: glycoside hydrolase | -0.29 | 1 |
| lmo0909 | LMRG_02009 | Transcriptional regulator, GntR family | -0.29 | 1 |
| lmo0987 | LMRG_02087 | Efflux ABC transporter, permease protein | -0.29 | 1 |
| lmo1701 | LMRG_02775 | hypothetical protein | -0.29 | 1 |
| lmo1843 | LMRG_00990 | LSU rRNA pseudouridine(1911/1915/1917) synthase (EC 5.4.99.23) | -0.29 | 1 |
| lmo2765 | LMRG_01930 | PTS system, cellobiose-specific IIA component (EC 2.7.1.205) | -0.29 | 1 |
| lmo2691 | LMRG_02238 | N-acetylmuramoyl-L-alanine amidase family 4 (EC 3.5.1.28), needed for cell separation and autolysis | -0.29 | 1 |
| lmo0538 | LMRG_00220 | N-acyl-L-amino acid amidohydrolase (EC 3.5.1.14) | -0.28 | 1 |
| lmo0565 | LMRG_00247 | Imidazole glycerol phosphate synthase amidotransferase subunit HisH | -0.28 | 1 |
| lmo0654 | LMRG_00341 | hypothetical protein | -0.28 | 1 |
| lmo0895 | LMRG_02319 | RNA polymerase sigma factor SigB | -0.28 | 1 |
| lmo1871 | LMRG_01018 | Phosphoglucomutase | -0.28 | 1 |
| lmo2121 | LMRG_01275 | Maltose phosphorylase (EC 2.4.1.8) | -0.28 | 1 |
| lmo2125 | LMRG_01279 | Maltodextrin ABC transporter, substrate-binding protein MdxE | -0.28 | 1 |
| lmo2450 | LMRG_01798 | Carboxylesterase (EC 3.1.1.1) | -0.28 | 1 |
| lmo2464 | LMRG_01784 | Transcriptional regulator, AcrR family | -0.28 | 1 |
| lmo2694 | LMRG_02241 | Arginine decarboxylase (EC 4.1.1.19) | -0.28 | 1 |
| lmo2736 | LMRG_01960 | Glycerate kinase (EC 2.7.1.31) | -0.28 | 1 |
| lmo2756 | LMRG_01940 | DNA topoisomerase III (EC 5.99.1.2) | -0.28 | 1 |
| lmo2821 | LMRG_01877 | Internalin-like protein (LPXTG motif) Lmo2821 homolog | -0.28 | 1 |
| lmor11 | LMRG_05511 | LSU rRNA ## 23S rRNA, large subunit ribosomal RNA | -0.28 | 1 |
| lmo0288 | LMRG_02583 | Two-component sensor kinase SA14-24 | -0.27 | 1 |
| lmo0616 | LMRG_00299 | membrane-anchoring domain / Glycerophosphoryl diester phosphodiesterase (EC 3.1.4.46) | -0.27 | 1 |
| lmo1502 | LMRG_01468 | Putative pre-16S rRNA nuclease YqgF | -0.27 | 1 |
| lmo1751 | LMRG_02977 | 23S rRNA (uracil(1939)-C(5))-methyltransferase (EC 2.1.1.190) | -0.27 | 1 |
| lmo1762 | LMRG_02509 | hypothetical protein | -0.27 | 1 |
| lmo2830 | LMRG_01868 | Thioredoxin | -0.27 | 1 |
| lmo0089 | LMRG_02969 | ATP synthase delta chain (EC 3.6.3.14) | -0.27 | 1 |
| lmo0036 | LMRG_02465 | Putrescine carbamoyltransferase (EC 2.1.3.6) | -0.26 | 1 |
| lmo0432 | LMRG_00124 | Oxidoreductase, short-chain dehydrogenase/reductase family | -0.26 | 1 |
| lmo0653 | LMRG_00340 | hypothetical protein | -0.26 | 1 |
| lmo0774 | LMRG_00462 | Diacylglycerol kinase-related protein | -0.26 | 1 |
| lmo0912 | LMRG_02012 | Formate efflux transporter FocA | -0.26 | 1 |
| lmo1019 | LMRG_02119 | Uncharacterized S1 RNA binding domain protein YitL | -0.26 | 1 |
| lmo1168 | LMRG_00611 | Propionate kinase, propanediol utilization (EC 2.7.2.1) | -0.26 | 1 |
| lmo1711 | LMRG_02558 | Aminopeptidase S (Leu, Val, Phe, Tyr preference) (EC 3.4.11.24) | -0.26 | 1 |
| lmo1761 | LMRG_02510 | Sodium-dependent transporter, SNF family | -0.26 | 1 |
| lmo1894 | LMRG_01041 | Endonuclease III (EC 4.2.99.18) | -0.26 | 1 |
| lmo2421 | LMRG_01827 | Two-component system sensor histidine kinase | -0.26 | 1 |
| lmo2492 | LMRG_01756 | hypothetical protein | -0.26 | 1 |
| lmo2687 | LMRG_02233 | Similar to rod shape-determining protein RodA | -0.26 | 1 |
| lmo0033 | LMRG_02462 | glycosyl hydrolase, family 9 | -0.25 | 1 |
| lmo0187 | LMRG_02752 | Ribonuclease M5 (EC 3.1.26.8) | -0.25 | 1 |
| lmo1009 | LMRG_02109 | CBS domain-containing protein YkuL | -0.25 | 1 |
| lmo1332 | LMRG_00782 | Probable GTPase related to EngC | -0.25 | 1 |
| lmo1378 | LMRG_00830 | Two-component system sensor histidine kinase | -0.25 | 1 |
| lmo1467 | LMRG_00919 | Phosphate starvation-inducible protein PhoH, predicted ATPase | -0.25 | 1 |
| lmo1638 | LMRG_01328 | Microcin C7 self-immunity protein mccF | -0.25 | 1 |
| lmo1764 | LMRG_02507 | Phosphoribosylamine--glycine ligase (EC 6.3.4.13) | -0.25 | 1 |
| lmo0675 | LMRG_02874 | Flagellar motor switch protein FliN | -0.25 | 1 |
| lmo0305 | LMRG_00002 | Low-specificity L-threonine aldolase (EC 4.1.2.48) | -0.24 | 1 |
| lmo0444 | LMRG_00136 | hypothetical protein | -0.24 | 1 |
| lmo0678 | LMRG_00366 | Flagellar biosynthesis protein FliR | -0.24 | 1 |
| lmo0843 | LMRG_02266 | Uncharacterized membrane protein YsdA | -0.24 | 1 |
| lmo1685 | LMRG_02759 | Glutamate-1-semialdehyde 2,1-aminomutase (EC 5.4.3.8) | -0.24 | 1 |
| lmo1981 | LMRG_01129 | hypothetical protein | -0.24 | 1 |
| lmo2423 | LMRG_01825 | Cobalt-zinc-cadmium resistance protein | -0.24 | 1 |
| lmo2445 | LMRG_01803 | Internalin-like protein Lmo2445 homolog | -0.24 | 1 |
| lmo2585 | LMRG_02682 | Hypothetical protein YrhD | -0.24 | 1 |
| lmo2646 | LMRG_02191 | Protein of unknown function DUF1341 | -0.24 | 1 |
| lmor05 | LMRG_05505 | LSU rRNA ## 23S rRNA, large subunit ribosomal RNA | -0.24 | 1 |
| lmo0656 | LMRG_00343 | Terminal oxidase biogenesis protein CtaM, putative heme A, heme O chaperone | -0.23 | 1 |
| lmo0752 | LMRG_00440 | Hydrolase/acyltransferase in BltB locus | -0.23 | 1 |
| lmo1456 | LMRG_00908 | hypothetical protein | -0.23 | 1 |
| lmo2088 | LMRG_01239 | Transcriptional regulator, AcrR family | -0.23 | 1 |
| lmo2159 | LMRG_01673 | Myo-inositol 2-dehydrogenase (EC 1.1.1.18) | -0.23 | 1 |
| lmo2539 | LMRG_01708 | Serine hydroxymethyltransferase (EC 2.1.2.1) | -0.23 | 1 |
| lmo2540 | LMRG_01707 | Low molecular weight protein tyrosine phosphatase (EC 3.1.3.48) | -0.23 | 1 |
| lmo2745 | LMRG_01951 | Multidrug resistance ABC transporter permease/ATP-binding protein BmrA | -0.23 | 1 |
| lmor08 | LMRG_05508 | LSU rRNA ## 23S rRNA, large subunit ribosomal RNA | -0.23 | 1 |
| lmor14 | LMRG_05514 | LSU rRNA ## 23S rRNA, large subunit ribosomal RNA | -0.23 | 1 |
| lmo0148 | LMRG_02391 | hypothetical protein | -0.22 | 1 |
| lmo0649 | LMRG_00336 | Transcriptional regulator, GntR family | -0.22 | 1 |
| lmo0706 | LMRG_00395 | Flagellar hook-associated protein FlgL | -0.22 | 1 |
| lmo0831 | LMRG_02254 | Auxin efflux carrier family protein | -0.22 | 1 |
| lmo1127 | LMRG_00569 | hypothetical protein | -0.22 | 1 |
| lmo1542 | LMRG_01428 | LSU ribosomal protein L21p | -0.22 | 1 |
| lmo2111 | LMRG_01265 | FMN reductase [NAD(P)H] (EC 1.5.1.39) | -0.22 | 1 |
| lmo2535 | LMRG_01713 | ATP synthase F0 sector subunit a (EC 3.6.3.14) | -0.22 | 1 |
| lmo2563 | LMRG_02706 | Uncharacterized membrane zinc metalloprotease YwhC | -0.22 | 1 |
| lmor02 | LMRG_05502 | LSU rRNA ## 23S rRNA, large subunit ribosomal RNA | -0.22 | 1 |
| lmo0186 | LMRG_02751 | Uncharacterized protein YabE | -0.21 | 1 |
| lmo0700 | LMRG_00389 | Flagellar motor switch protein FliN | -0.21 | 1 |
| lmo0893 | LMRG_02317 | Anti-sigma B factor antagonist RsbV | -0.21 | 1 |
| lmo1390 | LMRG_00842 | Purine nucleoside ABC transporter, permease protein 1 | -0.21 | 1 |
| lmo2569 | LMRG_02699 | Oligopeptide ABC transporter, substrate-binding protein OppA (TC 3.A.1.5.1) | -0.21 | 1 |
| lmo2824 | LMRG_02985 | D-3-phosphoglycerate dehydrogenase (EC 1.1.1.95) | -0.21 | 1 |
| lmo2845 | LMRG_01853 | Uncharacterized MFS-type transporter | -0.21 | 1 |
| lmo0050 | LMRG_02479 | Accessory gene regulator protein C | -0.2 | 1 |
| lmo0407 | LMRG_00100 | hypothetical protein | -0.2 | 1 |
| lmo1388 | LMRG_00840 | Purine nucleoside ABC transporter, substrate-binding protein @ CD4+ T cell-stimulating antigen, lipoprotein | -0.2 | 1 |
| lmo1447 | LMRG_00899 | Zinc ABC transporter, ATP-binding protein ZnuC | -0.2 | 1 |
| lmo1516 | LMRG_01454 | Ammonium transporter | -0.2 | 1 |
| lmo1646 | LMRG_01320 | Exonuclease SbcD | -0.2 | 1 |
| lmo2371 | LMRG_02716 | ABC transporter, permease protein | -0.2 | 1 |
| lmo2330 | LMRG_01513 | hypothetical protein | -0.2 | 1 |
| lmo0113 | LMRG_02362 | Phage protein | -0.19 | 1 |
| lmo0168 | LMRG_02413 | Transition state regulatory protein AbrB | -0.19 | 1 |
| lmo0178 | LMRG_02743 | ROK family sugar kinase or transcriptional regulator | -0.19 | 1 |
| lmo0333 | LMRG_00025 | Internalin-like protein (LPXTG motif) Lmo0333 homolog | -0.19 | 1 |
| lmo0826 | LMRG_02251 | Sodium-dependent phosphate transporter | -0.19 | 1 |
| lmo0948 | LMRG_02047 | Transcriptional regulator, GntR family | -0.19 | 1 |
| lmo1060 | LMRG_00522 | Two-component transcriptional response regulator, OmpR family | -0.19 | 1 |
| lmo1855 | LMRG_01002 | D-alanyl-D-alanine carboxypeptidase (EC 3.4.16.4) | -0.19 | 1 |
| lmo2388 | LMRG_02733 | hypothetical protein | -0.19 | 1 |
| lmo2494 | LMRG_01754 | Phosphate transport system regulatory protein PhoU | -0.19 | 1 |
| lmo2636 | LMRG_02181 | FAD:protein FMN transferase (EC 2.7.1.180) @ FAD:protein FMN transferase (EC 2.7.1.180), HepST-associated | -0.19 | 1 |
| lmo2780 | LMRG_01917 | PTS system, beta-glucoside-specific, IIA component | -0.19 | 1 |
| lmo0255 | LMRG_02654 | putative lipoprotein | -0.18 | 1 |
| lmo0508 | LMRG_00189 | PTS system, galactitol-specific IIC component | -0.18 | 1 |
| lmo0537 | LMRG_00219 | N-carbamoyl-L-amino acid hydrolase (EC 3.5.1.87) | -0.18 | 1 |
| lmo0664 | LMRG_00351 | Maltose O-acetyltransferase (EC 2.3.1.79) | -0.18 | 1 |
| lmo1024 | LMRG_02124 | hypothetical protein | -0.18 | 1 |
| lmo2576 | LMRG_02692 | Putative peptidoglycan bound protein (LPXTG motif) Lmo2576 homolog | -0.18 | 1 |
| lmo0351 | LMRG_00042 | Phosphoenolpyruvate-dihydroxyacetone phosphotransferase (EC 2.7.1.121), subunit DhaM | -0.17 | 1 |
| lmo0585 | LMRG_00267 | Extracellular protein | -0.17 | 1 |
| lmo0605 | LMRG_00288 | Na+-driven multidrug efflux pump | -0.17 | 1 |
| lmo0608 | LMRG_00291 | Efflux ABC transporter, permease/ATP-binding protein EF2592 | -0.17 | 1 |
| lmo1428 | LMRG_00880 | Choline ABC transport system, ATP-binding protein OpuBA | -0.17 | 1 |
| lmo1440 | LMRG_00892 | Uncharacterized membrane protein YqgB | -0.17 | 1 |
| lmo2552 | LMRG_01695 | UDP-N-acetylglucosamine 1-carboxyvinyltransferase (EC 2.5.1.7) | -0.17 | 1 |
| lmo2572 | LMRG_02696 | Dihydrofolate reductase homolog | -0.17 | 1 |
| lmo2818 | LMRG_01880 | Uncharacterized MFS-type transporter | -0.17 | 1 |
| lmo0077 | LMRG_02328 | Pentapeptide repeat family protein | -0.16 | 1 |
| lmo0282 | LMRG_02589 | Aliphatic amidase AmiE (EC 3.5.1.4) | -0.16 | 1 |
| lmo0587 | LMRG_00269 | cell surface protein precursor | -0.16 | 1 |
| lmo1509 | LMRG_01461 | RecD-like DNA helicase YrrC | -0.16 | 1 |
| lmo1938 | LMRG_01085 | SSU ribosomal protein S1p | -0.16 | 1 |
| lmo2500 | LMRG_01748 | Phosphate regulon sensor protein PhoR (SphS) (EC 2.7.13.3) | -0.16 | 1 |
| lmo2637 | LMRG_02182 | Putative pheromone precursor lipoprotein, related to Cad | -0.16 | 1 |
| lmo2837 | LMRG_01861 | Inner membrane ABC transporter permease protein YcjP | -0.16 | 1 |
| lmo0062 | LMRG_02493 | FIG00776004: hypothetical protein | -0.16 | 1 |
| lmo0269 | LMRG_02607 | Oligopeptide ABC transporter, permease protein OppC (TC 3.A.1.5.1) | -0.15 | 1 |
| lmo0526 | LMRG_00207 | Transcriptional regulator, MerR family | -0.15 | 1 |
| lmo0601 | LMRG_00284 | hypothetical protein | -0.15 | 1 |
| lmo0722 | LMRG_00411 | Pyruvate dehydrogenase (quinone) (EC 1.2.5.1) | -0.15 | 1 |
| lmo1246 | LMRG_00692 | ATP-dependent RNA helicase YxiN | -0.15 | 1 |
| lmo1280 | LMRG_00730 | GTP-sensing transcriptional pleiotropic repressor CodY | -0.15 | 1 |
| lmo2286 | LMRG_01546 | Putative tail or base plate protein gp17 [Bacteriophage A118] | -0.15 | 1 |
| lmo0079 | LMRG_02389 | hypothetical protein | -0.14 | 1 |
| lmo0379 | LMRG_00071 | hypothetical protein | -0.14 | 1 |
| lmo1008 | LMRG_02108 | Uncharacterized protein YkuJ | -0.14 | 1 |
| lmo1625 | LMRG_01341 | Probable cell division protein YtgP | -0.14 | 1 |
| lmo1936 | LMRG_01083 | Glycerol-3-phosphate dehydrogenase [NAD(P)+] (EC 1.1.1.94) | -0.14 | 1 |
| lmo1968 | LMRG_01115 | Creatinine amidohydrolase (EC 3.5.2.10) | -0.14 | 1 |
| lmo0130 | LMRG_02379 | 5'-nucleotidase (EC 3.1.3.5) | -0.13 | 1 |
| lmo0209 | LMRG_02631 | virulence cluster protein A VclA | -0.13 | 1 |
| lmo0594 | LMRG_00276 | Homoserine O-acetyltransferase (EC 2.3.1.31) | -0.13 | 1 |
| lmo2244 | LMRG_01588 | Similar to ribosomal large subunit pseudouridine synthase D, Bacillus subtilis YhcT type | -0.13 | 1 |
| lmo2444 | LMRG_01804 | alpha-glucosidase | -0.13 | 1 |
| lmo0408 | LMRG_00101 | hypothetical protein | -0.12 | 1 |
| lmo0452 | LMRG_00144 | FIG001454: Transglutaminase-like enzymes, putative cysteine proteases | -0.12 | 1 |
| lmo0623 | LMRG_00306 | hypothetical protein | -0.12 | 1 |
| lmo0717 | LMRG_00406 | Soluble lytic murein transglycosylase (EC 4.2.2.n1) | -0.12 | 1 |
| lmo1321 | LMRG_00771 | Bacterial ribosome SSU maturation protein RimP | -0.12 | 1 |
| lmo1479 | LMRG_00932 | Translation elongation factor LepA | -0.12 | 1 |
| lmo1641 | LMRG_01325 | Aconitate hydratase (EC 4.2.1.3) | -0.12 | 1 |
| lmo1769 | LMRG_02502 | Phosphoribosylformylglycinamidine synthase, synthetase subunit (EC 6.3.5.3) | -0.12 | 1 |
| lmo2203 | LMRG_01629 | N-acetylmuramoyl-L-alanine amidase, family 4 | -0.12 | 1 |
| lmo2476 | LMRG_01772 | Aldose 1-epimerase (EC 5.1.3.3) | -0.12 | 1 |
| lmo2536 | LMRG_01712 | ATP synthase protein I | -0.12 | 1 |
| lmo2578 | LMRG_02690 | cell surface hydrolase (putative) | -0.12 | 1 |
| lmo2588 | LMRG_02679 | Uncharacterized MFS-type transporter | -0.12 | 1 |
| lmo2725 | LMRG_01971 | Multi antimicrobial extrusion protein (Na(+)/drug antiporter), MATE family of MDR efflux pumps | -0.12 | 1 |
| lmo0047 | LMRG_02476 | putative lipoprotein | -0.11 | 1 |
| lmo0240 | LMRG_02666 | Mini-ribonuclease III | -0.11 | 1 |
| lmo0412 | LMRG_00104 | hypothetical protein | -0.11 | 1 |
| lmo0428 | LMRG_00120 | PTS system, IIC component | -0.11 | 1 |
| lmo0433 | LMRG_00126 | Internalin A (LPXTG motif) | -0.11 | 1 |
| lmo0809 | LMRG_02788 | Spermidine/putrescine import ABC transporter permease protein PotC (TC 3.A.1.11.1) | -0.11 | 1 |
| lmo0821 | LMRG_02246 | hypothetical protein | -0.11 | 1 |
| lmo0942 | LMRG_02040 | HtpG-like protein | -0.11 | 1 |
| lmo1046 | LMRG_00507 | Cyclic pyranopterin monophosphate synthase (EC 4.6.1.17) | -0.11 | 1 |
| lmo1128 | LMRG_00570 | hypothetical protein | -0.11 | 1 |
| lmo2254 | LMRG_01577 | Guanine/hypoxanthine permease PbuO | -0.11 | 1 |
| lmo2363 | LMRG_01479 | Glutamate decarboxylase (EC 4.1.1.15) | -0.11 | 1 |
| lmo2823 | LMRG_01875 | FIG008208: hypothetical protein | -0.11 | 1 |
| lmo1596a | LMRG_02946 | FIG00774215: hypothetical protein | -0.11 | 1 |
| lmo0057 | LMRG_02487 | Putative secretion accessory protein EsaA/YueB @ Bacteriophage SPP1 receptor | -0.1 | 1 |
| lmo0210 | LMRG_02632 | L-lactate dehydrogenase (EC 1.1.1.27) | -0.1 | 1 |
| lmo0359 | LMRG_00050 | Fructose-bisphosphate aldolase class II (EC 4.1.2.13) homolog | -0.1 | 1 |
| lmo0668 | LMRG_00355 | Uncharacterized efflux ABC transporter, permease protein YadH | -0.1 | 1 |
| lmo0955 | LMRG_02054 | hypothetical protein | -0.1 | 1 |
| lmo0966 | LMRG_02065 | hypothetical protein | -0.1 | 1 |
| lmo1743 | LMRG_02528 | hypothetical protein | -0.1 | 1 |
| lmo2200 | LMRG_01632 | Organic hydroperoxide resistance transcriptional regulator | -0.1 | 1 |
| lmo2370 | LMRG_02715 | Cystathionine beta-lyase (EC 4.4.1.8) | -0.1 | 1 |
| lmo2439 | LMRG_01809 | hypothetical protein | -0.1 | 1 |
| lmo0118 | LMRG_02367 | hypothetical protein | -0.09 | 1 |
| lmo0443 | LMRG_00135 | Cell envelope-associated transcriptional attenuator LytR-CpsA-Psr, subfamily F1 | -0.09 | 1 |
| lmo0778 | LMRG_00466 | hypothetical protein | -0.09 | 1 |
| lmo0798 | LMRG_02868 | Lysine-specific permease | -0.09 | 1 |
| lmo0892 | LMRG_02316 | Serine phosphatase RsbU, regulator of sigma subunit | -0.09 | 1 |
| lmo1421 | LMRG_00873 | Glycine betaine ABC transport system, ATP-binding protein OpuAA (EC 3.6.3.32) | -0.09 | 1 |
| lmo2517 | LMRG_01731 | FIG009148: Acetyltransferase, GNAT family | -0.09 | 1 |
| lmo2640 | LMRG_02185 | Heptaprenyl diphosphate synthase component I (EC 2.5.1.30) | -0.09 | 1 |
| lmo2754 | LMRG_01942 | D-alanyl-D-alanine carboxypeptidase (EC 3.4.16.4) | -0.09 | 1 |
| lmo0035 | LMRG_02464 | putative glucosamine-fructose-6-phosphate aminotransferase | -0.08 | 1 |
| lmo0101 | LMRG_02350 | Transcriptional regulator, ArsR family | -0.08 | 1 |
| lmo0181 | LMRG_02746 | N-acetyl-D-glucosamine ABC transporter, substrate-binding protein | -0.08 | 1 |
| lmo0539 | LMRG_00221 | Tagatose 1,6-bisphosphate aldolase (EC 4.1.2.40) | -0.08 | 1 |
| lmo0645 | LMRG_00332 | Uncharacterized amino acid permease, GabP family | -0.08 | 1 |
| lmo0857 | LMRG_02280 | Hydrolase, alpha/beta fold family | -0.08 | 1 |
| lmo1742 | LMRG_02529 | Adenine deaminase (EC 3.5.4.2) | -0.08 | 1 |
| lmo1975 | LMRG_01122 | DNA polymerase IV (EC 2.7.7.7) | -0.08 | 1 |
| lmo2279 | LMRG_01553 | holin | -0.08 | 1 |
| lmo2360 | LMRG_01482 | hypothetical protein | -0.08 | 1 |
| lmo2374 | LMRG_02719 | Aspartokinase (EC 2.7.2.4) | -0.08 | 1 |
| lmo2382 | LMRG_02727 | Na(+) H(+) antiporter subunit E | -0.08 | 1 |
| lmo2681 | LMRG_02226 | Potassium-transporting ATPase B chain (EC 3.6.3.12) (TC 3.A.3.7.1) | -0.08 | 1 |
| lmo2720 | LMRG_01976 | Acyl-coenzyme A synthetases/AMP-(fatty) acid ligases, YtcI homolog | -0.08 | 1 |
| lmo2812 | LMRG_01886 | D-alanyl-D-alanine carboxypeptidase (EC 3.4.16.4) | -0.08 | 1 |
| lmo0185 | LMRG_02750 | Uncharacterized metal-dependent hydrolase YcfH | -0.08 | 1 |
| lmo0179 | LMRG_02744 | N-acetyl-D-glucosamine ABC transporter, permease protein 1 | -0.07 | 1 |
| lmo0394 | LMRG_00087 | Peptidase Lmo0394 homolog | -0.07 | 1 |
| lmo0493 | LMRG_00174 | hydrolase, CocE/NonD family | -0.07 | 1 |
| lmo0535 | LMRG_00217 | Transcriptional regulator, LacI family | -0.07 | 1 |
| lmo1194 | LMRG_00640 | Cobalt-precorrin-5B (C1)-methyltransferase (EC 2.1.1.195) | -0.07 | 1 |
| lmo1300 | LMRG_00750 | Arsenite/antimonite:H+ antiporter ArsB | -0.07 | 1 |
| lmo1350 | LMRG_00800 | Glycine dehydrogenase [decarboxylating] (glycine cleavage system P2 protein) (EC 1.4.4.2) | -0.07 | 1 |
| lmo1384 | LMRG_00836 | Rhodanese domain protein UPF0176, Firmicutes subgroup | -0.07 | 1 |
| lmo1442 | LMRG_00894 | hypothetical protein | -0.07 | 1 |
| lmo2126 | LMRG_01280 | Neopullulanase (EC 3.2.1.135) | -0.07 | 1 |
| lmo2502 | LMRG_01746 | Cell division topological determinant MinJ | -0.07 | 1 |
| lmo2788 | LMRG_01909 | Beta-glucoside bgl operon antiterminator, BglG family | -0.07 | 1 |
| #N/A | LMRG_RS14955 | Cassette chromosome recombinase B | -0.07 | 1 |
| lmo0360a | LMRG_00052 | hypothetical protein | -0.07 | 1 |
| lmo0019 | LMRG_02448 | hypothetical protein | -0.06 | 1 |
| lmo0343 | LMRG_00034 | Transaldolase (EC 2.2.1.2) | -0.06 | 1 |
| lmo0519 | LMRG_00200 | Uncharacterized MFS-type transporter | -0.06 | 1 |
| lmo0523 | LMRG_00204 | Uncharacterized MFS-type transporter | -0.06 | 1 |
| lmo0555 | LMRG_00237 | Di-tripeptide/cation symporter DtpT | -0.06 | 1 |
| lmo0906 | LMRG_02006 | Glutathione reductase (EC 1.8.1.7) | -0.06 | 1 |
| lmo1136 | LMRG_00579 | Internalin-like protein (LPXTG motif) Lmo1136 homolog | -0.06 | 1 |
| lmo1253 | LMRG_00702 | Trehalose operon transcriptional repressor | -0.06 | 1 |
| lmo1737 | LMRG_02534 | Glycerol dehydrogenase (EC 1.1.1.6) | -0.06 | 1 |
| lmo1804 | LMRG_00951 | Chromosome partition protein smc | -0.06 | 1 |
| lmo2105 | LMRG_01258 | Ferrous iron transporter FeoB | -0.06 | 1 |
| lmo2127 | LMRG_01281 | CAAX amino terminal protease family protein | -0.06 | 1 |
| lmo2368 | LMRG_02713 | MutT/Nudix family protein | -0.06 | 1 |
| lmo2784 | LMRG_01913 | Transcription antiterminator, BglG family | -0.06 | 1 |
| lmo0262 | LMRG_02647 | Internalin G (LPXTG motif) | -0.05 | 1 |
| lmo0346 | LMRG_00037 | Triosephosphate isomerase (EC 5.3.1.1) | -0.05 | 1 |
| lmo0400 | LMRG_00093 | PTS system, IIC component | -0.05 | 1 |
| lmo0879 | LMRG_02303 | Sugar phosphate isomerases/epimerases | -0.05 | 1 |
| lmo1270 | LMRG_00720 | Signal peptidase I (EC 3.4.21.89) | -0.05 | 1 |
| lmo1468 | LMRG_00921 | Transamidase GatB domain protein | -0.05 | 1 |
| lmo2003 | LMRG_01151 | Transcriptional regulator, GntR family | -0.05 | 1 |
| lmo2099 | LMRG_01250 | Putative transcriptional antiterminator, BglG family / PTS system, IIA component | -0.05 | 1 |
| lmo2220 | LMRG_01612 | 3'->5' exoribonuclease Bsu YhaM | -0.05 | 1 |
| lmo2437 | LMRG_01811 | hypothetical protein | -0.05 | 1 |
| lmo0810 | LMRG_02787 | Spermidine/putrescine import ABC transporter substrate-binding protein PotD (TC 3.A.1.11.1) | -0.04 | 1 |
| lmo1370 | LMRG_00820 | Branched-chain acyl kinase | -0.04 | 1 |
| lmo1614 | LMRG_01352 | Quorum-quenching lactonase YtnP | -0.04 | 1 |
| lmo1697 | LMRG_02771 | cation efflux family protein | -0.04 | 1 |
| lmo1802 | LMRG_00949 | Signal recognition particle associated protein | -0.04 | 1 |
| lmo1963 | LMRG_01110 | ABC transporter, permease protein | -0.04 | 1 |
| lmo2107 | LMRG_01261 | Transcriptional repressor GlcR, DeoR family | -0.04 | 1 |
| lmo2264 | LMRG_01567 | UPF0750 membrane protein YitT | -0.04 | 1 |
| #N/A | LMRG_02848 | hypothetical protein | -0.04 | 1 |
| lmo0120 | LMRG_02369 | hypothetical protein | -0.03 | 1 |
| lmo0373 | LMRG_00065 | PTS system, cellobiose-specific IIC component | -0.03 | 1 |
| lmo0380 | LMRG_00073 | hypothetical protein | -0.03 | 1 |
| lmo0688 | LMRG_00376 | Glycosyl transferase, group 2 family | -0.03 | 1 |
| lmo0695 | LMRG_00384 | hypothetical protein | -0.03 | 1 |
| lmo0758 | LMRG_00446 | Glyoxalase in BtlB locus | -0.03 | 1 |
| lmo0904 | LMRG_02004 | hypothetical protein | -0.03 | 1 |
| lmo0918 | LMRG_02018 | PRD/PTS system IIA 2 domain protein | -0.03 | 1 |
| lmo1042 | LMRG_00503 | Molybdopterin molybdenumtransferase (EC 2.10.1.1) | -0.03 | 1 |
| lmo1812 | LMRG_00959 | L-serine dehydratase, alpha subunit (EC 4.3.1.17) | -0.03 | 1 |
| lmo1890 | LMRG_01037 | hyothetical protein | -0.03 | 1 |
| lmo2065 | LMRG_01215 | FIG007350: hypothetical protein co-occurring with bile hydrolase | -0.03 | 1 |
| lmo2092 | LMRG_01243 | Glycine betaine transporter OpuD | -0.03 | 1 |
| lmo2612 | LMRG_02156 | Protein translocase subunit SecY | -0.03 | 1 |
| lmo2733 | LMRG_01963 | PTS system, IIA component / PTS system, IIB component / PTS system, IIC component | -0.03 | 1 |
| lmo2771 | LMRG_01924 | 6-phospho-beta-glucosidase (EC 3.2.1.86) | -0.03 | 1 |
| lmo2846 | LMRG_02417 | L-rhamnose mutarotase (EC 5.1.3.32) | -0.03 | 1 |
| lmo0042 | LMRG_02471 | DedA protein | -0.02 | 1 |
| lmo0603 | LMRG_00286 | hypothetical protein | -0.02 | 1 |
| lmo1188 | LMRG_00634 | Putative major teichoic acid biosynthesis protein C | -0.02 | 1 |
| lmo1839 | LMRG_00986 | Uracil permease @ Uracil:proton symporter UraA | -0.02 | 1 |
| lmo2165 | LMRG_01667 | hypothetical protein | -0.02 | 1 |
| lmo2199 | LMRG_01633 | Organic hydroperoxide resistance protein | -0.02 | 1 |
| lmo2669 | LMRG_02214 | Uncharacterized DUF1113 membrane protein family | -0.02 | 1 |
| lmo1241 | LMRG_00687 | hypothetical protein | -0.02 | 1 |
| lmo0277 | LMRG_02598 | Oxidoreductase, Gfo/Idh/MocA family | -0.02 | 1 |
| lmo0215 | LMRG_02637 | FIG006789: Stage V sporulation protein | -0.01 | 1 |
| lmo0455 | LMRG_00147 | Regulator of polyketide synthase expression | -0.01 | 1 |
| lmo0663 | LMRG_00350 | Cof-like hydrolase | -0.01 | 1 |
| lmo0820 | LMRG_02245 | Acetyltransferase (GNAT family) SAS0976 | -0.01 | 1 |
| lmo0847 | LMRG_02270 | Glutamine ABC transporter, substrate-binding protein GlnH / Glutamine ABC transporter, permease protein GlnP | -0.01 | 1 |
| lmo0852 | LMRG_02275 | Transcriptional regulator, AcrR family | -0.01 | 1 |
| lmo1357 | LMRG_00807 | Biotin carboxylase of acetyl-CoA carboxylase (EC 6.3.4.14) | -0.01 | 1 |
| lmo1367 | LMRG_00817 | Arginine pathway regulatory protein ArgR, repressor of arg regulon | -0.01 | 1 |
| lmo2216 | LMRG_01616 | Histidine triad (HIT) nucleotide-binding protein, similarity with At5g48545 and yeast YDL125C (HNT1) | -0.01 | 1 |
| lmo2565 | LMRG_02704 | dNTP triphosphohydrolase, broad substrate specificity | -0.01 | 1 |
| lmo2644 | LMRG_02189 | hypothetical protein | -0.01 | 1 |
| lmo0638 | LMRG_00322 | Glutamyl endopeptidase precursor (EC 3.4.21.19), blaSE | -0.01 | 1 |
| lmo0701 | LMRG_00390 | hypothetical protein | -0.01 | 1 |
| lmo0001 | LMRG_02429 | Chromosomal replication initiator protein DnaA | 0 | 1 |
| lmo0005 | LMRG_02433 | DNA recombination and repair protein RecF | 0 | 1 |
| lmo0010 | LMRG_02439 | Mevalonate kinase (EC 2.7.1.36) | 0 | 1 |
| lmo0021 | LMRG_02450 | PTS system, IIA component | 0 | 1 |
| lmo0022 | LMRG_02451 | PTS system, mannose/fructose/sorbose family, IIB component | 0 | 1 |
| lmo0041 | LMRG_02470 | Transcriptional regulator, RpiR family | 0 | 1 |
| lmo0044 | LMRG_02473 | SSU ribosomal protein S6p | 0 | 1 |
| lmo0045 | LMRG_02474 | Single-stranded DNA-binding protein | 0 | 1 |
| lmo0046 | LMRG_02475 | SSU ribosomal protein S18p @ SSU ribosomal protein S18p, zinc-independent | 0 | 1 |
| lmo0048 | LMRG_02477 | Accessory gene regulator protein B (EC 3.4.-.-) | 0 | 1 |
| lmo0059 | LMRG_02489 | Putative secretion accessory protein EsaB/YukD | 0 | 1 |
| lmo0088 | LMRG_02337 | ATP synthase F0 sector subunit c (EC 3.6.3.14) | 0 | 1 |
| lmo0093 | LMRG_02342 | ATP synthase epsilon chain (EC 3.6.3.14) | 0 | 1 |
| lmo0102 | LMRG_02351 | hypothetical protein | 0 | 1 |
| lmo0114 | LMRG_02363 | repressor protein | 0 | 1 |
| lmo0115 | LMRG_02364 | Listeria protein LmaD, associated with virulence | 0 | 1 |
| lmo0135 | LMRG_02384 | Oligopeptide ABC transporter, substrate-binding protein OppA (TC 3.A.1.5.1) | 0 | 1 |
| lmo0136 | LMRG_02385 | Oligopeptide ABC transporter, permease protein OppB (TC 3.A.1.5.1) | 0 | 1 |
| lmo0138 | LMRG_02387 | hypothetical protein | 0 | 1 |
| lmo0139 | LMRG_02388 | hypothetical protein | 0 | 1 |
| lmo0197 | LMRG_02619 | DNA-binding protein SpoVG | 0 | 1 |
| lmo0198 | LMRG_02620 | N-acetylglucosamine-1-phosphate uridyltransferase (EC 2.7.7.23) / Glucosamine-1-phosphate N-acetyltransferase (EC 2.3.1.157) | 0 | 1 |
| lmo0199 | LMRG_02621 | Ribose-phosphate pyrophosphokinase (EC 2.7.6.1) | 0 | 1 |
| lmo0208 | LMRG_02630 | UPF0145 protein YbjQ | 0 | 1 |
| lmo0217 | LMRG_02639 | Cell division protein DivIC (FtsB), stabilizes FtsL against RasP cleavage | 0 | 1 |
| lmo0225 | LMRG_02839 | Dihydroneopterin aldolase (EC 4.1.2.25) | 0 | 1 |
| lmo0229 | LMRG_02677 | Transcriptional regulator CtsR | 0 | 1 |
| lmo2333 | LMRG_01510 | Competence transcription factor | 0 | 1 |
| lmo0239 | LMRG_02667 | Cysteinyl-tRNA synthetase (EC 6.1.1.16) | 0 | 1 |
| lmo0245 | LMRG_02661 | Protein translocase subunit SecE | 0 | 1 |
| lmo0246 | LMRG_02660 | Transcription antitermination protein NusG | 0 | 1 |
| lmo0248 | LMRG_02658 | LSU ribosomal protein L11p (L12e) | 0 | 1 |
| lmo0249 | LMRG_02657 | LSU ribosomal protein L1p (L10Ae) | 0 | 1 |
| lmo0251 | LMRG_02655 | LSU ribosomal protein L7p/L12p (P1/P2) | 0 | 1 |
| lmo0283 | LMRG_02588 | Methionine ABC transporter permease protein | 0 | 1 |
| lmo0287 | LMRG_02584 | Two-component response regulator SA14-24 | 0 | 1 |
| lmo0289 | LMRG_02582 | Two-component system YycFG regulatory protein YycH | 0 | 1 |
| lmo0293 | LMRG_02578 | 23S rRNA (pseudouridine(1915)-N(3))-methyltransferase (EC 2.1.1.177) | 0 | 1 |
| lmo0299 | LMRG_02569 | PTS system, cellobiose-specific IIB component (EC 2.7.1.205) | 0 | 1 |
| lmo0306 | LMRG_00003 | hypothetical protein | 0 | 1 |
| lmo0308 | LMRG_00005 | hypothetical protein | 0 | 1 |
| lmo0318 | LMRG_00011 | Thiamin-phosphate pyrophosphorylase (EC 2.5.1.3) | 0 | 1 |
| lmo0335 | LMRG_00027 | hypothetical protein | 0 | 1 |
| lmo0336 | LMRG_00028 | hypothetical protein | 0 | 1 |
| lmo0345 | LMRG_00036 | Ribose-5-phosphate isomerase B (EC 5.3.1.6) | 0 | 1 |
| lmo0349 | LMRG_00040 | hypothetical protein | 0 | 1 |
| lmo0357 | LMRG_00048 | PTS system, IIA component | 0 | 1 |
| lmo0360 | LMRG_00051 | Transcriptional regulator, DeoR family | 0 | 1 |
| lmo0362 | LMRG_00054 | Twin-arginine translocation protein TatAy | 0 | 1 |
| lmo0399 | LMRG_00092 | PTS system, IIB component | 0 | 1 |
| lmo0419 | LMRG_00111 | Membrane protein | 0 | 1 |
| lmo0442 | LMRG_00134 | hypothetical protein | 0 | 1 |
| lmo0486 | LMRG_00167 | LSU ribosomal protein L32p @ LSU ribosomal protein L32p, zinc-independent | 0 | 1 |
| lmo0500 | LMRG_00181 | Transaldolase (EC 2.2.1.2) | 0 | 1 |
| lmo0504 | LMRG_00185 | hypothetical protein | 0 | 1 |
| lmo0532 | LMRG_00214 | hypothetical protein | 0 | 1 |
| lmo0533 | LMRG_00215 | ACT domain protein CAC_0478 | 0 | 1 |
| lmo0545 | LMRG_00227 | Glucitol operon activator protein | 0 | 1 |
| lmo0550 | LMRG_00232 | Putative peptidoglycan bound protein (LPXTG motif) Lmo0550 homolog | 0 | 1 |
| lmo0570 | LMRG_00252 | Histidinol-phosphatase (EC 3.1.3.15) | 0 | 1 |
| lmo0579 | LMRG_00261 | bacterial seryl-tRNA synthetase related | 0 | 1 |
| lmo0597 | LMRG_00280 | cyclic nucleotide-binding protein | 0 | 1 |
| lmo0619 | LMRG_00302 | hypothetical protein | 0 | 1 |
| lmo0655 | LMRG_00342 | Serine/threonine protein phosphatase (EC 3.1.3.16) | 0 | 1 |
| lmo0658 | LMRG_00345 | endonuclease III domain protein | 0 | 1 |
| lmo0680 | LMRG_00368 | Flagellar biosynthesis protein FlhA | 0 | 1 |
| lmo0684 | LMRG_00372 | hypothetical protein | 0 | 1 |
| lmo0698 | LMRG_00387 | Flagellar motor switch protein FliN | 0 | 1 |
| lmo0708 | LMRG_00397 | Flagellar biosynthesis protein FliS | 0 | 1 |
| lmo0709 | LMRG_00398 | hypothetical protein | 0 | 1 |
| lmo0761 | LMRG_00449 | Flavin reductase like domain protein in BltB locus | 0 | 1 |
| lmo0780 | LMRG_00468 | hypothetical protein | 0 | 1 |
| lmo0802 | LMRG_02795 | GTP pyrophosphokinase (EC 2.7.6.5) | 0 | 1 |
| lmo0812 | LMRG_02785 | GTP pyrophosphokinase (EC 2.7.6.5) | 0 | 1 |
| lmo0817 | LMRG_02242 | PhnB protein | 0 | 1 |
| lmo0825 | LMRG_02250 | Hydroxymethylglutaryl-CoA reductase (EC 1.1.1.34) | 0 | 1 |
| lmo0853 | LMRG_02276 | small multidrug resistance family (SMR) protein | 0 | 1 |
| lmo0855 | LMRG_02278 | D-alanine--D-alanine ligase (EC 6.3.2.4) | 0 | 1 |
| lmo0870 | LMRG_02294 | hypothetical protein | 0 | 1 |
| lmo0874 | LMRG_02298 | PTS system, beta-glucoside-specific, IIA component | 0 | 1 |
| lmo0885 | LMRG_02309 | Holo-[acyl-carrier-protein] synthase (EC 2.7.8.7) | 0 | 1 |
| lmo0887 | LMRG_02311 | Programmed cell death antitoxin YdcD | 0 | 1 |
| lmo0914 | LMRG_02014 | PTS system, cellobiose-specific IIB component (EC 2.7.1.205) | 0 | 1 |
| lmo0930 | LMRG_02029 | Metal-dependent hydrolases of the beta-lactamase superfamily I | 0 | 1 |
| lmo0970 | LMRG_02069 | Enoyl-[acyl-carrier-protein] reductase [NADH] (EC 1.3.1.9) | 0 | 1 |
| lmo0986 | LMRG_02086 | Efflux ABC transporter, ATP-binding protein | 0 | 1 |
| lmo1010 | LMRG_02110 | LysR-family transcriptional regulator CcpC, catabolite repressor of CitB and CitZ expression | 0 | 1 |
| lmo1012 | LMRG_02112 | N-acetyl-L,L-diaminopimelate deacetylase (EC 3.5.1.47) | 0 | 1 |
| lmo1020 | LMRG_02120 | Membrane protein LiaF(VraT), specific inhibitor of LiaRS(VraRS) signaling pathway | 0 | 1 |
| lmo1028 | LMRG_02128 | Protein of unknown function DUF1447 | 0 | 1 |
| lmo1032 | LMRG_00493 | Transketolase, N-terminal section (EC 2.2.1.1) | 0 | 1 |
| lmo1033 | LMRG_00494 | Transketolase, C-terminal section (EC 2.2.1.1) | 0 | 1 |
| lmo1038 | LMRG_00499 | Molybdenum cofactor guanylyltransferase (EC 2.7.7.77) | 0 | 1 |
| lmo1040 | LMRG_00501 | Molybdenum ABC transporter permease protein ModB | 0 | 1 |
| lmo1043 | LMRG_00504 | Molybdopterin-guanine dinucleotide biosynthesis protein MobB | 0 | 1 |
| lmo1044 | LMRG_00505 | Molybdopterin synthase catalytic subunit MoaE (EC 2.8.1.12) | 0 | 1 |
| lmo1045 | LMRG_00506 | Molybdopterin synthase sulfur carrier subunit | 0 | 1 |
| lmo1051 | LMRG_00512 | Peptide deformylase (EC 3.5.1.88) | 0 | 1 |
| lmo1074 | LMRG_00536 | Teichoic acid translocation permease protein TagG | 0 | 1 |
| lmo1078 | LMRG_00540 | UTP--glucose-1-phosphate uridylyltransferase (EC 2.7.7.9) | 0 | 1 |
| lmo1087 | LMRG_00549 | Xylitol dehydrogenase | 0 | 1 |
| lmo1089 | LMRG_00551 | Glycerol-3-phosphate cytidylyltransferase (EC 2.7.7.39) | 0 | 1 |
| lmo1091 | LMRG_00553 | Beta-1,3-glucosyltransferase | 0 | 1 |
| lmo1093 | LMRG_00555 | NAD synthetase (EC 6.3.1.5) | 0 | 1 |
| lmo1117 | LMRG_00560 | Glyoxalase family protein | 0 | 1 |
| lmo1121 | LMRG_00563 | hypothetical protein | 0 | 1 |
| lmo1130 | LMRG_00572 | hypothetical protein | 0 | 1 |
| lmo1144 | LMRG_00587 | Propanediol utilization polyhedral body protein PduU | 0 | 1 |
| lmo1145 | LMRG_00588 | Propanediol utilization protein PduV | 0 | 1 |
| lmo1147 | LMRG_00590 | Adenosylcobinamide kinase (EC 2.7.1.156) / Adenosylcobinamide-phosphate guanylyltransferase (EC 2.7.7.62) | 0 | 1 |
| lmo1149 | LMRG_00592 | Alpha-ribazole-5'-phosphate phosphatase (EC 3.1.3.73) | 0 | 1 |
| lmo1151 | LMRG_00594 | Propanediol utilization polyhedral body protein PduA | 0 | 1 |
| lmo1162 | LMRG_00605 | Propanediol utilization protein PduM | 0 | 1 |
| lmo1163 | LMRG_00606 | Propanediol utilization polyhedral body protein PduN | 0 | 1 |
| lmo1166 | LMRG_00609 | Propanol dehydrogenase [NAD+] | 0 | 1 |
| lmo1169 | LMRG_00612 | L-threonine 3-O-phosphate decarboxylase (EC 4.1.1.81) | 0 | 1 |
| lmo1174 | LMRG_00620 | Ethanolamine utilization protein EutA | 0 | 1 |
| lmo1190 | LMRG_00636 | Substrate-specific component CblT of predicted B12-regulated ECF transporter for dimethylbenzimidazole | 0 | 1 |
| lmo1200 | LMRG_00646 | Cobalt-precorrin-6A reductase (EC 1.3.1.106) | 0 | 1 |
| lmo1203 | LMRG_00649 | Cobalt-precorrin-2 C(20)-methyltransferase (EC 2.1.1.151) | 0 | 1 |
| lmo1205 | LMRG_00651 | Cobalt ECF transporter, additional substrate-binding protein CbiN | 0 | 1 |
| lmo1206 | LMRG_00652 | Cobalt ECF transporter, transmembrane component of energizing module CbiQ | 0 | 1 |
| lmo1212 | LMRG_00658 | hypothetical protein | 0 | 1 |
| lmo1222 | LMRG_00668 | Phenylalanyl-tRNA synthetase beta chain (EC 6.1.1.20) | 0 | 1 |
| lmo1229 | LMRG_00675 | Z-ring-associated protein | 0 | 1 |
| lmo1239 | LMRG_00685 | Nucleoside 5-triphosphatase RdgB (dHAPTP, dITP, XTP-specific) (EC 3.6.1.66) | 0 | 1 |
| lmo1248 | LMRG_00694 | MutT/Nudix family protein | 0 | 1 |
| lmo1249 | LMRG_00695 | hypothetical protein | 0 | 1 |
| lmo1263 | LMRG_00712 | DNA-binding protein | 0 | 1 |
| lmo1272 | LMRG_00722 | LSU ribosomal maturation GTPase RbgA (B. subtilis YlqF) | 0 | 1 |
| lmo1282 | LMRG_00732 | Uncharacterized protein YneQ | 0 | 1 |
| lmo1284 | LMRG_00734 | Acyl-phosphate:glycerol-3-phosphate O-acyltransferase PlsY (EC 2.3.1.n3) | 0 | 1 |
| lmo1286 | LMRG_00736 | DNA topoisomerase IV subunit B (EC 5.99.1.3) | 0 | 1 |
| lmo1306 | LMRG_00756 | UPF0154 protein YneF | 0 | 1 |
| lmo1309 | LMRG_00759 | Co-activator of prophage gene expression IbrB | 0 | 1 |
| lmo1313 | LMRG_00763 | Uridylate kinase (EC 2.7.4.22) | 0 | 1 |
| lmo1315 | LMRG_00765 | Undecaprenyl diphosphate synthase (EC 2.5.1.31) | 0 | 1 |
| lmo1316 | LMRG_00766 | Phosphatidate cytidylyltransferase (EC 2.7.7.41) | 0 | 1 |
| lmo1319 | LMRG_00769 | Prolyl-tRNA synthetase (EC 6.1.1.15), bacterial type | 0 | 1 |
| lmo1323 | LMRG_00773 | COG2740: Predicted nucleic-acid-binding protein implicated in transcription termination | 0 | 1 |
| lmo1330 | LMRG_00780 | SSU ribosomal protein S15p (S13e) | 0 | 1 |
| lmo1333 | LMRG_00783 | hypothetical protein | 0 | 1 |
| lmo1343 | LMRG_00793 | Late competence protein ComGE, FIG015564 | 0 | 1 |
| lmo1344 | LMRG_00794 | Late competence protein ComGD, access of DNA to ComEA, FIG012777 | 0 | 1 |
| lmo1345 | LMRG_00795 | Late competence protein ComGC, access of DNA to ComEA, FIG007487 | 0 | 1 |
| lmo1347 | LMRG_00797 | Late competence protein ComGA, access of DNA to ComEA | 0 | 1 |
| lmo1352 | LMRG_00802 | hypothetical protein | 0 | 1 |
| lmo1355 | LMRG_00805 | Translation elongation factor P | 0 | 1 |
| lmo1356 | LMRG_00806 | Biotin carboxyl carrier protein of acetyl-CoA carboxylase | 0 | 1 |
| lmo1359 | LMRG_00809 | Transcription termination protein NusB | 0 | 1 |
| lmo1362 | LMRG_00812 | Exodeoxyribonuclease VII small subunit (EC 3.1.11.6) | 0 | 1 |
| lmo1364 | LMRG_00814 | Cold shock protein of CSP family | 0 | 1 |
| lmo1383 | LMRG_00835 | Isopentenyl-diphosphate delta-isomerase, FMN-dependent (EC 5.3.3.2) | 0 | 1 |
| lmo1399 | LMRG_00851 | Ribonuclease Y | 0 | 1 |
| lmo1402 | LMRG_00854 | YMCA protein | 0 | 1 |
| lmo1414 | LMRG_00866 | 3-ketoacyl-CoA thiolase (EC 2.3.1.16) @ Acetyl-CoA acetyltransferase (EC 2.3.1.9) | 0 | 1 |
| lmo1415 | LMRG_00867 | Hydroxymethylglutaryl-CoA synthase (EC 2.3.3.10) | 0 | 1 |
| lmo1420 | LMRG_00872 | UDP-N-acetylenolpyruvoylglucosamine reductase (EC 1.3.1.98) | 0 | 1 |
| lmo1435 | LMRG_00887 | 4-hydroxy-tetrahydrodipicolinate synthase (EC 4.3.3.7) | 0 | 1 |
| lmo1436 | LMRG_00888 | Aspartokinase (EC 2.7.2.4) | 0 | 1 |
| lmo1437 | LMRG_00889 | Aspartate-semialdehyde dehydrogenase (EC 1.2.1.11) | 0 | 1 |
| lmo1438 | LMRG_00890 | Uncharacterized protein YqgF | 0 | 1 |
| lmo1441 | LMRG_00893 | (E)-4-hydroxy-3-methylbut-2-enyl-diphosphate synthase (flavodoxin) (EC 1.17.7.3) | 0 | 1 |
| lmo1448 | LMRG_00900 | Manganese-dependent inorganic pyrophosphatase (EC 3.6.1.1) | 0 | 1 |
| lmo1453 | LMRG_00905 | tRNA (adenine(22)-N(1))-methyltransferase (EC 2.1.1.217) | 0 | 1 |
| lmo1454 | LMRG_00906 | RNA polymerase sigma factor RpoD | 0 | 1 |
| lmo1455 | LMRG_00907 | DNA primase DnaG | 0 | 1 |
| lmo1463 | LMRG_00915 | Cytidine deaminase (EC 3.5.4.5) | 0 | 1 |
| lmo1465 | LMRG_00917 | Metal-dependent hydrolase YbeY, involved in rRNA and/or ribosome maturation and assembly | 0 | 1 |
| lmo1472 | LMRG_00925 | Chaperone protein DnaJ | 0 | 1 |
| lmo1474 | LMRG_00927 | Heat shock protein GrpE | 0 | 1 |
| lmo1480 | LMRG_00933 | SSU ribosomal protein S20p | 0 | 1 |
| lmo1488 | LMRG_00941 | Nicotinate-nucleotide adenylyltransferase (EC 2.7.7.18) | 0 | 1 |
| lmo1490 | LMRG_00943 | Shikimate 5-dehydrogenase I alpha (EC 1.1.1.25) | 0 | 1 |
| lmo1491 | LMRG_00944 | GTP-binding protein YqeH, required for biogenesis of 30S ribosome subunit | 0 | 1 |
| lmo1492 | LMRG_00945 | FIG001553: Hydrolase, HAD subfamily IIIA | 0 | 1 |
| lmo1496 | LMRG_01474 | Transcription elongation factor GreA | 0 | 1 |
| lmo1504 | LMRG_01466 | Alanyl-tRNA synthetase (EC 6.1.1.7) | 0 | 1 |
| lmo1513 | LMRG_01457 | Cysteine desulfurase (EC 2.8.1.7) | 0 | 1 |
| lmo1519 | LMRG_01451 | Aspartyl-tRNA synthetase (EC 6.1.1.12) | 0 | 1 |
| lmo1520 | LMRG_01450 | Histidyl-tRNA synthetase (EC 6.1.1.21) | 0 | 1 |
| lmo1532 | LMRG_01438 | Holliday junction ATP-dependent DNA helicase RuvB (EC 3.6.4.12) | 0 | 1 |
| lmo1533 | LMRG_01437 | Holliday junction ATP-dependent DNA helicase RuvA (EC 3.6.4.12) | 0 | 1 |
| lmo1540 | LMRG_01430 | LSU ribosomal protein L27p | 0 | 1 |
| lmo1541 | LMRG_01429 | FIG139598: Potential ribosomal protein | 0 | 1 |
| lmo1543 | LMRG_01427 | Ribonuclease G | 0 | 1 |
| lmo1545 | LMRG_01425 | Septum site-determining protein MinC | 0 | 1 |
| lmo1549 | LMRG_01420 | UPF0758 family protein | 0 | 1 |
| lmo1551 | LMRG_01417 | Dihydrofolate synthase (EC 6.3.2.12) @ Folylpolyglutamate synthase (EC 6.3.2.17) | 0 | 1 |
| lmo1552 | LMRG_01416 | Valyl-tRNA synthetase (EC 6.1.1.9) | 0 | 1 |
| lmo1555 | LMRG_01413 | Uroporphyrinogen-III synthase (EC 4.2.1.75) | 0 | 1 |
| lmo1556 | LMRG_01412 | Porphobilinogen deaminase (EC 2.5.1.61) | 0 | 1 |
| lmo1561 | LMRG_01406 | Helicase loader DnaB | 0 | 1 |
| lmo1563 | LMRG_01404 | Dephospho-CoA kinase (EC 2.7.1.24) | 0 | 1 |
| lmo1568 | LMRG_01399 | UPF0756 membrane protein YtwI | 0 | 1 |
| lmo1572 | LMRG_01395 | Acetyl-coenzyme A carboxyl transferase alpha chain (EC 6.4.1.2) | 0 | 1 |
| lmo1582 | LMRG_01385 | Adenine-specific methyltransferase (EC 2.1.1.72) | 0 | 1 |
| lmo1600 | LMRG_01367 | Chorismate mutase I (EC 5.4.99.5) / 2-keto-3-deoxy-D-arabino-heptulosonate-7-phosphate synthase I beta (EC 2.5.1.54) | 0 | 1 |
| lmo1602 | LMRG_01365 | UPF0478 protein YtxG | 0 | 1 |
| lmo1610 | LMRG_01356 | hypothetical protein | 0 | 1 |
| lmo1629 | LMRG_01337 | Phosphoribosylanthranilate isomerase (EC 5.3.1.24) | 0 | 1 |
| lmo1630 | LMRG_01336 | Indole-3-glycerol phosphate synthase (EC 4.1.1.48) | 0 | 1 |
| lmo1632 | LMRG_01334 | Anthranilate synthase, amidotransferase component (EC 4.1.3.27) | 0 | 1 |
| lmo1635 | LMRG_01331 | PhnB protein | 0 | 1 |
| lmo1647 | LMRG_01319 | Acyl-ACP:1-acyl-sn-glycerol-3-phosphate acyltransferase (EC 2.3.1.n4) | 0 | 1 |
| lmo1655 | LMRG_01312 | Uncharacterized membrane spanning protein, contains VanZ-like domain | 0 | 1 |
| lmo1656 | LMRG_01311 | hypothetical protein | 0 | 1 |
| lmo1657 | LMRG_01310 | Translation elongation factor Ts | 0 | 1 |
| lmo1658 | LMRG_01309 | SSU ribosomal protein S2p (SAe) | 0 | 1 |
| lmo1667 | LMRG_01300 | L-lactate dehydrogenase (EC 1.1.1.27) | 0 | 1 |
| lmo1668 | LMRG_01299 | Uncharacterized protein YtmB | 0 | 1 |
| lmo1669 | LMRG_01298 | 8-oxo-dGTPase Bsu YtkD / 8-oxo-GTPase Bsu YtkD | 0 | 1 |
| lmo1693 | LMRG_02767 | Regulatory protein RecX | 0 | 1 |
| lmo1709 | LMRG_02561 | Methionine aminopeptidase (EC 3.4.11.18) | 0 | 1 |
| lmo1719 | LMRG_02552 | PTS system, beta-glucoside-specific IIA component | 0 | 1 |
| lmo1735 | LMRG_02536 | Glutamate synthase operon transcriptional activator GltC, LysR family | 0 | 1 |
| lmo1756 | LMRG_02515 | Aspartyl-tRNA(Asn) amidotransferase subunit C (EC 6.3.5.6) @ Glutamyl-tRNA(Gln) amidotransferase subunit C (EC 6.3.5.7) | 0 | 1 |
| lmo1766 | LMRG_02505 | Phosphoribosylglycinamide formyltransferase (EC 2.1.2.2) | 0 | 1 |
| lmo1772 | LMRG_02499 | Phosphoribosylaminoimidazole-succinocarboxamide synthase (EC 6.3.2.6) | 0 | 1 |
| lmo1792 | LMRG_02816 | tRNA (guanine(37)-N(1))-methyltransferase (EC 2.1.1.228) | 0 | 1 |
| lmo1793 | LMRG_02817 | 16S rRNA processing protein RimM | 0 | 1 |
| lmo1796 | LMRG_02820 | KH domain RNA binding protein YlqC | 0 | 1 |
| lmo1797 | LMRG_02821 | SSU ribosomal protein S16p | 0 | 1 |
| lmo1801 | LMRG_00948 | Signal recognition particle protein Ffh | 0 | 1 |
| lmo1806 | LMRG_00953 | Acyl carrier protein | 0 | 1 |
| lmo1807 | LMRG_00954 | 3-oxoacyl-[acyl-carrier protein] reductase (EC 1.1.1.100), FadG | 0 | 1 |
| lmo1808 | LMRG_00955 | Malonyl CoA-acyl carrier protein transacylase (EC 2.3.1.39) | 0 | 1 |
| lmo1816 | LMRG_00963 | LSU ribosomal protein L28p @ LSU ribosomal protein L28p, zinc-independent | 0 | 1 |
| lmo1817 | LMRG_00964 | Thiamin pyrophosphokinase (EC 2.7.6.2) | 0 | 1 |
| lmo1820 | LMRG_00967 | Serine/threonine protein kinase PrkC, regulator of stationary phase | 0 | 1 |
| lmo1825 | LMRG_00972 | Phosphopantothenoylcysteine decarboxylase (EC 4.1.1.36) / Phosphopantothenoylcysteine synthetase (EC 6.3.2.5) | 0 | 1 |
| lmo1827 | LMRG_00974 | Guanylate kinase (EC 2.7.4.8) | 0 | 1 |
| lmo1834 | LMRG_00981 | Dihydroorotate dehydrogenase (NAD(+)), electron transfer subunit (EC 1.3.1.14) | 0 | 1 |
| lmo1836 | LMRG_00983 | Carbamoyl-phosphate synthase small chain (EC 6.3.5.5) | 0 | 1 |
| lmo1841 | LMRG_00988 | hypothetical protein | 0 | 1 |
| lmo1852 | LMRG_00999 | Copper(I) chaperone CopZ | 0 | 1 |
| lmo1874 | LMRG_01021 | Thymidylate synthase (EC 2.1.1.45) | 0 | 1 |
| lmo1879 | LMRG_01026 | Cold shock protein of CSP family => CspD (naming convention as in B.subtlis) | 0 | 1 |
| lmo1882 | LMRG_01029 | SSU ribosomal protein S14p (S29e) @ SSU ribosomal protein S14p (S29e), zinc-independent | 0 | 1 |
| lmo1891 | LMRG_01038 | RecU Holliday junction resolvase | 0 | 1 |
| lmo1895 | LMRG_01042 | Chromosome replication initiation protein DnaD | 0 | 1 |
| lmo1903 | LMRG_01050 | bacteriocin transport accessory protein | 0 | 1 |
| lmo1904 | LMRG_01051 | Biotin operon repressor / Biotin--protein ligase (EC 6.3.4.9)(EC 6.3.4.10)(EC 6.3.4.11)(EC 6.3.4.15) | 0 | 1 |
| lmo1905 | LMRG_01052 | CCA tRNA nucleotidyltransferase (EC 2.7.7.72) | 0 | 1 |
| lmo1907 | LMRG_01054 | 4-hydroxy-tetrahydrodipicolinate reductase (EC 1.17.1.8) | 0 | 1 |
| lmo1922 | LMRG_01069 | FIG009300: TPR-repeat-containing protein | 0 | 1 |
| lmo1934 | LMRG_01081 | DNA-binding protein HBsu | 0 | 1 |
| lmo1951 | LMRG_01098 | Segregation and condensation protein A | 0 | 1 |
| lmo1973 | LMRG_01120 | PTS system, IIA component | 0 | 1 |
| lmo1977 | LMRG_01124 | Ribonuclease Z (EC 3.1.26.11) | 0 | 1 |
| lmo1985 | LMRG_01133 | Acetolactate synthase small subunit (EC 2.2.1.6) | 0 | 1 |
| lmo2002 | LMRG_01150 | PTS system, mannose-specific IIB component (EC 2.7.1.191) | 0 | 1 |
| lmo2010 | LMRG_01158 | DNA-binding response regulator, AraC family | 0 | 1 |
| lmo2012 | LMRG_01160 | hypothetical protein | 0 | 1 |
| lmo2017 | LMRG_01166 | putative membrane-associated phospholipid phosphatase, PAP2 superfamily | 0 | 1 |
| lmo2020 | LMRG_01169 | Cell division initiation protein DivIVA | 0 | 1 |
| lmo2032 | LMRG_01181 | Cell division protein FtsZ | 0 | 1 |
| lmo2033 | LMRG_01182 | Cell division protein FtsA | 0 | 1 |
| lmo2034 | LMRG_01183 | Cell division protein FtsQ | 0 | 1 |
| lmo2035 | LMRG_01184 | UDP-N-acetylglucosamine--N-acetylmuramyl-(pentapeptide) pyrophosphoryl-undecaprenol N-acetylglucosamine transferase (EC 2.4.1.227) | 0 | 1 |
| lmo2036 | LMRG_01185 | UDP-N-acetylmuramoyl-L-alanine--D-glutamate ligase (EC 6.3.2.9) | 0 | 1 |
| lmo2040 | LMRG_01189 | Cell division protein FtsL | 0 | 1 |
| lmo2041 | LMRG_01190 | 16S rRNA (cytosine(1402)-N(4))-methyltransferase (EC 2.1.1.199) | 0 | 1 |
| lmo2052 | LMRG_01202 | Phosphopantetheine adenylyltransferase (EC 2.7.7.3) | 0 | 1 |
| lmo2053 | LMRG_01203 | 16S rRNA (guanine(966)-N(2))-methyltransferase (EC 2.1.1.171) | 0 | 1 |
| lmo2063 | LMRG_01213 | hypothetical protein | 0 | 1 |
| lmo2069 | LMRG_01219 | Heat shock protein 10 kDa family chaperone GroES | 0 | 1 |
| lmo2075 | LMRG_01226 | N(6)-L-threonylcarbamoyladenine synthase (EC 2.3.1.234) | 0 | 1 |
| lmo2077 | LMRG_01228 | tRNA threonylcarbamoyladenosine biosynthesis protein TsaB | 0 | 1 |
| lmo2083 | LMRG_01234 | hypothetical protein | 0 | 1 |
| lmo2093 | LMRG_01244 | hypothetical protein | 0 | 1 |
| lmo2097 | LMRG_01248 | PTS system, galactitol-specific IIB component (EC 2.7.1.200) | 0 | 1 |
| lmo2100 | LMRG_01251 | Transcriptional regulator of pyridoxine metabolism / Pyridoxamine phosphate aminotransferase (EC 2.6.1.54) | 0 | 1 |
| lmo2101 | LMRG_01252 | Pyridoxal 5'-phosphate synthase (glutamine hydrolyzing), synthase subunit (EC 4.3.3.6) | 0 | 1 |
| lmo2102 | LMRG_01253 | Pyridoxal 5'-phosphate synthase (glutamine hydrolyzing), glutaminase subunit (EC 4.3.3.6) | 0 | 1 |
| lmo2104 | LMRG_01257 | Ferrous iron transporter-associated protein FeoA | 0 | 1 |
| lmo2120 | LMRG_01274 | Diadenylate cyclase spyDAC | 0 | 1 |
| lmo2138 | LMRG_02802 | Transcription antiterminator, BglG family | 0 | 1 |
| lmo2152 | LMRG_01680 | Putative thioredoxin | 0 | 1 |
| lmo2153 | LMRG_01679 | Flavodoxin | 0 | 1 |
| lmo2154 | LMRG_01678 | Ribonucleotide reductase of class Ia (aerobic), beta subunit (EC 1.17.4.1) | 0 | 1 |
| lmo2187 | LMRG_01645 | hypothetical protein | 0 | 1 |
| lmo2192 | LMRG_01640 | Oligopeptide ABC transporter, ATP-binding protein OppF (TC 3.A.1.5.1) | 0 | 1 |
| lmo2198 | LMRG_01634 | Tryptophanyl-tRNA synthetase (EC 6.1.1.2) | 0 | 1 |
| lmo2202 | LMRG_01630 | 3-oxoacyl-[acyl-carrier-protein] synthase, KASIII (EC 2.3.1.180) | 0 | 1 |
| lmo2211 | LMRG_01621 | Coproporphyrin ferrochelatase (EC 4.99.1.9) | 0 | 1 |
| lmo2212 | LMRG_01620 | Uroporphyrinogen III decarboxylase (EC 4.1.1.37) | 0 | 1 |
| lmo2217 | LMRG_01615 | Hypothetical protein SAV1839 | 0 | 1 |
| lmo2239 | LMRG_01593 | Membrane protein | 0 | 1 |
| lmo2247 | LMRG_01585 | oxidoreductase of aldo/keto reductase family, subgroup 2 | 0 | 1 |
| lmo2261 | LMRG_01570 | PaaD-like protein (DUF59) involved in Fe-S cluster assembly | 0 | 1 |
| lmo2267 | LMRG_02922 | ATP-dependent helicase/nuclease AddAB, subunit A | 0 | 1 |
| lmo2281 | LMRG_01551 | gp22 | 0 | 1 |
| lmo2282 | LMRG_01550 | Putative short tail fibre [Bacteriophage A118] | 0 | 1 |
| lmo2283 | LMRG_01549 | gp20 | 0 | 1 |
| lmo2321 | LMRG_01523 | Protein gp45 [Bacteriophage A118] | 0 | 1 |
| lmo2322 | LMRG_01522 | Protein gp44 [Bacteriophage A118] | 0 | 1 |
| lmo2326 | LMRG_01518 | Protein gp41 [Bacteriophage A118] | 0 | 1 |
| lmo2327 | LMRG_01517 | hypothetical protein | 0 | 1 |
| lmo2329 | LMRG_01514 | Repressor (CI-like) [Bacteriophage A118] | 0 | 1 |
| lmo2344 | LMRG_01499 | Putative glutaredoxin YtnI | 0 | 1 |
| lmo2350 | LMRG_01493 | Uncharacterized N-acetyltransferase YtmI | 0 | 1 |
| lmo2367 | LMRG_02712 | Glucose-6-phosphate isomerase (EC 5.3.1.9) | 0 | 1 |
| lmo2377 | LMRG_02722 | Uncharacterized MFS-type transporter YuxJ | 0 | 1 |
| lmo2380 | LMRG_02725 | Na(+) H(+) antiporter subunit C | 0 | 1 |
| lmo2383 | LMRG_02728 | Na(+) H(+) antiporter subunit F | 0 | 1 |
| lmo2385 | LMRG_02730 | 1,4-dihydroxy-2-naphthoyl-CoA hydrolase (EC 3.1.2.28) in menaquinone biosynthesis | 0 | 1 |
| lmo2390 | LMRG_02735 | Thioredoxin reductase (EC 1.8.1.9) | 0 | 1 |
| lmo2391 | LMRG_02736 | oxidoreductase ylbE | 0 | 1 |
| lmo2397 | LMRG_01851 | NifU family protein | 0 | 1 |
| lmo2400 | LMRG_01848 | Acetyltransferase, GNAT family | 0 | 1 |
| lmo2413 | LMRG_01835 | Cysteine desulfurase (EC 2.8.1.7) => SufS | 0 | 1 |
| lmo2414 | LMRG_01834 | Iron-sulfur cluster assembly protein SufD | 0 | 1 |
| lmo2415 | LMRG_01833 | Iron-sulfur cluster assembly ATPase protein SufC | 0 | 1 |
| lmo2422 | LMRG_01826 | Two-component transcriptional response regulator, OmpR family | 0 | 1 |
| lmo2424 | LMRG_01824 | Thioredoxin | 0 | 1 |
| lmo2426 | LMRG_01822 | FIG138056: a glutathione-dependent thiol reductase | 0 | 1 |
| lmo2435 | LMRG_01813 | hypothetical protein | 0 | 1 |
| lmo2448 | LMRG_01800 | tmRNA-binding protein SmpB | 0 | 1 |
| lmo2455 | LMRG_01793 | Enolase (EC 4.2.1.11) | 0 | 1 |
| lmo2468 | LMRG_01780 | ATP-dependent Clp protease proteolytic subunit ClpP (EC 3.4.21.92) | 0 | 1 |
| lmo2472 | LMRG_01776 | Sporulation transcription regulator WhiA | 0 | 1 |
| lmo2505 | LMRG_01743 | D-glutamyl-L-m-Dpm peptidase P45 | 0 | 1 |
| lmo2507 | LMRG_01741 | Cell-division-associated, ABC-transporter-like signaling protein FtsE | 0 | 1 |
| lmo2524 | LMRG_01724 | 3-hydroxyacyl-[acyl-carrier-protein] dehydratase, FabZ form (EC 4.2.1.59) | 0 | 1 |
| lmo2526 | LMRG_01722 | UDP-N-acetylglucosamine 1-carboxyvinyltransferase (EC 2.5.1.7) | 0 | 1 |
| lmo2533 | LMRG_01715 | ATP synthase F0 sector subunit b (EC 3.6.3.14) | 0 | 1 |
| lmo2538 | LMRG_01709 | Uracil phosphoribosyltransferase (EC 2.4.2.9) | 0 | 1 |
| lmo2541 | LMRG_01706 | Threonylcarbamoyl-AMP synthase (EC 2.7.7.87) / SUA5 domain with internal deletion | 0 | 1 |
| lmo2543 | LMRG_01704 | Peptide chain release factor 1 | 0 | 1 |
| lmo2548 | LMRG_01699 | LSU ribosomal protein L31p @ LSU ribosomal protein L31p, zinc-independent | 0 | 1 |
| lmo2556 | LMRG_01691 | Fructose-bisphosphate aldolase class II (EC 4.1.2.13) | 0 | 1 |
| lmo2561 | LMRG_02708 | Arginyl-tRNA synthetase (EC 6.1.1.19) | 0 | 1 |
| lmo2562 | LMRG_02707 | Uncharacterized beta-barrel protein YwiB | 0 | 1 |
| lmo2584 | LMRG_02683 | Sulfur carrier protein FdhD | 0 | 1 |
| lmo2596 | LMRG_02140 | SSU ribosomal protein S9p (S16e) | 0 | 1 |
| lmo2597 | LMRG_02141 | LSU ribosomal protein L13p (L13Ae) | 0 | 1 |
| lmo2600 | LMRG_02144 | ATPase component of general energizing module of ECF transporters | 0 | 1 |
| lmo2601 | LMRG_02145 | ATPase component of general energizing module of ECF transporters | 0 | 1 |
| lmo2605 | LMRG_02149 | LSU ribosomal protein L17p | 0 | 1 |
| lmo2607 | LMRG_02151 | SSU ribosomal protein S11p (S14e) | 0 | 1 |
| lmo2608 | LMRG_02152 | SSU ribosomal protein S13p (S18e) | 0 | 1 |
| lmo2609 | LMRG_02153 | LSU ribosomal protein L36p @ LSU ribosomal protein L36p, zinc-dependent | 0 | 1 |
| lmo2610 | LMRG_02906 | Translation initiation factor 1 | 0 | 1 |
| lmo2611 | LMRG_02155 | Adenylate kinase (EC 2.7.4.3) | 0 | 1 |
| lmo2614 | LMRG_02158 | LSU ribosomal protein L30p (L7e) | 0 | 1 |
| lmo2616 | LMRG_02160 | LSU ribosomal protein L18p (L5e) | 0 | 1 |
| lmo2618 | LMRG_02162 | SSU ribosomal protein S8p (S15Ae) | 0 | 1 |
| lmo2620 | LMRG_02164 | LSU ribosomal protein L5p (L11e) | 0 | 1 |
| lmo2621 | LMRG_02165 | LSU ribosomal protein L24p (L26e) | 0 | 1 |
| lmo2622 | LMRG_02166 | LSU ribosomal protein L14p (L23e) | 0 | 1 |
| lmo2623 | LMRG_02167 | SSU ribosomal protein S17p (S11e) | 0 | 1 |
| lmo2624 | LMRG_02168 | LSU ribosomal protein L29p (L35e) | 0 | 1 |
| lmo2625 | LMRG_02169 | LSU ribosomal protein L16p (L10e) | 0 | 1 |
| lmo2626 | LMRG_02170 | SSU ribosomal protein S3p (S3e) | 0 | 1 |
| lmo2627 | LMRG_02171 | LSU ribosomal protein L22p (L17e) | 0 | 1 |
| lmo2628 | LMRG_02172 | SSU ribosomal protein S19p (S15e) | 0 | 1 |
| lmo2630 | LMRG_02174 | LSU ribosomal protein L23p (L23Ae) | 0 | 1 |
| lmo2631 | LMRG_02175 | LSU ribosomal protein L4p (L1e) | 0 | 1 |
| lmo2632 | LMRG_02176 | LSU ribosomal protein L3p (L3e) | 0 | 1 |
| lmo2633 | LMRG_02177 | SSU ribosomal protein S10p (S20e) | 0 | 1 |
| lmo2653 | LMRG_02198 | Translation elongation factor Tu | 0 | 1 |
| lmo2656 | LMRG_02201 | SSU ribosomal protein S12p (S23e) | 0 | 1 |
| lmo2663 | LMRG_02208 | Galactitol-1-phosphate 5-dehydrogenase (EC 1.1.1.251) | 0 | 1 |
| lmo2664 | LMRG_02209 | Sorbitol dehydrogenase homologue (EC:1.1.1.14) | 0 | 1 |
| lmo2666 | LMRG_02211 | PTS system, galactitol-specific IIB component (EC 2.7.1.200) | 0 | 1 |
| lmo2667 | LMRG_02212 | PTS system, galactitol-specific IIA component (EC 2.7.1.200) | 0 | 1 |
| lmo2693 | LMRG_02240 | Thymidylate kinase (EC 2.7.4.9) | 0 | 1 |
| lmo2702 | LMRG_01995 | Recombination protein RecR | 0 | 1 |
| lmo2719 | LMRG_01977 | tRNA-specific adenosine-34 deaminase (EC 3.5.4.33) | 0 | 1 |
| lmo2726 | LMRG_01970 | Transcriptional regulator, MarR family | 0 | 1 |
| lmo2747 | LMRG_01949 | Seryl-tRNA synthetase (EC 6.1.1.11) | 0 | 1 |
| lmo2762 | LMRG_01933 | PTS system, cellobiose-specific IIB component (EC 2.7.1.205) | 0 | 1 |
| lmo2789 | LMRG_01908 | Uncharacterized protein CAC3725 | 0 | 1 |
| lmo2803 | LMRG_01893 | hypothetical protein | 0 | 1 |
| lmo2810 | LMRG_01888 | tRNA-5-carboxymethylaminomethyl-2-thiouridine(34) synthesis protein MnmG | 0 | 1 |
| lmo2831 | LMRG_01867 | Beta-phosphoglucomutase (EC 5.4.2.6) | 0 | 1 |
| lmo2855 | LMRG_02426 | Ribonuclease P protein component (EC 3.1.26.5) | 0 | 1 |
| lmo2856 | LMRG_02427 | LSU ribosomal protein L34p | 0 | 1 |
| lmo2857 | LMRG_02428 | hypothetical protein | 0 | 1 |
| lmo1085 | LMRG_00547 | CDP-ribitol:poly(ribitol phosphate) ribitol phosphotransferase | 0 | 1 |
| lmo1088 | LMRG_00550 | CDP-glycerol:glycerophosphate glycerophosphotransferase | 0 | 1 |
| #N/A | LMRG_02864 | hypothetical protein | 0 | 1 |
| lmo1193 | LMRG_00639 | Cobalt-precorrin-8 methylmutase (EC 5.4.99.60) | 0 | 1 |
| lmo0012 | LMRG_02441 | Phosphomevalonate kinase (EC 2.7.4.2) | 0 | 1 |
| lmo1322 | LMRG_00772 | Transcription termination protein NusA | 0 | 1 |
| #N/A | LMRG_02395 | FIG00774842: hypothetical protein | 0 | 1 |
| lmo1558 | LMRG_01409 | GTP-binding protein EngB | 0 | 1 |
| lmo1755 | LMRG_02516 | Aspartyl-tRNA(Asn) amidotransferase subunit A (EC 6.3.5.6) @ Glutamyl-tRNA(Gln) amidotransferase subunit A (EC 6.3.5.7) | 0 | 1 |
| lmo1880 | LMRG_01027 | Ribonuclease HI, Bacillus nonfunctional homolog | 0 | 1 |
| lmo2066 | LMRG_01216 | FIG019766: hypothetical protein co-occurring with bile hydrolase | 0 | 1 |
| lmo2141 | LMRG_02799 | Acetyltransferase, GNAT family | 0 | 1 |
| lmo0224 | LMRG_02645 | Dihydropteroate synthase (EC 2.5.1.15) | 0 | 1 |
| lmo2269 | LMRG_01561 | hypothetical protein | 0 | 1 |
| lmo2301 | LMRG_01531 | putative terminase small subunit | 0 | 1 |
| lmo2308 | LMRG_02474 | Phage recombination protein Bet | 0 | 1 |
| lmo2325 | LMRG_02984 | Phage protein | 0 | 1 |
| lmo2328 | LMRG_01516 | hypothetical protein | 0 | 1 |
| #N/A | LMRG_01515 | FIG00774055: hypothetical protein | 0 | 1 |
| lmo2379 | LMRG_02724 | Na(+) H(+) antiporter subunit B | 0 | 1 |
| lmo2384 | LMRG_02729 | Na(+) H(+) antiporter subunit G | 0 | 1 |
| lmo2394 | LMRG_02915 | hypothetical protein | 0 | 1 |
| lmo2458 | LMRG_01790 | Phosphoglycerate kinase (EC 2.7.2.3) | 0 | 1 |
| #N/A | LMRG_02136 | CRISPR-associated protein Cas2 | 0 | 1 |
| lmo2619 | LMRG_02163 | SSU ribosomal protein S14p (S29e) @ SSU ribosomal protein S14p (S29e), zinc-dependent | 0 | 1 |
| lmo0324 | LMRG_00016 | hypothetical protein | 0 | 1 |
[truncated: 143,439 more chars]
